# Modulating Crossover Frequency and Interference for Obligate Crossovers in *Saccharomyces cerevisiae* Meiosis

**DOI:** 10.1534/g3.117.040071

**Published:** 2017-03-17

**Authors:** Parijat Chakraborty, Ajith V. Pankajam, Gen Lin, Abhishek Dutta, G. Nandanan Krishnaprasad, Manu M. Tekkedil, Akira Shinohara, Lars M. Steinmetz, K. Thazath Nishant

**Affiliations:** * School of Biology, Indian Institute of Science Education and Research, Thiruvananthapuram, Trivandrum 695016, India; † Genome Biology Unit, European Molecular Biology Laboratory, 69117 Heidelberg, Germany; ‡ Institute for Protein Research, Osaka University, 565-0871, Japan; § Department of Genetics, Stanford University, California 94305; ** Stanford Genome Technology Center, Palo Alto, California 94304; †† Center for Computation, Modelling and Simulation, Indian Institute of Science Education and Research, Thiruvananthapuram, Trivandrum 695016, India

**Keywords:** crossover frequency, crossover assurance, meiotic chromosome segregation, genetic interference, genome wide recombination map

## Abstract

Meiotic crossover frequencies show wide variation among organisms. But most organisms maintain at least one crossover per homolog pair (obligate crossover). In *Saccharomyces cerevisiae*, previous studies have shown crossover frequencies are reduced in the mismatch repair related mutant *mlh3*Δ and enhanced in a meiotic checkpoint mutant *pch2*Δ by up to twofold at specific chromosomal loci, but both mutants maintain high spore viability. We analyzed meiotic recombination events genome-wide in *mlh3*Δ, *pch2*Δ, and *mlh3*Δ *pch2*Δ mutants to test the effect of variation in crossover frequency on obligate crossovers. *mlh3*Δ showed ∼30% genome-wide reduction in crossovers (64 crossovers per meiosis) and loss of the obligate crossover, but nonexchange chromosomes were efficiently segregated. *pch2*Δ showed ∼50% genome-wide increase in crossover frequency (137 crossovers per meiosis), elevated noncrossovers as well as loss of chromosome size dependent double-strand break formation. Meiotic defects associated with *pch2∆* did not cause significant increase in nonexchange chromosome frequency. Crossovers were restored to wild-type frequency in the double mutant *mlh3*Δ *pch2*Δ (100 crossovers per meiosis), but obligate crossovers were compromised. Genetic interference was reduced in *mlh3*Δ, *pch2*Δ, and *mlh3*Δ *pch2*Δ. Triple mutant analysis of *mlh3*Δ *pch2*Δ with other resolvase mutants showed that most of the crossovers in *mlh3*Δ *pch2*Δ are made through the Mus81-Mms4 pathway. These results are consistent with a requirement for increased crossover frequencies in the absence of genetic interference for obligate crossovers. In conclusion, these data suggest crossover frequencies and the strength of genetic interference in an organism are mutually optimized to ensure obligate crossovers.

Meiosis is a reductional division that produces haploid gametes or spores from diploid progenitor cells. Ploidy reduction is achieved by one round of DNA replication, followed by two consecutive nuclear divisions (Meiosis I and II), producing four daughter cells (Roeder 1997). Crossovers promote the formation of chiasma which serves as a physical linkage between two homologs and opposes the spindle generated forces that pull apart the homolog pairs. This opposing set of forces provides the tension necessary to promote proper disjunction of homolog pairs at Meiosis I (Petronczki *et al.* 2003). Failure to maintain at least one crossover per homolog pair increases the probability of nondisjunction, resulting in aneuploid gametes (Serrentino and Borde 2012). Although crossovers are important for chromosome segregation, nonexchange chromosomes have been observed to segregate accurately forming viable gametes (Hawley *et al.* 1992; Davis and Smith 2003; Kemp *et al.* 2004; Newnham *et al.* 2010; Krishnaprasad *et al.* 2015).

Meiotic crossovers (and noncrossovers) are initiated in *S. cerevisiae* by 150–170 programmed double-strand breaks (DSBs) formed by an evolutionarily conserved topoisomerase-like protein Spo11 and other accessory proteins (reviewed in Neale and Keeney 2006). The frequency and distribution of crossovers on chromosomes is a tightly regulated process. Four known aspects of this regulation are crossover interference, obligate crossovers, crossover homeostasis, and crossover invariance. Crossover interference regulates the spatial patterning of crossovers, where the presence of a crossover prevents the formation of a second crossover in its vicinity. As a consequence, an even distribution of crossovers is ensured, and closely spaced crossovers are less frequently observed than expected from a random distribution (Berchowitz and Copenhaver 2010). An obligate crossover per homolog pair facilitates disjunction. The obligate crossover is not a specific type of crossover, but refers to the observation that process(es) that generate most of the crossovers result in at least one crossover per homolog pair (Barchi *et al.* 2008). The obligate crossovers are ensured in different organisms with different crossover frequencies. For example, organisms that have strong genetic interference like *Caenorhabditis elegans*, *Drosophila*, *Arabidopsis*, mouse, and humans ensure obligate crossovers, although the number of crossovers per bivalent pair is low (∼1–2) (Copenhaver *et al.* 2002; Hillers and Villeneuve 2003; Berchowitz and Copenhaver 2010; Libuda *et al.* 2013). But organisms with weaker interference, like *S. cerevisiae*, make ∼90 crossovers, or ∼6 times the number of bivalent pairs (16), to ensure an obligate event. Crossover homeostasis ensures that the number of crossovers is maintained (at the expense of noncrossovers) when the DSB precursors become limiting (Martini *et al.* 2006). This crossover buffering mechanism is also seen when the DSBs are increased (Cole *et al.* 2012). Crossover invariance is another mechanism that maintains the number of crossovers in organisms that lack interference. This phenomenon refers to the observation that, in *Schizosaccharomyces pombe*, the ratio of intersister to interhomolog repair is modulated in response to variations in DSB intensity to ensure uniform crossover distribution (Hyppa and Smith 2010). Recent studies suggest crossover interference, obligate crossovers, and crossover homeostasis are the outcomes of a single crossover patterning process regulated by the “mechanical stress relief” of chromosomes (Wang *et al.* 2015; Zickler and Kleckner 2016). As per this model, crossover interference and homeostasis are linked processes. But the designation of an obligate crossover is not dependent on either interference or homeostasis (Zhang *et al.* 2014; Wang *et al.* 2015).

The majority of the crossovers (Class I) in *S. cerevisiae* show interference, and are formed through a pathway involving the ZMM (Zip1, Zip2, Zip3, Zip4, Mer3, Spo16, and Msh4-Msh5) and STR (Sgs1, Rmi1, and Top3) procrossover factors and mismatch repair related proteins Exo1 and MutLγ (Mlh1-Mlh3) (Allers and Lichten 2001; Hunter and Kleckner 2001; Borner *et al.* 2004; Nishant *et al.* 2008; Shinohara *et al.* 2008; De Muyt *et al.* 2012; Zakharyevich *et al.* 2012). Cytological studies in the mouse have shown that the MutLγ complex acts downstream of Msh4-Msh5 (MutSγ) (Baker *et al.* 1996; Lipkin *et al.* 2002; Kolas *et al.* 2005). Further physical, genetic, and biochemical analysis in *S. cerevisiae* suggest MutLγ has an endonuclease activity that can generate crossovers from dHJs in a pathway comprising MutSγ, Exo1 and Sgs1 (Nishant *et al.* 2008; Hunter 2011; Zakharyevich *et al.* 2012; Rogacheva *et al.* 2014). Noninterfering crossovers are formed by a separate pathway involving the structure specific endonuclease Mus81-Mms4 (Class II crossovers). Mainly aberrant and multichromatid joint molecules are resolved by this pathway (de los Santos *et al.* 2003; Oh *et al.* 2008). The structure specific endonucleases Yen1 and Slx1-Slx4 have a minor role in meiotic crossover formation, which is observed in *mms4*Δ*/mms4*Δ *sgs1*Δ background (Zakharyevich *et al.* 2012; Higashide and Shinohara 2016). Genetic interference between crossovers is compromised in most ZMM mutants (*zip1*Δ, *mer3*Δ, *msh4*Δ, and *msh5*Δ) (Sym and Roeder 1994; Nakagawa and Ogawa 1999; Novak *et al.* 2001; Argueso *et al.* 2004; Zakharyevich *et al.* 2012), but the distribution of Zip3 foci (measured in physical distances), which mark DSB sites designated to be repaired as crossovers, show interference in *zip1*Δ and *msh4*Δ mutants (Zhang *et al.* 2014).

It is not clear why organisms have vastly different crossover frequencies and how this is related to ensuring an obligate crossover. In this study, we address the relationship of obligate crossovers with crossover frequency using *mlh3*Δ and *pch2*Δ mutants. In *S. cerevisiae*, both these mutants have very different crossover frequencies but show high spore viability. At specific chromosomal loci, *mlh3*Δ shows up to a 50% reduction in crossover frequency but with good spore viability ranging from 72% and up to 92% depending on the strain background (Nishant *et al.* 2008; Cotton *et al.* 2010; Sonntag Brown *et al.* 2013). Pch2 is an evolutionarily conserved hexameric ring AAA+ ATPase protein that has roles in meiotic checkpoint signaling triggered by unrepaired DSBs, remodeling the chromosome axis at the future crossover sites and interhomolog bias (San-Segundo and Roeder 1999; Borner *et al.* 2008; Ho and Burgess 2011; Zanders *et al.* 2011; Chen *et al.* 2014). *pch2*Δ mutants are observed to make 50% more crossovers than wild type at specific loci, and also have good viability (95%) (Zanders and Alani 2009; but see Joshi *et al.* 2009). The *mlh3*Δ and *pch2*Δ mutants are therefore useful to test the effect of a wide range of crossover frequencies on obligate crossovers. It is important to note that pleiotropic effects of *pch2*Δ could affect the interpretation of changes in crossover frequencies on the obligate crossover.

We performed genome-wide mapping of meiotic recombination events in *mlh3*Δ, *pch2*Δ and *mlh3*Δ *pch2*Δ using the S288c/YJM789 hybrid. Crossovers in all three mutants, *mlh3*Δ (64 crossovers per meiosis on average), *pch2*Δ (137 crossovers per meiosis on average) and *mlh3*Δ *pch2*Δ (100 crossovers per meiosis on average) showed reduced genetic interference. Noncrossovers were not affected in *mlh3*Δ, but increased in *pch2*Δ and *mlh3*Δ *pch2*Δ. Crossover and noncrossover data suggest loss of chromosome size dependent DSB formation in *pch2*Δ mutants. *mlh3*Δ *pch2*Δ showed uniform crossover density on all chromosomes consistent with loss of genetic interference. Nonexchange chromosomes were abundant in *mlh3∆* (47% of meioses), but efficiently segregated. *pch2*Δ (7%) had a reduced percentage of meioses with nonexchange chromosomes, showing that high crossover frequencies can promote crossover assurance in the absence of genetic interference. Even though the *mlh3*Δ *pch2*Δ mutant made as many crossovers as wild type, 20% of meioses showed nonexchange chromosomes, consistent with noninterfering crossovers being inefficient in promoting crossover assurance.

## Materials and Methods

### Media and strains

All *S. cerevisiae* strains used in this study (S288c, YJM789, and SK1) were grown on either YPD (yeast extract, peptone, and dextrose) or synthetic complete medium at 30° (Rose *et al.* 1990). The drugs, geneticin (Invitrogen), nourseothricin (Werner BioAgents), and hygromycin (HiMedia) were added to YPD as described in Goldstein and McCusker (1999). Sporulation media for the genetic analysis was prepared as described in Argueso *et al.* (2004). For whole genome recombination analysis, S288c and YJM789 strains were used. For tetrad analysis and cytology, the SK1 strains, EAY1108/EAY1112, NHY1168/NHY1162, and their derivatives, were used (Argueso *et al.* 2004; Martini *et al.* 2006). All strains were transformed using standard techniques (Gietz *et al.* 1995). The *mlh3*Δ::*kanMX4* strains were created using plasmid pEAI168. *pch2*Δ::*natMX4*, *slx4*Δ::*kanMX4* and *mlh3*Δ::*hphMX4* were made using deletion constructs amplified by PCR. The meiotic depleted allele of *MMS4* (*mms4-md*) in EAY1108/EAY1112 was created by transformation with the *kanMX-pCLB2-3HA-MMS4* PCR product generated from the haploid parents of MJL3172 (De Muyt *et al.* 2012). Strain information is given in Supplemental Material, Table S1.

### Tetrad analysis

Diploids were sporulated following the zero growth mating protocol (Argueso *et al.* 2003). Haploid strains were mixed together on synthetic complete media. After incubation for 4 hr at 30°, the resulting diploids were patched on sporulation media. After 2 d of incubation at 30°, tetrads were dissected on synthetic complete medium using a Zeiss dissection microscope. The dissected tetrads were grown for 2 d, after which they were replica plated to various selective media. The replica plates were scored after 1 d of incubation. Marker segregation was analyzed using the recombination analysis software RANA (Argueso *et al.* 2004).

### Meiotic time course

Wild type and *pch2*Δ diploids were streaked on YPD media, and a single colony was inoculated in 4 ml YPD and grown overnight at 30°. To obtain synchronized meiosis, cultures were grown in presporulation medium (SPS) (0.5% yeast extract, 1% peptone, 0.67% yeast nitrogen base, 1% potassium acetate, and 0.05 M potassium biphthalate) before transfer to the sporulation medium (2% potassium acetate, amino acids, and 0.001% polypropylene glycol) (Murakami *et al.* 2009).

### Cytology

Meiotic cultures (5 ml) of wild type and *pch2*Δ from 2 to 8 hr postinduction into meiosis were collected at hourly intervals. Cultures were treated with Zymolyase 100T to obtain spheroplasts. Spheroplasts were washed and resuspended in MES/sorbitol solution (1 M sorbitol, 1 mM EDTA, 0.5 mM MgCl_2_, 0.1 M MES pH 6.5). Chromosome spreads were prepared on ethanol washed glass slides, using detergent (1% Lipsol) and 4% paraformaldehyde/3.4% sucrose as fixative. Slides were dried overnight and immunostained as described previously (Bishop 1994; Shinohara *et al.* 2000).

### Analysis of Rad51 foci

Slides were stained with rabbit anti-Rad51 antibody (1:500) (a gift from Dr. Akira Shinohara), incubated overnight at 4°, followed by 2 hr incubation with secondary antibody anti-rabbit TRITC (1:1500) (Jackson ImmunoResearch). Immunofluorescence images of Rad51 foci were captured using 100× oil immersion objective on a Leica SP5 confocal microscope. Images were captured at 0.25 μm interval Z sections. Maximum intensity images were produced by projecting the optical sections (Z stacks). For each time point 100 images were analyzed in three independent experiments. Image analysis and 3D deconvolution was done using Leica Application Suite (LAS) Advanced Fluorescence (AF) Lite 2.8.0 software.

### DNA extraction and whole genome sequencing of meiotic spores

Spore colonies from the four spore viable tetrads of *mlh3∆*, *pch2∆*, and *mlh3∆ pch2∆* were grown overnight at 30° in 4 ml YPD broth. DNA was isolated from the culture using PrepEase DNA isolation kit (Affymetrix). Whole genome sequencing on Illumina platform was performed at Fasteris, Switzerland, as well as at the European Molecular Biology Laboratory (EMBL) Genomics Core Facilities (GeneCore), Heidelberg, Germany.

### Annotation of recombination events

The raw reads were demultiplexed using the NGS QC Toolkit (version 2.3.3, http://www.nipgr.res.in/ngsqctoolkit.html), and processed for quality control (QC) using the trimmomatic tool (Version 0.36, http://www.usadellab.org/cms/index.php?page=trimmomatic) (Bolger *et al.* 2014). These QC filtered high quality reads were then mapped to the *S. cerevisiae* genome (version R64-1-1, 2011) using bowtie 2 (version 2.2.6) (Langmead and Salzberg 2012). Only uniquely mapped reads were considered for variant calling using Picard tools. Local realignment around indels was performed to reduce misalignment. Genotyping was done using GATK (UnifiedGenotyper, version 3.4-46) (McKenna *et al.* 2010; DePristo *et al.* 2011). From the alignment, an average of 59,215 SNPs was genotyped. Recombination events, such as crossovers, noncrossovers (type 0 gene conversions exhibiting 1:3 or 3:1 segregation of SNP markers), crossover independent gene conversions (type 0, 2, 3, and 4 gene conversions), and crossover associated gene conversions were annotated using the CrossOver program (v6.3) of the ReCombine program suite (v2.1) (Anderson *et al.* 2011). Parameters used for the CrossOver program were as described previously in Krishnaprasad *et al.* (2015). Custom R scripts were used to convert vcf files to segregation files (input file for the CrossOver program), to generate plots from the CrossOver program output and to perform various statistical tests. Detection of copy number variation was performed as described in Krishnaprasad *et al.* (2015).

### Interference analysis

Interference analysis using the one pathway gamma model was performed as described previously (McPeek and Speed 1995; Krishnaprasad *et al.* 2015). For the two pathway analysis of interference, crossover distances between each crossover per chromosome was calculated for each tetrad, and converted to centimorgans. A gamma mixture model was fitted using an EM algorithm similar to that in the R package mixtools (Benaglia *et al.* 2009). The number of mixture components was fixed at two and the shape parameter for one of the components (Class II crossovers) was fixed at one. For the Coefficient of Coincidence (CoC) analysis, the genome was partitioned into 25 kb bins. The frequency of crossover events in each bin was calculated along with the expected and observed frequencies of double crossovers in all pairs of bins. The CoC (observed crossover frequency/expected crossover frequency) was analyzed for all bin pairs (with nonzero expected frequency) separated by a given interval (in kilobase) and averaged. Chi-square test was used to measure the statistical significance of observed and expected double crossover frequencies between wild type and the mutants.

### Data availability

All strains listed in Table S1 are available upon request. Sequence data are available from the National Centre for Biotechnology Information Sequence Read Archive: Accession number SRP082254. The raw recombination data files and the custom R scripts are available online at the Dryad data repository (http://datadryad.org; http://dx.doi.org/10.5061/dryad.5vm4g).

## Results

### Genome-wide recombination maps for mlh3**Δ**, pch2**Δ**, and mlh3**Δ** pch2**Δ** by whole genome sequencing

*mlh3*Δ, *pch2*Δ, and *mlh3*Δ *pch2*Δ mutations were introduced in the S288c and YJM789 strains. Spore viability of *mlh3*Δ (85%) was similar to the wild-type S288c/YJM789 hybrid (Figure S1 and Table 1). Spore viability of *pch2*Δ (75%), in the S288c/YJM789 hybrid, was reduced compared to wild-type hybrid (*P* = 0.0001, Fischer’s exact test) (Figure S1 and Table 1). The *mlh3*Δ *pch2*Δ mutant also showed significantly less viability compared to wild type (59%, *P* = 7.9 × 10^−21^, Fischer’s exact test). We performed whole genome sequencing of spores from 54 four-viable spore tetrads of the S288c × YJM789 hybrid bearing *mlh3*Δ, *pch2*Δ, and *mlh3*Δ *pch2*Δ mutations (Table S2) (sequence data available from National Centre for Biotechnology Information Sequence Read Archive under accession number: SRP082254). High-resolution genome-wide recombination data were generated in *mlh3*Δ, *pch2*Δ, and *mlh3*Δ *pch2*Δ mutants by analyzing segregation of SNPs in the 54 tetrads (File S1). These include 19 tetrads in *mlh3*Δ, 15 tetrads in *pch2*Δ and 20 tetrads in *mlh3*Δ *pch2*Δ. Genome-wide recombination data for wild type were generated from 66 tetrads by combining previously published studies (Mancera *et al.* 2008; Krishnaprasad *et al.* 2015). Crossover and noncrossover counts for each of the 120 tetrads are shown in Table S3.

**Table 1:** Crossover (CO), noncrossover (NCO) counts and spore viability of *mlh3***Δ**, *pch2***Δ**, *mlh3***Δ** *pch2***Δ** mutants in the S288c/YJM789 hybrid

Genotype	*N*	S.V%	Tetrads Genotyped	Average CO Counts ± SD (Median)	Average NCO Counts ± SD (Median)
S288c × YJM789	180	84	66*^a^*	93.4 ± 10.86 (94)	46 ± 15.6 (43)
S288c × YJM789 *mlh3*Δ	120	85	19	64.4 ± 8.5 (62)	49.5 ± 11.6 (45)
S288c × YJM789 *pch2*Δ	164	75	15	136.5 ± 25.6 (134)	85.9 ± 25.9 (91)
S288c × YJM789 *mlh3*Δ *pch2*Δ	120	59	20	99.8 ± 21.6 (96)	93.6 ± 27.6 (86)

Spore viability (S.V) data for wild type is from Krishnaprasad *et al.* (2015). *N*, number of tetrads analyzed for spore viability.

a Merged data set from Krishnaprasad *et al.* (2015) and Mancera *et al.* (2008).

### Crossover defects in mlh3**Δ** are distinct from msh4**Δ**

**Figure 1:**
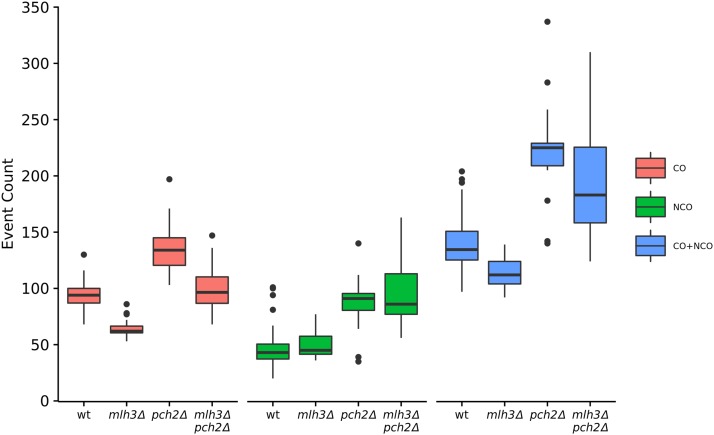
Crossover, noncrossover and total events per meiosis for wild type, *mlh3*Δ, *pch2*Δ, and *mlh3*Δ *pch2*Δ. The box plots show minimum, first quantile, median, third quantile, and maximum count.

The *mlh3*Δ mutant showed a significant genome-wide reduction in crossovers compared to wild type (64 crossovers on average in *mlh3*Δ *vs.* 93 in wild type, *P* = 1.7 × 10^−14^, *t*-test) (Figure 1 and Table 1). These results are consistent with crossover data obtained from analysis of specific loci in *mlh3*Δ (Nishant *et al.* 2008; Sonntag Brown *et al.* 2013). Genome-wide crossover defects are therefore stronger in *msh4*Δ (average of 49 crossovers per meiosis) compared to *mlh3*Δ (*P* = 2.3 × 10^−3^, *t*-test), although the Msh4-Msh5, Mlh1-Mlh3 proteins are thought to act in the same pathway for making crossovers (Chen *et al.* 2008; Krishnaprasad *et al.* 2015). Previous work by Sonntag Brown *et al.* (2013) also showed stronger crossover defects in *msh5*Δ (37 cM) compared to *mlh3*Δ (54.5 cM) for chromosome XV in the SK1 EAY1108/EAY1112 background. Average noncrossover counts in *mlh3*Δ (50 per meiosis) were similar to wild type (46 per meiosis) (*P* = 0.29, *t*-test). Since the crossover defects in *mlh3*Δ were not associated with a concomitant increase in noncrossovers, perhaps in the absence of the Mlh3 protein DSBs that were not repaired as crossovers may be repaired using the sister chromatid.

**Figure 2:**
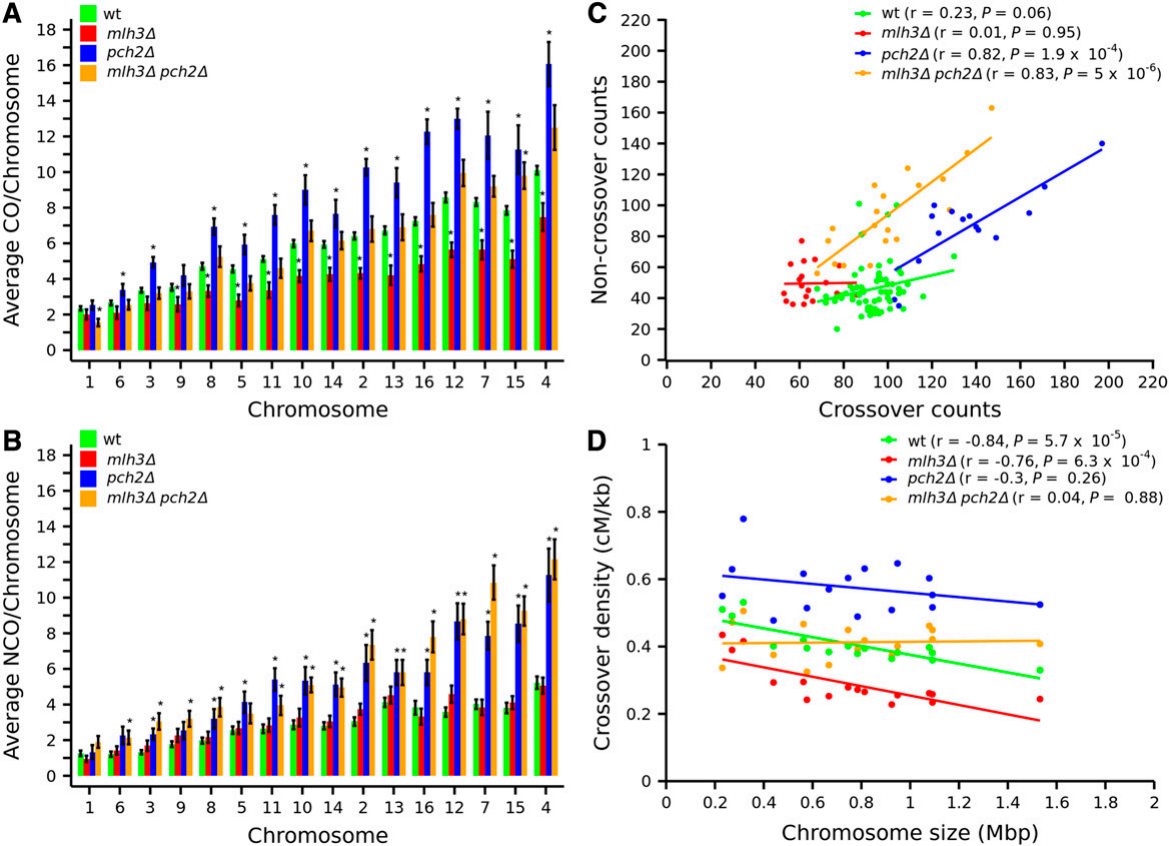
Crossover and noncrossover distribution on chromosomes for wild type, *mlh3*Δ, *pch2*Δ, and *mlh3*Δ *pch2*Δ. (A, B) Bar plot showing average crossover and noncrossover counts per chromosome for wild type, *mlh3*Δ, *pch2*Δ, and *mlh3*Δ *pch2*Δ. The asterisk symbol (*) shows chromosomes with significant difference (*P* < 0.05, *t*-test) in crossover or noncrossover counts compared to wild type. Chromosomes are arranged according to size from left to right. Error bars are “mean ± SE.” (C) Crossover *vs.* noncrossover scatter plot with correlation coefficient (*r*) and *P* values for wild type, *mlh3*Δ, *pch2*Δ, and *mlh3*Δ *pch2*Δ. (D) Crossover density (centimorgans per kilobase) plotted as a function of chromosome length (megabase) for wild type, *mlh3*Δ, *pch2*Δ, and *mlh3*Δ *pch2*Δ along with the correlation coefficient (*r*) and *P* values.

The effects of chromosome size on crossover distribution were analyzed using average crossover counts per chromosome (Figure 2A and Table S4). The *mlh3*Δ mutant showed a significant decrease in crossovers on medium and large chromosomes (Figure 2A and Figure S2A). Crossovers on small chromosomes also showed a decrease in *mlh3*Δ, but these were not significant except for chromosome IX. Crossover defects in *mlh3*Δ are therefore different from *msh4*Δ and *msh4-R676W*, which had significant crossover reduction on all (small, medium, and large) chromosomes (Krishnaprasad *et al.* 2015). Noncrossover counts in *mlh3*Δ did not show significant differences on any of the chromosomes compared to wild type (Figure 2B and Figure S2D). The noncrossover data in *mlh3∆* are different from *msh4*Δ, where average noncrossover counts were increased on several chromosomes (Krishnaprasad *et al.* 2015). When the noncrossover counts are regressed against the crossover counts, we observed no correlation in wild type and *mlh3*Δ (wild type, *r* = 0.23, *P* = 0.06; *mlh3*Δ, *r* = 0.01, *P* = 0.95; Figure 2C).

Wild-type cells show variation in crossover density (centimorgan per kilobase) with chromosome size (Figure 2D, and see Pan *et al.* 2011). Previous studies have shown that high crossover density on small chromosomes is mostly a consequence of higher DSB density on small chromosomes (Pan *et al.* 2011). We plotted crossover density *vs.* chromosome length for wild type, and observed a pattern similar to that observed for DSB density (Figure S3, and see Pan *et al.* 2011). First, the crossover density decreases with chromosome size, indicating that larger chromosomes have fewer crossovers than expected for their physical length (assuming crossover density is equal on all chromosomes). Second, if we take into account this inverse relationship between chromosome length and crossover density, we observed that the smaller chromosomes deviate a lot from the regression line, indicating that they have a much higher crossover density than expected (Figure S3). *mlh3*Δ showed a significant negative correlation coefficient (*r* = −0.76, *P* = 6.3 × 10^−4^), implying an inverse relation between crossover density and chromosome size (Figure 2D). The correlation coefficient for *mlh3*Δ was comparable to wild type (*r* = −0.84, *P* = 5.7 × 10^−5^). Crossover density was higher on smaller chromosomes in wild type and *mlh3*Δ, even when the analysis excluded the three smallest chromosomes (I, III, and VI), which have the highest DSB densities (Figure S4A, and see Sun *et al.* 2015). Noncrossover density, as well as crossover plus noncrossover density, also showed a significant negative correlation with chromosome size in wild type and *mlh3*Δ (Figure S4, B–D). The relationship between crossover or noncrossover density and chromosome size was therefore similar between wild type and *mlh3*Δ. These results suggest the regulation of DSB distribution based on chromosome size is intact in *mlh3*Δ like in wild type. Further, since most crossovers in wild type and *mlh3*Δ are Class I and Class II, respectively, these results suggest the chromosome size effect seen for crossover density variation is dependent on DSB density, and not on the recombination pathway as shown previously (Pan *et al.* 2011). It is important to note that using crossover and noncrossover density as a proxy for DSBs does not consider the effect of any changes in homolog bias in these mutants. Crossover and noncrossover distributions at centromere and telomere regions were comparable to wild type, but noncrossovers were elevated within 10 kb of centromere region in *mlh3*Δ (File S2). Chromosome-wide crossover distribution for *mlh3*Δ is shown in Figure S5.

### Genome-wide increase in crossovers and noncrossovers in pch2**Δ**

Average crossovers in *pch2*Δ (137) were significantly more than wild type (*P* = 1.15 × 10^−5^, *t*-test) (Figure 1). The increase in crossovers was statistically significant on all the medium and large chromosomes, and on two of the smaller chromosomes (III and VI) (Figure 2A and Figure S2B). This result is consistent with increased crossovers observed at specific loci on medium and large chromosomes in SK1 background (Zanders and Alani 2009), but different from Joshi *et al.* (2009) who observed no increase in crossovers across nine intervals on three chromosomes. The increase in crossovers on chromosome III is different from the results observed by Zanders and Alani (2009) and San-Segundo and Roeder (1999). Such differences may be because of the limited number of chromosomal loci analyzed in the previous studies (San-Segundo and Roeder 1999; Joshi *et al.* 2009; Zanders and Alani 2009). Noncrossovers in *pch2*Δ (average of 86 per meiosis) were also significantly higher than wild type (46, *P* = 2.9 × 10^−5^, *t*-test) (Figure 1 and Table 1). Similar to the trend seen for crossovers, increased noncrossovers in *pch2∆* were significant for all medium and large chromosomes (Figure 2B and Figure S2E). These results suggest that specific regulation of DSB formation on medium and large chromosomes is affected in *pch2∆*. Among the small chromosomes, Chromosome III was exceptional in showing an increase in both crossovers and noncrossovers. This might be due to an enhanced distribution of DSBs on chromosome III in *pch2*Δ mutants. An increase in DSB formation at the *HIS4*::*LEU2* locus has been observed previously in *pch2*Δ (Farmer *et al.* 2012). Interestingly Borner *et al.* (2008) reported increased noncrossovers and decreased crossovers for the *HIS4*::*LEU2* locus on chromosome III for *pch2*Δ. When the noncrossover counts are regressed against the crossover counts, we observed a well correlated linear relationship (Figure 2C). *pch2*Δ cells with more crossovers also had more noncrossovers (*r* = 0.82, *P* = 1.9 × 10^−4^). Although *pch2*Δ showed a negative correlation coefficient (*r* = −0.30) between crossover density and chromosome size, it was not significant (*P* = 0.26), consistent with the enhanced crossovers on large and medium chromosomes (Figure 2D). Unlike wild type, *pch2*Δ did not show a significant negative correlation between chromosome size and crossover density, noncrossover density, as well as crossover plus noncrossover density (Figure S4).

Crossover and noncrossover distributions within 10 kb of the centromere were comparable between wild type and *pch2*Δ (File S2). But crossover and noncrossover distributions in *pch2*Δ were suppressed for an extended distance from the telomere (up to 80 kb) compared to the wild type (File S2). These differences may reflect changes in DSB distribution near telomeres. Chromosome-wide analysis of crossover distribution showed distinct crossover peaks compared to wild type, including elevated crossovers around the rDNA locus (Figure S6). This is consistent with the observation of increased DSBs and recombination at the outermost rDNA repeats in *pch2*Δ and the role of Pch2 in maintaining the integrity of the rDNA cluster (Vader *et al.* 2011).

**Figure 3:**
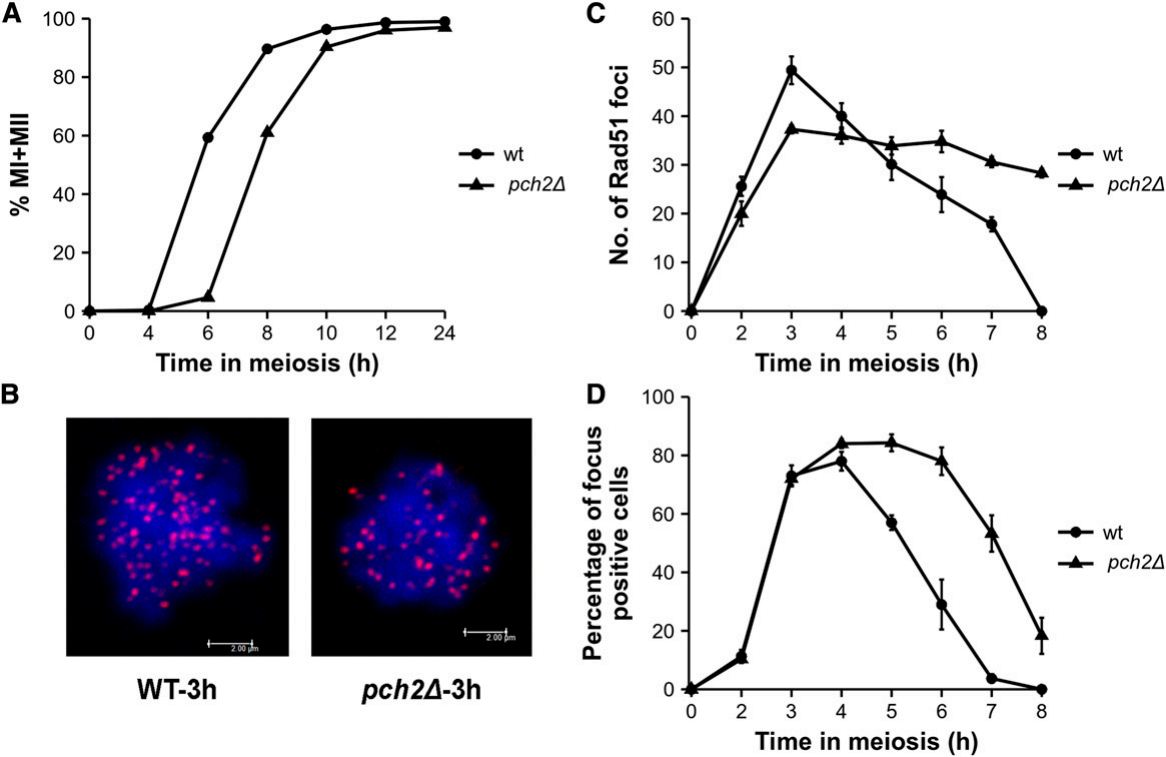
Rad51 foci analysis in wild type and *pch2*Δ mutants. (A) Meiotic time course assay for wild type and *pch2*Δ. Data are averaged across three independent meiotic time courses. (B) Representative image of Rad51 foci in wild type and *pch2*Δ at the 3 hr time point. (C) Average Rad51 foci counts with SE from three independent experiments in wild type and *pch2*Δ. Rad51 foci from 100 images were counted for each time point and each experiment. (D) Percentage of cells in a field that are positive for Rad51 foci at each time point. Cells with >5 Rad51 foci are counted as positive for Rad51 foci. Data are averaged across three independent experiments and plotted with SE measurements.

Genome-wide recombination analysis showed a correlated increase in both crossovers and noncrossovers in *pch2*Δ (Figure 1 and Figure 2C). The simplest explanation is that *pch2*Δ makes more DSBs. Rad51 focus analysis can provide information on whether *pch2*Δ mutants show increased DSBs. We measured Rad51 focus counts in wild type and *pch2*Δ mutants in three independent meiotic time courses to determine changes in cellular DSB frequency. *pch2*Δ mutants showed a 2 hr delay in meiotic divisions, as observed previously (Wu and Burgess 2006; Zanders and Alani 2009) (Figure 3A). We analyzed Rad51 foci in 1 hr increments from 2 to 7 hr time points in wild type, and from 2 to 8 hr time points in *pch2*Δ. For wild type and *pch2*Δ, maximum Rad51 foci were detected at 3 hr, and representative images are shown in Figure 3B. The maximum (average) Rad51 focus counts for *pch2*Δ (37) were reduced compared to wild type (49). However, unlike wild type, where the Rad51 foci peaked at 3 hr and could not be detected after 7 hr, *pch2*Δ showed a gradual decline of Rad51 foci over an extended time from 3 hr until 8 hr (Figure 3C). In wild type, the percentage of cells that show Rad51 foci was negligible (3.7%) at 7 hr, while, in *pch2*Δ, almost 50% of the cells showed Rad51 foci (Figure 3D). The persistent presence of DSBs may be due to ongoing formation and repair of new DSBs (turnover), which may explain the increased crossovers and noncrossovers observed in *pch2*Δ. In addition, such a pattern may be caused by delayed repair of DSBs, which may cause more DSBs, and, in turn, result in more crossovers and noncrossovers (Hochwagen *et al.* 2005; Borner *et al.* 2008). The cytological observations are consistent with a prophase delay in *pch2*Δ for a variable amount of time, during which additional DSBs are made asynchronously, so that there is a much greater range in the total number of crossover and noncrossover events (Figure 1, Figure 2C, and Table 1). Persistent DSBs in the *pch2∆* mutant have been observed previously at the *HIS4*::*LEU2* locus by Southern blot analysis, and also from analysis of Rad51 foci in *pch2*Δ *ndt80*Δ mutants (Hochwagen *et al.* 2005; Subramanian *et al.* 2016).

### The mlh3**Δ** pch2**Δ** mutant has wild-type crossover frequency and uniform crossover density on all chromosomes

Since *pch2*Δ shows increased crossovers in wild-type background as well as in crossover mutants like *msh5*Δ and *mms4*Δ (Zanders and Alani 2009), we were curious to see if a *pch2*Δ mutation in the *mlh3*Δ background would restore crossovers to wild-type frequencies. *mlh3*Δ *pch2*Δ made, on average, 100 crossovers per meiosis, comparable to the wild-type S288c × YJM789 hybrid (Figure 1 and Table 1) (*P* = 0.22, *t*-test). None of the chromosomes (except chromosomes I and XV) in *mlh3*Δ *pch2*Δ showed significant differences in crossovers compared to wild type (Figure 2A and Figure S2C). Noncrossovers in *mlh3*Δ *pch2*Δ (average of 94 per meiosis) were significantly more than wild type (*P* = 1.84 × 10^−7^, *t*-test) and comparable to *pch2*Δ (*P* = 0.40, *t*-test). These results suggest most of the increase in noncrossovers in *pch2*Δ mutants comes from the activity of the structure-specific endonucleases, which are less biased toward resolution of the dHJs into crossovers. The increase in noncrossovers was observed across small (VI, III, and IX), and all medium and large chromosomes (except V) (Figure 2B and Figure S2F). Similar to the pattern observed in *pch2*Δ, crossover, and noncrossover counts in *mlh3*Δ *pch2*Δ were positively correlated (*r* = 0.83, *P* = 5 × 10^−6^) (Figure 2C).

The crossover density in *mlh3*Δ *pch2*Δ was uniform across all chromosome sizes and the correlation coefficient (*r* = 0.04, *P* = 0.88) was close to zero (Figure 2D). A similar lack of correlation was seen for crossover, noncrossover, and crossover plus noncrossover density with chromosome size (including/excluding the smallest chromosomes I, III, and VI) (Figure S4). Like *pch2*Δ, crossover and noncrossover distributions were suppressed for an extended distance from the telomere (up to 80 kb) in *mlh3*Δ *pch2*Δ (File S2). Chromosome-wide distribution showed distinct crossover peaks in *mlh3*Δ *pch2*Δ compared to wild type, especially around the rDNA locus (Figure S7).

### Enhanced gene conversion frequencies and long gene conversion tracts in pch2**Δ** mutants

Most markers (>97.5%) showed 2:2 segregation in wild type and *mlh3*Δ mutant (Table S5). *pch2*Δ mutants showed higher gene conversion frequencies than wild type or *mlh3*Δ mutant. The percentage of SNP markers segregating 2:2 was 94.7% in *pch2∆*, compared to >97.5% in wild type and *mlh3*Δ. Like the *pch2*Δ single mutant, the *mlh3*Δ *pch2*Δ mutant also showed higher gene conversion frequencies than wild type (94% of markers segregating 2:2). Increases in gene conversion frequencies in *pch2*Δ at specific loci have been previously observed by Zanders and Alani (2009) and Joshi *et al.* (2009). The median gene conversion tract lengths associated with crossovers (2.4, 2.7, and 3.9 kb) were significantly longer than the noncrossover gene conversion tract lengths (1.7, 1.8, and 2.2 kb) for the *mlh3*Δ, *pch2*Δ, and *mlh3*Δ *pch2*Δ mutants (Figure S8, A–C and Table S5). These results are similar to observations in wild type and other crossover mutants (Terasawa *et al.* 2007; Chen *et al.* 2008; Mancera *et al.* 2008; Oke *et al.* 2014; Krishnaprasad *et al.* 2015). Both crossover- and noncrossover-associated gene conversion tract lengths were significantly longer in *mlh3*Δ, *pch2*Δ, and *mlh3*Δ *pch2*Δ compared to wild type (Figure S8, A–C and Table S5). In addition, long gene conversion tracts associated with crossovers (>4 kb) and noncrossovers (>2 kb) were over-represented in *pch2*Δ and *mlh3*Δ *pch2*Δ compared to wild type and *mlh3*Δ (Figure S8, B and C, see *Discussion*).

### Most crossovers in mlh3**Δ** pch2**Δ** are made by the Mus81-Mms4 pathway

**Figure 4:**
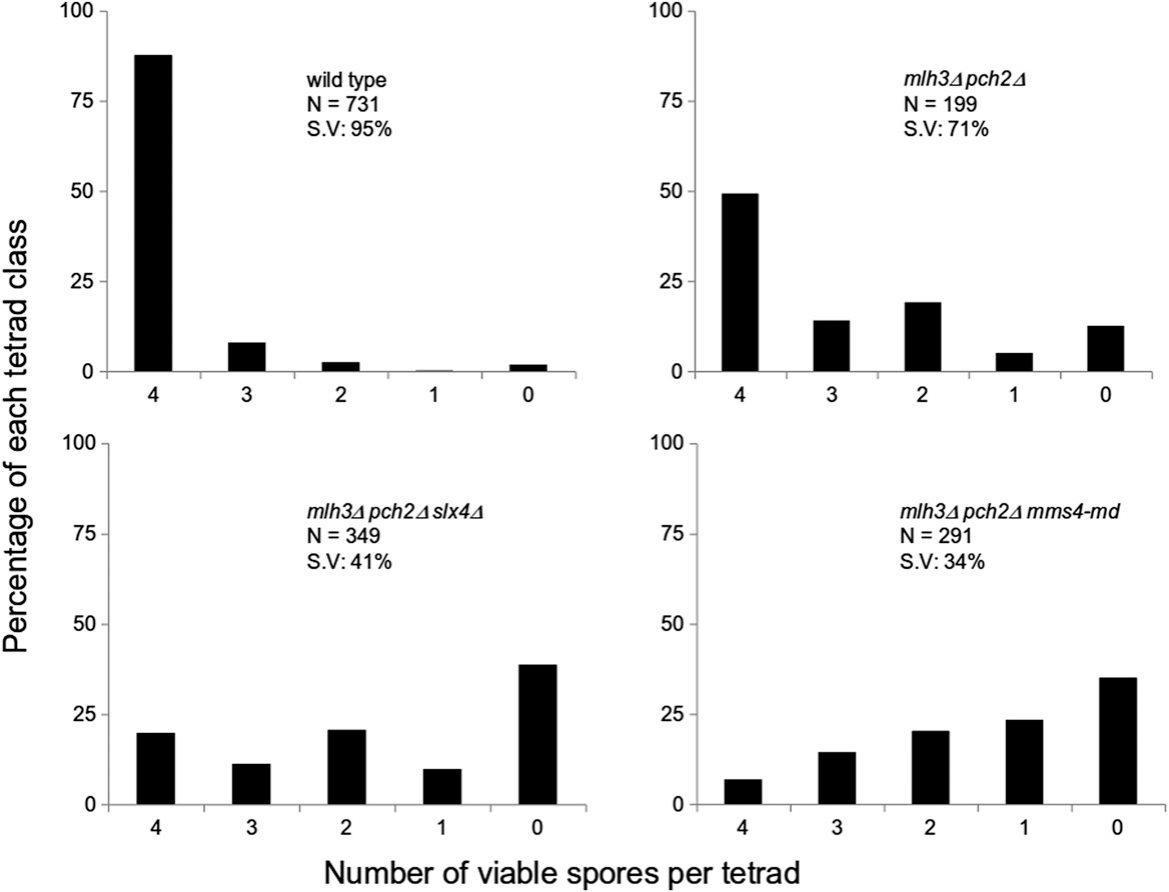
Spore viability analysis of wild type, *mlh3*Δ *pch2*Δ, *mlh3*Δ *pch2*Δ *slx4*Δ, and *mlh3*Δ *pch2*Δ *mms4-md* in the EAY1108/EAY1112 genetic background. The viable spores per tetrad (*x* axis) and the percentage of each tetrad class (*y* axis) are shown. *N*, number of tetrads dissected; S.V, percentage spore viability.

**Figure 5:**
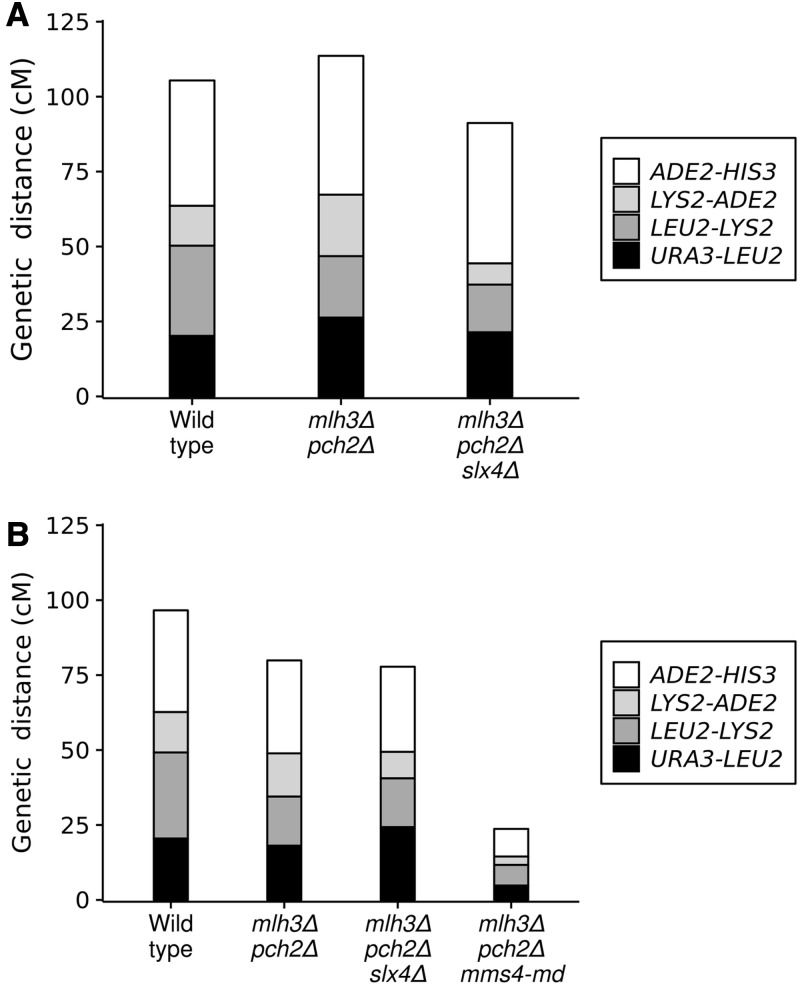
Crossovers in *mlh3*Δ *pch2*Δ are made predominantly by the Mus81-Mms4 pathway. Genetic map distances for complete tetrads (A) and total spores (B) are shown for a set of four intervals on chromosome XV in the EAY1108/EAY1112 genetic background. Raw data are shown in Table S6 and Table S7 for complete tetrads and total spores, respectively.

Although *mlh3*Δ has ∼30% reduction in crossovers, *mlh3*Δ *pch2*Δ has wild-type crossover frequency. In *S. cerevisiae*, the major pathway for resolving joint molecules into crossovers involves Mlh1-Mlh3/Exo1 proteins (De Muyt *et al.* 2012; Zakharyevich *et al.* 2012). Joint molecules are also resolved into crossovers by minor pathways involving the structure selective nucleases Mus81-Mms4, Yen1, and Slx1-Slx4 (De Muyt *et al.* 2012; Zakharyevich *et al.* 2012). Yen1 is thought to process joint molecules that escape Mus81-Mms4 (Matos *et al.* 2011). We were curious to know how wild-type frequency of crossovers is possible in *mlh3*Δ *pch2*Δ in the absence of the major resolvase (Mlh3). We tested the role of the two joint molecule resolvases, Mus81-Mms4 and Slx1-Slx4, in making crossovers in the *mlh3*Δ *pch2*Δ background. For testing the role of Mus81-Mms4, a meiotic depleted allele of *MMS4* (*mms4-md*) was used (De Muyt *et al.* 2012). Spore viability and genetic map distances of *mlh3*Δ *pch2*Δ *mms4-md*, and *mlh3*Δ *pch2*Δ *slx4*Δ, were compared with *mlh3*Δ *pch2*Δ in the EAY1108/EAY1112 background (Argueso *et al.* 2004). *mlh3*Δ *pch2*Δ has 71% spore viability in the EAY1108/EAY1112 background and has a spore viability pattern (4, 2, and 0 viable spore tetrad class >3 and 1 viable spore tetrad class) suggesting Meiosis I nondisjunction (Figure 4). Introduction of *mms4-md* and *slx4*Δ mutations reduced the spore viability to 34% and 41%, respectively (Figure 4). Genetic map distances in four intervals in *mlh3*Δ *pch2*Δ (113.6 cM), based on four-viable spore tetrads was similar to wild type (105.4 cM), in agreement with the whole genome sequencing data from the S288c/YJM789 hybrid (Figure 1 and Figure 5A). However, map distances when all viable spores were considered showed a reduction in *mlh3*Δ *pch2*Δ (79.9 cM) relative to wild type (96.6 cM) (Figure 5B). These results suggest restoration of crossovers in *mlh3*Δ *pch2*Δ to wild-type frequency occurs through an increase in both single crossovers measured as tetratypes (TT), as well as double crossovers measured as nonparental ditypes (NPDs). The increase in double crossovers (NPDs) contributes to map distances from tetrad data but not from spore data (Table S6 and Table S7). More NPD tetrads are also observed in *pch2*Δ mutants relative to wild type (Joshi *et al.* 2009; Zanders and Alani 2009).

*mlh3*Δ *pch2*Δ *slx4*Δ (91.2 cM) showed reduced genetic map distances compared to *mlh3*Δ *pch2*Δ (113.6 cM) in measures based on complete tetrads, but the genetic map distances from spore data were similar: 77.8 cM in *mlh3*Δ *pch2*Δ *slx4*Δ compared to 79.9 cM in *mlh3*Δ *pch2*Δ (Figure 5, A and B and Table S6, and Table S7). These results suggest Slx1-Slx4 does not significantly contribute to increased crossovers in *mlh3*Δ *pch2*Δ except for contributing to the NPD class. This is consistent with the meiotic role of the Slx1-Slx4 complex in the non-ZMM pathway characterized by multi-chromatid joint molecules (Zakharyevich *et al.* 2012; Kaur *et al.* 2015). *mlh3*Δ *pch2*Δ *mms4-md* mutants showed strong reduction in genetic map distances (23.7 cM) compared to *mlh3*Δ *pch2*Δ (79.9 cM) (Figure 5B and Table S7). Map distances from tetrad data could not be accurately estimated in *mlh3*Δ *pch2*Δ *mms4-md* due to the low frequency of four-spore viable tetrads and the strong reduction in the map distances. These results suggest that the majority of the crossovers in *mlh3*Δ *pch2*Δ are made through the Mus81-Mms4 pathway.

### Genetic interference is reduced across a range of crossover frequencies in the mlh3**Δ**, pch2**Δ**, and mlh3**Δ** pch2**Δ** mutants

**Figure 6:**
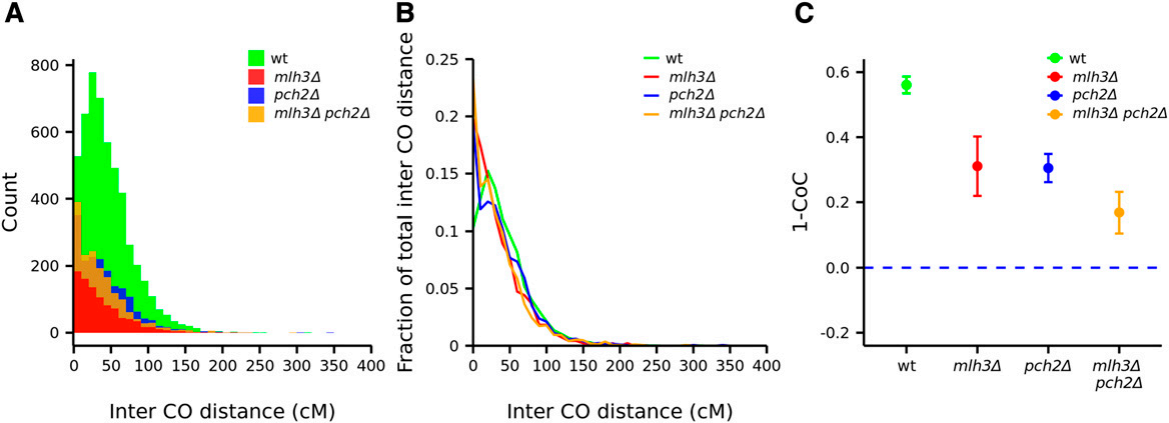
Analysis of crossover interference in wild type, *mlh3*Δ, *pch2*Δ, and *mlh3*Δ *pch2*Δ. (A) Histogram showing actual count of intercrossover distances. (B) Line plot showing intercrossover distances as a fraction of the total for wild type, *mlh3*Δ, *pch2*Δ, and *mlh3*Δ *pch2*Δ. (C) Interference (1–CoC) in wild type (0.56), *mlh3*Δ (0.31), *pch2*Δ (0.30), and *mlh3*Δ *pch2*Δ (0.17) plotted for 0–25 kb adjacent intervals.

Interference limits crossover number and ensures that multiple crossovers along the chromosome are widely spaced (Muller 1916; Hillers 2004; Kleckner *et al.* 2004; Stahl *et al.* 2004). Intercrossover distances modeled using a gamma distribution can be used to determine the strength of genetic interference: *γ* = 1 corresponds to no interference, while *γ* > 1 indicates positive interference (Anderson *et al.* 2011). Intercrossover distances in physical units were converted into genetic distances (centimorgans) in wild type, *pch2*Δ, *mlh3*Δ, *mlh3*Δ *pch2*Δ, to account for differences in crossover frequencies (*Materials and Methods*) (Figure 6A). To account for differences in the number of tetrads analyzed, we also converted the counts for intercrossover distances as a fraction of the total for wild type and each mutant (Figure 6B). For the wild-type strain, the *γ* value was 1.83. Interference was reduced in *pch2*Δ (*γ* = 1.33), *mlh3*Δ (*γ* = 1.29) and *mlh3*Δ *pch2*Δ (*γ* = 1.13) compared to wild type. Interference analysis using the two pathway model that incorporates contribution from both interfering and noninterfering pathways also showed reduced interference in *mlh3*Δ, *pch2*Δ, and *mlh3*Δ *pch2*Δ (File S3, and see Copenhaver *et al.* 2002; Housworth and Stahl 2003). We also analyzed interference from the genome wide crossover data using the CoC method (*Materials and Methods*, and see Anderson *et al.* 2015). Statistical significance of the difference between observed and expected frequency of double crossovers was estimated using the chi-square test. Interference (1 – CoC) was significantly reduced in *mlh3*Δ (*P* = 0.00033), *pch2*Δ (*P* = 4.36 × 10^−6^), and *mlh3*Δ *pch2*Δ (*P* = 1.85 × 10^−9^) relative to the wild type (Figure 6C).

The reduced interference in *mlh3*Δ is consistent with the two pathway model for crossover formation (Copenhaver *et al.* 2002; Stahl *et al.* 2004; Getz *et al.* 2008). *pch2*Δ has been shown previously to lack genetic interference (Joshi *et al.* 2009; Zanders and Alani 2009). However, analysis of Zip3 foci that mark DSB sites that will be repaired as crossovers, suggests that interference is observed in *pch2*Δ at the crossover designation stage (Zhang *et al.* 2014). The loss of genetic interference in *pch2*Δ was thought to be due to the misregulation of the crossover/noncrossover decision step, and not because of the excess crossovers (Zanders and Alani 2009). We observe an increase in both crossovers and noncrossovers in *pch2*Δ mutants and Zip3 localization is known to be maintained in *pch2*Δ (Table 1, Zhang *et al.* 2014). Therefore, the reduced interference in *pch2*Δ is most likely due to the excess crossovers made by the MutLγ-dependent interfering, and the Mus81-Mms4-dependent noninterfering crossover pathways.

Since *mlh3*Δ *pch2*Δ also makes more noncrossovers, it is unlikely that the loss of interference is due to impairment of the crossover/noncrossover decision. The loss of interference is most likely because nearly all crossovers in *mlh3*Δ *pch2*Δ are made through the Mus81-Mms4 pathway (Figure 5B). Loss of interference in the *mlh3*Δ *pch2*Δ mutant is also supported from the uniform crossover density observed across all chromosome sizes (Figure 2D). The loss of genetic interference in *mlh3*Δ *pch2*Δ is particularly interesting as it makes as many crossovers on average as wild type. In summary, genetic interference is reduced across all three categories of crossover frequencies (reduced, equivalent, and greater than wild-type crossovers for *mlh3*Δ, *mlh3*Δ *pch2*Δ, and *pch2*Δ, respectively). No chromatid interference was observed among wild type, *mlh3*Δ, and *pch2*Δ *mutants* (*P* > 0.05, chi-square test), but negative chromatid interference characterized by a slight excess of two strand crossovers may be present in *mlh3*Δ *pch2*Δ mutants (0.01 < *P* < 0.05, chi-square test) (Table S8).

### Noninterfering crossovers are less efficient in promoting crossover assurance

We tested the effect of a wide range of crossover frequencies on obligate crossovers using data from genome-wide analysis of meiotic recombination in the *mlh3*Δ, *pch2*Δ, and *mlh3*Δ *pch2*Δ mutants. We determined the frequency of meiosis with nonexchange events in *mlh3*Δ, *pch2*Δ, and *mlh3*Δ *pch2*Δ mutants. The percentage of meiosis with 1 or >1 nonexchange chromosome was 3% for wild type, 7% for *pch2*Δ, 47% for *mlh3*Δ, and 20% for *mlh3*Δ *pch2*Δ (Table S3). *mlh3*Δ, which showed a decrease in both genetic interference and crossover frequencies, had the highest number of meioses with nonexchange events. In 5% of the meioses, the *mlh3*Δ mutant also had >1 nonexchange chromosome. However, *mlh3*Δ had an overall lower amount of nonexchange events among four-spore viable tetrads (47%) compared to *msh4*Δ (72%) but similar to *msh4-R676W* (42%, Krishnaprasad *et al.* 2015). These results suggest that, even though *mlh3*Δ makes 64 crossovers, the obligate crossover is not maintained. These findings reinforce our earlier observations that simultaneous reductions in crossover frequencies and genetic interference compromise the obligate crossover (Krishnaprasad *et al.* 2015). In *pch2*Δ, the number of meioses with nonexchange events (7%) was comparable to wild type (3%) (*P* = 0.37, Binomial test). This is likely due to the excess crossovers made in *pch2*Δ that compensate for the loss of genetic interference. Previously, we had estimated that, if the crossovers are distributed randomly, a wild-type yeast cell would require up to ∼200 crossovers to ensure (with probability >0.98) the absence of nonexchange chromosomes (Krishnaprasad *et al.* 2015). The observations with the *pch2*Δ mutant support the theoretical predictions that genetic interference reduces the number of crossovers required for crossover assurance. The results also suggest that *pch2*Δ does not significantly increase nonexchange chromosome frequency through other mechanisms. The *mlh3*Δ *pch2*Δ mutant was most interesting, since, although it made as many crossovers as wild type, the percentage of meiosis with nonexchange events (20%) was significantly more than wild type (3%) (*P* = 2.7 × 10^−3^, Binomial test). These data experimentally demonstrate that if the wild-type frequency of crossovers were randomly distributed in *S. cerevisiae*, there would be more nonexchange chromosomes.

**Figure 7:**
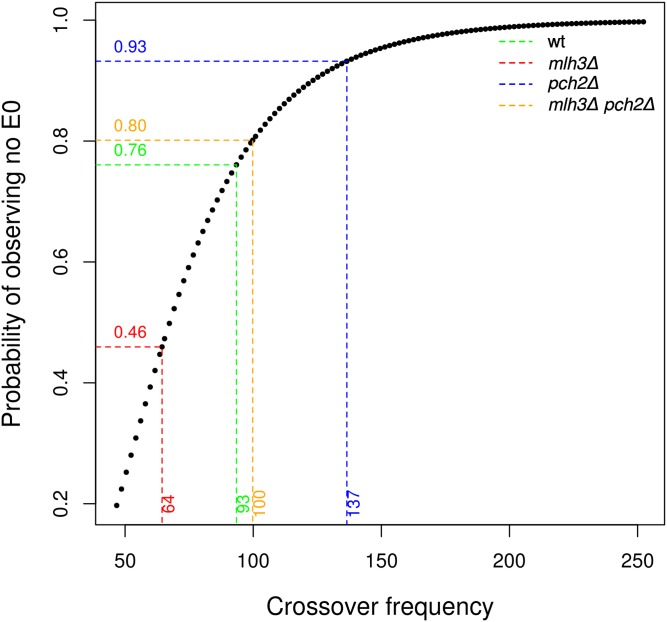
Probability of observing no E0 (chromosomes with zero crossovers per meiosis) for wild type, *mlh3*Δ, *pch2*Δ, and *mlh3*Δ *pch2*Δ. The probability of observing no E0 events is plotted against the crossover frequency modeled as a Poisson distribution. The dotted lines indicate the probabilities for finding no E0s in the absence of genetic interference for the experimentally observed average crossover counts per cell (*mlh3*Δ: 64; wild type: 93; *mlh3*Δ *pch2*Δ: 100; *pch2*Δ: 137).

To further test how crossover frequency affects obligate crossovers, we estimated the percentage of meiosis with no nonexchange chromosomes expected for *mlh3*Δ, *pch2*Δ, and *mlh3*Δ *pch2*Δ by modeling the crossovers as a Poisson distribution (the Poisson distribution assumes the crossover events are independent) (Figure 7). The expected percentage of meiosis with no nonexchange chromosomes, 46% (*mlh3*Δ), 80% (*mlh3*Δ *pch2*Δ), 93% (*pch2*Δ) matched well with the experimental observations: 53% (*mlh3*Δ), 80% (*mlh3*Δ *pch2*Δ) and 93% (*pch2*Δ). Crossover distributions in *mlh3*Δ, *pch2*Δ, and *mlh3*Δ *pch2*Δ, therefore, approximate a Poisson process. For wild type, the percentage of meiosis with nonexchange chromosomes (3%) is significantly lower than expected (24%) due to the presence of interference. These observations are consistent with the requirement for higher crossover frequencies in the absence of genetic interference to ensure obligate crossovers (Krishnaprasad *et al.* 2015; Berchowitz and Copenhaver 2010; Kaback *et al.* 1999).

In mutants with reduced genetic interference, adjacent crossovers may be closely spaced. As a result, they could be annotated as double noncrossovers inflating the number of nonexchange chromosomes. We inspected each nonexchange chromosome in the wild type, *mlh3*Δ, *pch2*Δ, and *mlh3*Δ *pch2*Δ mutants for ambiguity in the annotation for double crossovers and double noncrossovers, and did not find any significant difference in the estimate of nonexchange chromosomes (Table S9). All nonexchange chromosomes, except one, had gene conversion events (Table S9). This observation suggests that lack of an obligate crossover was not a consequence of the absence of recombination interactions between the homologs, but rather a failure to convert even one of them into a crossover.

### Small chromosomes are most sensitive to loss of the obligate crossover

**Figure 8:**
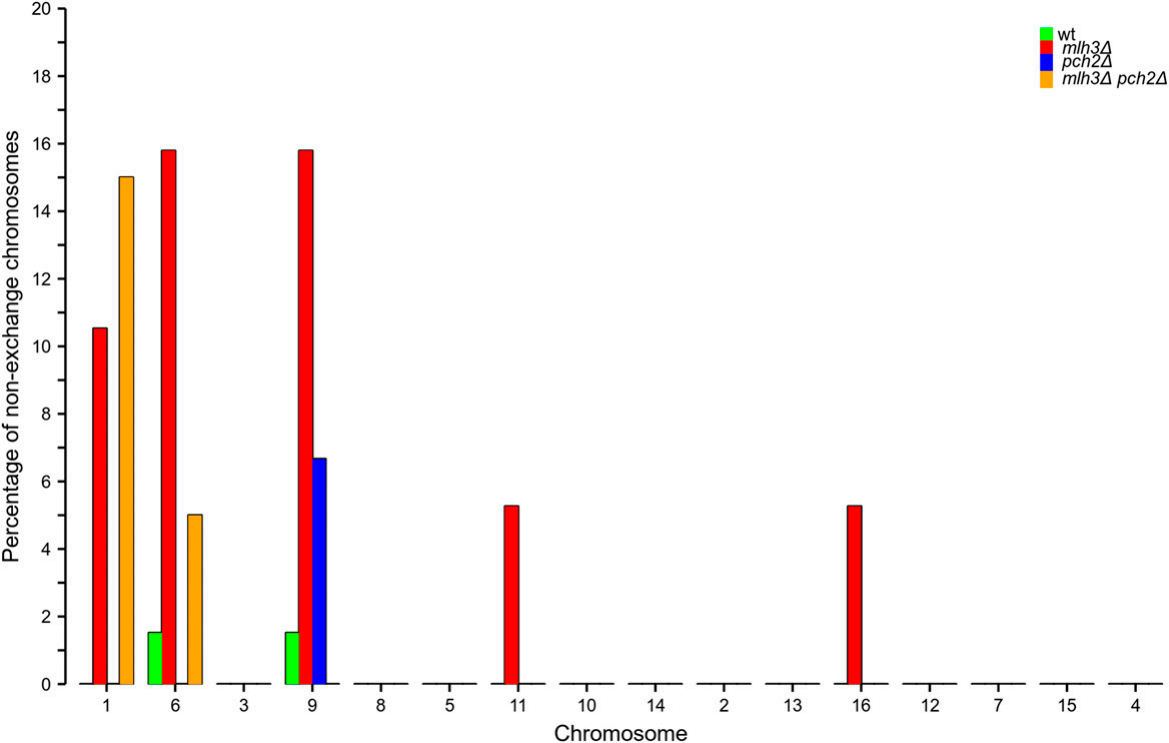
The percentage of meiosis that are nonexchange for each chromosome for wild type, *mlh3*Δ, *pch2*Δ, and *mlh3*Δ *pch2*Δ. Chromosomes are shown in increasing order of size.

We analyzed the influence of chromosome size on the occurrence of nonexchange chromosomes in the wild type, *mlh3*Δ, *pch2*Δ, and *mlh3*Δ *pch2*Δ mutants (Figure 8). For *mlh3*Δ, nonexchange chromosomes included small (I, VI, and IX), medium (XI), and large chromosomes (XVI). This observation is similar to the distribution of nonexchange chromosomes in *msh4*Δ observed previously, except that the number of nonexchange chromosomes is much less in *mlh3*Δ (Krishnaprasad *et al.* 2015). For *pch2*Δ, the only nonexchange chromosome was a small chromosome (IX). For *mlh3*Δ *pch2*Δ, only small chromosomes (I and VI) were nonexchange. These observations suggest that small chromosomes are particularly susceptible to the loss of the obligate crossover when there are crossover and/or interference defects. A stronger reduction in crossover frequencies, as observed in *mlh3*Δ mutants, is required for the loss of obligate crossovers on medium and large chromosomes. These observations further consolidate our previous results showing that small chromosomes are particularly sensitive to variations in crossover frequency (Krishnaprasad *et al.* 2015). A high frequency of nonexchange has been observed for smaller chromosomes in other crossover defective mutants, and also in human meiosis (Chen *et al.* 2008; Fledel-Alon *et al.* 2009). Among the small chromosomes, we observed a lack of E0s for the chromosome III. A similar phenomenon was seen in Krishnaprasad *et al.* (2015), where chromosome III had the fewest E0s among the small chromosomes. Analysis of copy number variation using read depth information did not detect aneuploidy in any of the *mlh3*Δ, *pch2*Δ, and *mlh3*Δ *pch2*Δ sequenced tetrads (Figure S9).

## Discussion

We previously analyzed genome-wide crossovers in a *msh4* hypomorph to demonstrate that a random distribution of reduced number of crossover events can increase nonexchange chromosome frequencies (Krishnaprasad *et al.* 2015). Our current work generalizes this observation to a larger context by analyzing nonexchange events when there is a random distribution of reduced, wild-type, and greater than wild-type frequency of crossovers using the *mlh3*Δ, *pch2*Δ, and *mlh3*Δ *pch2*Δ mutants.

### Obligate crossover is sensitive to crossover frequencies and genetic interference

Genetic interference is affected in *mlh3*Δ, *pch2*Δ, and *mlh3*Δ *pch2*Δ due to increased contributions from the noninterfering pathway. Interference is also compromised in *mlh3*Δ and *mlh3*Δ *pch2*Δ because of defects in converting crossover designated sites into actual crossovers. The *pch2*Δ results suggest that mutants with reduced genetic interference can maintain an obligate crossover if crossover frequencies are significantly higher than in wild type (Figure 7). If the crossover frequency is reduced compared to wild type, there is a loss of the obligate crossover, as in the case of *mlh3*Δ. In *mlh3*Δ *pch2*Δ, we observed crossover frequencies equivalent to wild type (100 crossovers per meiosis), but an increased proportion of nonexchange chromosomes. Three lines of evidence support enhanced nonexchange chromosome frequency in *mlh3*Δ *pch2*Δ. First, we observe nonexchange chromosomes in 20% of meiosis in *mlh3*Δ *pch2*Δ in the S288c/YJM789 hybrid (Figure 8 and Table S3). Second, theoretical predictions by modeling the *mlh3*Δ *pch2*Δ crossover distribution as a Poisson process also support higher nonexchange chromosome frequencies consistent with the experimental results (Figure 7). Third, *mlh3*Δ *pch2*Δ showed spore viability pattern typical of Meiosis I nondisjunction in the SK1 EAY1108/1112 background (Figure 4). Since most crossovers in *mlh3*Δ *pch2*Δ are made by the Mus81-Mms4 pathway (Figure 5B), these observations are consistent with noninterfering crossovers being less efficient in supporting an obligate crossover. The higher E0 frequency in *mlh3∆ pch2∆* also supports the prediction that *S. cerevisiae* will require up to 200 crossovers in the absence of genetic interference to have >0.98% probability of an obligate crossover on every homolog pair (Krishnaprasad *et al.* 2015).

Triple mutant analysis with another resolvase (Slx4) showed that genetic map distances measured from spore data were not significantly different between *mlh3*Δ *pch2*Δ (79.9 cM) and *mlh3*Δ *pch2*Δ *slx4*Δ (77.8 cM). However, map distances from tetrad data showed significant differences between *mlh3*Δ *pch2*Δ (113.6 cM) and *mlh3*Δ *pch2*Δ *slx4*Δ (91.2 cM). These results suggest Slx4 only makes a minor contribution to crossovers in the *mlh3*Δ *pch2*Δ mutant. Slx4 may be involved in resolving a fraction of closely spaced double crossovers that contribute to the NPD class of tetrads.

### Segregation of nonexchange chromosomes in mlh3**Δ**

Since *mlh3*Δ and wild type have identical spore viability in the S288c/YJM789 hybrid and similar proportion of the four-viable spore tetrad class, we tested the association of spore viability with the nonexchange chromosome frequency. Mlh3 acts downstream of Msh4/5 at the final steps of Holliday Junction resolution (Baker *et al.* 1996; Lipkin *et al.* 2002; Snowden *et al.* 2004; Kolas *et al.* 2005). *mlh3*Δ mutants, therefore, provide an advantage in relating variations in crossover frequency with the loss of obligate crossovers without the confounding effects that may arise in other mutants that have an early role in the crossover/noncrossover decision step or that affect DSB formation. It is also important to recognize that the data for the nonexchange chromosome frequency comes from the analysis of the four viable spore tetrads only. *mlh3*Δ has high spore viability (85%), strong crossover defects (64 crossovers per meiosis), and abundant single nonexchange chromosomes (Figure 8 and Table 1). These data are similar to the pattern seen with *msh4-R676W* earlier (Krishnaprasad *et al.* 2015). The *mlh3*Δ mutant reinforces the idea that a reduction in crossover frequency results in loss of the obligate crossover, even though the average crossover number is still four times the number of homologs. The high spore viability of *mlh3*Δ, suggests these single nonexchange chromosomes are efficiently segregated as observed with *msh4-R676W* (Krishnaprasad *et al.* 2015), supporting the hypothesis that crossovers facilitate but are not essential for chromosome segregation. Other mechanisms, such as distributive segregation, spindle checkpoint, and centromere pairing may also contribute to the segregation of achiasmate chromosomes in *S. cerevisiae* (Guacci and Kaback 1991; Shonn *et al.* 2000; Kemp *et al.* 2004). These observations also suggest spore viability is a poor measure of the status of obligate crossovers. The *mlh3*Δ mutant showed 64 crossovers genome-wide compared to 49 reported in *msh4*Δ (Krishnaprasad *et al.* 2015). These results provide support to previous observation on Msh4/5 dependent but Mlh1/3 independent crossover pathways (Argueso *et al.* 2004). It is also expected if, in *mlh3*Δ, some of the crossover precursor joint molecules are resolved by structure selective nucleases as both crossovers and noncrossovers.

### Recombination maps in pch2**Δ** capture DSB variation

We observed a simultaneous increase in crossovers and noncrossovers in *pch2*Δ, which suggested that DSBs are increased in *pch2*Δ. Previous studies have shown a complex role for Pch2 in DSB formation, with increased DSB formation at certain loci, such as the rDNA locus (Vader *et al.* 2011), and a general reduction in DSBs at other loci (Farmer *et al.* 2012). Pch2 also has a role in the processing of early DSBs (Joshi *et al.* 2015). These studies suggest overall DSB levels may remain constant in *pch2*Δ, and the effects need to be examined on a per locus/chromosome basis (Farmer *et al.* 2012). High-resolution crossover and noncrossover data in *pch2*Δ provide an indirect readout of variation in DSB formation. For example, *pch2*Δ mutants do not show negative correlation of chromosome size with crossover plus noncrossover density. We observe more crossovers around the rDNA locus in *pch2*Δ mutants. *pch2*Δ, as well as *mlh3*Δ *pch2*Δ, also show suppression of both crossovers and noncrossovers near telomeres, which may suggest an altered DSB distribution near telomeric regions. Also, we observed increased crossovers as well as noncrossovers on Chr III, consistent with increased DSB formation on chr III (Farmer *et al.* 2012). Such differences in DSB distribution, possibly by regulating Hop1 function (Borner *et al.* 2008; Wojtasz *et al.* 2009), and the use of the Mus81-Mms4 pathway, may be responsible for the differences in crossover and noncrossover patterns in *pch2*Δ and *mlh3*Δ *pch2*Δ compared to wild type (Figure S4, Figure S6, and Figure S7).

Although Rad51 foci analyses do not give information on regional DSB variation, it can provide information on total cellular DSB levels and its turnover. We did not observe peak Rad51 focus counts in *pch2*Δ to be greater than wild type. This is consistent with the physical analysis of DSBs, which suggests that, at least at specific loci, there is no increase in DSB formation in *pch2*Δ mutants (Zanders *et al.* 2011; Farmer *et al.* 2012; Joshi *et al.* 2015). Instead, *pch2*Δ showed the persistence of peak DSB foci over an extended period of the meiotic time course from 3 to 8 hr. It is possible there is DSB turnover in *pch2*Δ, so that DSBs are made and repaired and the process is repeated, leading to an overall increase in crossover and noncrossovers. Such a possibility is supported by previous observations of the accumulation of Rad51 foci in *pch2*Δ mutants (Subramanian *et al.* 2016). The only other alternative is that there is more repair of DSBs by the interhomolog pathway in *pch2*Δ mutants, which may account for increased crossovers and noncrossovers. But all available evidence suggests that in *pch2*Δ there is more intersister recombination than in wild type (Zanders *et al.* 2011; Joshi *et al.* 2015). Defects in DSB processing (*e.g.*, longer resection) may also result in longer ssDNA filaments stabilized by Rad51 that may account for the long gene conversion tracts observed in *pch2*Δ mutants (Figure S8, B and C). The role of Pch2 in DSB regulation requires further investigation.

In conclusion, the *mlh3*Δ *pch2*Δ data provides experimental support from genome-wide analysis that wild-type crossover frequencies distributed randomly cannot maintain an obligate crossover on all homolog pairs (Kaback *et al.* 1999; Berchowitz and Copenhaver 2010; Krishnaprasad *et al.* 2015). The distribution of crossovers and noncrossovers suggest that chromosome-size dependent DSB formation is affected in *pch2∆*. *pch2*Δ mutants also show that obligate crossovers can be ensured through a random distribution of excess crossovers. These results are consistent with MutL gamma dependent interfering crossovers being more efficient in promoting homolog disjunction compared to the Mus81-Mms4 dependent noninterfering crossovers.

## Supplementary Material

Supplemental material is available online at www.g3journal.org/lookup/suppl/doi:10.1534/g3.117.040071/-/DC1.

## References

[bib1] Allers T. Lichten M., 2001 Differential timing and control of noncrossover and crossover recombination during meiosis. Cell 106: 47–57. 11461701 10.1016/s0092-8674(01)00416-0

[bib2] Anderson C. M. Chen S. Y. Dimon M. T. Oke A. DeRisi J. L., 2011 ReCombine: a suite of programs for detection and analysis of meiotic recombination in whole-genome datasets. PLoS One 6: e25509. 22046241 10.1371/journal.pone.0025509 PMC3201961

[bib3] Anderson C. M. Oke A. Yam P. Zhuge T. Fung J. C., 2015 Reduced crossover interference and increased ZMM-independent recombination in the absence of Tel1/ATM. PLoS Genet. 11: e1005478. 26305689 10.1371/journal.pgen.1005478 PMC4549261

[bib4] Argueso J. L. Kijas A. W. Sarin S. Heck J. Waase M., 2003 Systematic mutagenesis of the *Saccharomyces cerevisiae* MLH1 gene reveals distinct roles for Mlh1p in meiotic crossing over and in vegetative and meiotic mismatch repair. Mol. Cell. Biol. 23: 873–886. 12529393 10.1128/MCB.23.3.873-886.2003 PMC140715

[bib5] Argueso J. L. Wanat J. Gemici Z. Alani E., 2004 Competing crossover pathways act during meiosis in *Saccharomyces cerevisiae*. Genetics 168: 1805–1816. 15611158 10.1534/genetics.104.032912 PMC1448724

[bib6] Baker S. M. Plug A. W. Prolla T. A. Bronner C. E. Harris A. C., 1996 Involvement of mouse Mlh1 in DNA mismatch repair and meiotic crossing over. Nat. Genet. 13: 336–342. 8673133 10.1038/ng0796-336

[bib7] Barchi M. Roig I. Di Giacomo M. de Rooij D. G. Keeney S., 2008 ATM promotes the obligate XY crossover and both crossover control and chromosome axis integrity on autosomes. PLoS Genet. 4: e1000076. 18497861 10.1371/journal.pgen.1000076 PMC2374915

[bib8] Benaglia T. Chauveau D. Hunter D. Young D., 2009 mixtools: an R package for analyzing finite mixture models. J. Stat. Softw. 32: 1–29.

[bib9] Berchowitz L. E. Copenhaver G. P., 2010 Genetic interference: don’t stand so close to me. Curr. Genomics 11: 91–102. 20885817 10.2174/138920210790886835 PMC2874225

[bib10] Bishop D. K., 1994 RecA homologs Dmc1 and Rad51 interact to form multiple nuclear complexes prior to meiotic chromosome synapsis. Cell 79: 1081–1092. 7528104 10.1016/0092-8674(94)90038-8

[bib11] Bolger A. M. Lohse M. Usadel B., 2014 Trimmomatic: a flexible trimmer for Illumina sequence data. Bioinformatics 30: 2114–2120. 24695404 10.1093/bioinformatics/btu170 PMC4103590

[bib12] Borner G. V. Kleckner N. Hunter N., 2004 Crossover/noncrossover differentiation, synaptonemal complex formation, and regulatory surveillance at the leptotene/zygotene transition of meiosis. Cell 117: 29–45. 15066280 10.1016/s0092-8674(04)00292-2

[bib13] Borner G. V. Barot A. Kleckner N., 2008 Yeast Pch2 promotes domainal axis organization, timely recombination progression, and arrest of defective recombinosomes during meiosis. Proc. Natl. Acad. Sci. USA 105: 3327–3332. 18305165 10.1073/pnas.0711864105 PMC2265181

[bib14] Chen C. Jomaa A. Ortega J. Alani E. E., 2014 Pch2 is a hexameric ring ATPase that remodels the chromosome axis protein Hop1. Proc. Natl. Acad. Sci. USA 111: E44–E53. 24367111 10.1073/pnas.1310755111 PMC3890899

[bib15] Chen S. Y. Tsubouchi T. Rockmill B. Sandler J. S. Richards D. R., 2008 Global analysis of the meiotic crossover landscape. Dev. Cell 15: 401–415. 18691940 10.1016/j.devcel.2008.07.006 PMC2628562

[bib16] Cole F. Kauppi L. Lange J. Roig I. Wang R., 2012 Homeostatic control of recombination is implemented progressively in mouse meiosis. Nat. Cell Biol. 14: 424–430. 22388890 10.1038/ncb2451 PMC3319518

[bib17] Copenhaver G. P. Housworth E. A. Stahl F. W., 2002 Crossover interference in *Arabidopsis*. Genetics 160: 1631–1639. 11973316 10.1093/genetics/160.4.1631 PMC1462055

[bib18] Cotton V. E. Hoffmann E. R. Borts R. H., 2010 Distinct regulation of Mlh1p heterodimers in meiosis and mitosis in *Saccharomyces cerevisiae*. Genetics 185: 459–467. 20382827 10.1534/genetics.110.116806 PMC2881129

[bib19] Davis L. Smith G. R., 2003 Nonrandom homolog segregation at meiosis I in *Schizosaccharomyces pombe* mutants lacking recombination. Genetics 163: 857–874. 12663528 10.1093/genetics/163.3.857 PMC1462471

[bib20] de los Santos T. Hunter N. Lee C. Larkin B. Loidl J., 2003 The Mus81/Mms4 endonuclease acts independently of double-Holliday junction resolution to promote a distinct subset of crossovers during meiosis in budding yeast. Genetics 164: 81–94. 12750322 10.1093/genetics/164.1.81 PMC1462551

[bib21] De Muyt A. Jessop L. Kolar E. Sourirajan A. Chen J., 2012 BLM helicase ortholog Sgs1 is a central regulator of meiotic recombination intermediate metabolism. Mol. Cell 46: 43–53. 22500736 10.1016/j.molcel.2012.02.020 PMC3328772

[bib22] DePristo M. A. Banks E. Poplin R. Garimella K. V. Maguire J. R., 2011 A framework for variation discovery and genotyping using next-generation DNA sequencing data. Nat. Genet. 43: 491–498. 21478889 10.1038/ng.806 PMC3083463

[bib23] Farmer S. Hong E. J. Leung W. K. Argunhan B. Terentyev Y., 2012 Budding yeast Pch2, a widely conserved meiotic protein, is involved in the initiation of meiotic recombination. PLoS One 7: e39724. 22745819 10.1371/journal.pone.0039724 PMC3382142

[bib24] Fledel-Alon A. Wilson D. J. Broman K. Wen X. Ober C., 2009 Broad-scale recombination patterns underlying proper disjunction in humans. PLoS Genet. 5: e1000658. 19763175 10.1371/journal.pgen.1000658 PMC2734982

[bib25] Getz T. J. Banse S. A. Young L. S. Banse A. V. Swanson J., 2008 Reduced mismatch repair of heteroduplexes reveals “non”-interfering crossing over in wild-type *Saccharomyces cerevisiae*. Genetics 178: 1251–1269. 18385111 10.1534/genetics.106.067603 PMC2278109

[bib26] Gietz R. D. Schiestl R. H. Willems A. R. Woods R. A., 1995 Studies on the transformation of intact yeast cells by the LiAc/ss‐DNA/PEG procedure. Yeast 11: 355–360. 7785336 10.1002/yea.320110408

[bib27] Goldstein A. L. McCusker J. H., 1999 Three new dominant drug resistance cassettes for gene disruption in *Saccharomyces cerevisiae*. Yeast 15: 1541–1553. 10514571 10.1002/(SICI)1097-0061(199910)15:14<1541::AID-YEA476>3.0.CO;2-K

[bib28] Guacci V. Kaback D. B., 1991 Distributive disjunction of authentic chromosomes in *Saccharomyces cerevisiae*. Genetics 127: 475–488. 2016050 10.1093/genetics/127.3.475 PMC1204375

[bib29] Hawley R. S. Irick H. Zitron A. E. Haddox D. A. Lohe A., 1992 There are two mechanisms of achiasmate segregation in *Drosophila* females, one of which requires heterochromatic homology. Dev. Genet. 13: 440–467. 1304424 10.1002/dvg.1020130608

[bib30] Higashide M. Shinohara M., 2016 Budding yeast SLX4 contributes to the appropriate distribution of crossovers and meiotic double-strand break formation on bivalents during meiosis. G3 Bethesda 6: 2033–2042. 27172214 10.1534/g3.116.029488 PMC4938656

[bib31] Hillers K. J., 2004 Crossover interference. Curr. Biol. 14: R1036–R1037. 15620632 10.1016/j.cub.2004.11.038

[bib32] Hillers K. J. Villeneuve A. M., 2003 Chromosome-wide control of meiotic crossing over in *C. elegans*. Curr. Biol. 13: 1641–1647. 13678597 10.1016/j.cub.2003.08.026

[bib33] Ho H. C. Burgess S. M., 2011 Pch2 acts through Xrs2 and Tel1/ATM to modulate interhomolog bias and checkpoint function during meiosis. PLoS Genet. 7: e1002351. 22072981 10.1371/journal.pgen.1002351 PMC3207854

[bib34] Hochwagen A. Tham W. H. Brar G. A. Amon A., 2005 The FK506 binding protein Fpr3 counteracts protein phosphatase 1 to maintain meiotic recombination checkpoint activity. Cell 122: 861–873. 16179256 10.1016/j.cell.2005.07.010

[bib35] Housworth E. A. Stahl F. W., 2003 Crossover interference in humans. Am. J. Hum. Genet. 73: 188–197. 12772089 10.1086/376610 PMC1180580

[bib36] Hunter N., 2011 Double duty for Exo1 during meiotic recombination. Cell Cycle 10: 2607–2609. 21785266 10.4161/cc.10.16.16452

[bib37] Hunter N. Kleckner N., 2001 The single-end invasion: an asymmetric intermediate at the double-strand break to double-Holliday junction transition of meiotic recombination. Cell 106: 59–70. 11461702 10.1016/s0092-8674(01)00430-5

[bib38] Hyppa R. W. Smith G. R., 2010 Crossover invariance determined by partner choice for meiotic DNA break repair. Cell 142: 243–255. 20655467 10.1016/j.cell.2010.05.041 PMC2911445

[bib39] Joshi N. Barot A. Jamison C. Borner G. V., 2009 Pch2 links chromosome axis remodeling at future crossover sites and crossover distribution during yeast meiosis. PLoS Genet. 5: e1000557. 19629172 10.1371/journal.pgen.1000557 PMC2708914

[bib40] Joshi N. Brown M. S. Bishop D. K. Borner G. V., 2015 Gradual implementation of the meiotic recombination program via checkpoint pathways controlled by global DSB levels. Mol. Cell 57: 797–811. 25661491 10.1016/j.molcel.2014.12.027 PMC4392720

[bib41] Kaback D. B. Barber D. Mahon J. Lamb J. You J., 1999 Chromosome size-dependent control of meiotic reciprocal recombination in *Saccharomyces cerevisiae*: the role of crossover interference. Genetics 152: 1475–1486. 10430577 10.1093/genetics/152.4.1475 PMC1460698

[bib42] Kaur H. De Muyt A. Lichten M., 2015 Top3-Rmi1 DNA single-strand decatenase is integral to the formation and resolution of meiotic recombination intermediates. Mol. Cell 57: 583–594. 25699707 10.1016/j.molcel.2015.01.020 PMC4338413

[bib43] Kemp B. Boumil R. M. Stewart M. N. Dawson D. S., 2004 A role for centromere pairing in meiotic chromosome segregation. Genes Dev. 18: 1946–1951. 15289462 10.1101/gad.1227304 PMC514173

[bib44] Kleckner N. Zickler D. Jones G. H. Dekker J. Padmore R., 2004 A mechanical basis for chromosome function. Proc. Natl. Acad. Sci. USA 101: 12592–12597. 15299144 10.1073/pnas.0402724101 PMC515102

[bib45] Kolas N. K. Svetlanov A. Lenzi M. L. Macaluso F. P. Lipkin S. M., 2005 Localization of MMR proteins on meiotic chromosomes in mice indicates distinct functions during prophase I. J. Cell Biol. 171: 447–458. 16260499 10.1083/jcb.200506170 PMC2171243

[bib46] Krishnaprasad G. N. Anand M. T. Lin G. Tekkedil M. M. Steinmetz L. M., 2015 Variation in crossover frequencies perturb crossover assurance without affecting meiotic chromosome segregation in *Saccharomyces cerevisiae*. Genetics 199: 399–412. 25467183 10.1534/genetics.114.172320 PMC4317650

[bib47] Langmead B. Salzberg S. L., 2012 Fast gapped-read alignment with Bowtie 2. Nat. Methods 9: 357–359. 22388286 10.1038/nmeth.1923 PMC3322381

[bib48] Libuda D. E. Uzawa S. Meyer B. J. Villeneuve A. M., 2013 Meiotic chromosome structures constrain and respond to designation of crossover sites. Nature 502: 703–706. 24107990 10.1038/nature12577 PMC3920622

[bib49] Lipkin S. M. Moens P. B. Wang V. Lenzi M. Shanmugarajah D., 2002 Meiotic arrest and aneuploidy in MLH3-deficient mice. Nat. Genet. 31: 385–390. 12091911 10.1038/ng931

[bib50] Mancera E. Bourgon R. Brozzi A. Huber W. Steinmetz L. M., 2008 High-resolution mapping of meiotic crossovers and non-crossovers in yeast. Nature 454: 479–485. 18615017 10.1038/nature07135 PMC2780006

[bib51] Martini E. Diaz R. L. Hunter N. Keeney S., 2006 Crossover homeostasis in yeast meiosis. Cell 126: 285–295. 16873061 10.1016/j.cell.2006.05.044 PMC1949389

[bib52] Matos J. Blanco M. G. Maslen S. Skehel J. M. West S. C., 2011 Regulatory control of the resolution of DNA recombination intermediates during meiosis and mitosis. Cell 147: 158–172. 21962513 10.1016/j.cell.2011.08.032 PMC3560330

[bib53] McKenna A. Hanna M. Banks E. Sivachenko A. Cibulskis K., 2010 The genome analysis toolkit: a MapReduce framework for analyzing next-generation DNA sequencing data. Genome Res. 20: 1297–1303. 20644199 10.1101/gr.107524.110 PMC2928508

[bib54] McPeek M. S. Speed T. P., 1995 Modeling interference in genetic recombination. Genetics 139: 1031–1044. 7713406 10.1093/genetics/139.2.1031 PMC1206354

[bib55] Muller H. J., 1916 The mechanism of crossing-over. Am. Nat. 50: 284–305.

[bib56] Murakami H. Borde V. Nicolas A. Keeney S., 2009 Gel electrophoresis assays for analyzing DNA double-strand breaks in *Saccharomyces cerevisiae* at various spatial resolutions. Methods Mol. Biol. 557: 117–142. 19799180 10.1007/978-1-59745-527-5_9 PMC3157973

[bib57] Nakagawa T. Ogawa H., 1999 The *Saccharomyces cerevisiae* MER3 gene, encoding a novel helicase-like protein, is required for crossover control in meiosis. EMBO J. 18: 5714–5723. 10523314 10.1093/emboj/18.20.5714 PMC1171638

[bib58] Neale M. J. Keeney S., 2006 Clarifying the mechanics of DNA strand exchange in meiotic recombination. Nature 442: 153–158. 16838012 10.1038/nature04885 PMC5607947

[bib59] Newnham L. Jordan P. Rockmill B. Roeder G. S. Hoffmann E., 2010 The synaptonemal complex protein, Zip1, promotes the segregation of nonexchange chromosomes at meiosis I. Proc. Natl. Acad. Sci. USA 107: 781–785. 20080752 10.1073/pnas.0913435107 PMC2818913

[bib60] Nishant K. T. Plys A. J. Alani E., 2008 A mutation in the putative MLH3 endonuclease domain confers a defect in both mismatch repair and meiosis in *Saccharomyces cerevisiae*. Genetics 179: 747–755. 18505871 10.1534/genetics.108.086645 PMC2429871

[bib61] Novak J. E. Ross-Macdonald P. B. Roeder G. S., 2001 The budding yeast Msh4 protein functions in chromosome synapsis and the regulation of crossover distribution. Genetics 158: 1013–1025. 11454751 10.1093/genetics/158.3.1013 PMC1461720

[bib62] Oh S. D. Lao J. P. Taylor A. F. Smith G. R. Hunter N., 2008 RecQ helicase, Sgs1, and XPF family endonuclease, Mus81-Mms4, resolve aberrant joint molecules during meiotic recombination. Mol. Cell 31: 324–336. 18691965 10.1016/j.molcel.2008.07.006 PMC2587322

[bib63] Oke A. Anderson C. M. Yam P. Fung J. C., 2014 Controlling meiotic recombinational repair - specifying the roles of ZMMs, Sgs1 and Mus81/Mms4 in crossover formation. PLoS Genet. 10: e1004690. 25329811 10.1371/journal.pgen.1004690 PMC4199502

[bib64] Pan J. Sasaki M. Kniewel R. Murakami H. Blitzblau H. G., 2011 A hierarchical combination of factors shapes the genome-wide topography of yeast meiotic recombination initiation. Cell 144: 719–731. 21376234 10.1016/j.cell.2011.02.009 PMC3063416

[bib65] Petronczki M. Siomos M. F. Nasmyth K., 2003 Un menage a quatre: the molecular biology of chromosome segregation in meiosis. Cell 112: 423–440. 12600308 10.1016/s0092-8674(03)00083-7

[bib66] Roeder G. S., 1997 Meiotic chromosomes: it takes two to tango. Genes Dev. 11: 2600–2621. 9334324 10.1101/gad.11.20.2600

[bib67] Rogacheva M. V. Manhart C. M. Chen C. Guarne A. Surtees J., 2014 Mlh1-Mlh3, a meiotic crossover and DNA mismatch repair factor, is a Msh2-Msh3-stimulated endonuclease. J. Biol. Chem. 289: 5664–5673. 24403070 10.1074/jbc.M113.534644 PMC3937641

[bib68] Rose M. D. Winston F. M. Heiter P., 1990 *Methods in Yeast Genetics: A Laboratory Course Manual* Cold Spring Harbor Laboratory Press, Cold Spring Harbor, New York.

[bib69] San-Segundo P. A. Roeder G. S., 1999 Pch2 links chromatin silencing to meiotic checkpoint control. Cell 97: 313–324. 10319812 10.1016/s0092-8674(00)80741-2

[bib70] Serrentino M. E. Borde V., 2012 The spatial regulation of meiotic recombination hotspots: are all DSB hotspots crossover hotspots? Exp. Cell Res. 318: 1347–1352. 22487095 10.1016/j.yexcr.2012.03.025

[bib71] Shinohara M. Gasior S. L. Bishop D. K. Shinohara A., 2000 Tid1/Rdh54 promotes colocalization of rad51 and dmc1 during meiotic recombination. Proc. Natl. Acad. Sci. USA 97: 10814–10819. 11005857 10.1073/pnas.97.20.10814 PMC27106

[bib72] Shinohara M. Oh S. D. Hunter N. Shinohara A., 2008 Crossover assurance and crossover interference are distinctly regulated by the ZMM proteins during yeast meiosis. Nat. Genet. 40: 299–309. 18297071 10.1038/ng.83

[bib73] Shonn M. A. McCarroll R. Murray A. W., 2000 Requirement of the spindle checkpoint for proper chromosome segregation in budding yeast meiosis. Science 289: 300–303. 10894778 10.1126/science.289.5477.300

[bib74] Snowden T. Acharya S. Butz C. Berardini M. Fishel R., 2004 hMSH4-hMSH5 recognizes Holliday junctions and forms a meiosis-specific sliding clamp that embraces homologous chromosomes. Mol. Cell 15: 437–451. 15304223 10.1016/j.molcel.2004.06.040

[bib75] Sonntag Brown M. Lim E. Chen C. Nishant K. T. Alani E., 2013 Genetic analysis of *mlh3* mutations reveals interactions between crossover promoting factors during meiosis in baker’s yeast. G3 Bethesda 3: 9–22. 23316435 10.1534/g3.112.004622 PMC3538346

[bib76] Stahl F. W. Foss H. M. Young L. S. Borts R. H. Abdullah M. F., 2004 Does crossover interference count in *Saccharomyces cerevisiae?* Genetics 168: 35–48. 15454525 10.1534/genetics.104.027789 PMC1448104

[bib77] Subramanian V. V. MacQueen A. J. Vader G. Shinohara M. Sanchez A., 2016 Chromosome synapsis alleviates Mek1-dependent suppression of meiotic DNA repair. PLoS Biol. 14: e1002369. 26870961 10.1371/journal.pbio.1002369 PMC4752329

[bib78] Sun X. Huang L. Markowitz T. E. Blitzblau H. G. Chen D., 2015 Transcription dynamically patterns the meiotic chromosome-axis interface. Elife 4: e07424. 10.7554/eLife.07424 PMC4530585 26258962

[bib79] Sym M. Roeder G. S., 1994 Crossover interference is abolished in the absence of a synaptonemal complex protein. Cell 79: 283–292. 7954796 10.1016/0092-8674(94)90197-x

[bib80] Terasawa M. Ogawa H. Tsukamoto Y. Shinohara M. Shirahige K., 2007 Meiotic recombination-related DNA synthesis and its implications for cross-over and non-cross-over recombinant formation. Proc. Natl. Acad. Sci. USA 104: 5965–5970. 17384152 10.1073/pnas.0611490104 PMC1851600

[bib81] Vader G. Blitzblau H. G. Tame M. A. Falk J. E. Curtin L., 2011 Protection of repetitive DNA borders from self-induced meiotic instability. Nature 477: 115–119. 21822291 10.1038/nature10331 PMC3166416

[bib82] Wang S. Zickler D. Kleckner N. Zhang L., 2015 Meiotic crossover patterns: obligatory crossover, interference and homeostasis in a single process. Cell Cycle 14: 305–314. 25590558 10.4161/15384101.2014.991185 PMC4353236

[bib83] Wojtasz L. Daniel K. Roig I. Bolcun-Filas E. Xu H., 2009 Mouse HORMAD1 and HORMAD2, two conserved meiotic chromosomal proteins, are depleted from synapsed chromosome axes with the help of TRIP13 AAA-ATPase. PLoS Genet. 5: e1000702. 19851446 10.1371/journal.pgen.1000702 PMC2758600

[bib84] Wu H. Y. Burgess S. M., 2006 Two distinct surveillance mechanisms monitor meiotic chromosome metabolism in budding yeast. Curr. Biol. 16: 2473–2479. 17174924 10.1016/j.cub.2006.10.069 PMC1876825

[bib85] Zakharyevich K. Tang S. Ma Y. Hunter N., 2012 Delineation of joint molecule resolution pathways in meiosis identifies a crossover-specific resolvase. Cell 149: 334–347. 22500800 10.1016/j.cell.2012.03.023 PMC3377385

[bib86] Zanders S. Alani E., 2009 The pch2Delta mutation in baker’s yeast alters meiotic crossover levels and confers a defect in crossover interference. PLoS Genet. 5: e1000571. 19629178 10.1371/journal.pgen.1000571 PMC2709914

[bib87] Zanders S. Sonntag Brown M. Chen C. Alani E., 2011 Pch2 modulates chromatid partner choice during meiotic double-strand break repair in *Saccharomyces cerevisiae*. Genetics 188: 511–521. 21515575 10.1534/genetics.111.129031 PMC3176543

[bib88] Zhang L. Wang S. Yin S. Hong S. Kim K. P., 2014 Topoisomerase II mediates meiotic crossover interference. Nature 511: 551–556. 25043020 10.1038/nature13442 PMC4128387

[bib89] Zickler D. Kleckner N., 2016 A few of our favorite things: pairing, the bouquet, crossover interference and evolution of meiosis. Semin. Cell Dev. Biol. 54: 135–148. 26927691 10.1016/j.semcdb.2016.02.024 PMC4867269

