# Supplementary material for: Modulating Crossover Frequency and Interference for Obligate Crossovers in Saccharomyces cerevisiae Meiosis
Source: G3 (Bethesda). 2017 Mar 17;7(5):1511–24. doi: 10.1534/g3.117.040071 (PMC5427503; doi:10.1534/g3.117.040071)
Supplement: Supplementary file 1 [file 1511FigureS1.pptx]

## Slide 1
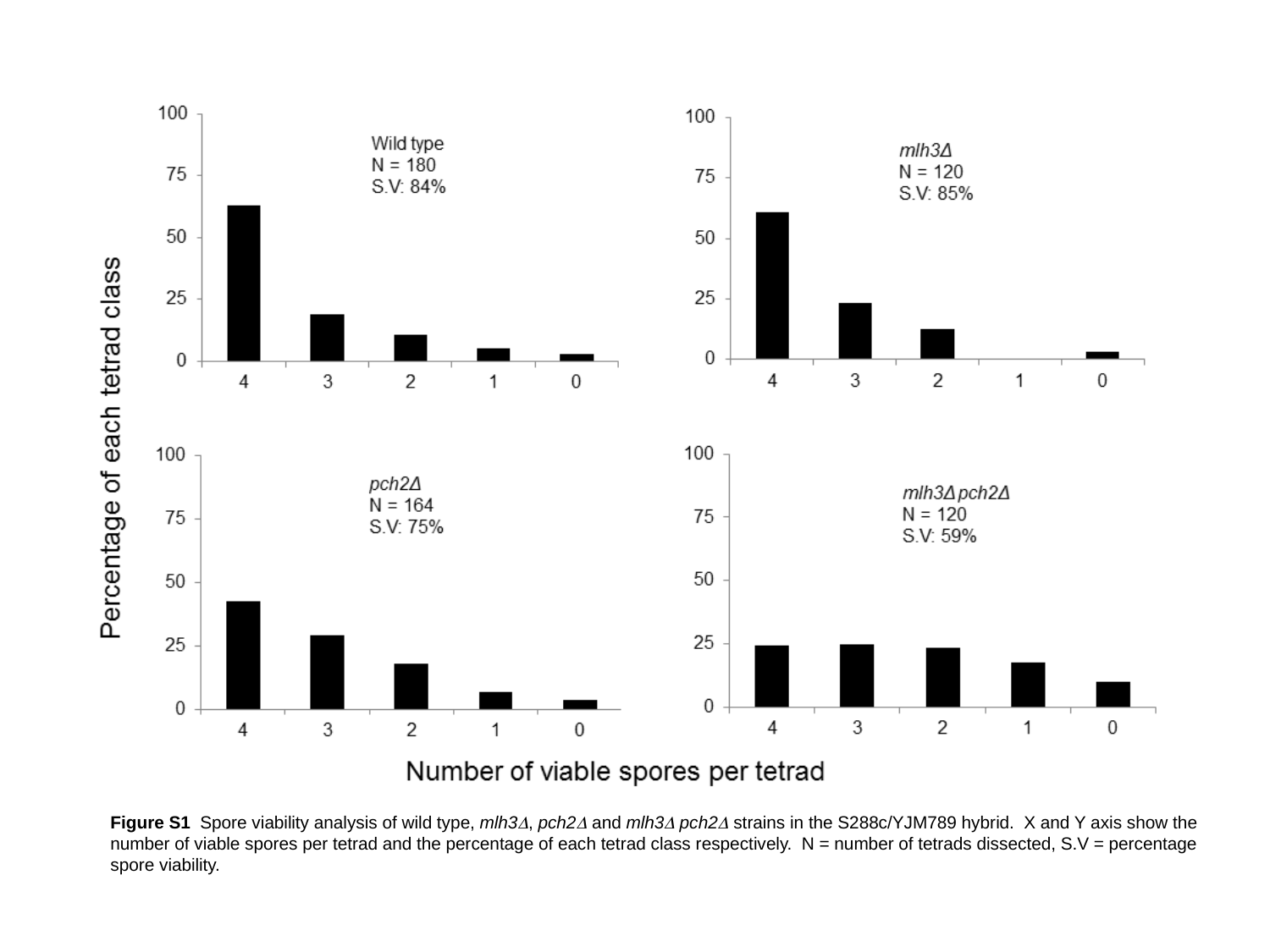

Figure S1 Spore viability analysis of wild type, mlh3Δ, pch2Δ and mlh3Δ pch2Δ strains in the S288c/YJM789 hybrid. X and Y axis show the number of viable spores per tetrad and the percentage of each tetrad class respectively. N = number of tetrads dissected, S.V = percentage spore viability.
