# Supplementary material for: Modulating Crossover Frequency and Interference for Obligate Crossovers in Saccharomyces cerevisiae Meiosis
Source: G3 (Bethesda). 2017 Mar 17;7(5):1511–24. doi: 10.1534/g3.117.040071 (PMC5427503; doi:10.1534/g3.117.040071)
Supplement: Supplementary file 2 [file 1511FigureS2.pptx]

## Slide 1
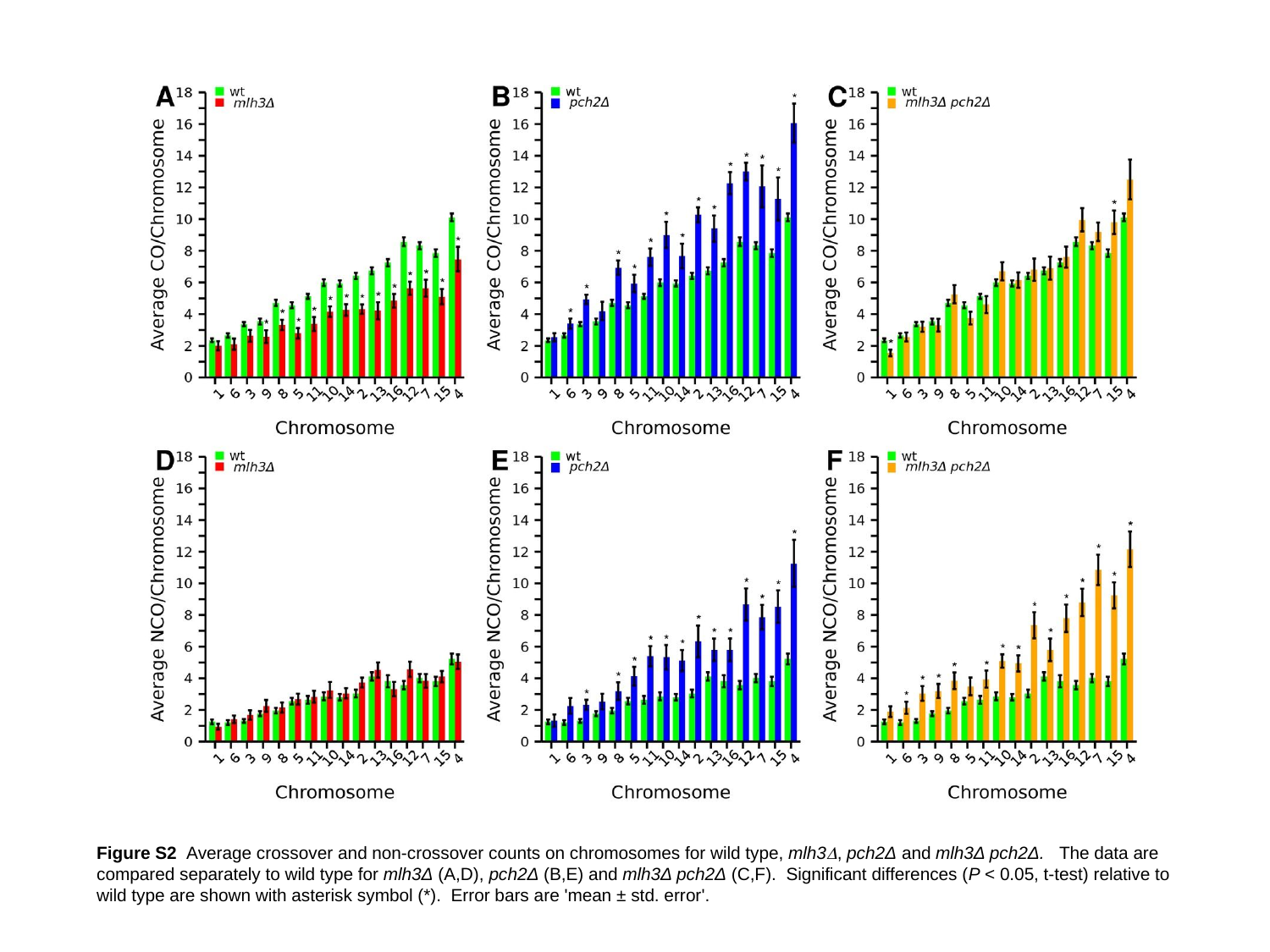

Figure S2 Average crossover and non-crossover counts on chromosomes for wild type, mlh3Δ, pch2Δ and mlh3Δ pch2Δ. The data are compared separately to wild type for mlh3Δ (A,D), pch2Δ (B,E) and mlh3Δ pch2Δ (C,F). Significant differences (P < 0.05, t-test) relative to wild type are shown with asterisk symbol (*). Error bars are 'mean ± std. error'.
