# Supplementary material for: Modulating Crossover Frequency and Interference for Obligate Crossovers in Saccharomyces cerevisiae Meiosis
Source: G3 (Bethesda). 2017 Mar 17;7(5):1511–24. doi: 10.1534/g3.117.040071 (PMC5427503; doi:10.1534/g3.117.040071)
Supplement: Supplementary file 3 [file 1511FigureS3.pptx]

## Slide 1
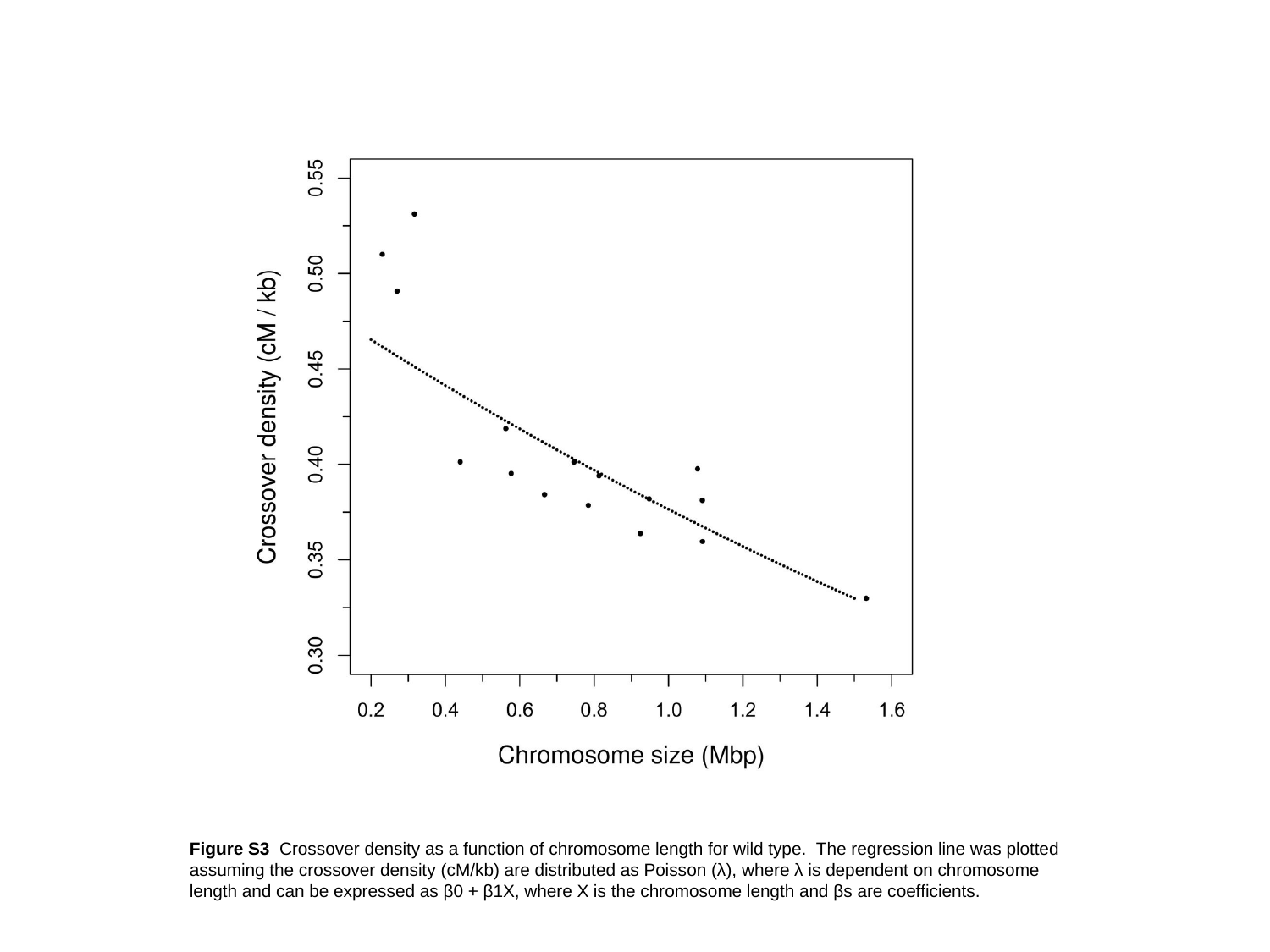

Figure S3 Crossover density as a function of chromosome length for wild type. The regression line was plotted assuming the crossover density (cM/kb) are distributed as Poisson (λ), where λ is dependent on chromosome length and can be expressed as β0 + β1X, where X is the chromosome length and βs are coefficients.
