# Supplementary material for: Modulating Crossover Frequency and Interference for Obligate Crossovers in Saccharomyces cerevisiae Meiosis
Source: G3 (Bethesda). 2017 Mar 17;7(5):1511–24. doi: 10.1534/g3.117.040071 (PMC5427503; doi:10.1534/g3.117.040071)
Supplement: Supplementary file 4 [file 1511FigureS4.pptx]

## Slide 1
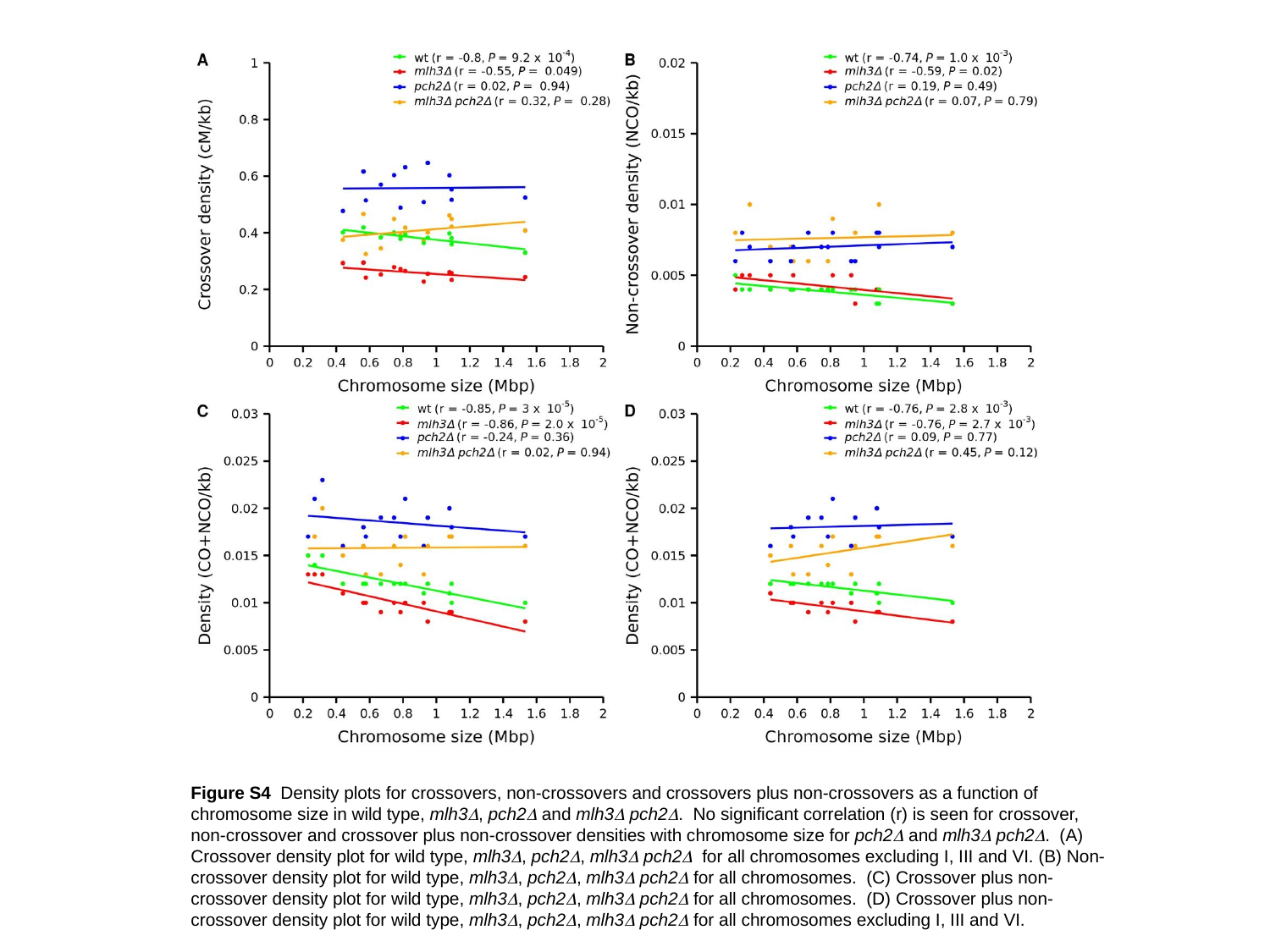

Figure S4 Density plots for crossovers, non-crossovers and crossovers plus non-crossovers as a function of chromosome size in wild type, mlh3Δ, pch2Δ and mlh3Δ pch2Δ. No significant correlation (r) is seen for crossover, non-crossover and crossover plus non-crossover densities with chromosome size for pch2Δ and mlh3Δ pch2Δ. (A) Crossover density plot for wild type, mlh3Δ, pch2Δ, mlh3Δ pch2Δ for all chromosomes excluding I, III and VI. (B) Non-crossover density plot for wild type, mlh3Δ, pch2Δ, mlh3Δ pch2Δ for all chromosomes. (C) Crossover plus non-crossover density plot for wild type, mlh3Δ, pch2Δ, mlh3Δ pch2Δ for all chromosomes. (D) Crossover plus non-crossover density plot for wild type, mlh3Δ, pch2Δ, mlh3Δ pch2Δ for all chromosomes excluding I, III and VI.
