# Supplementary material for: Modulating Crossover Frequency and Interference for Obligate Crossovers in Saccharomyces cerevisiae Meiosis
Source: G3 (Bethesda). 2017 Mar 17;7(5):1511–24. doi: 10.1534/g3.117.040071 (PMC5427503; doi:10.1534/g3.117.040071)
Supplement: Supplementary file 7 [file 1511FigureS7.pptx]

## Slide 1
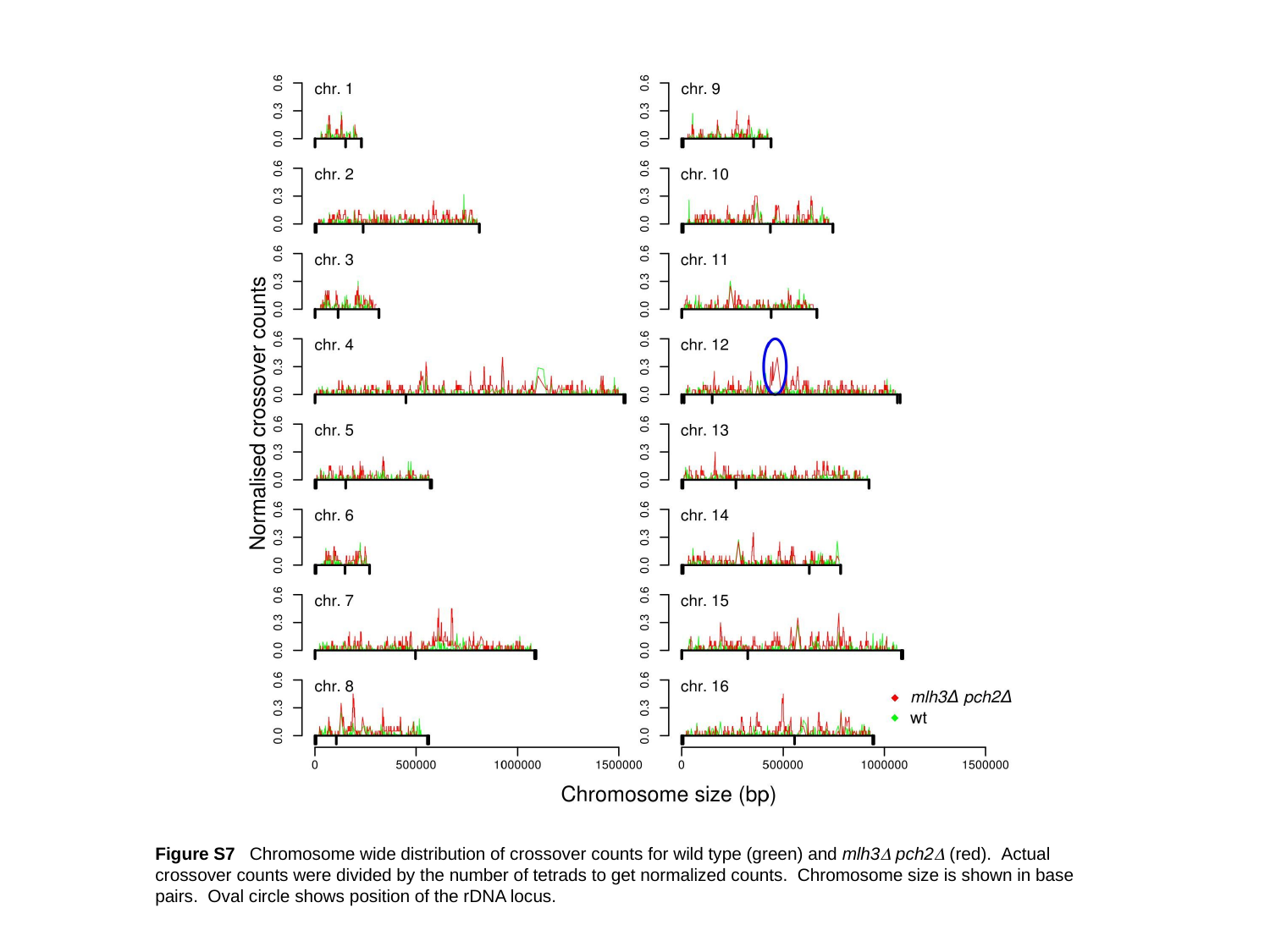

Figure S7 Chromosome wide distribution of crossover counts for wild type (green) and mlh3Δ pch2Δ (red). Actual crossover counts were divided by the number of tetrads to get normalized counts. Chromosome size is shown in base pairs. Oval circle shows position of the rDNA locus.
