# Supplementary material for: Modulating Crossover Frequency and Interference for Obligate Crossovers in Saccharomyces cerevisiae Meiosis
Source: G3 (Bethesda). 2017 Mar 17;7(5):1511–24. doi: 10.1534/g3.117.040071 (PMC5427503; doi:10.1534/g3.117.040071)
Supplement: Supplementary file 8 [file 1511FigureS8.pptx]

## Slide 1
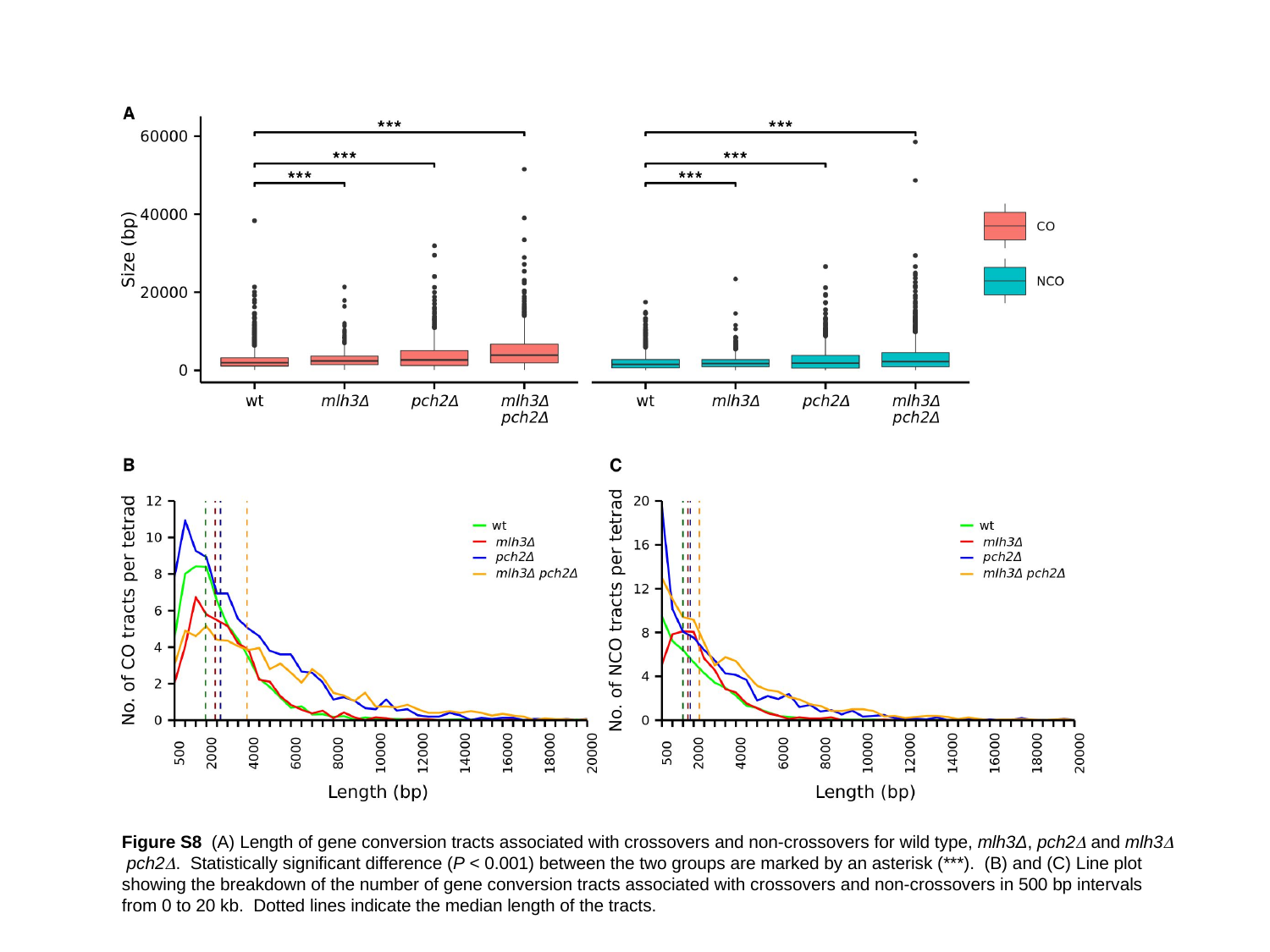

Figure S8 (A) Length of gene conversion tracts associated with crossovers and non-crossovers for wild type, mlh3Δ, pch2Δ and mlh3Δ pch2Δ. Statistically significant difference (P < 0.001) between the two groups are marked by an asterisk (***). (B) and (C) Line plot showing the breakdown of the number of gene conversion tracts associated with crossovers and non-crossovers in 500 bp intervals from 0 to 20 kb. Dotted lines indicate the median length of the tracts.
