# Supplementary material for: Modulating Crossover Frequency and Interference for Obligate Crossovers in Saccharomyces cerevisiae Meiosis
Source: G3 (Bethesda). 2017 Mar 17;7(5):1511–24. doi: 10.1534/g3.117.040071 (PMC5427503; doi:10.1534/g3.117.040071)
Supplement: Supplementary file 9 [file 1511FigureS9.pdf]

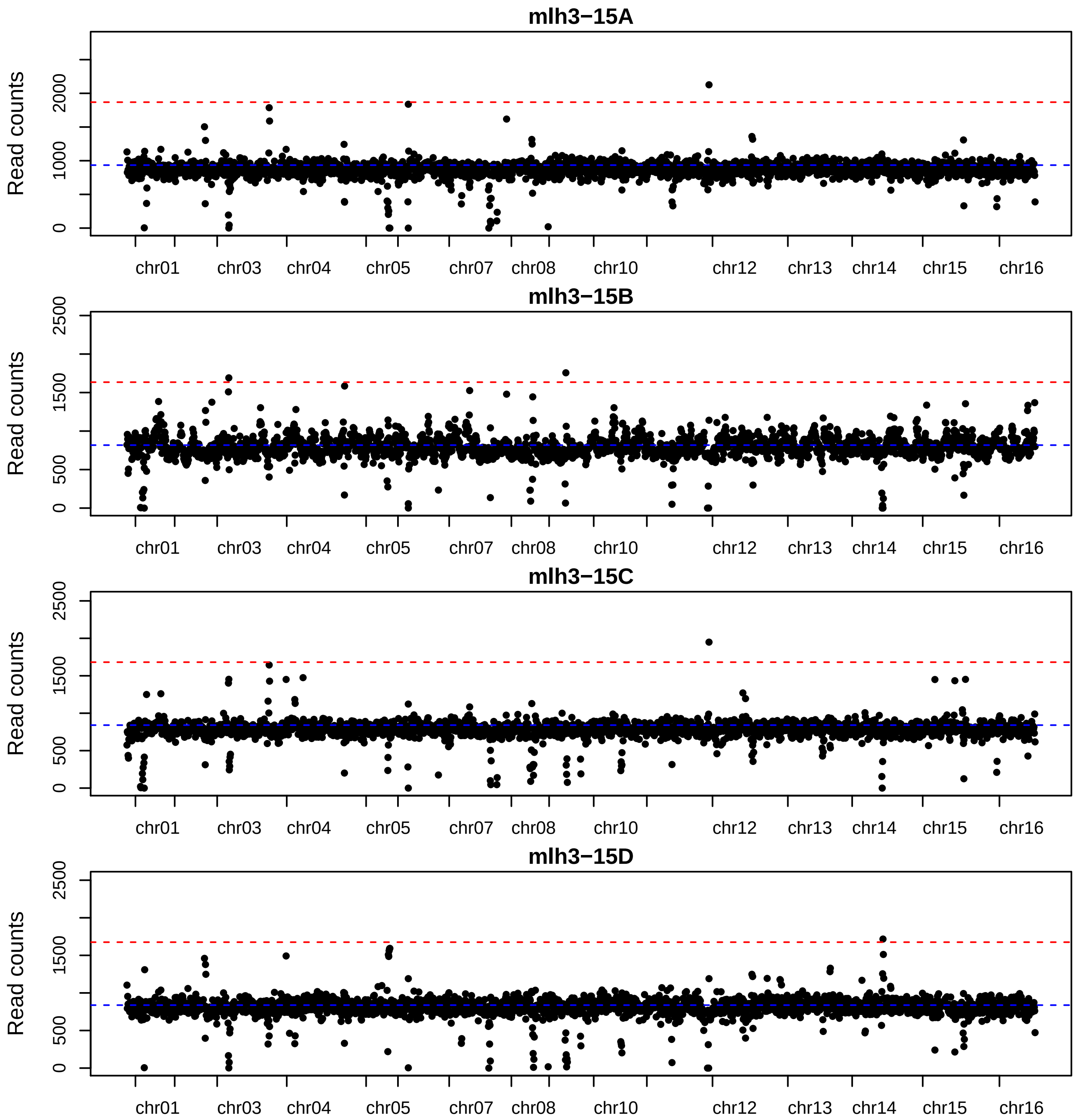
mlh3−15A
Read counts
0
1000
2000
chr01
chr03
chr04
chr05
chr07
chr08
chr10
chr12
chr13
chr14
chr15
chr16
mlh3−15B
Read counts
0
500
1500
2500
mlh3−15C
Read counts
0
500
1500
2500
mlh3−15D
Read counts
0
500
1500
2500

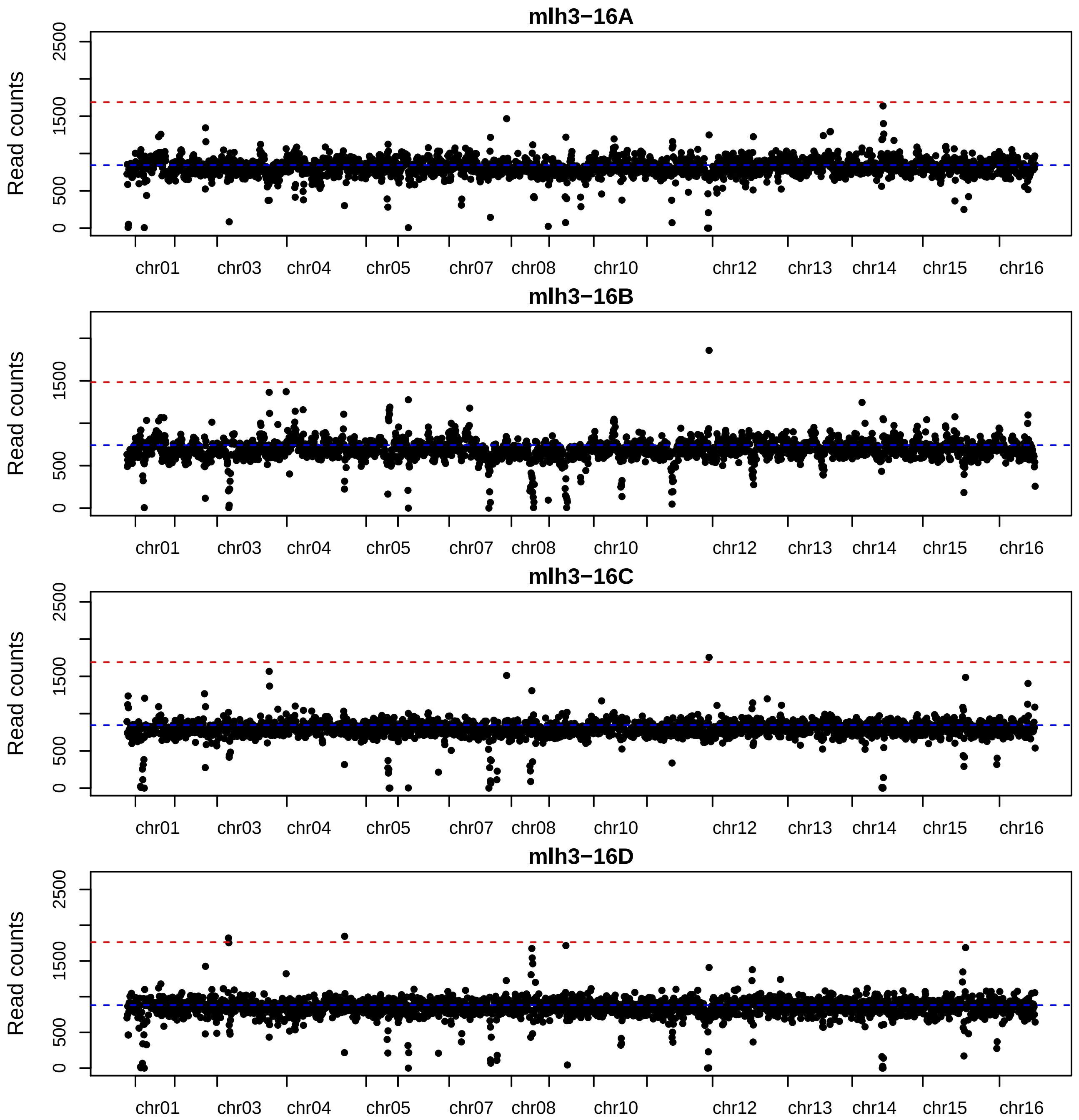
mlh3−16A
mlh3−16B
mlh3−16C
mlh3−16D
Read counts
0
500
1500
2500
chr01
chr03
chr04
chr05
chr07
chr08
chr10
chr12
chr13
chr14
chr15
chr16

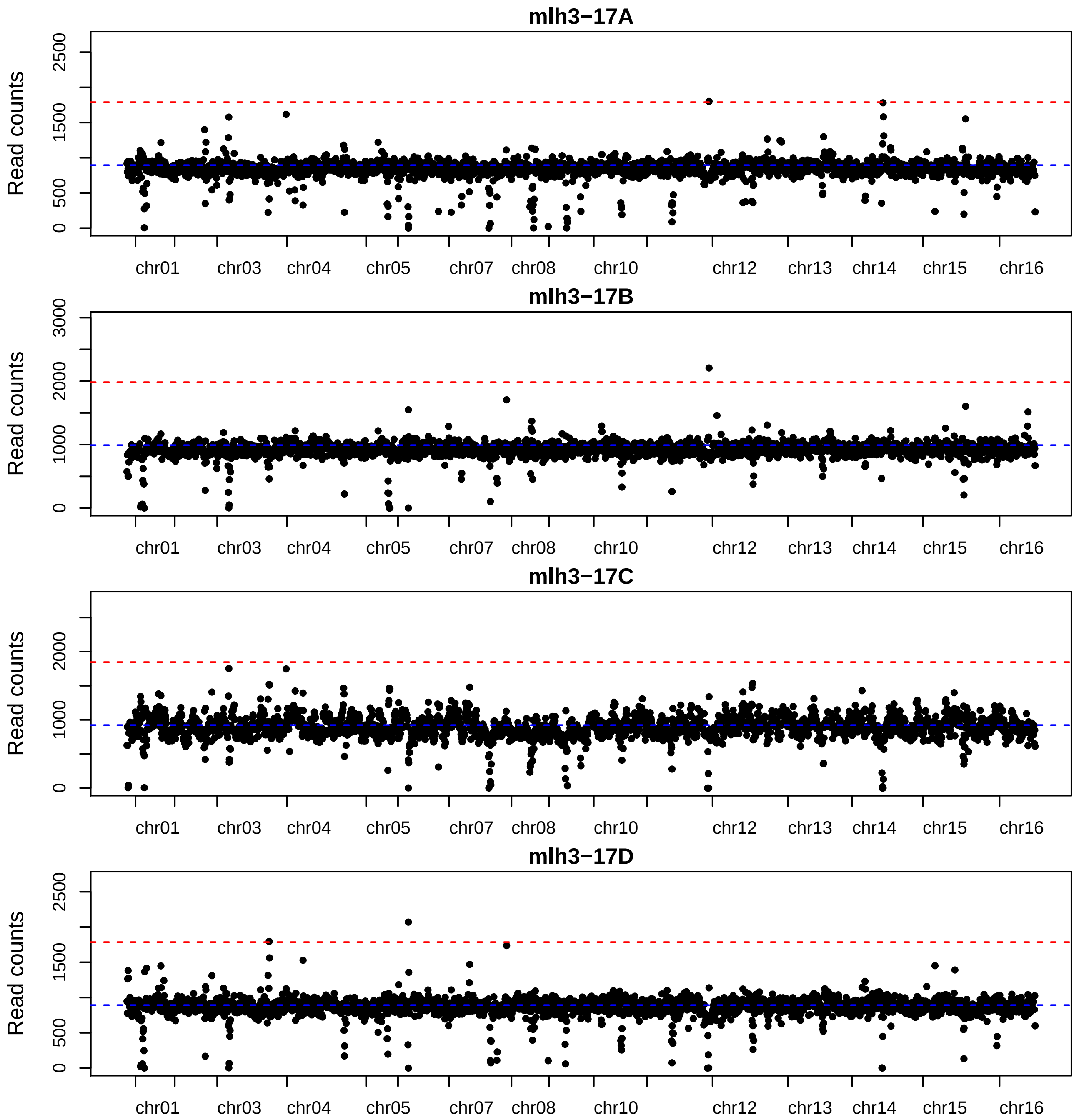
mlh3−17A
mlh3−17B
mlh3−17C
mlh3−17D
Read counts
0
500
1000
1500
2000
2500
3000
chr01
chr03
chr04
chr05
chr07
chr08
chr10
chr12
chr13
chr14
chr15
chr16

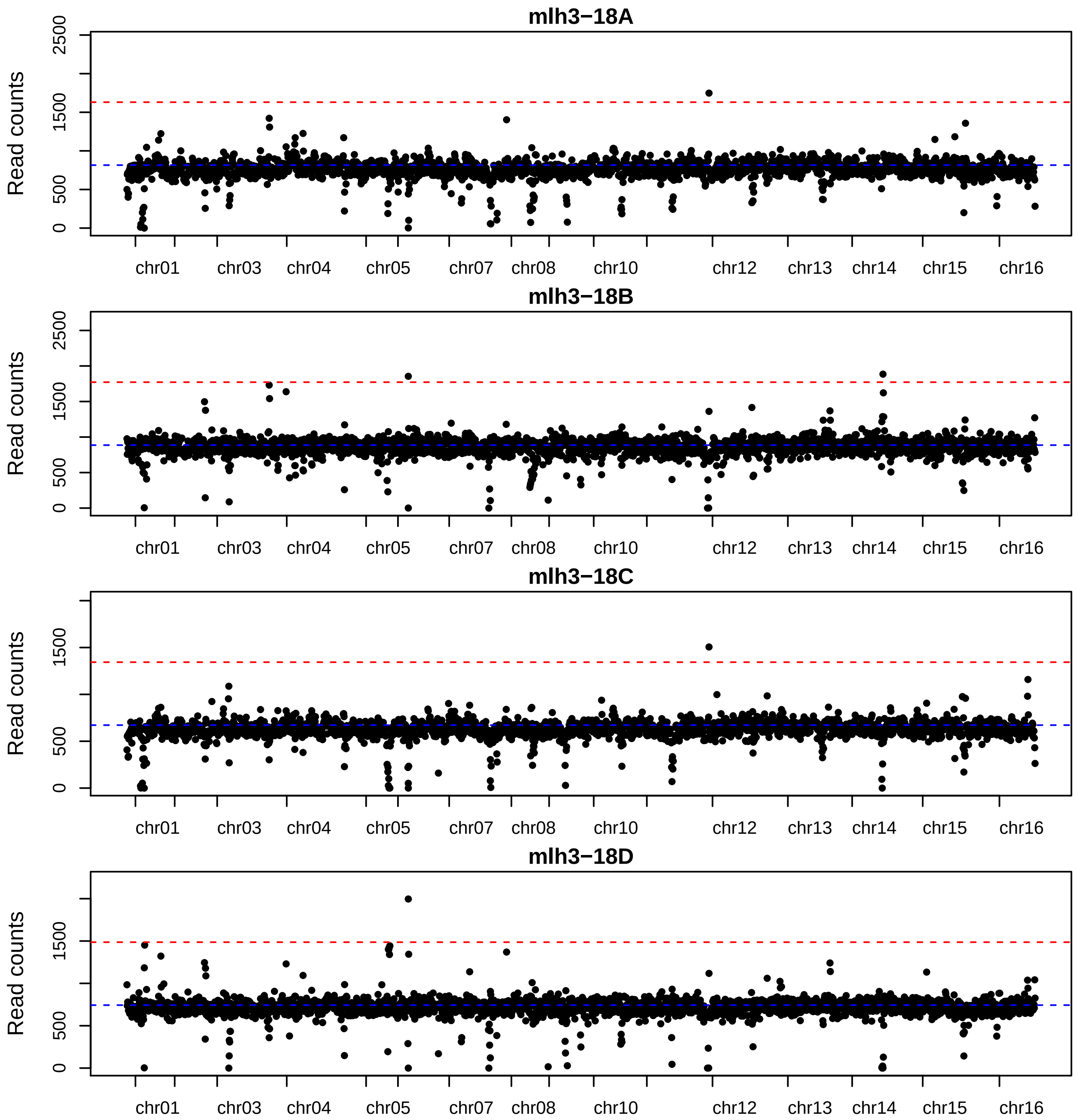
mlh3−18A
mlh3−18B
mlh3−18C
mlh3−18D
Read counts
0
500
1500
2500
chr01
chr03
chr04
chr05
chr07
chr08
chr10
chr12
chr13
chr14
chr15
chr16

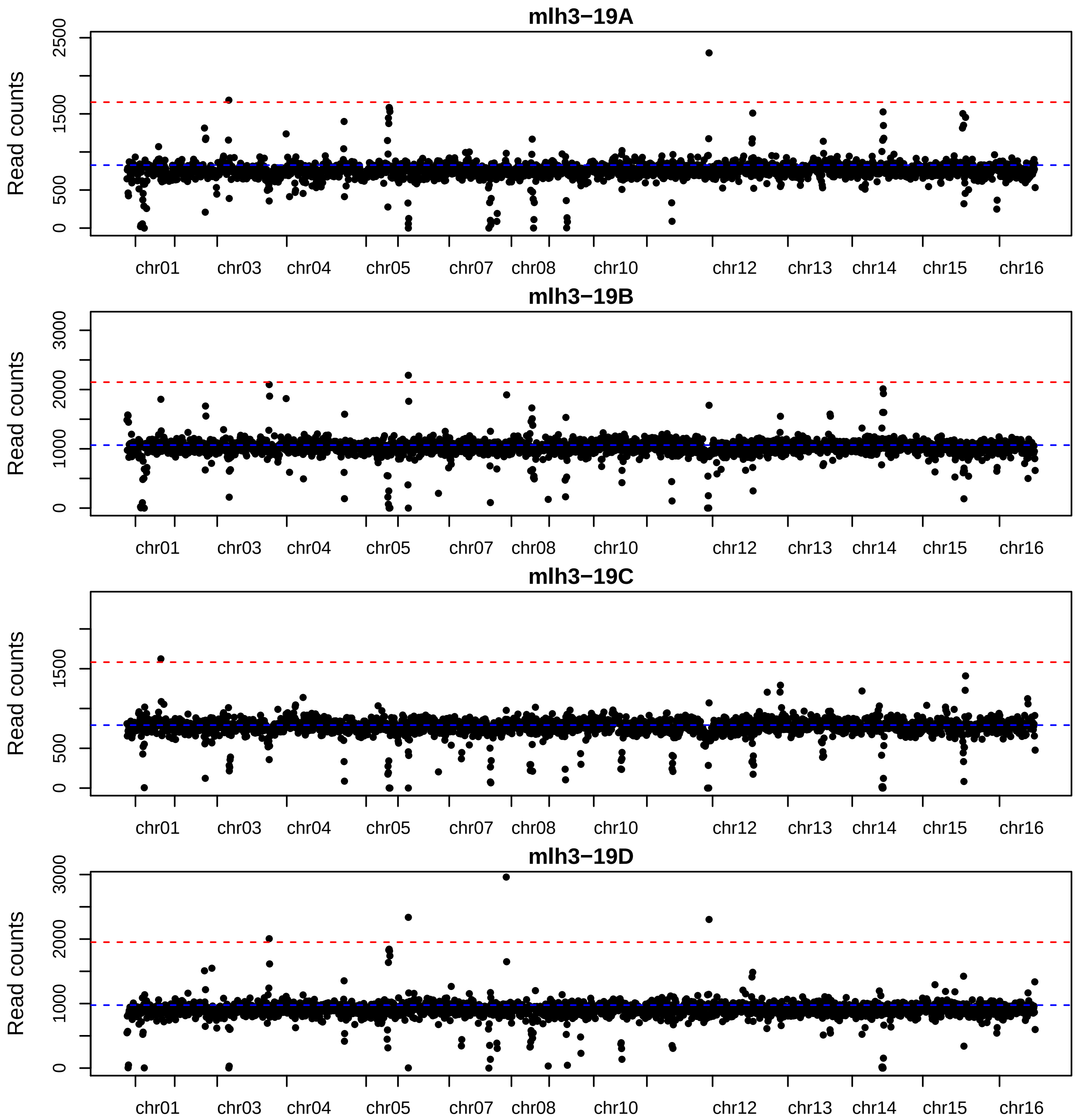

mlh3−19A
Read counts
0
500
1500
2500
chr01
chr03
chr04
chr05
chr07
chr08
chr10
chr12
chr13
chr14
chr15
chr16
mlh3−19B
Read counts
0
1000
2000
3000
mlh3−19C
Read counts
0
500
1500
mlh3−19D
Read counts
0
1000
2000
3000

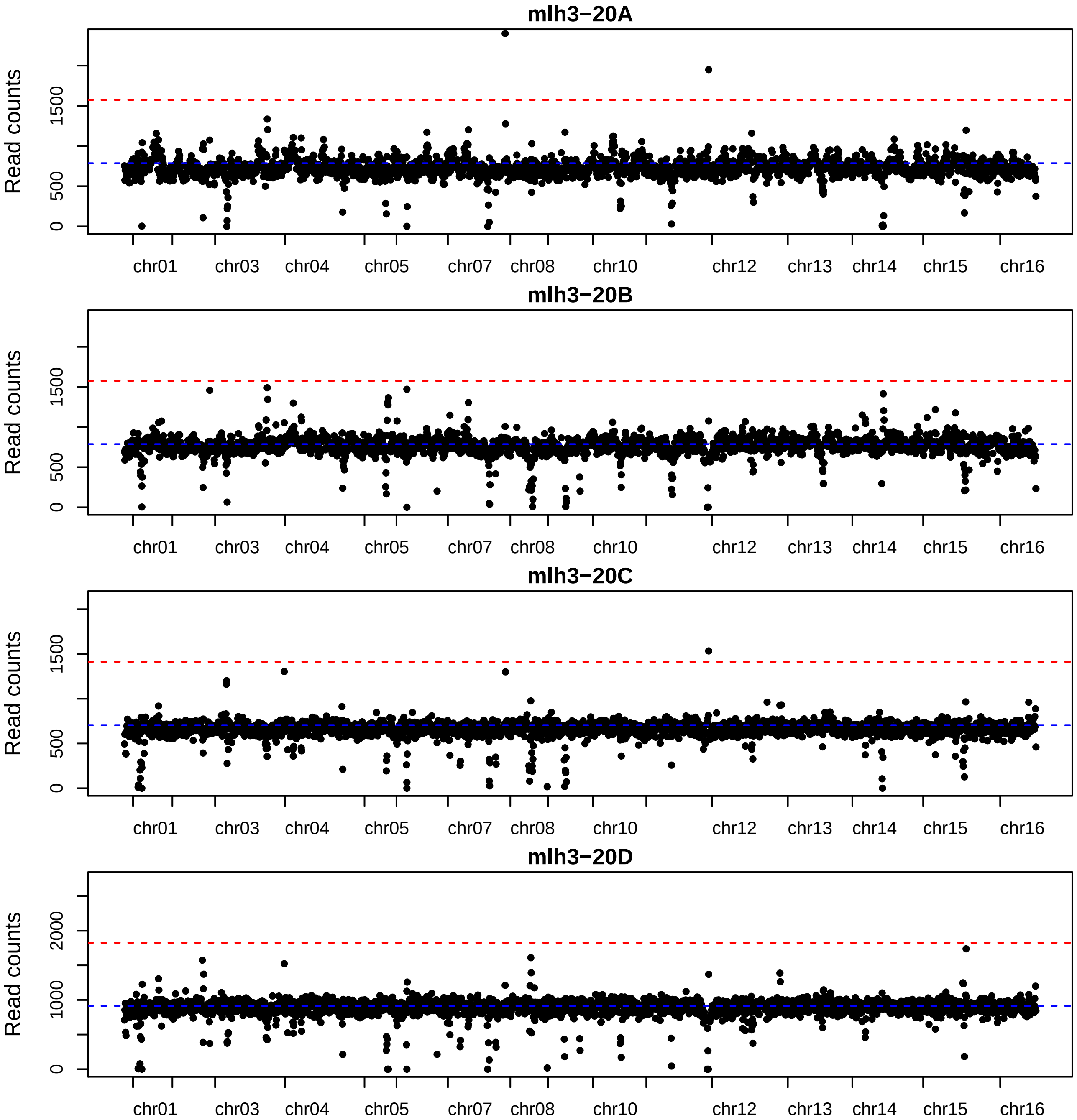
mlh3−20A
mlh3−20B
mlh3−20C
mlh3−20D
Read counts
0
500
1000
1500
2000
chr01
chr03
chr04
chr05
chr07
chr08
chr10
chr12
chr13
chr14
chr15
chr16

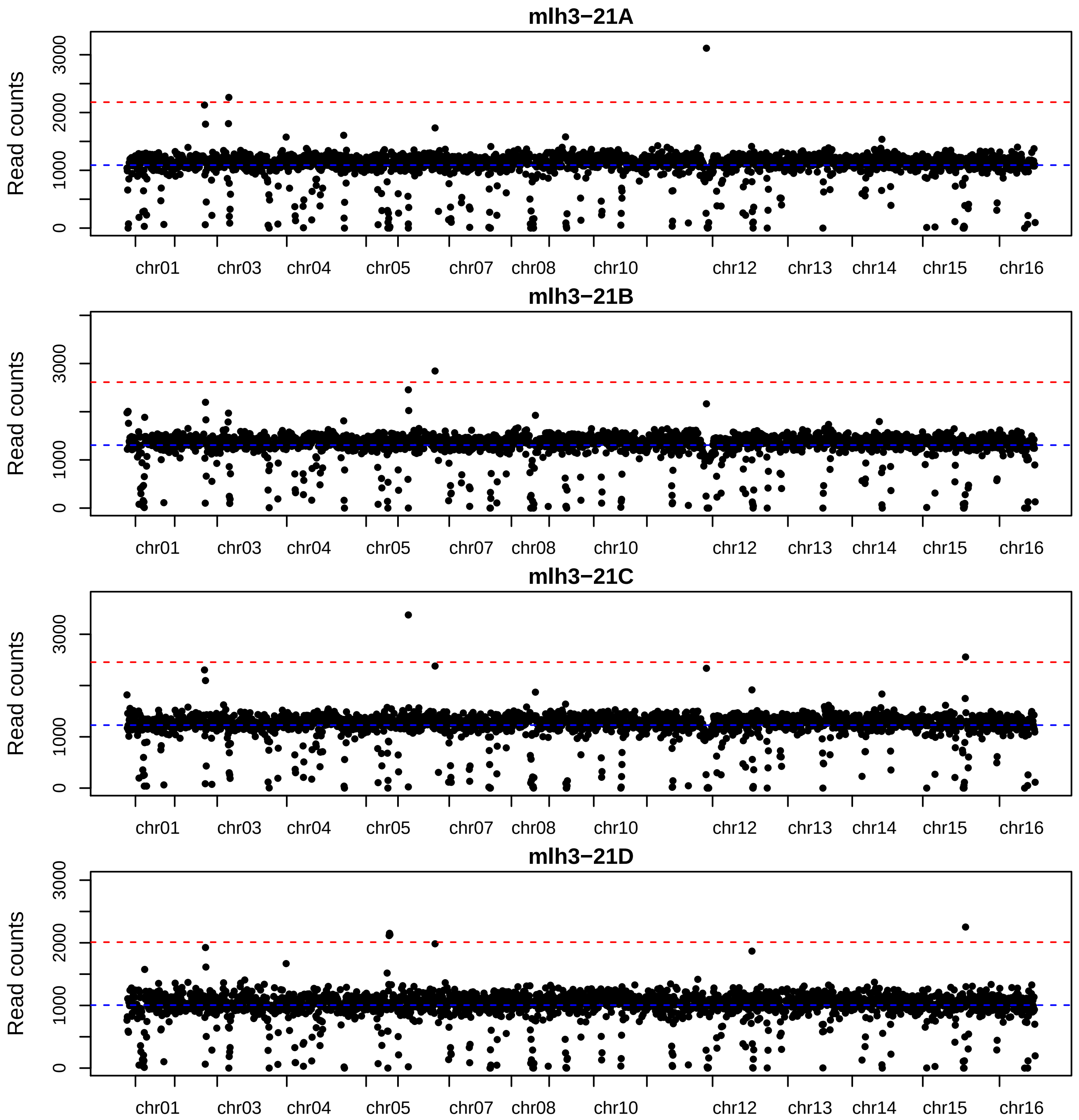

mlh3−21A
Read counts
0
1000
2000
3000
chr01
chr03
chr04
chr05
chr07
chr08
chr10
chr12
chr13
chr14
chr15
chr16
mlh3−21B
Read counts
0
1000
3000
chr01
chr03
chr04
chr05
chr07
chr08
chr10
chr12
chr13
chr14
chr15
chr16
mlh3−21C
Read counts
0
1000
3000
chr01
chr03
chr04
chr05
chr07
chr08
chr10
chr12
chr13
chr14
chr15
chr16
mlh3−21D
Read counts
0
1000
2000
3000
chr01
chr03
chr04
chr05
chr07
chr08
chr10
chr12
chr13
chr14
chr15
chr16

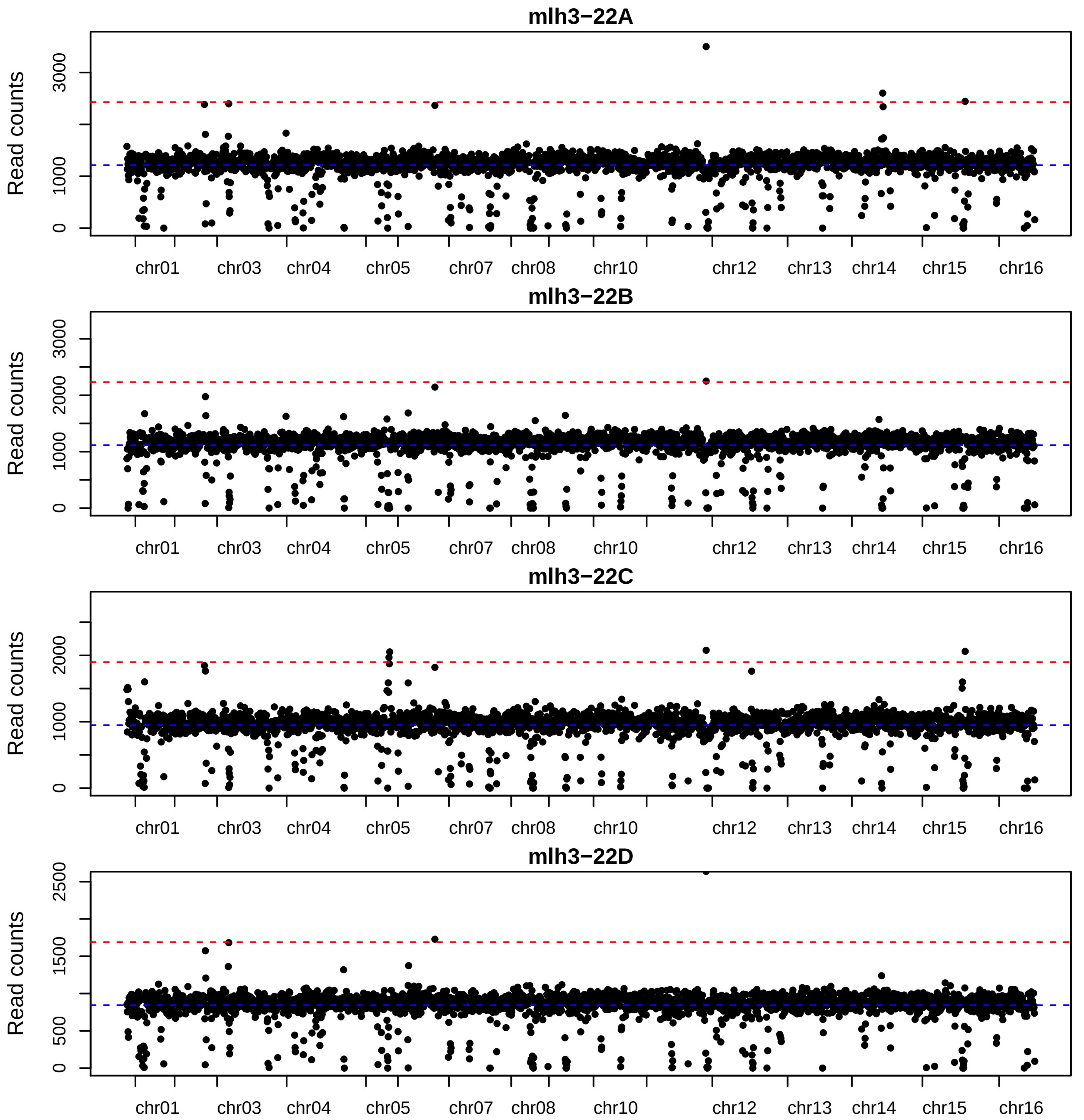
mlh3−22A
mlh3−22B
mlh3−22C
mlh3−22D
Read counts
0
500
1000
1500
2000
2500
3000
chr01
chr03
chr04
chr05
chr07
chr08
chr10
chr12
chr13
chr14
chr15
chr16

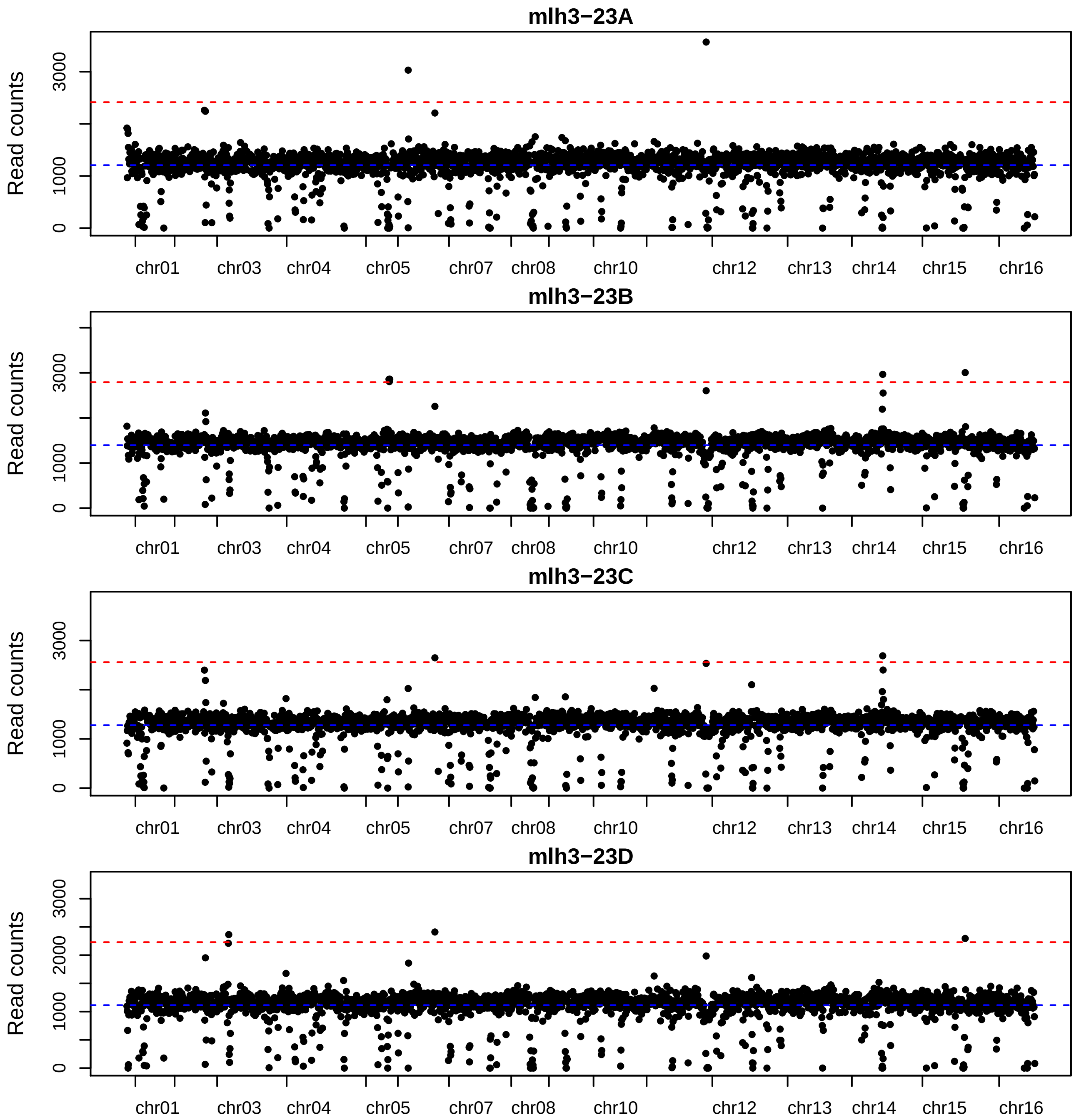
mlh3−23A
mlh3−23B
mlh3−23C
mlh3−23D
Read counts
0
1000
2000
3000
chr01
chr03
chr04
chr05
chr07
chr08
chr10
chr12
chr13
chr14
chr15
chr16

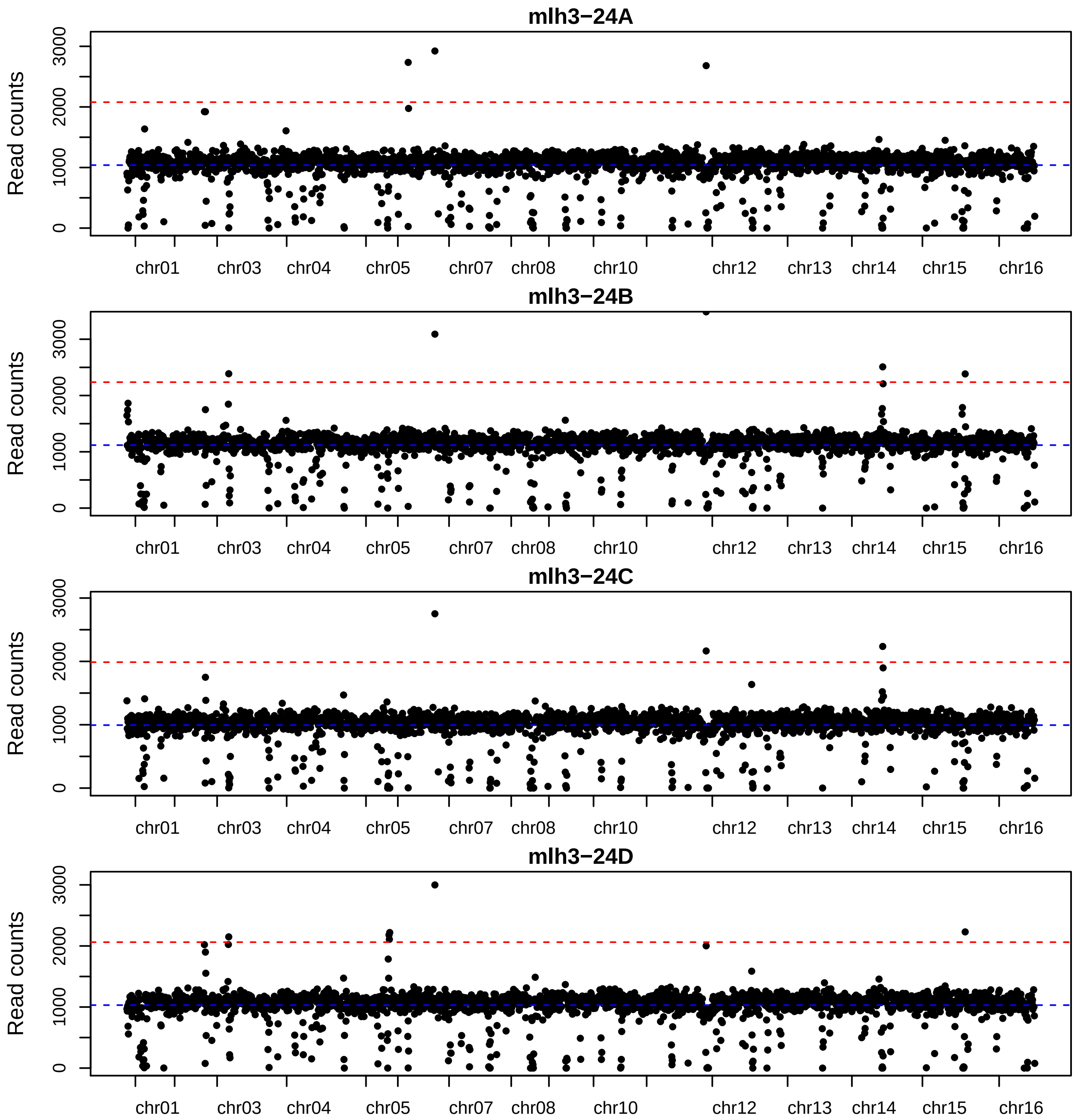
mlh3−24A
Read counts
0
1000
2000
3000
chr01
chr03
chr04
chr05
chr07
chr08
chr10
chr12
chr13
chr14
chr15
chr16
mlh3−24B
mlh3−24C
mlh3−24D

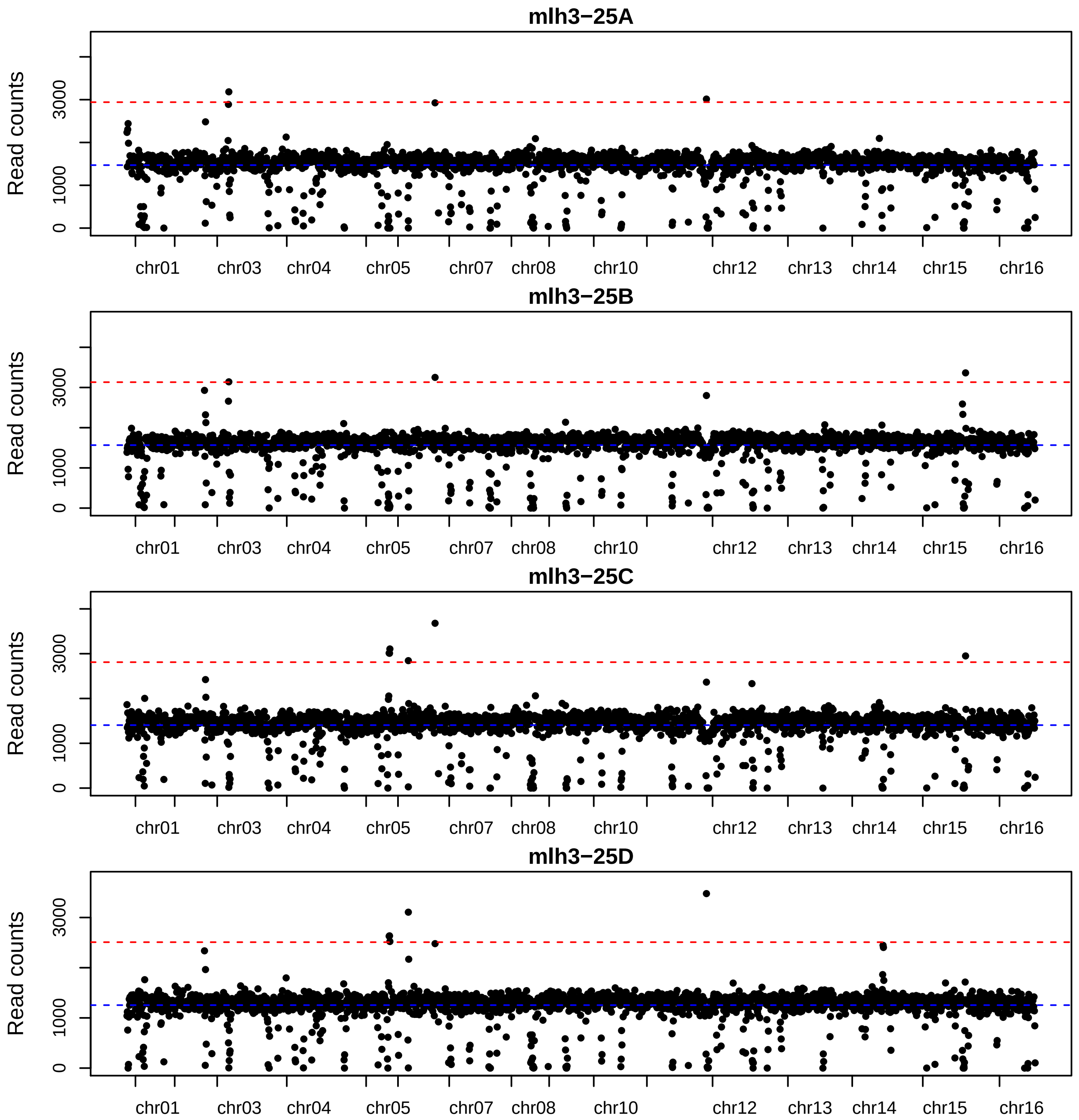
mlh3−25A
mlh3−25B
mlh3−25C
mlh3−25D
Read counts
0
1000
3000
chr01
chr03
chr04
chr05
chr07
chr08
chr10
chr12
chr13
chr14
chr15
chr16

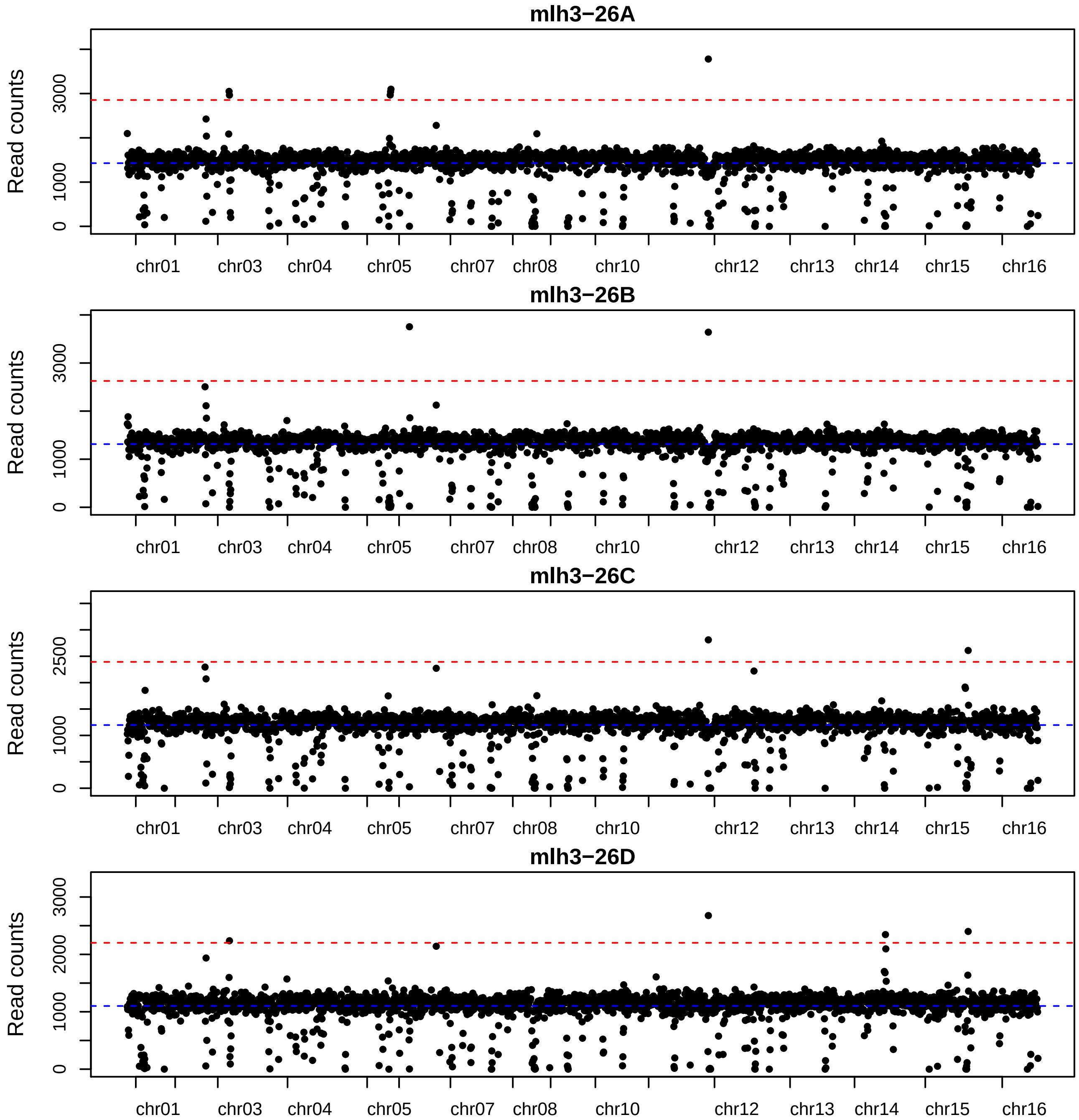
mlh3−26A
mlh3−26B
mlh3−26C
mlh3−26D
Read counts
0
1000
2000
2500
3000
chr01
chr03
chr04
chr05
chr07
chr08
chr10
chr12
chr13
chr14
chr15
chr16

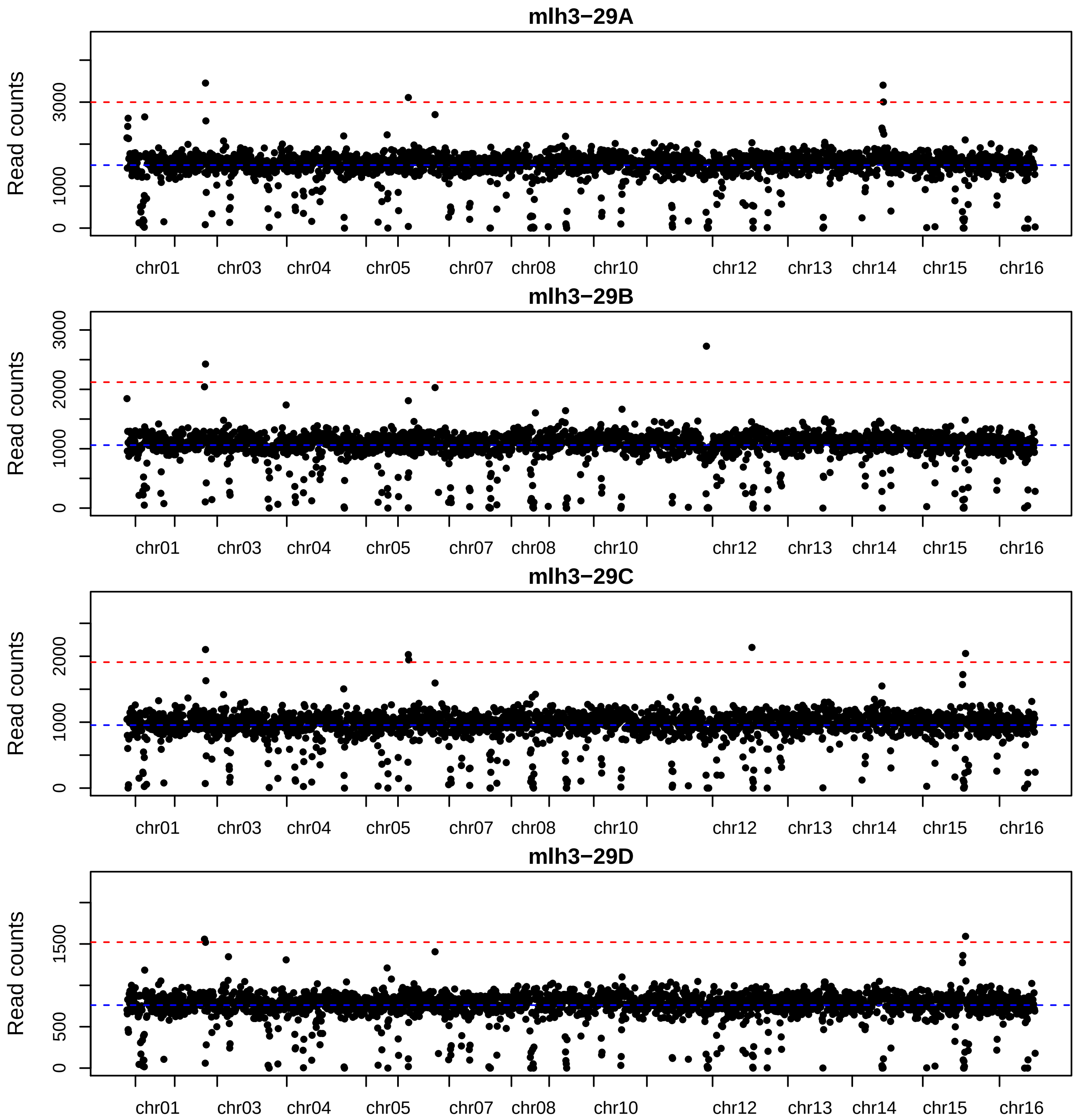

mlh3−29A
Read counts
0
1000
3000
chr01
chr03
chr04
chr05
chr07
chr08
chr10
chr12
chr13
chr14
chr15
chr16
mlh3−29B
Read counts
0
1000
2000
3000
chr01
chr03
chr04
chr05
chr07
chr08
chr10
chr12
chr13
chr14
chr15
chr16
mlh3−29C
Read counts
0
1000
2000
chr01
chr03
chr04
chr05
chr07
chr08
chr10
chr12
chr13
chr14
chr15
chr16
mlh3−29D
Read counts
0
500
1500
chr01
chr03
chr04
chr05
chr07
chr08
chr10
chr12
chr13
chr14
chr15
chr16

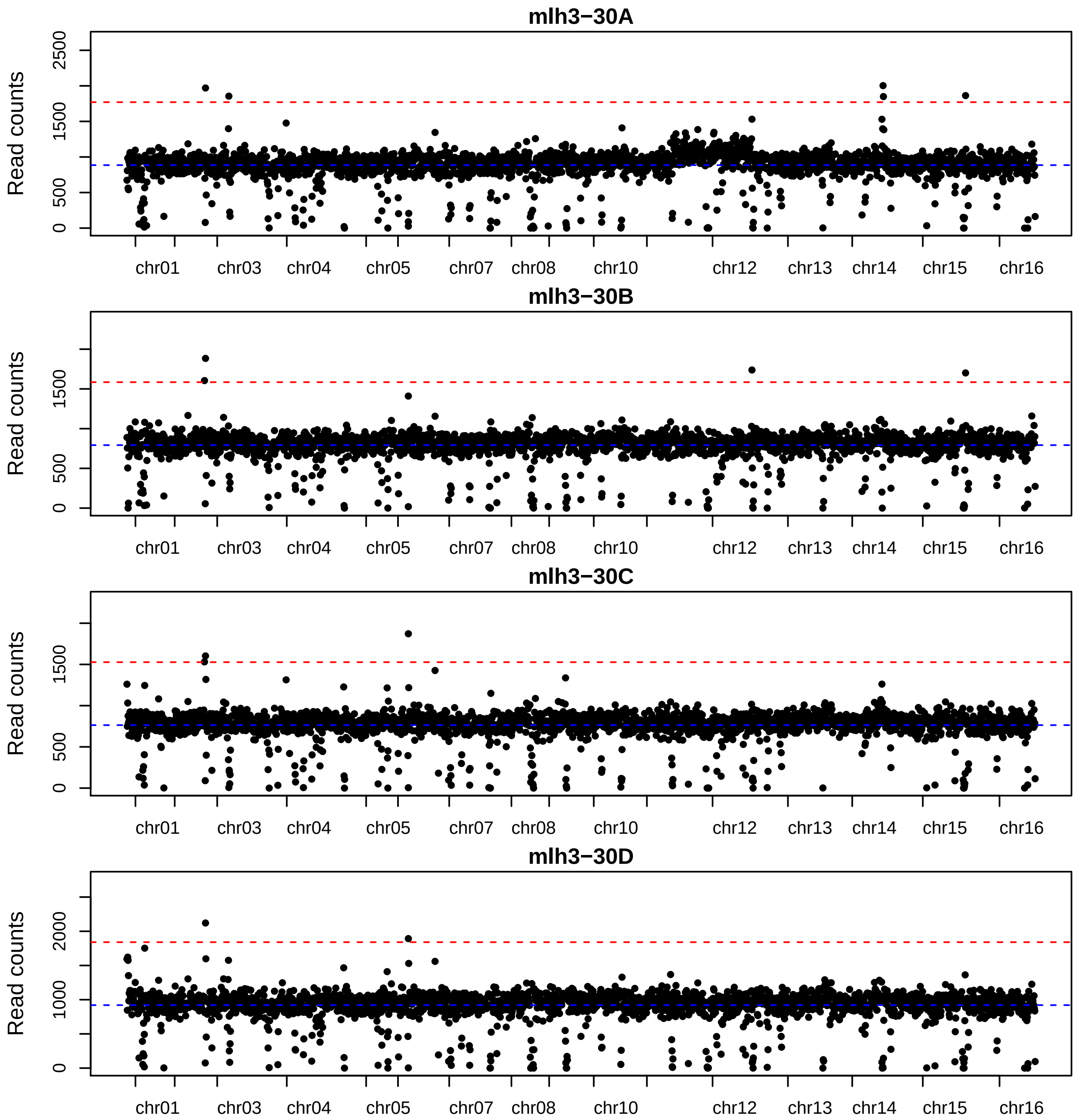
mlh3−30A
Read counts
0
500
1500
2500
chr01
chr03
chr04
chr05
chr07
chr08
chr10
chr12
chr13
chr14
chr15
chr16
mlh3−30B
Read counts
0
500
1500
mlh3−30C
Read counts
0
500
1500
mlh3−30D
Read counts
0
1000
2000

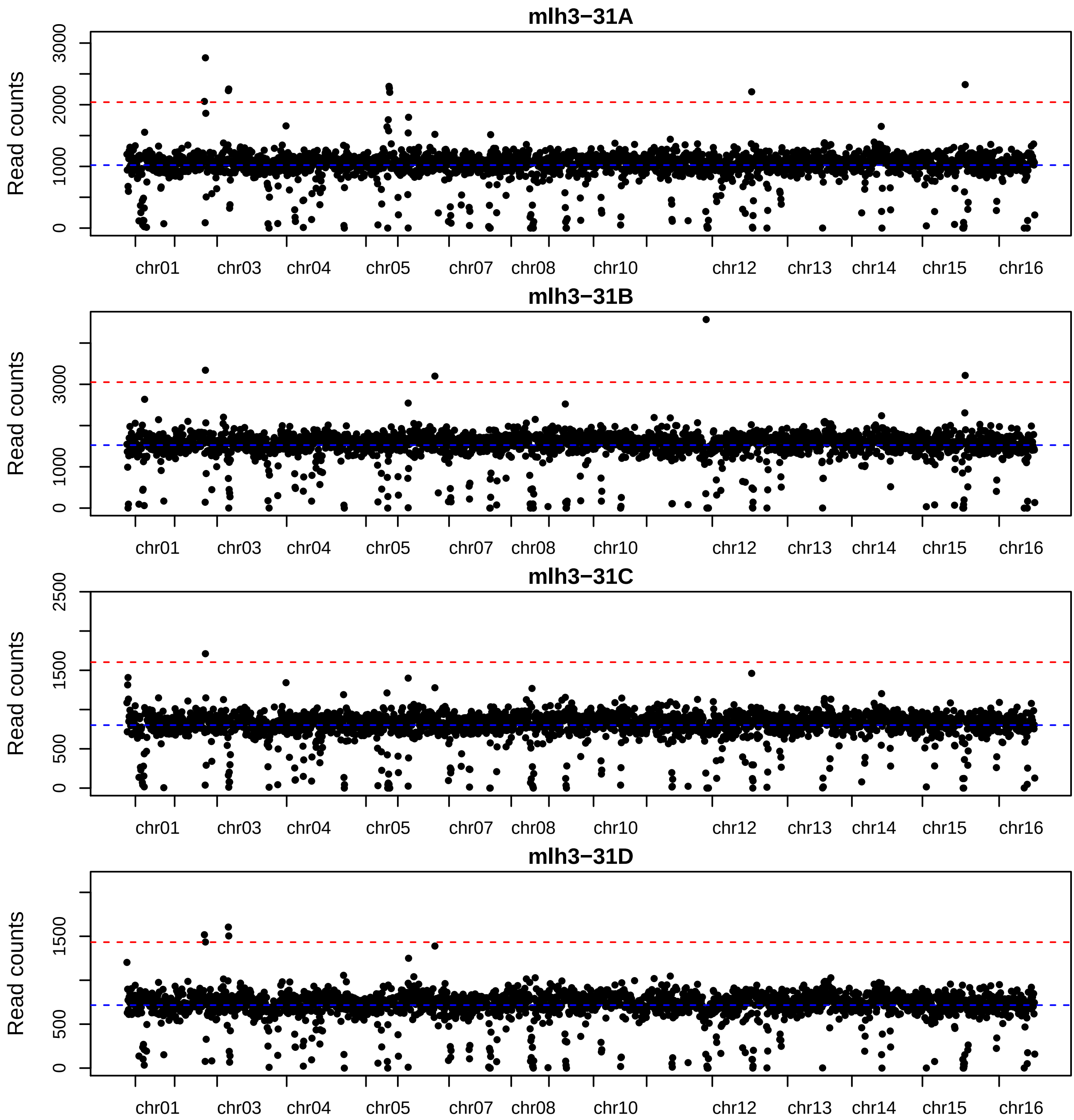
mlh3−31A
mlh3−31B
mlh3−31C
mlh3−31D
Read counts
chr01
chr03
chr04
chr05
chr07
chr08
chr10
chr12
chr13
chr14
chr15
chr16

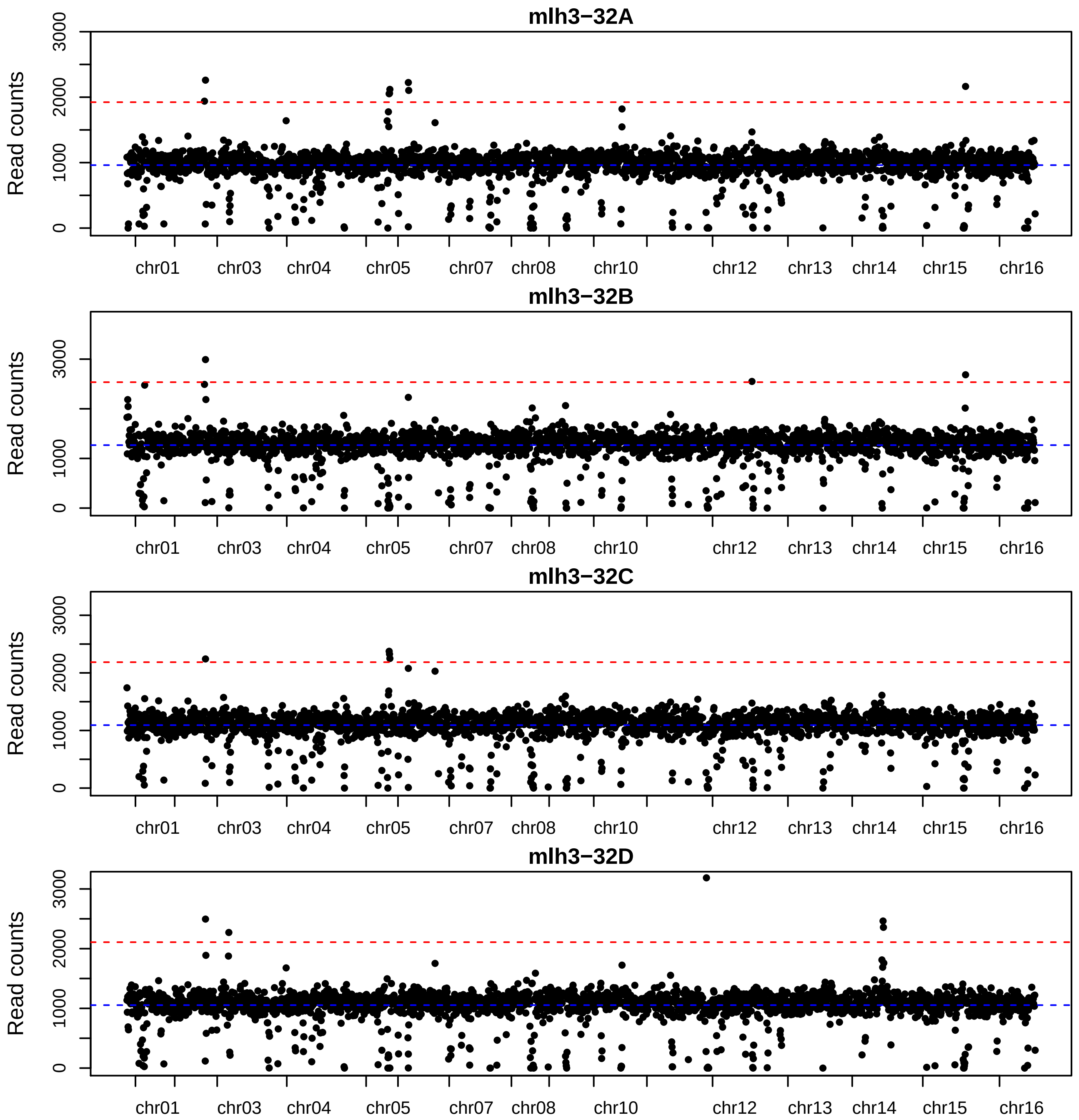
mlh3−32A
mlh3−32B
mlh3−32C
mlh3−32D
Read counts
0
1000
2000
3000
chr01
chr03
chr04
chr05
chr07
chr08
chr10
chr12
chr13
chr14
chr15
chr16

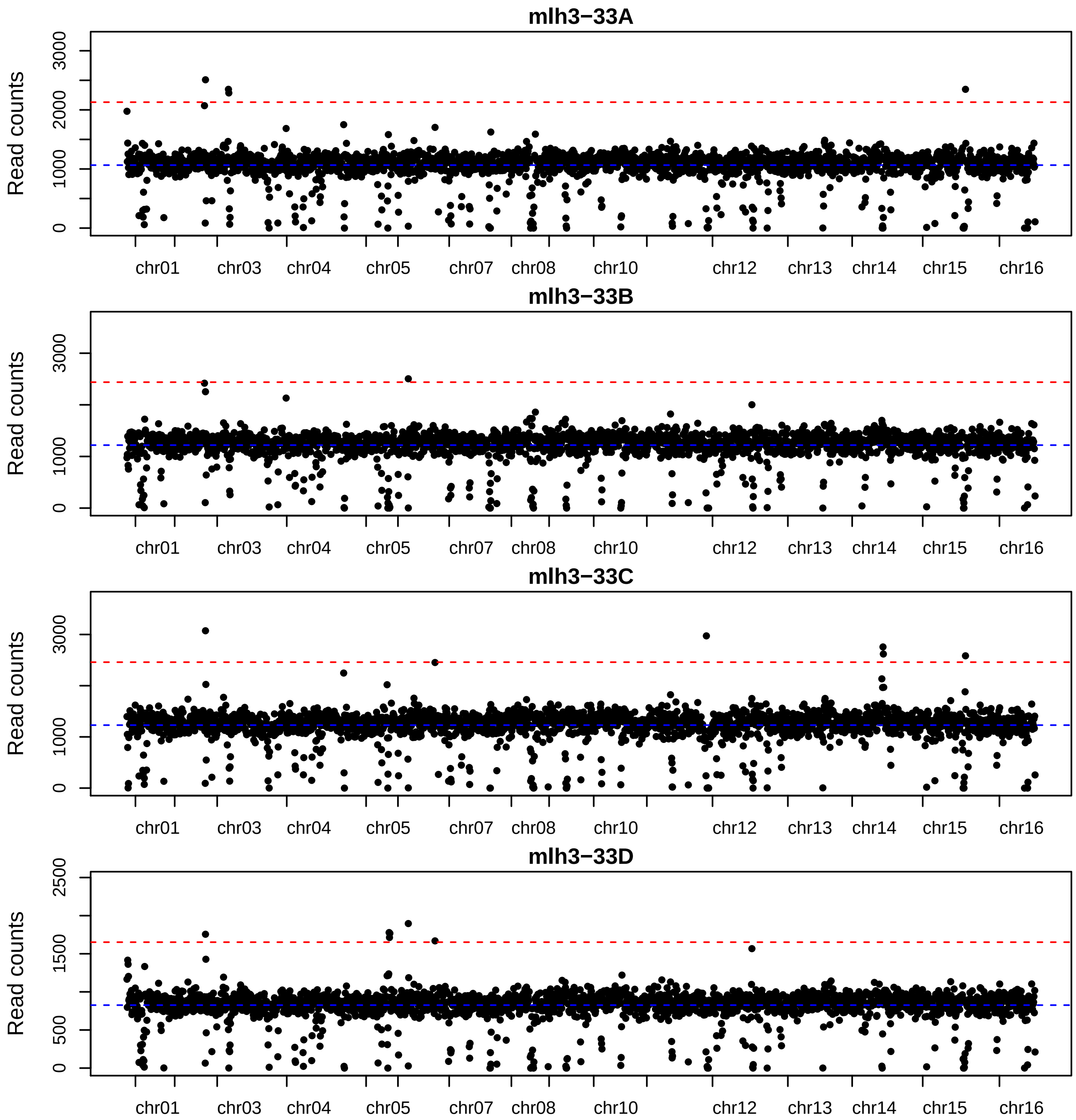
mlh3−33A
mlh3−33B
mlh3−33C
mlh3−33D
Read counts
0
500
1000
1500
2000
2500
3000
chr01
chr03
chr04
chr05
chr07
chr08
chr10
chr12
chr13
chr14
chr15
chr16

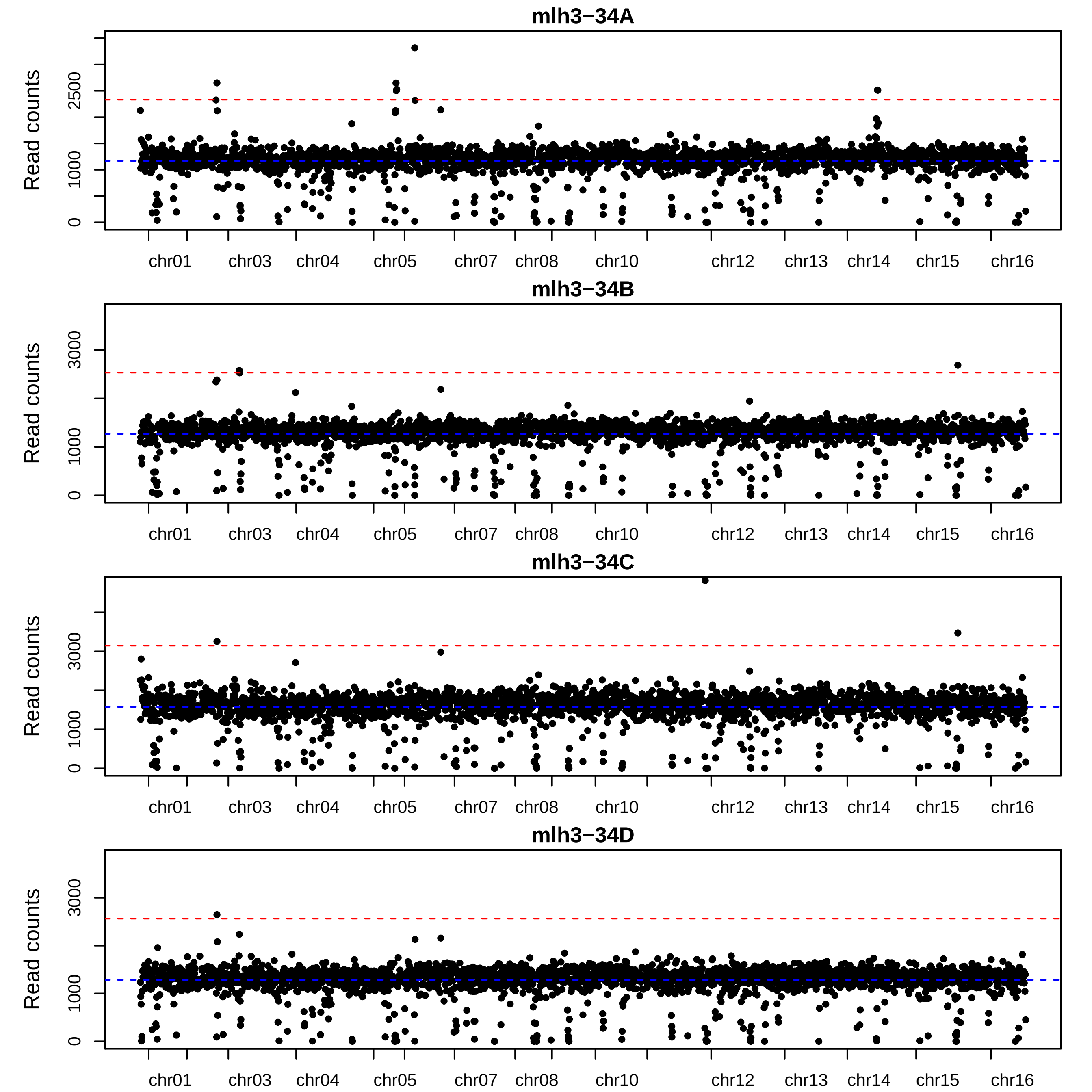
mlh3−34A
mlh3−34B
mlh3−34C
mlh3−34D
Read counts
0
1000
2500
3000
chr01
chr03
chr04
chr05
chr07
chr08
chr10
chr12
chr13
chr14
chr15
chr16

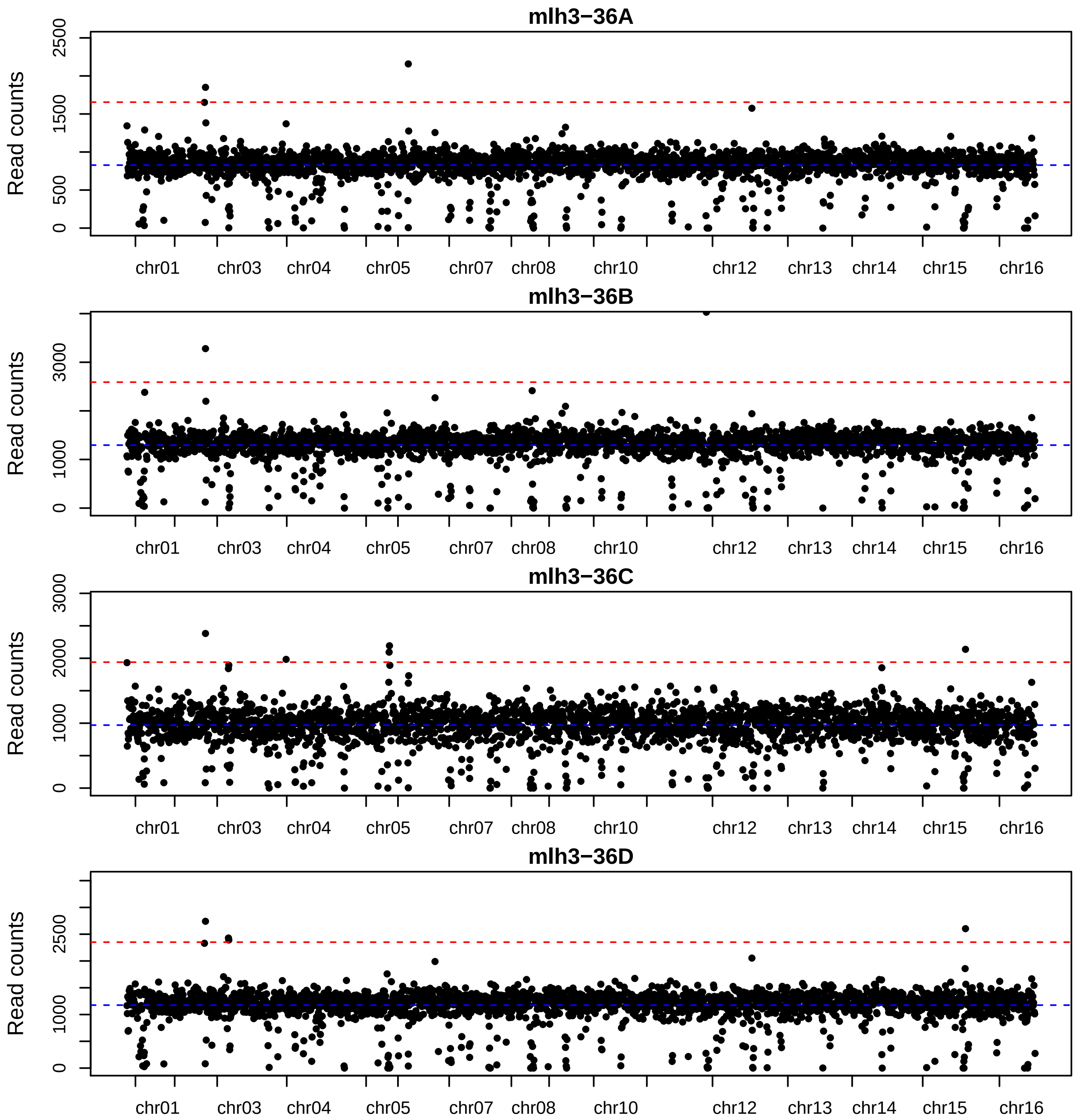
mlh3−36A
Read counts
0
500
1500
2500
chr01
chr03
chr04
chr05
chr07
chr08
chr10
chr12
chr13
chr14
chr15
chr16
mlh3−36B
Read counts
0
1000
3000
chr01
chr03
chr04
chr05
chr07
chr08
chr10
chr12
chr13
chr14
chr15
chr16
mlh3−36C
Read counts
0
1000
2000
3000
chr01
chr03
chr04
chr05
chr07
chr08
chr10
chr12
chr13
chr14
chr15
chr16
mlh3−36D
Read counts
0
1000
2500
chr01
chr03
chr04
chr05
chr07
chr08
chr10
chr12
chr13
chr14
chr15
chr16

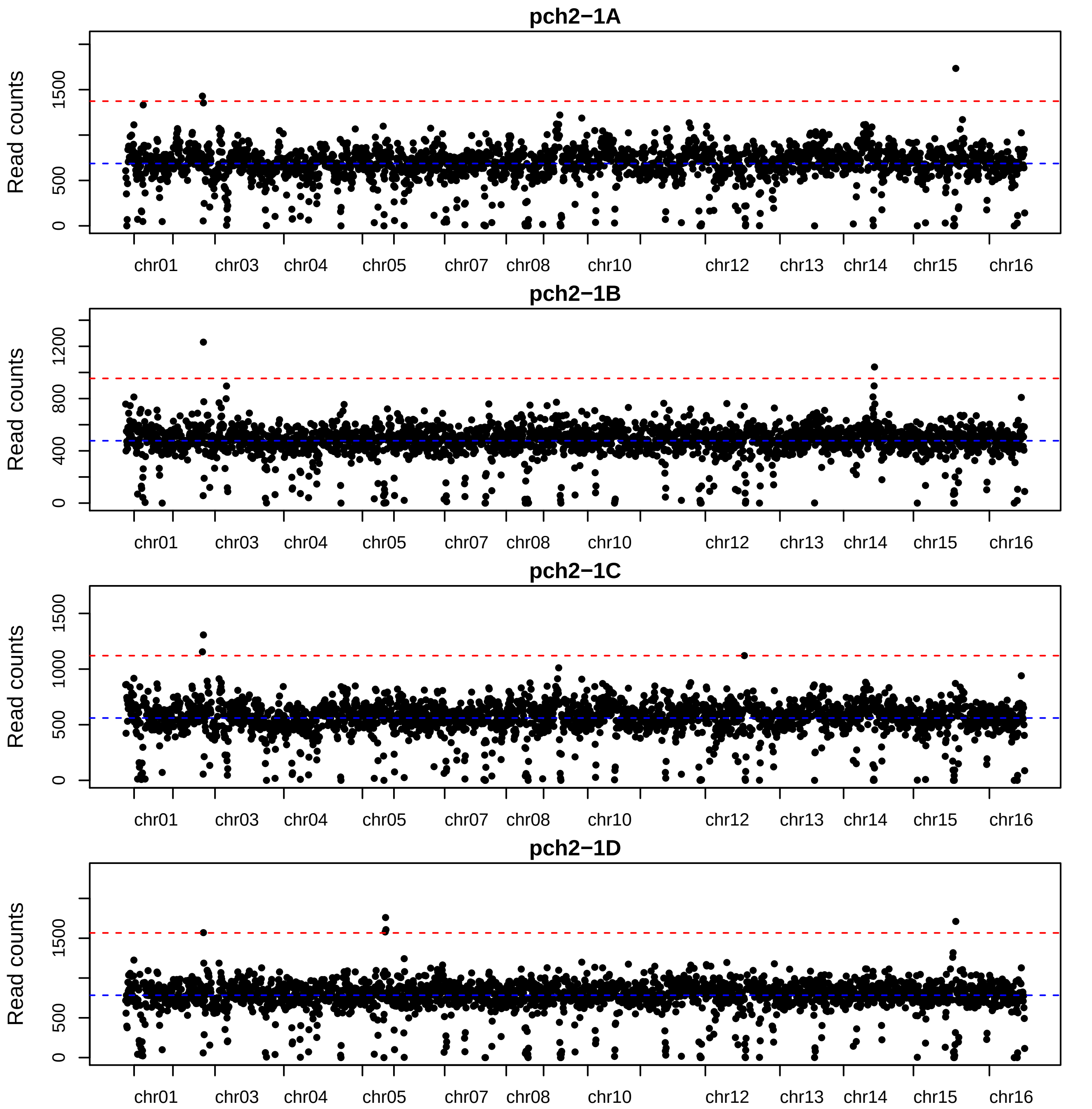

pch2−1A
pch2−1B
pch2−1C
pch2−1D
Read counts
0
500
1000
1500
400
800
1200
chr01
chr03
chr04
chr05
chr07
chr08
chr10
chr12
chr13
chr14
chr15
chr16

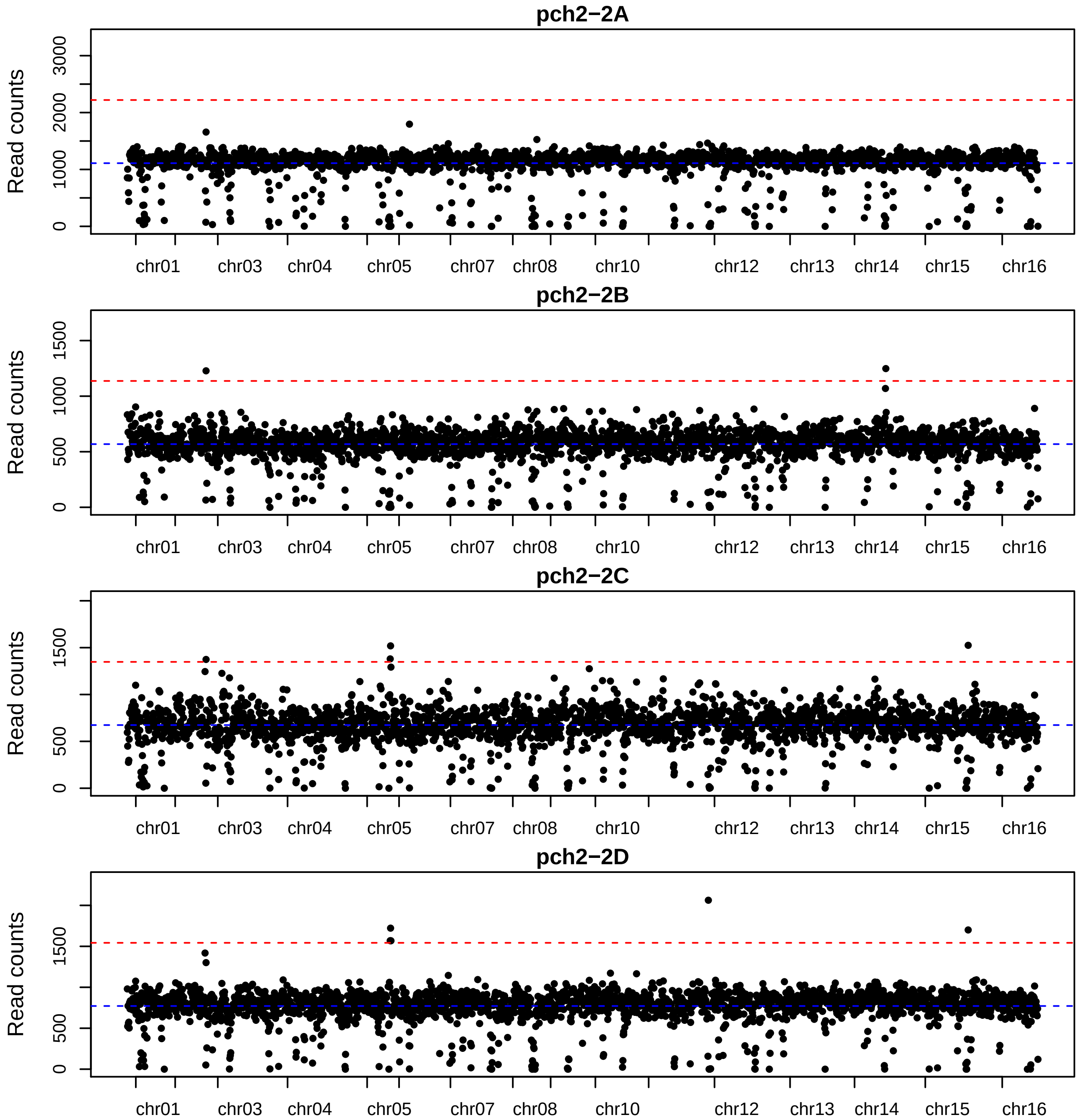
pch2−2A
pch2−2B
pch2−2C
pch2−2D
Read counts
0
1000
2000
3000
500
1500
chr01
chr03
chr04
chr05
chr07
chr08
chr10
chr12
chr13
chr14
chr15
chr16

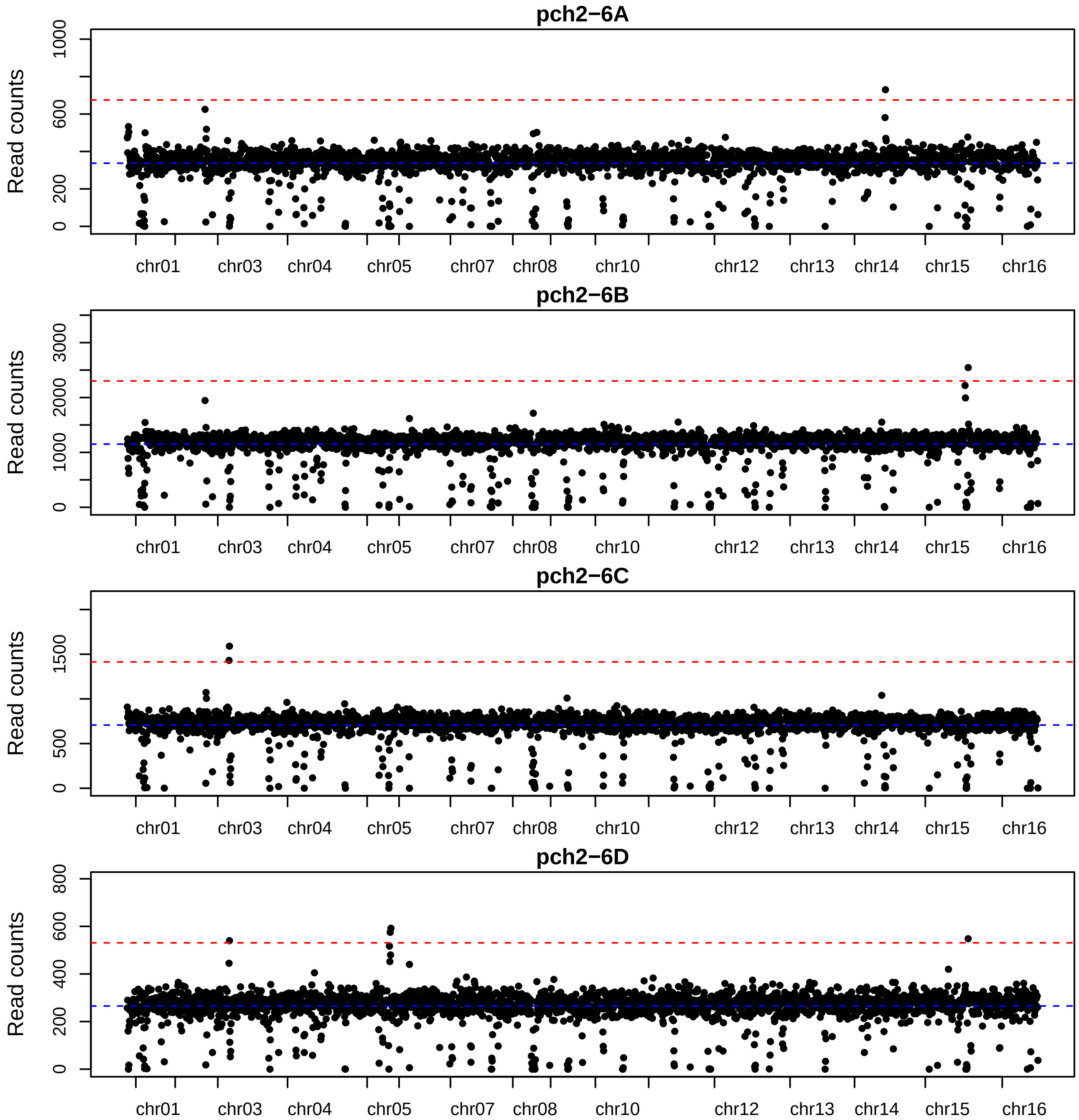
pch2−6A
pch2−6B
pch2−6C
pch2−6D
Read counts
chr01
chr03
chr04
chr05
chr07
chr08
chr10
chr12
chr13
chr14
chr15
chr16

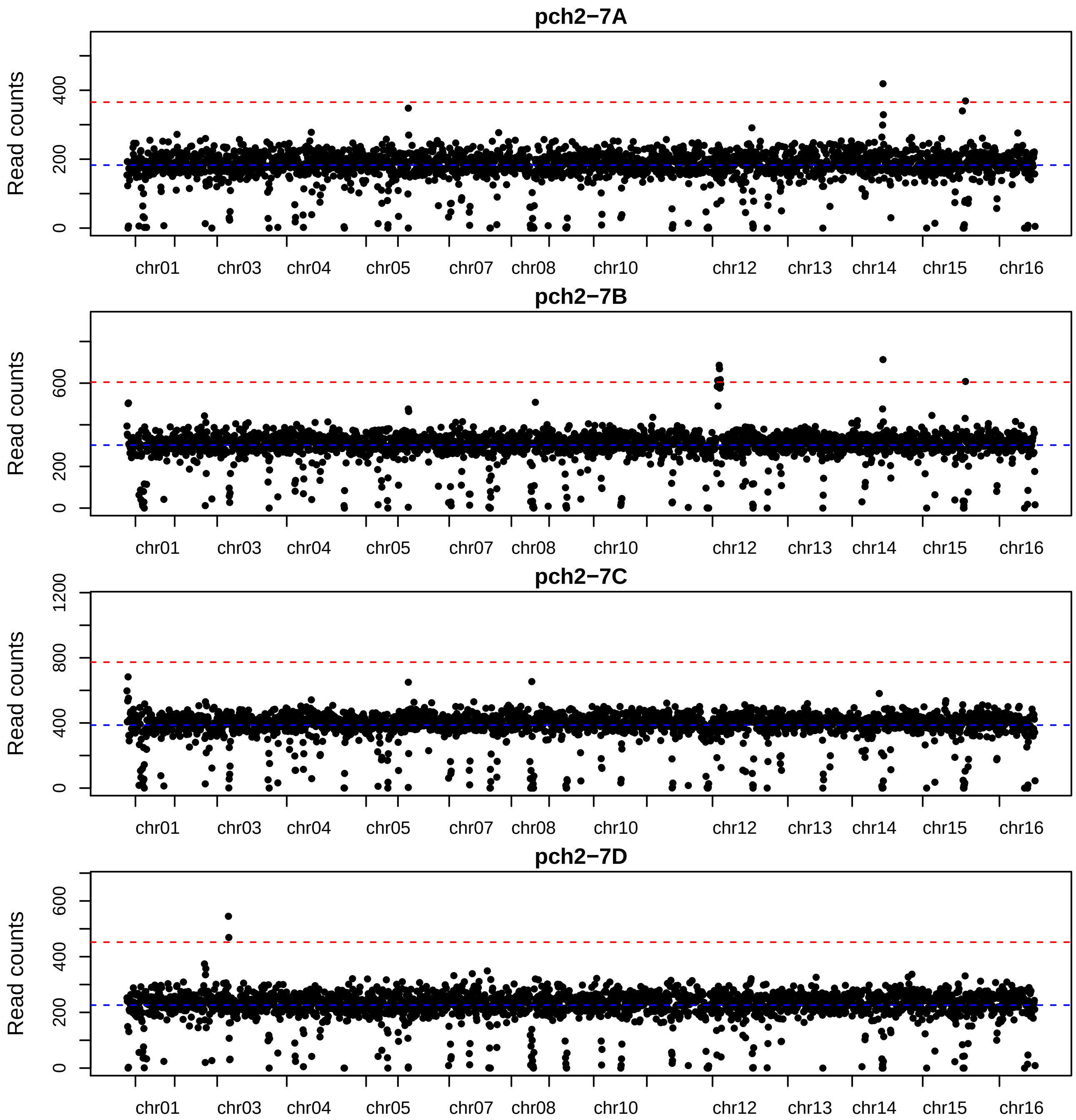
pch2−7A
Read counts
0
200
400
chr01
chr03
chr04
chr05
chr07
chr08
chr10
chr12
chr13
chr14
chr15
chr16
pch2−7B
Read counts
0
200
600
chr01
chr03
chr04
chr05
chr07
chr08
chr10
chr12
chr13
chr14
chr15
chr16
pch2−7C
Read counts
0
400
800
1200
chr01
chr03
chr04
chr05
chr07
chr08
chr10
chr12
chr13
chr14
chr15
chr16
pch2−7D
Read counts
0
200
400
600
chr01
chr03
chr04
chr05
chr07
chr08
chr10
chr12
chr13
chr14
chr15
chr16

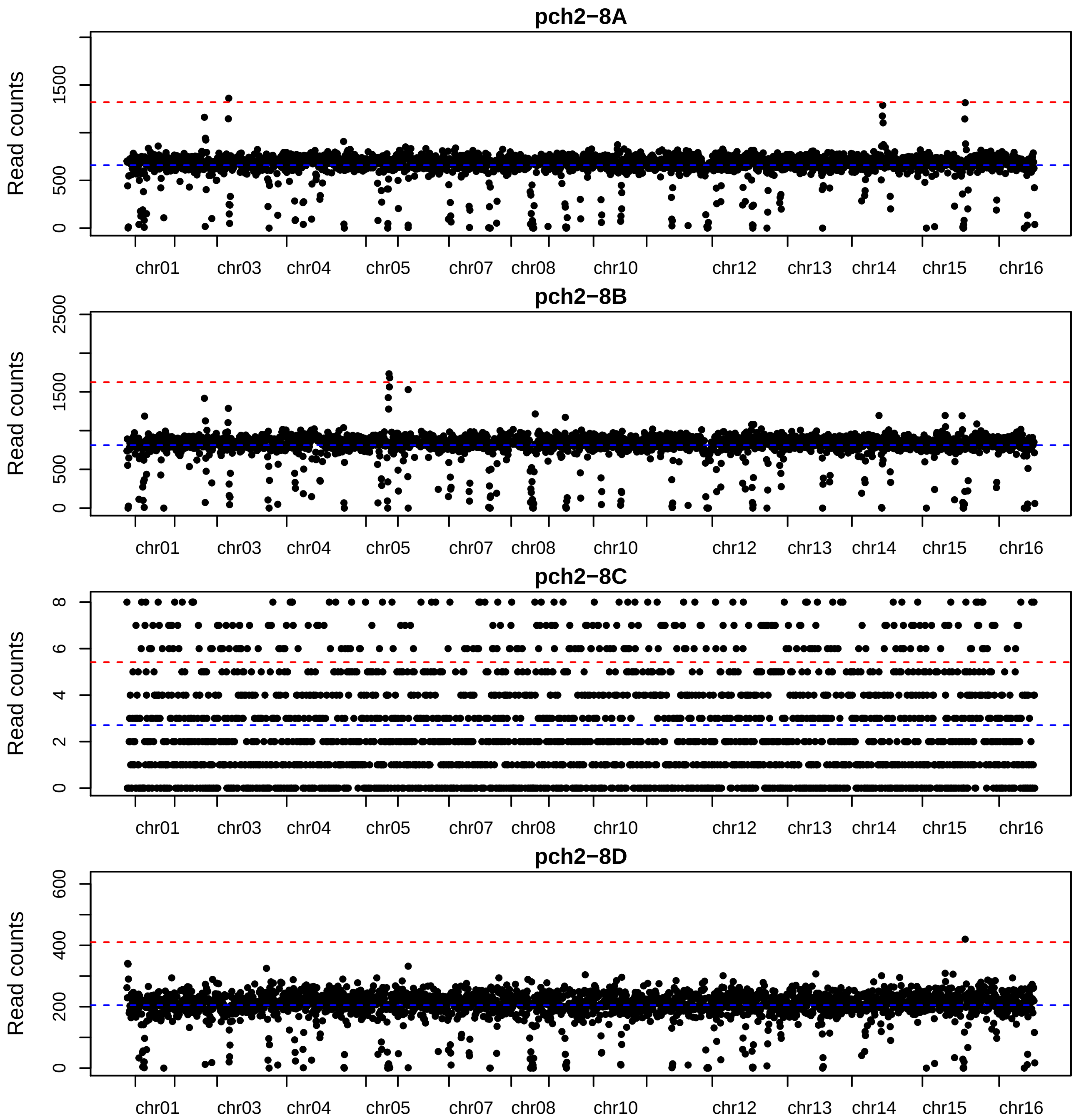
pch2−8A
Read counts
0
500
1500
pch2−8B
Read counts
0
500
1500
2500
pch2−8C
Read counts
0
2
4
6
8
pch2−8D
Read counts
0
200
400
600
chr01
chr03
chr04
chr05
chr07
chr08
chr10
chr12
chr13
chr14
chr15
chr16

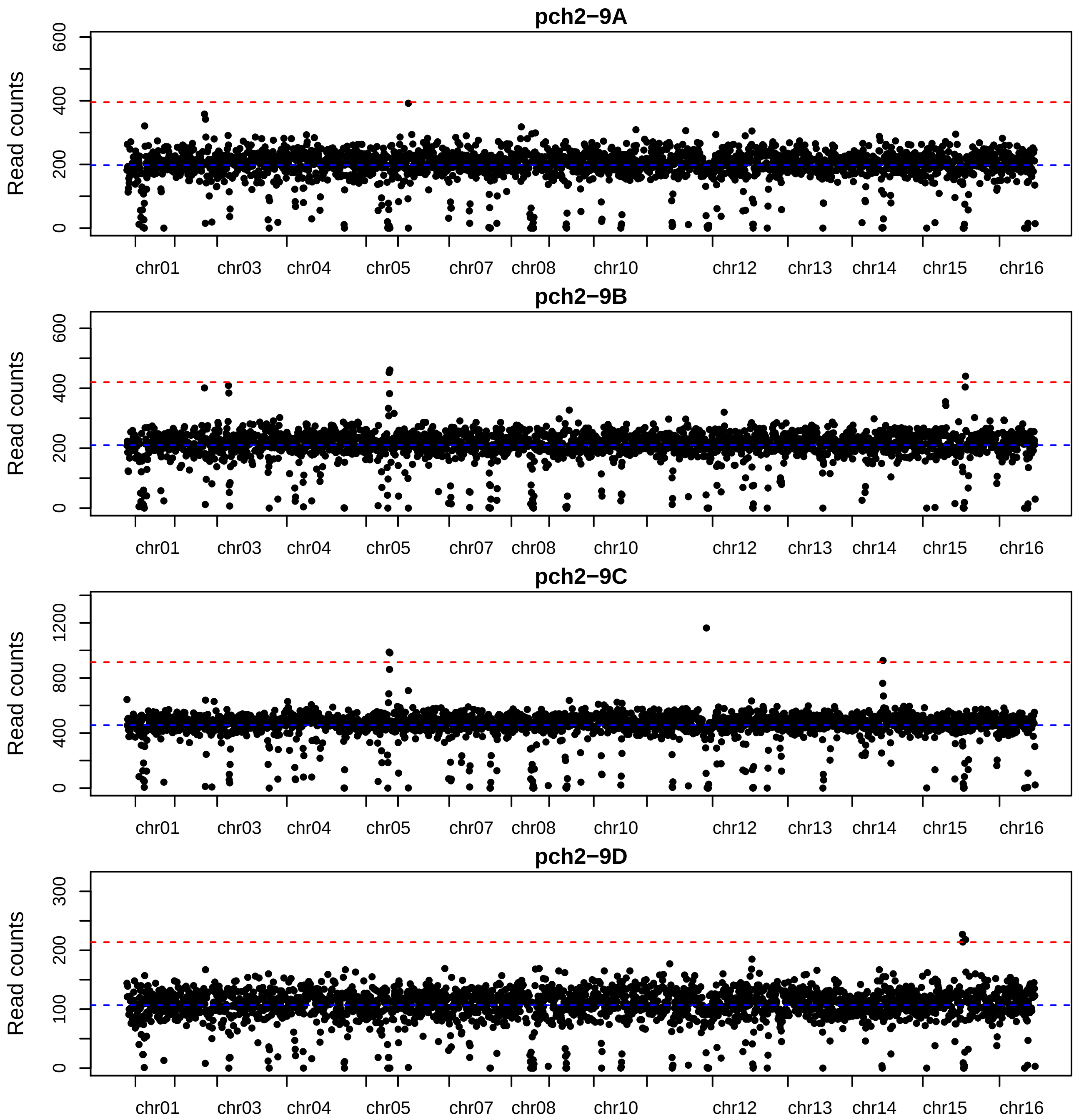
pch2−9A
Read counts
0
200
400
600
chr01
chr03
chr04
chr05
chr07
chr08
chr10
chr12
chr13
chr14
chr15
chr16
pch2−9B
Read counts
0
200
400
600
chr01
chr03
chr04
chr05
chr07
chr08
chr10
chr12
chr13
chr14
chr15
chr16
pch2−9C
Read counts
0
400
800
1200
chr01
chr03
chr04
chr05
chr07
chr08
chr10
chr12
chr13
chr14
chr15
chr16
pch2−9D
Read counts
0
100
200
300
chr01
chr03
chr04
chr05
chr07
chr08
chr10
chr12
chr13
chr14
chr15
chr16

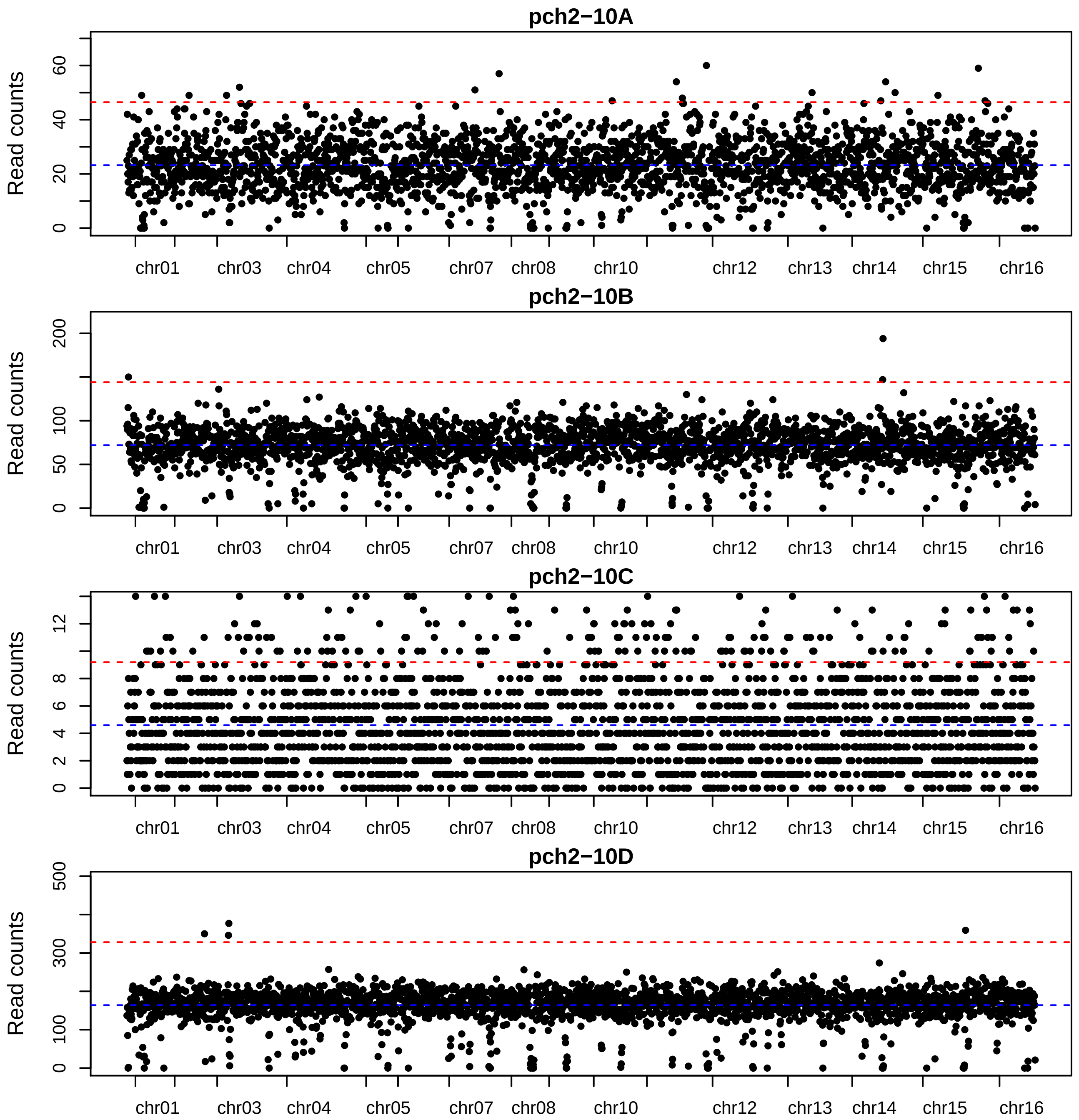
pch2−10A
Read counts
0
20
40
60
chr01
chr03
chr04
chr05
chr07
chr08
chr10
chr12
chr13
chr14
chr15
chr16
pch2−10B
Read counts
0
50
100
200
pch2−10C
Read counts
0
2
4
6
8
12
pch2−10D
Read counts
0
100
300
500

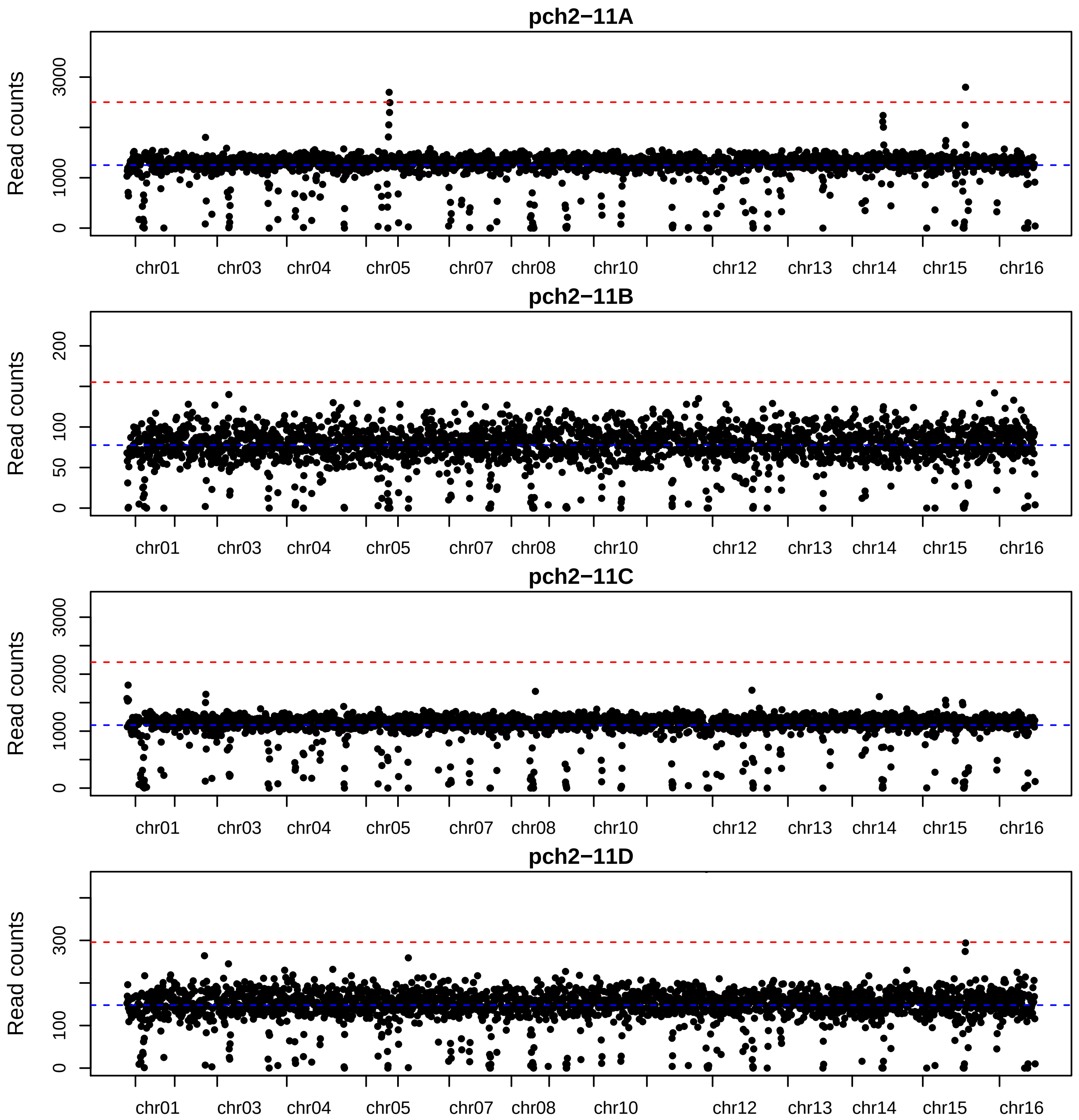

pch2−11A
Read counts
0
1000
3000
chr01
chr03
chr04
chr05
chr07
chr08
chr10
chr12
chr13
chr14
chr15
chr16
pch2−11B
Read counts
0
50
100
200
chr01
chr03
chr04
chr05
chr07
chr08
chr10
chr12
chr13
chr14
chr15
chr16
pch2−11C
Read counts
0
1000
2000
3000
chr01
chr03
chr04
chr05
chr07
chr08
chr10
chr12
chr13
chr14
chr15
chr16
pch2−11D
Read counts
0
100
300
chr01
chr03
chr04
chr05
chr07
chr08
chr10
chr12
chr13
chr14
chr15
chr16

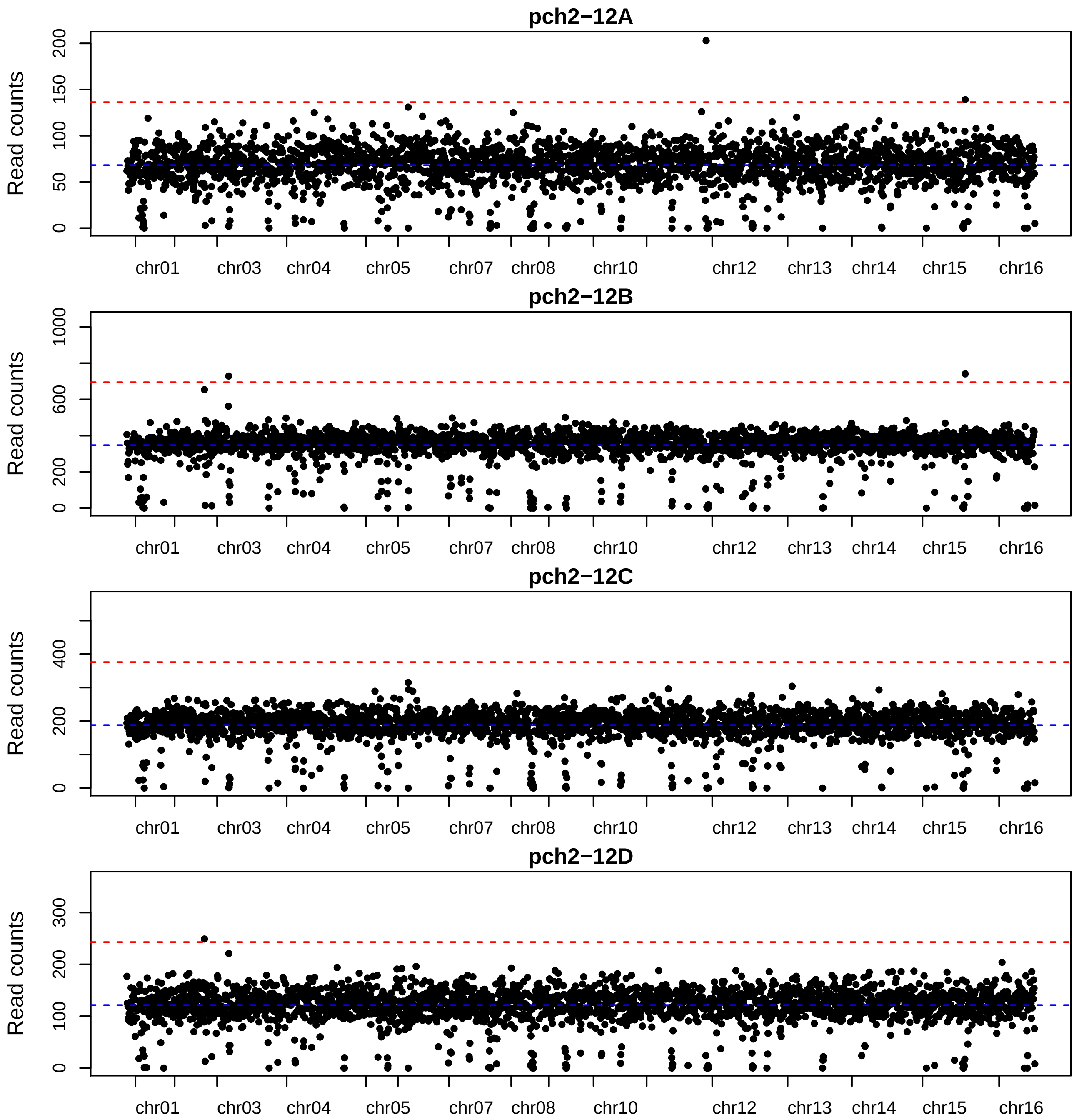
pch2−12A
pch2−12B
pch2−12C
pch2−12D
Read counts
0
50
100
150
200
0
200
600
1000
0
200
400
0
100
200
300
chr01
chr03
chr04
chr05
chr07
chr08
chr10
chr12
chr13
chr14
chr15
chr16

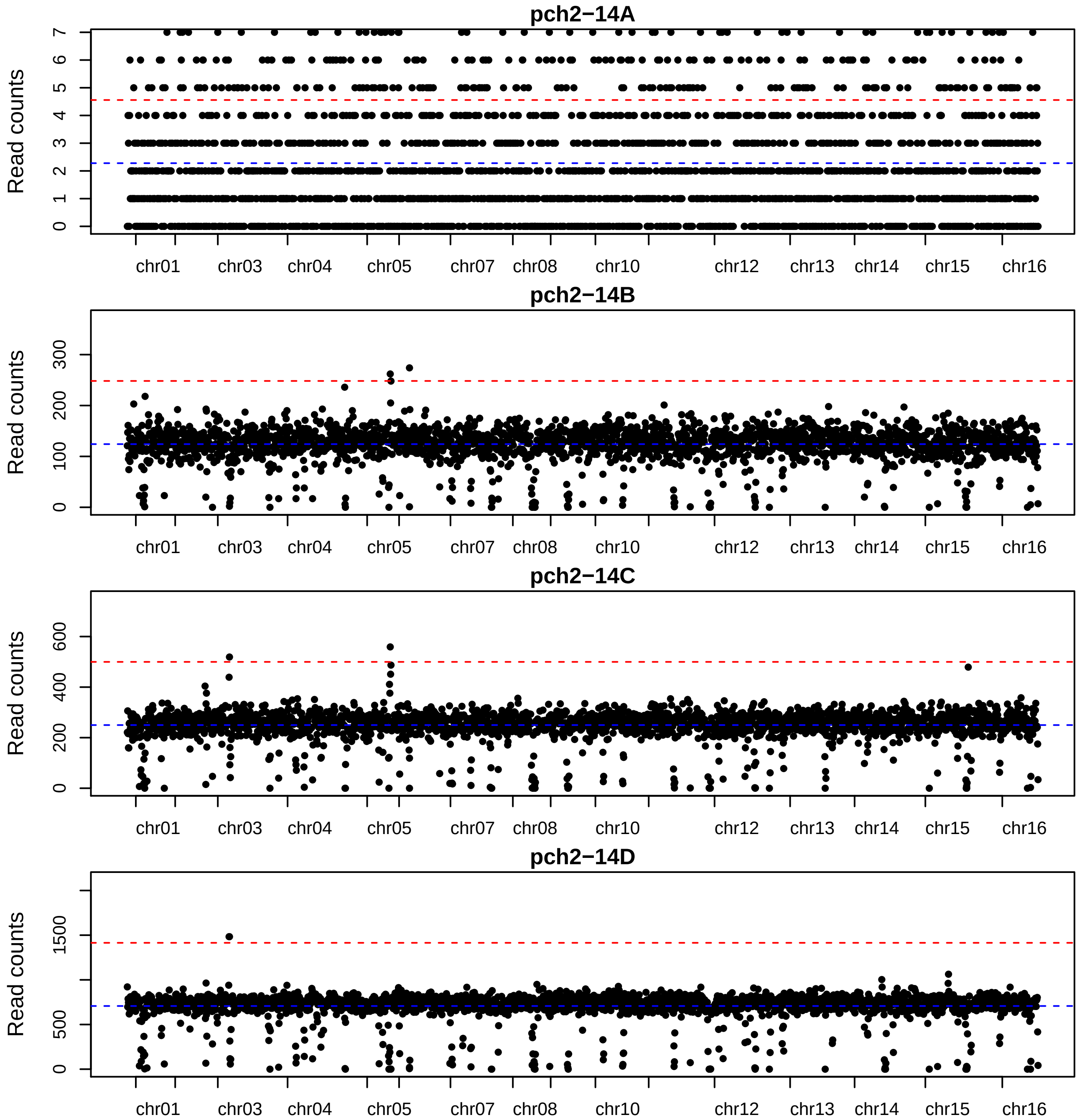
pch2−14A
pch2−14B
pch2−14C
pch2−14D
Read counts
chr01
chr03
chr04
chr05
chr07
chr08
chr10
chr12
chr13
chr14
chr15
chr16
0
1
2
3
4
5
6
7
100
200
300
400
600
500
1500

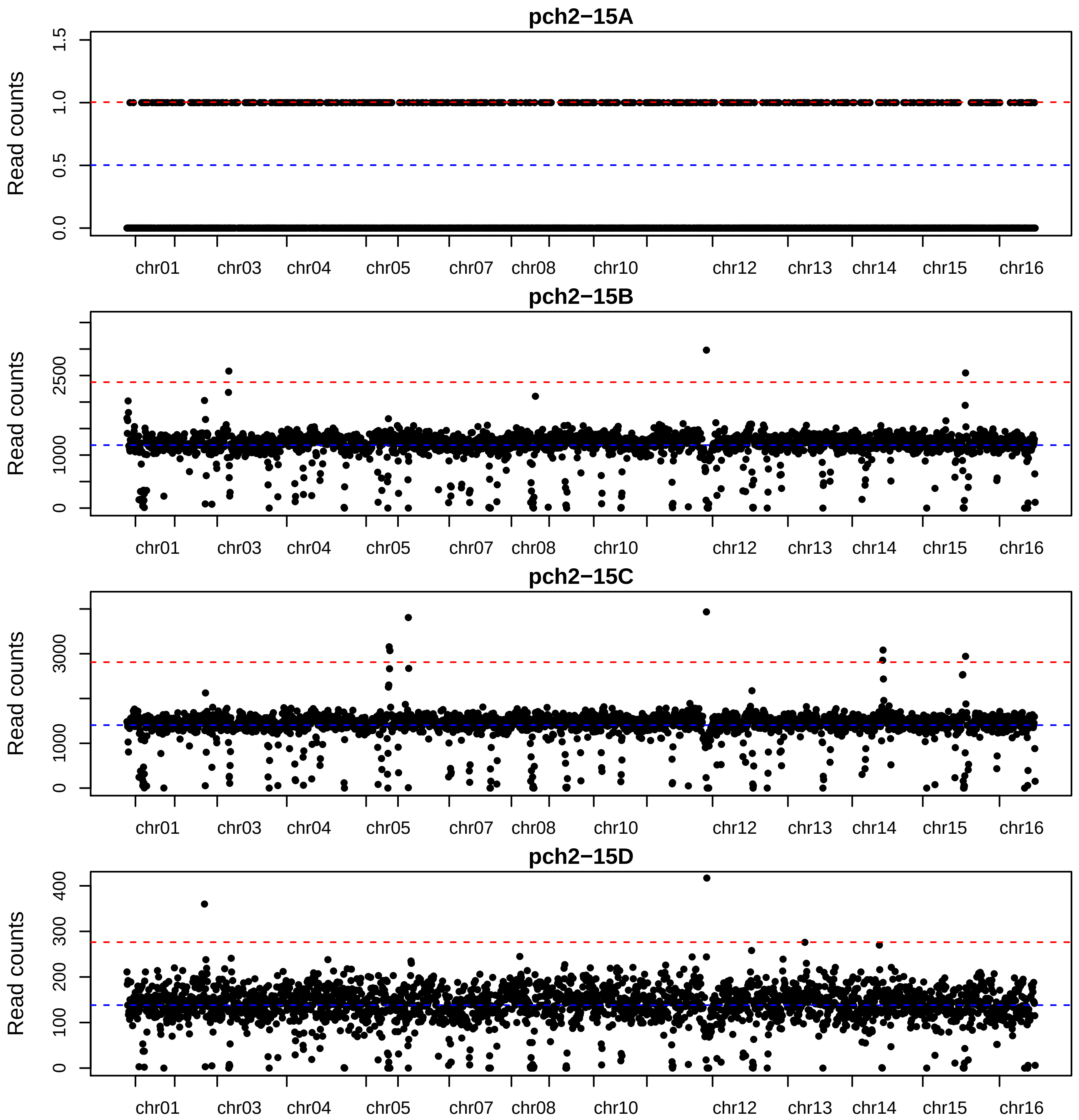

pch2−15A
Read counts
0.0
0.5
1.0
1.5
chr01
chr03
chr04
chr05
chr07
chr08
chr10
chr12
chr13
chr14
chr15
chr16
pch2−15B
Read counts
0
1000
2500
chr01
chr03
chr04
chr05
chr07
chr08
chr10
chr12
chr13
chr14
chr15
chr16
pch2−15C
Read counts
0
1000
3000
chr01
chr03
chr04
chr05
chr07
chr08
chr10
chr12
chr13
chr14
chr15
chr16
pch2−15D
Read counts
0
100
200
300
400
chr01
chr03
chr04
chr05
chr07
chr08
chr10
chr12
chr13
chr14
chr15
chr16

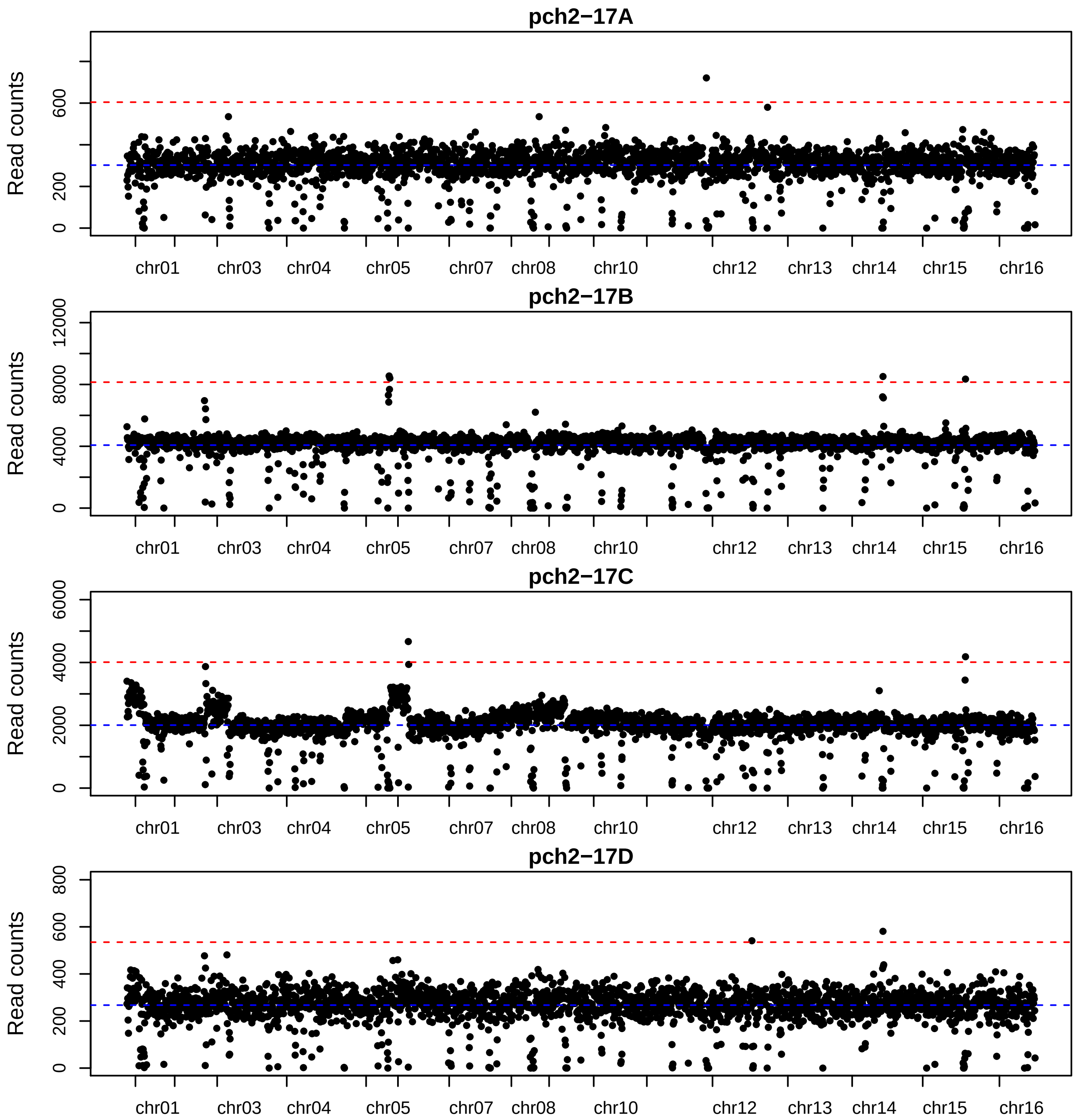
pch2−17A
pch2−17B
pch2−17C
pch2−17D
Read counts
0
200
600
0
4000
8000
12000
0
2000
4000
6000
0
200
400
600
800
chr01
chr03
chr04
chr05
chr07
chr08
chr10
chr12
chr13
chr14
chr15
chr16

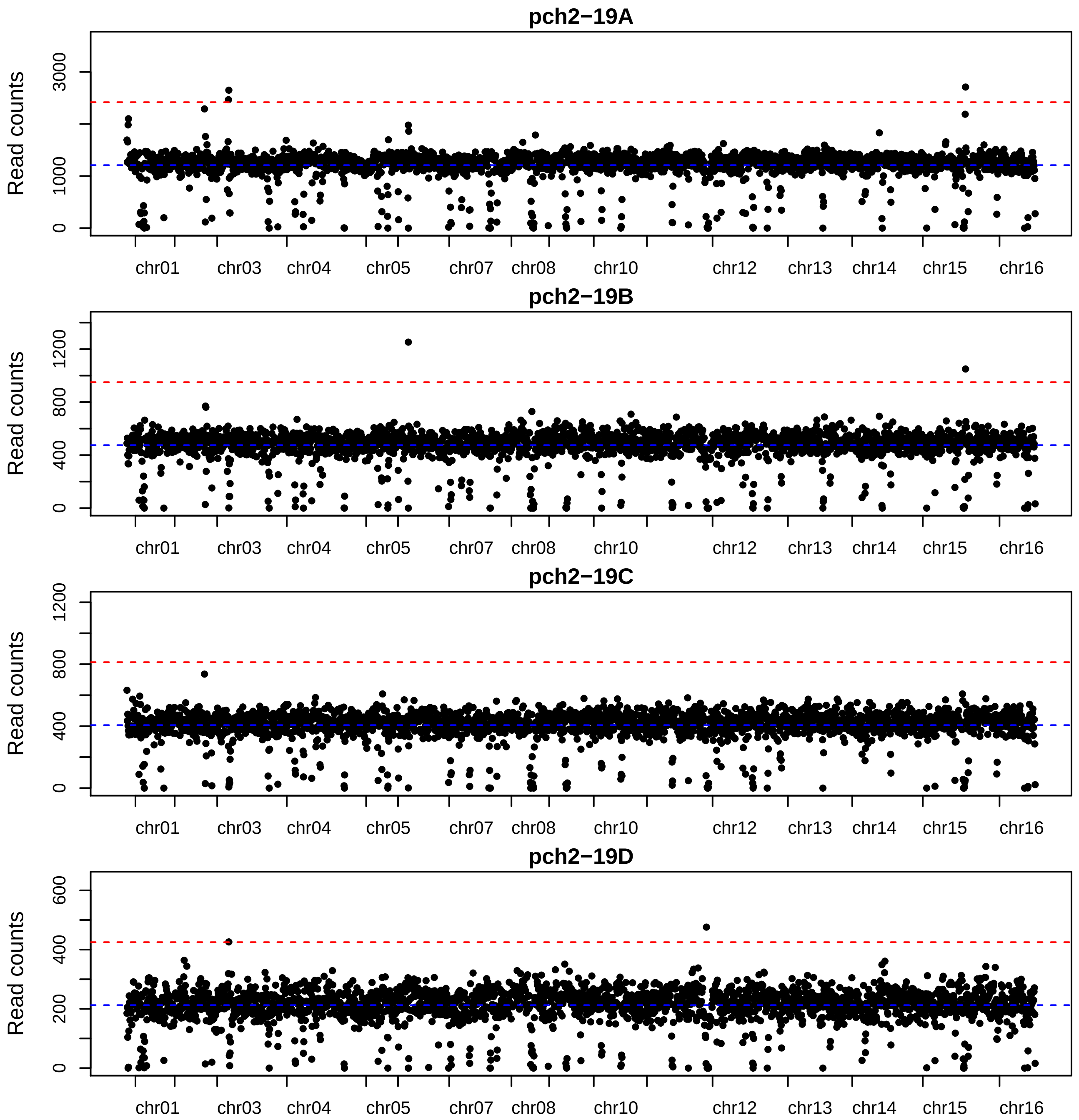
pch2−19A
Read counts
0
1000
3000
chr01
chr03
chr04
chr05
chr07
chr08
chr10
chr12
chr13
chr14
chr15
chr16
pch2−19B
Read counts
0
400
800
1200
chr01
chr03
chr04
chr05
chr07
chr08
chr10
chr12
chr13
chr14
chr15
chr16
pch2−19C
Read counts
0
400
800
1200
chr01
chr03
chr04
chr05
chr07
chr08
chr10
chr12
chr13
chr14
chr15
chr16
pch2−19D
Read counts
0
200
400
600
chr01
chr03
chr04
chr05
chr07
chr08
chr10
chr12
chr13
chr14
chr15
chr16

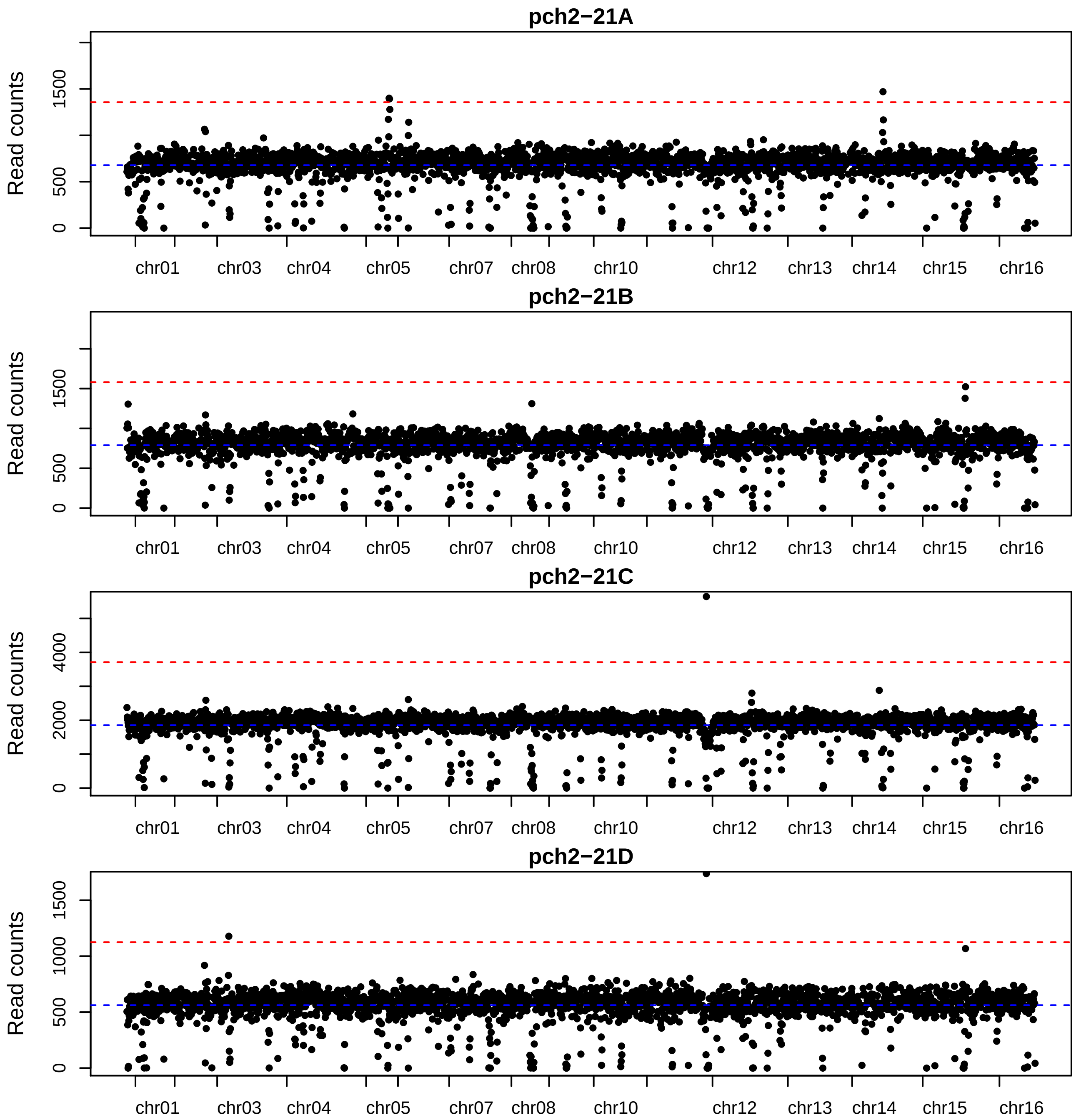

pch2−21A
pch2−21B
pch2−21C
pch2−21D
Read counts
0
500
1500
1000
2000
4000
chr01
chr03
chr04
chr05
chr07
chr08
chr10
chr12
chr13
chr14
chr15
chr16

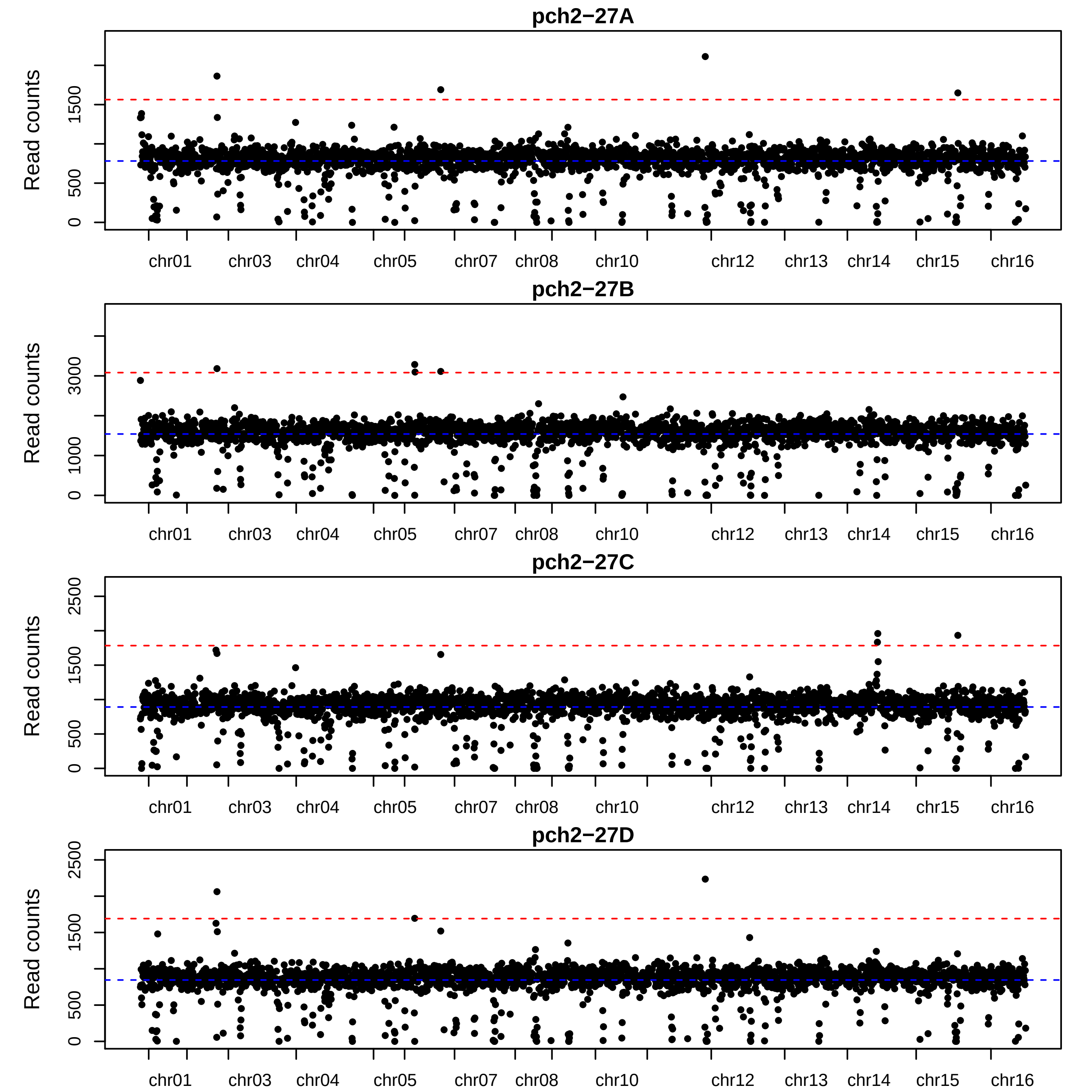
pch2−27A
Read counts
0
500
1500
chr01
chr03
chr04
chr05
chr07
chr08
chr10
chr12
chr13
chr14
chr15
chr16
pch2−27B
Read counts
0
1000
3000
pch2−27C
Read counts
0
500
1500
2500
pch2−27D
Read counts
0
500
1500
2500

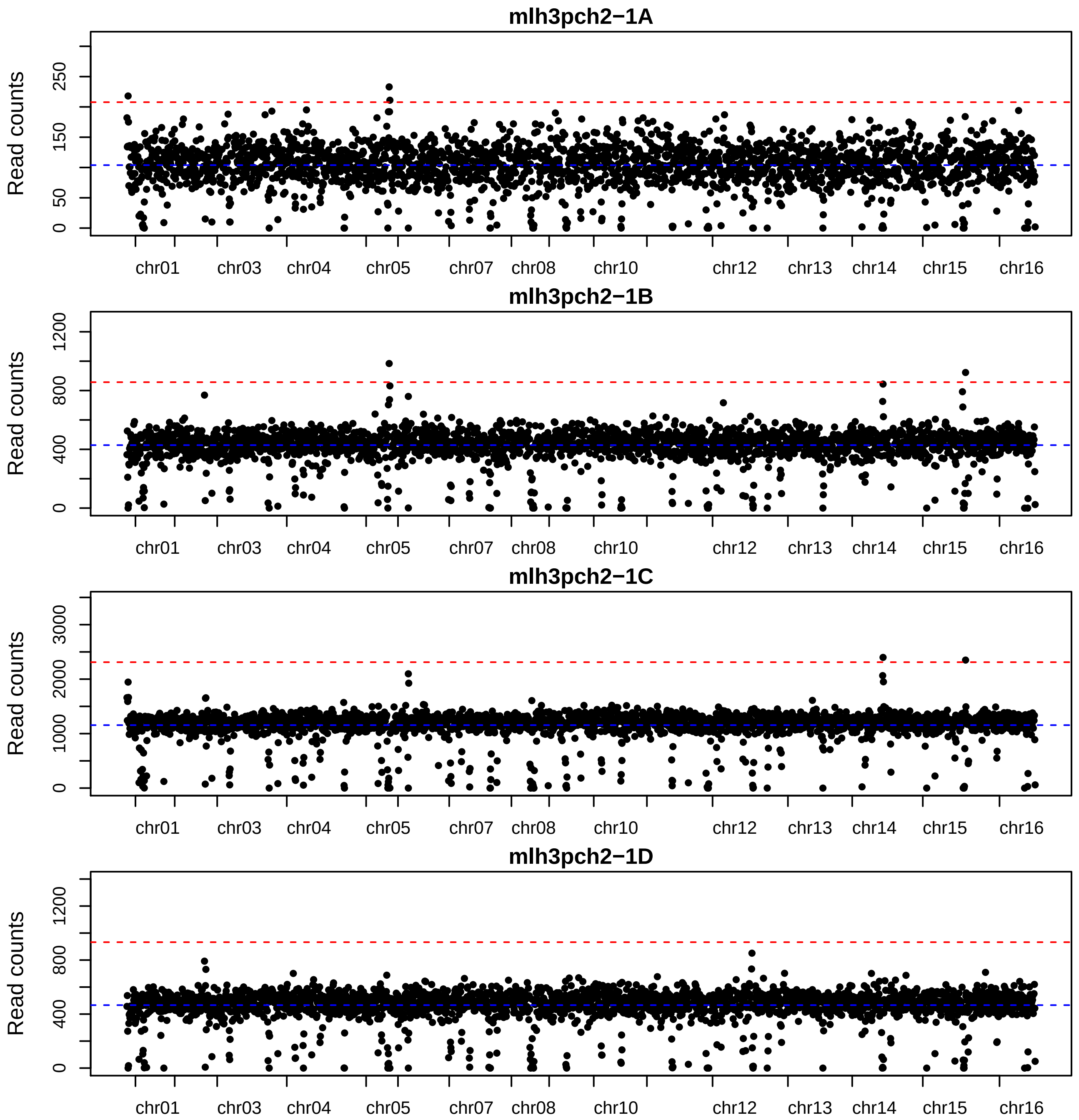
mlh3pch2−1A
Read counts
0
50
150
250
chr01
chr03
chr04
chr05
chr07
chr08
chr10
chr12
chr13
chr14
chr15
chr16
mlh3pch2−1B
Read counts
0
400
800
1200
chr01
chr03
chr04
chr05
chr07
chr08
chr10
chr12
chr13
chr14
chr15
chr16
mlh3pch2−1C
Read counts
0
1000
2000
3000
chr01
chr03
chr04
chr05
chr07
chr08
chr10
chr12
chr13
chr14
chr15
chr16
mlh3pch2−1D
Read counts
0
400
800
1200
chr01
chr03
chr04
chr05
chr07
chr08
chr10
chr12
chr13
chr14
chr15
chr16

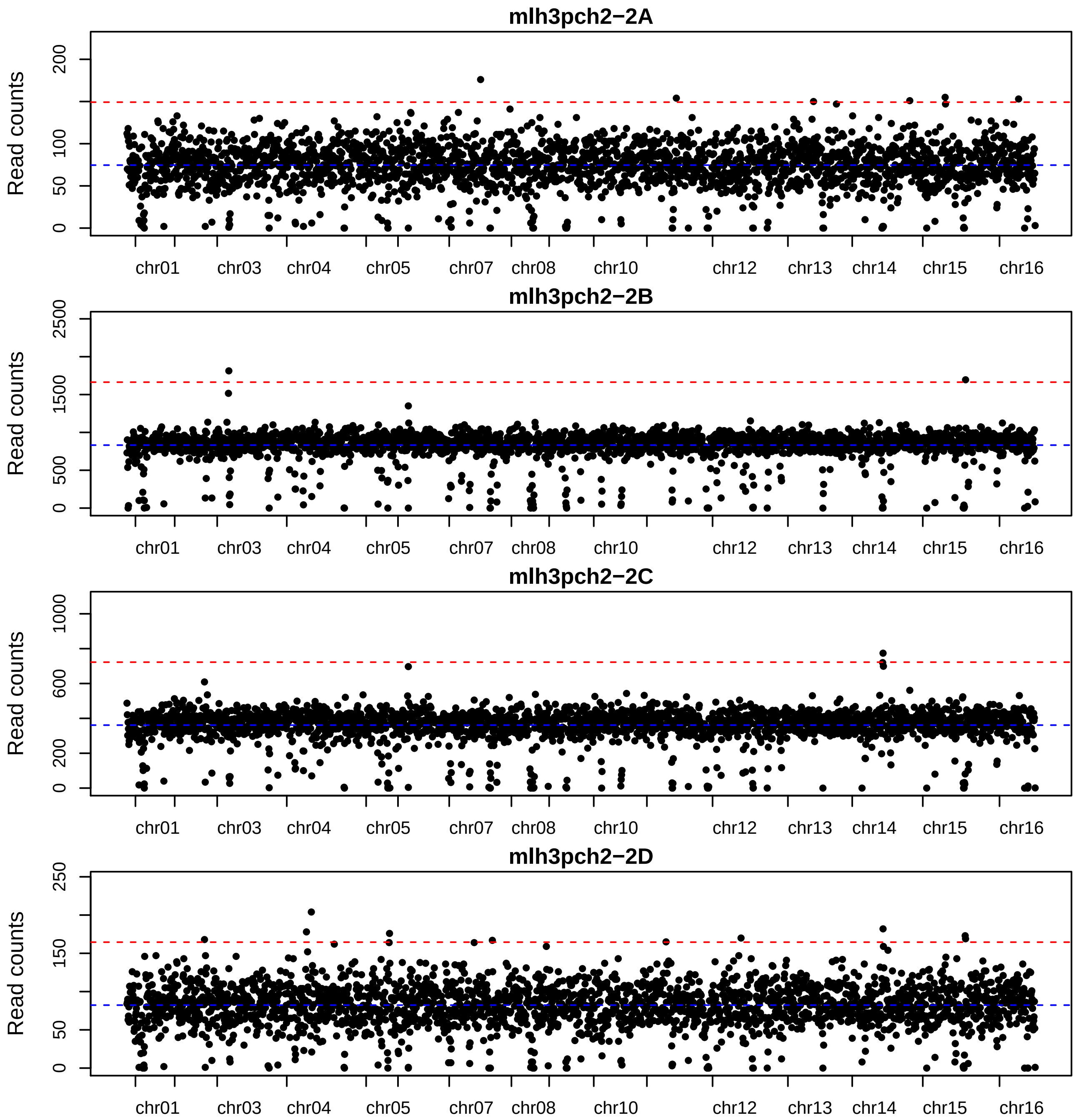

mlh3pch2−2A
mlh3pch2−2B
mlh3pch2−2C
mlh3pch2−2D
Read counts
chr01
chr03
chr04
chr05
chr07
chr08
chr10
chr12
chr13
chr14
chr15
chr16

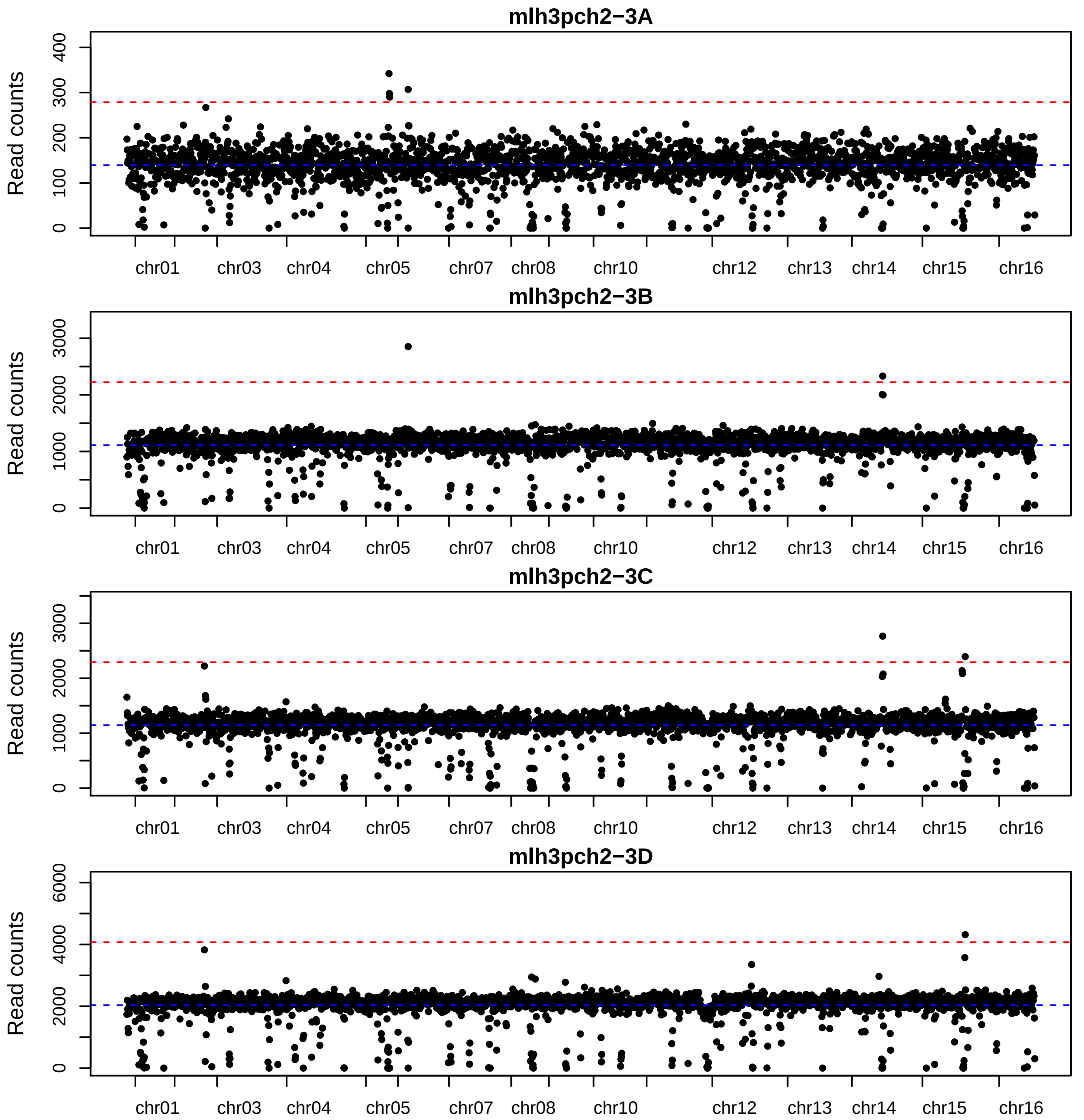
mlh3pch2−3A
mlh3pch2−3B
mlh3pch2−3C
mlh3pch2−3D
Read counts
chr01
chr03
chr04
chr05
chr07
chr08
chr10
chr12
chr13
chr14
chr15
chr16

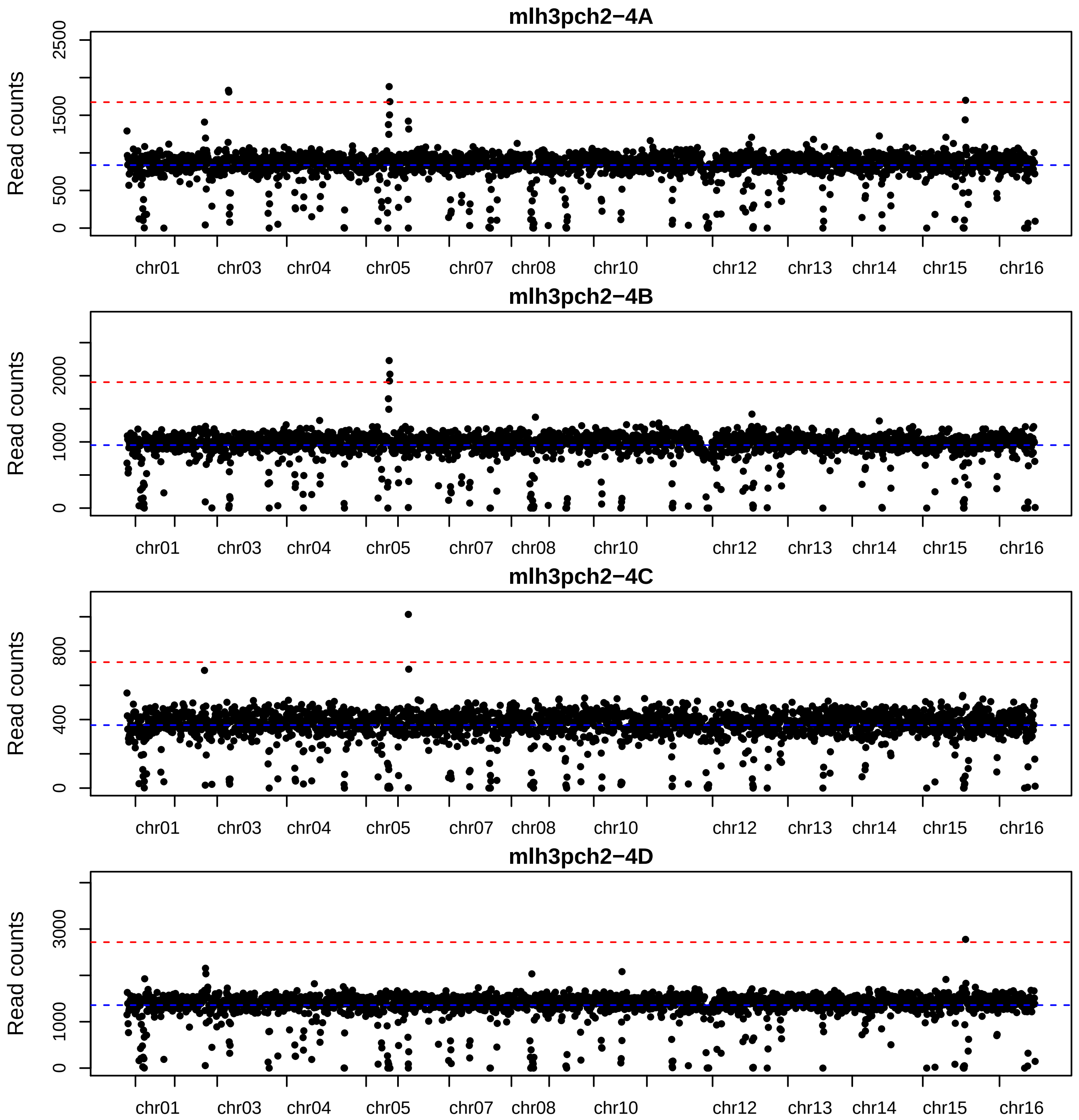
mlh3pch2−4A
Read counts
0
500
1500
2500
chr01
chr03
chr04
chr05
chr07
chr08
chr10
chr12
chr13
chr14
chr15
chr16
mlh3pch2−4B
Read counts
0
1000
2000
chr01
chr03
chr04
chr05
chr07
chr08
chr10
chr12
chr13
chr14
chr15
chr16
mlh3pch2−4C
Read counts
0
400
800
chr01
chr03
chr04
chr05
chr07
chr08
chr10
chr12
chr13
chr14
chr15
chr16
mlh3pch2−4D
Read counts
0
1000
3000
chr01
chr03
chr04
chr05
chr07
chr08
chr10
chr12
chr13
chr14
chr15
chr16

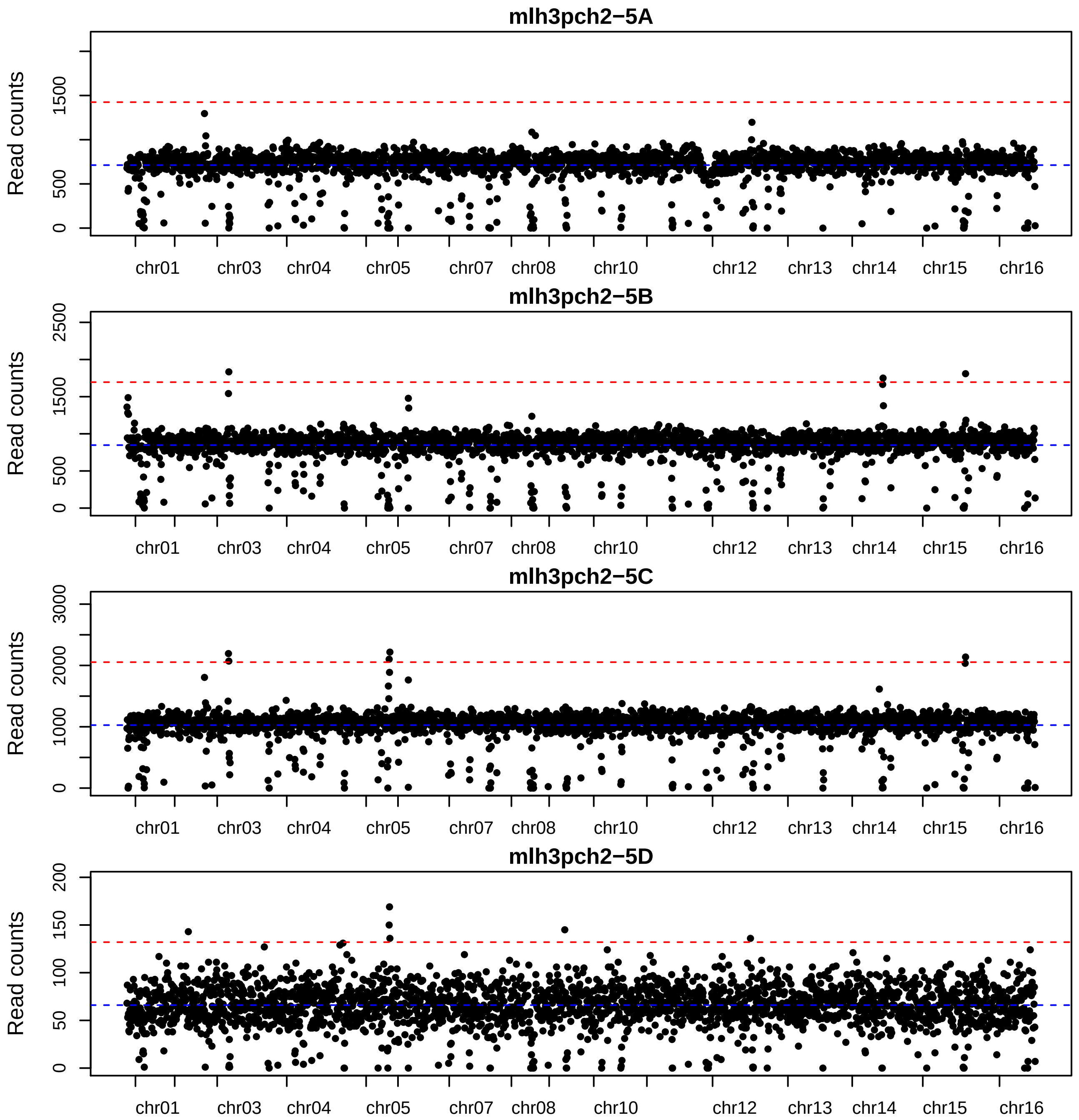
mlh3pch2−5A
mlh3pch2−5B
mlh3pch2−5C
mlh3pch2−5D
Read counts
chr01
chr03
chr04
chr05
chr07
chr08
chr10
chr12
chr13
chr14
chr15
chr16

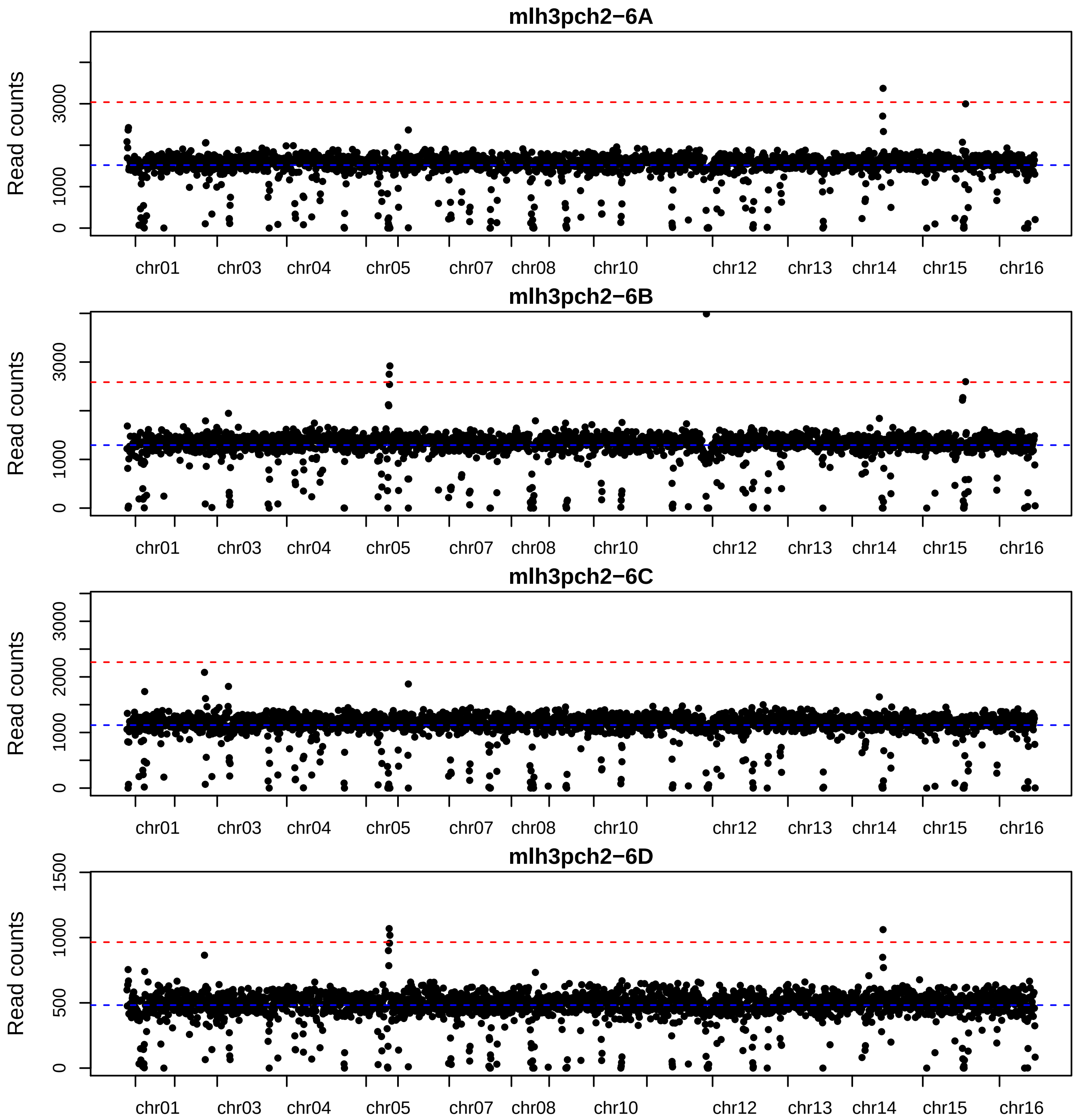

mlh3pch2−6A
mlh3pch2−6B
mlh3pch2−6C
mlh3pch2−6D
Read counts
0
1000
2000
3000
500
1500
chr01
chr03
chr04
chr05
chr07
chr08
chr10
chr12
chr13
chr14
chr15
chr16

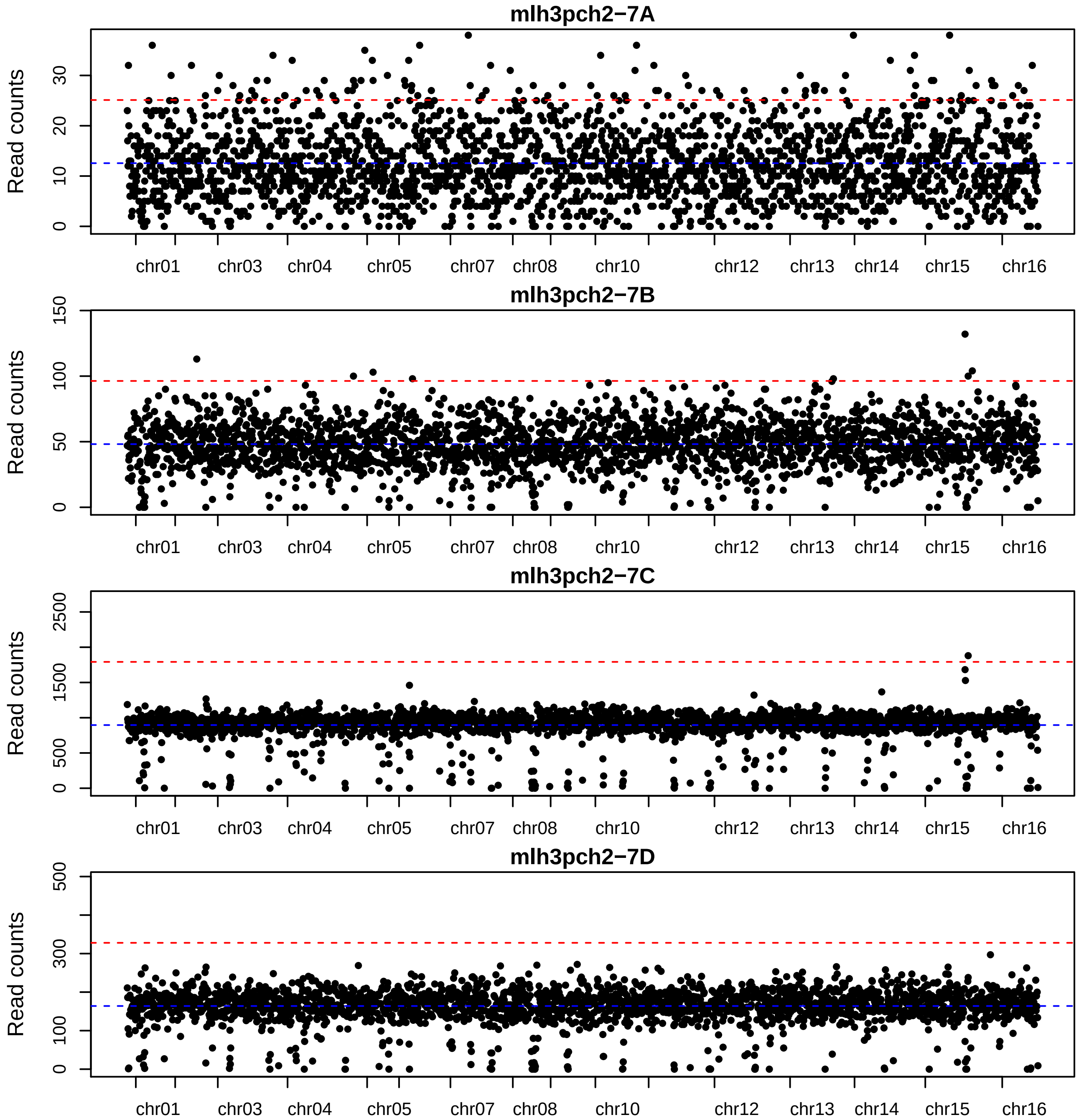
mlh3pch2−7A
Read counts
0
10
20
30
chr01
chr03
chr04
chr05
chr07
chr08
chr10
chr12
chr13
chr14
chr15
chr16
mlh3pch2−7B
Read counts
0
50
100
150
chr01
chr03
chr04
chr05
chr07
chr08
chr10
chr12
chr13
chr14
chr15
chr16
mlh3pch2−7C
Read counts
0
500
1500
2500
chr01
chr03
chr04
chr05
chr07
chr08
chr10
chr12
chr13
chr14
chr15
chr16
mlh3pch2−7D
Read counts
0
100
300
500
chr01
chr03
chr04
chr05
chr07
chr08
chr10
chr12
chr13
chr14
chr15
chr16

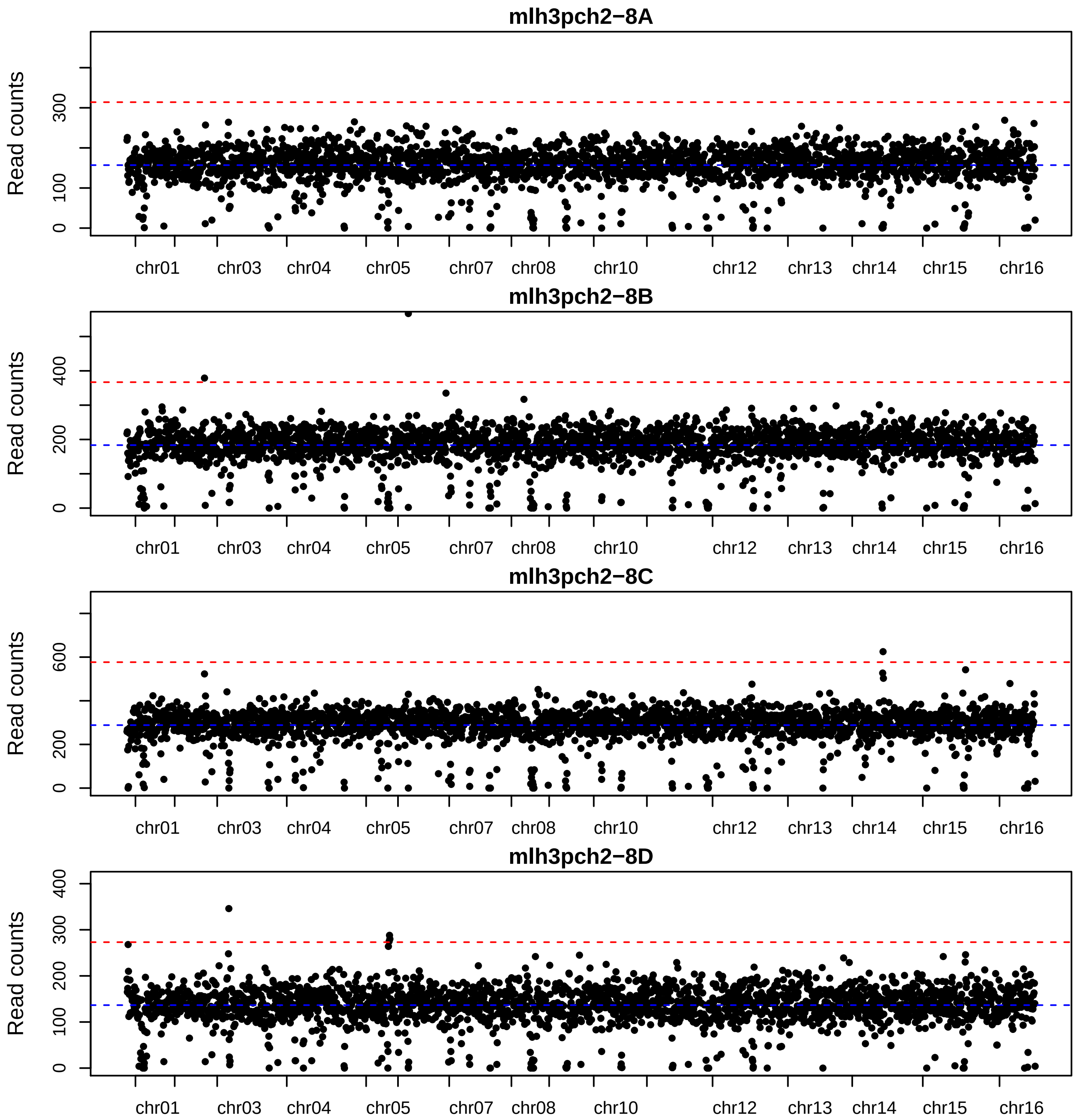
mlh3pch2−8A
Read counts
0
100
300
chr01
chr03
chr04
chr05
chr07
chr08
chr10
chr12
chr13
chr14
chr15
chr16
mlh3pch2−8B
Read counts
0
200
400
chr01
chr03
chr04
chr05
chr07
chr08
chr10
chr12
chr13
chr14
chr15
chr16
mlh3pch2−8C
Read counts
0
200
600
chr01
chr03
chr04
chr05
chr07
chr08
chr10
chr12
chr13
chr14
chr15
chr16
mlh3pch2−8D
Read counts
0
100
200
300
400
chr01
chr03
chr04
chr05
chr07
chr08
chr10
chr12
chr13
chr14
chr15
chr16

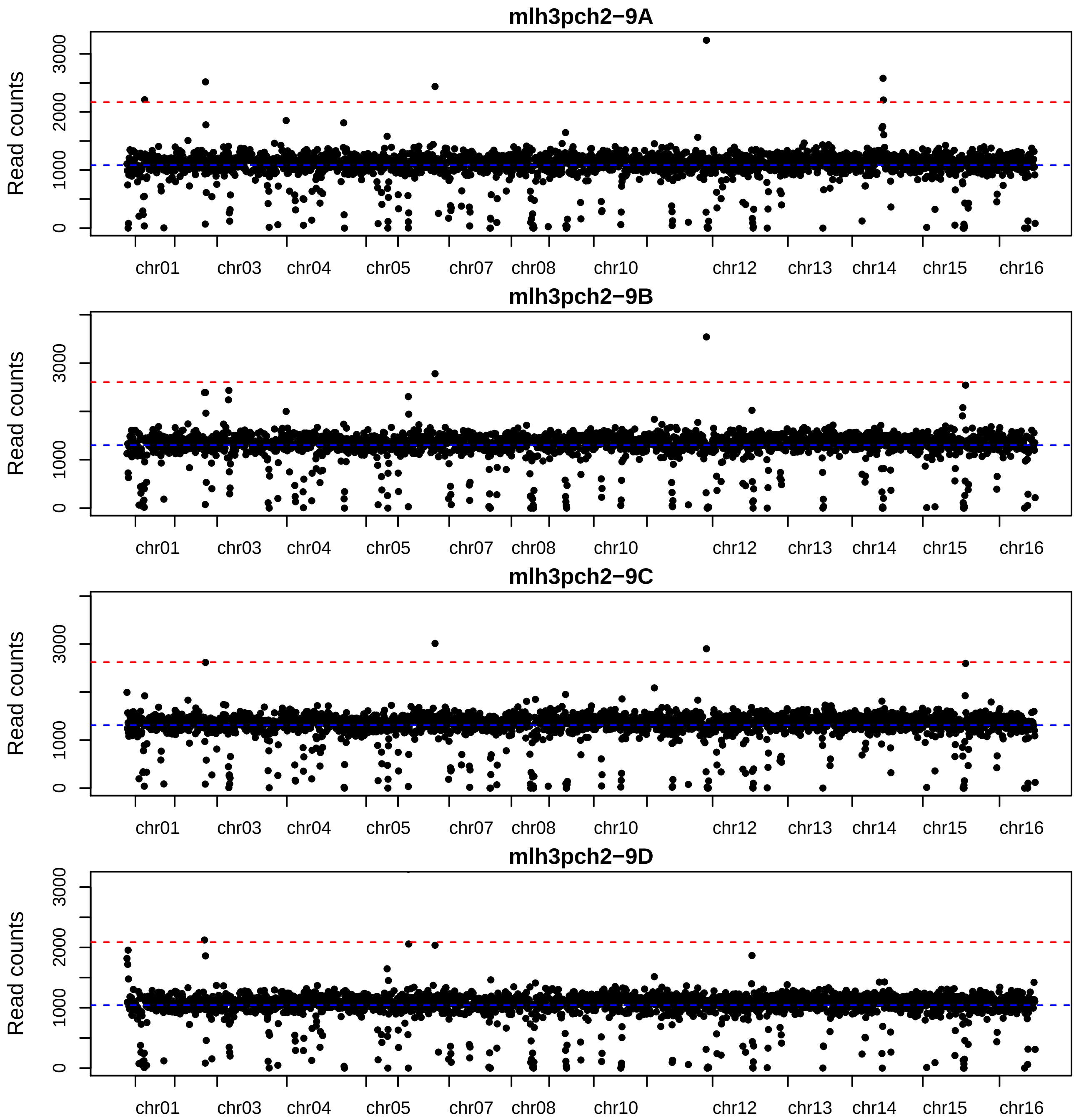
mlh3pch2−9A
mlh3pch2−9B
mlh3pch2−9C
mlh3pch2−9D
Read counts
0
1000
2000
3000
chr01
chr03
chr04
chr05
chr07
chr08
chr10
chr12
chr13
chr14
chr15
chr16

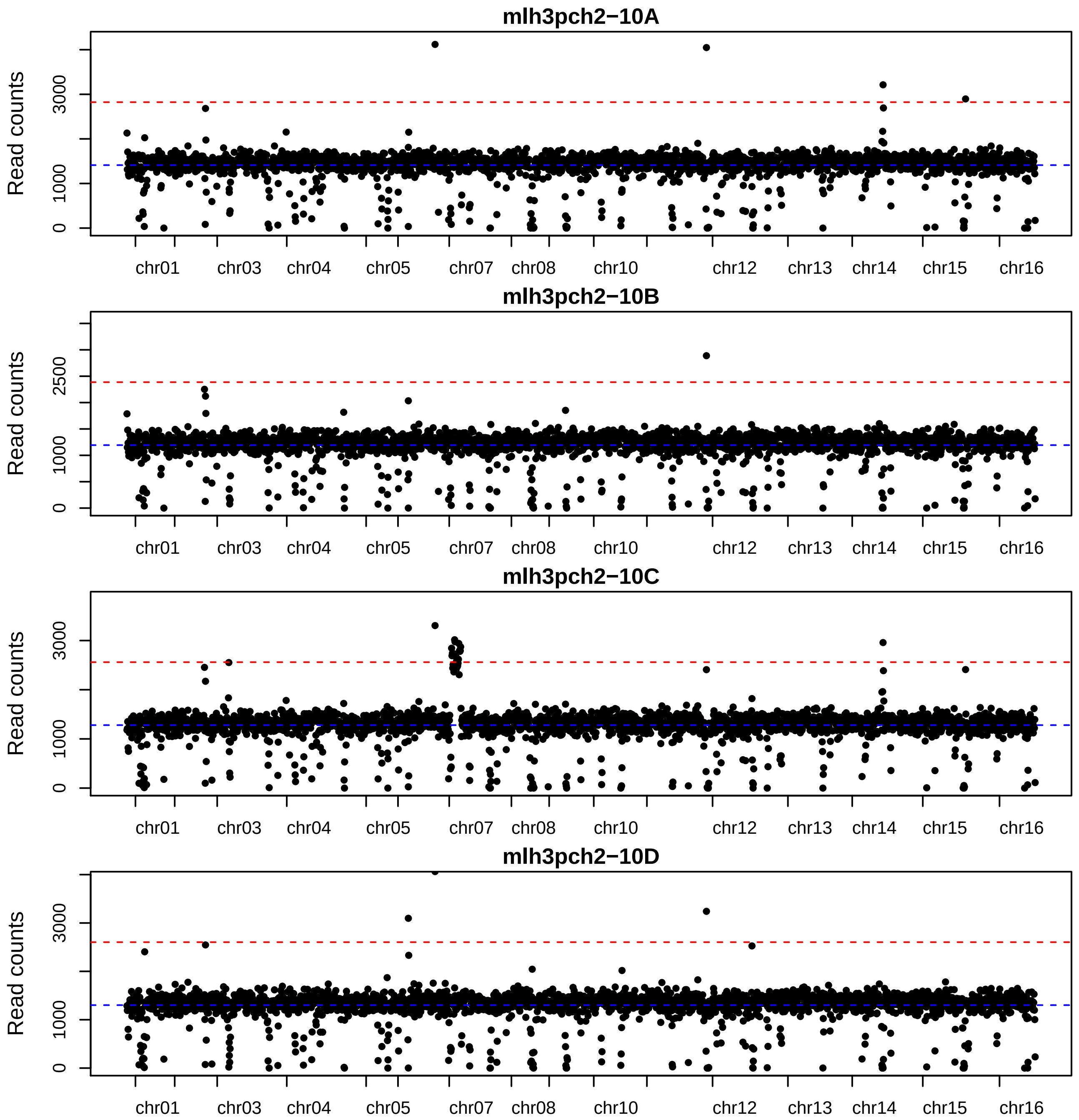

mlh3pch2−10A
mlh3pch2−10B
mlh3pch2−10C
mlh3pch2−10D
Read counts
0
1000
2500
3000
chr01
chr03
chr04
chr05
chr07
chr08
chr10
chr12
chr13
chr14
chr15
chr16

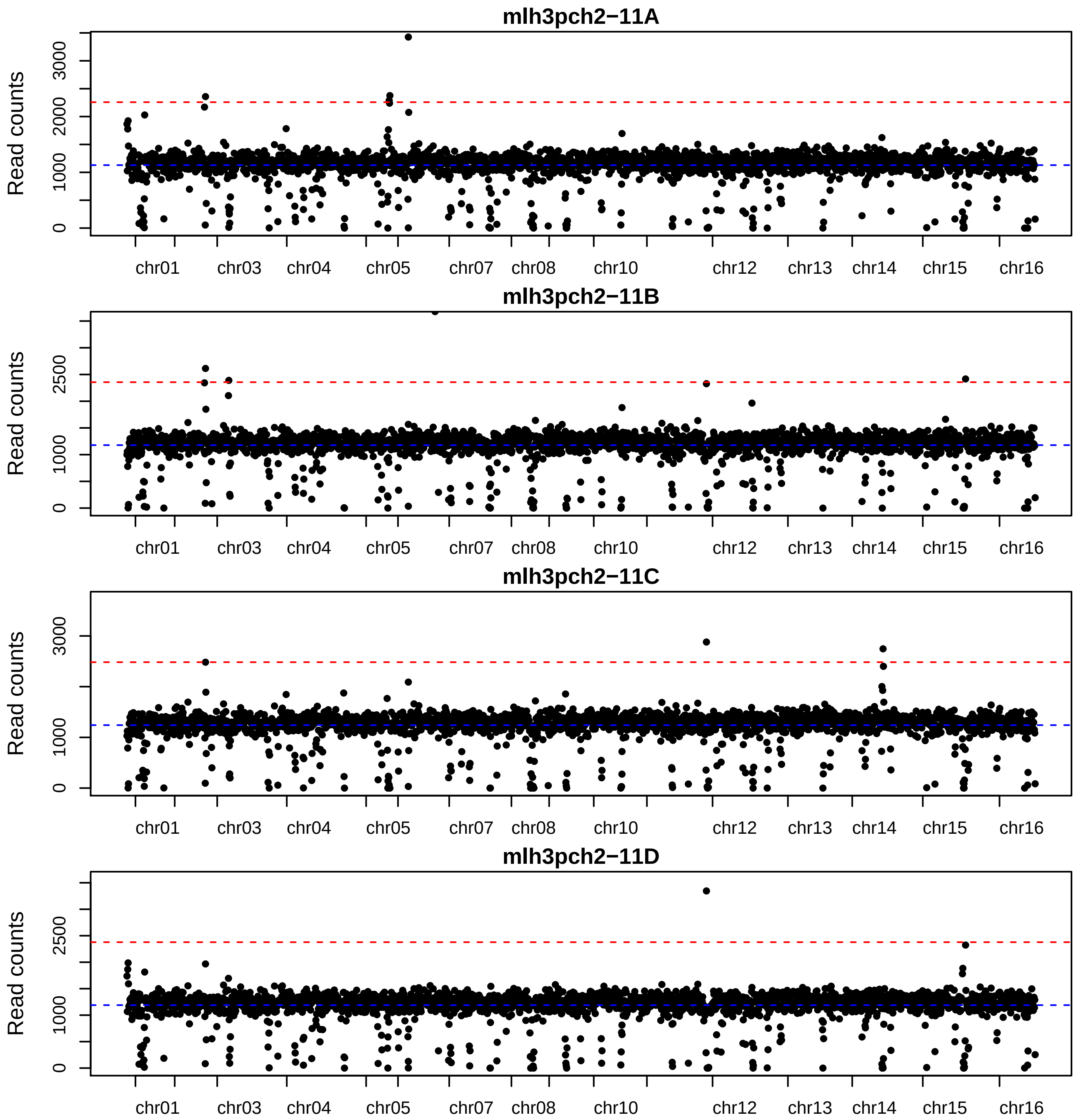
mlh3pch2−11A
Read counts
0
1000
2000
3000
chr01
chr03
chr04
chr05
chr07
chr08
chr10
chr12
chr13
chr14
chr15
chr16
mlh3pch2−11B
Read counts
0
1000
2500
chr01
chr03
chr04
chr05
chr07
chr08
chr10
chr12
chr13
chr14
chr15
chr16
mlh3pch2−11C
Read counts
0
1000
3000
chr01
chr03
chr04
chr05
chr07
chr08
chr10
chr12
chr13
chr14
chr15
chr16
mlh3pch2−11D
Read counts
0
1000
2500
chr01
chr03
chr04
chr05
chr07
chr08
chr10
chr12
chr13
chr14
chr15
chr16

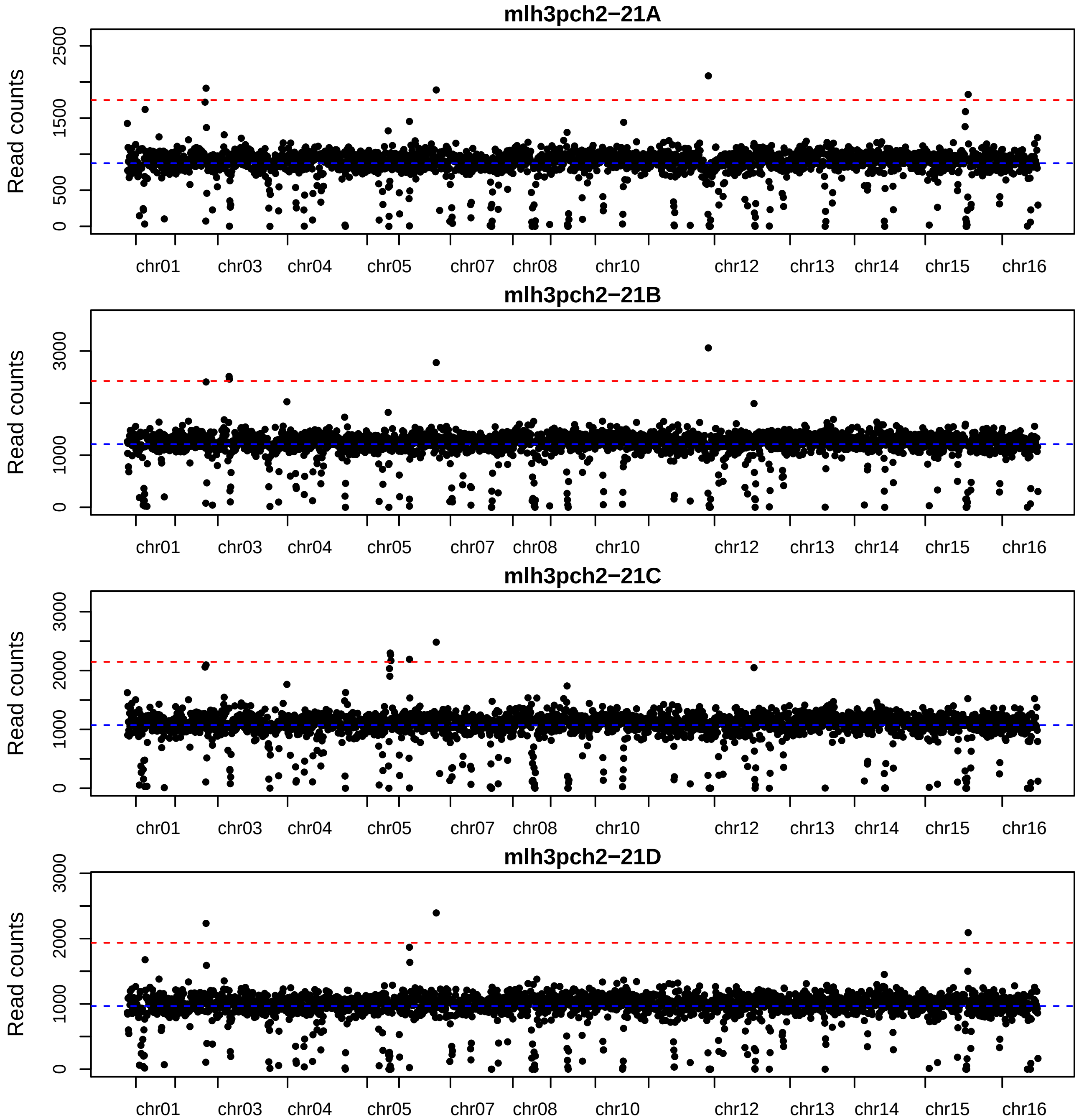
mlh3pch2−21A
mlh3pch2−21B
mlh3pch2−21C
mlh3pch2−21D
Read counts
0
500
1500
2500
1000
3000
2000
chr01
chr03
chr04
chr05
chr07
chr08
chr10
chr12
chr13
chr14
chr15
chr16

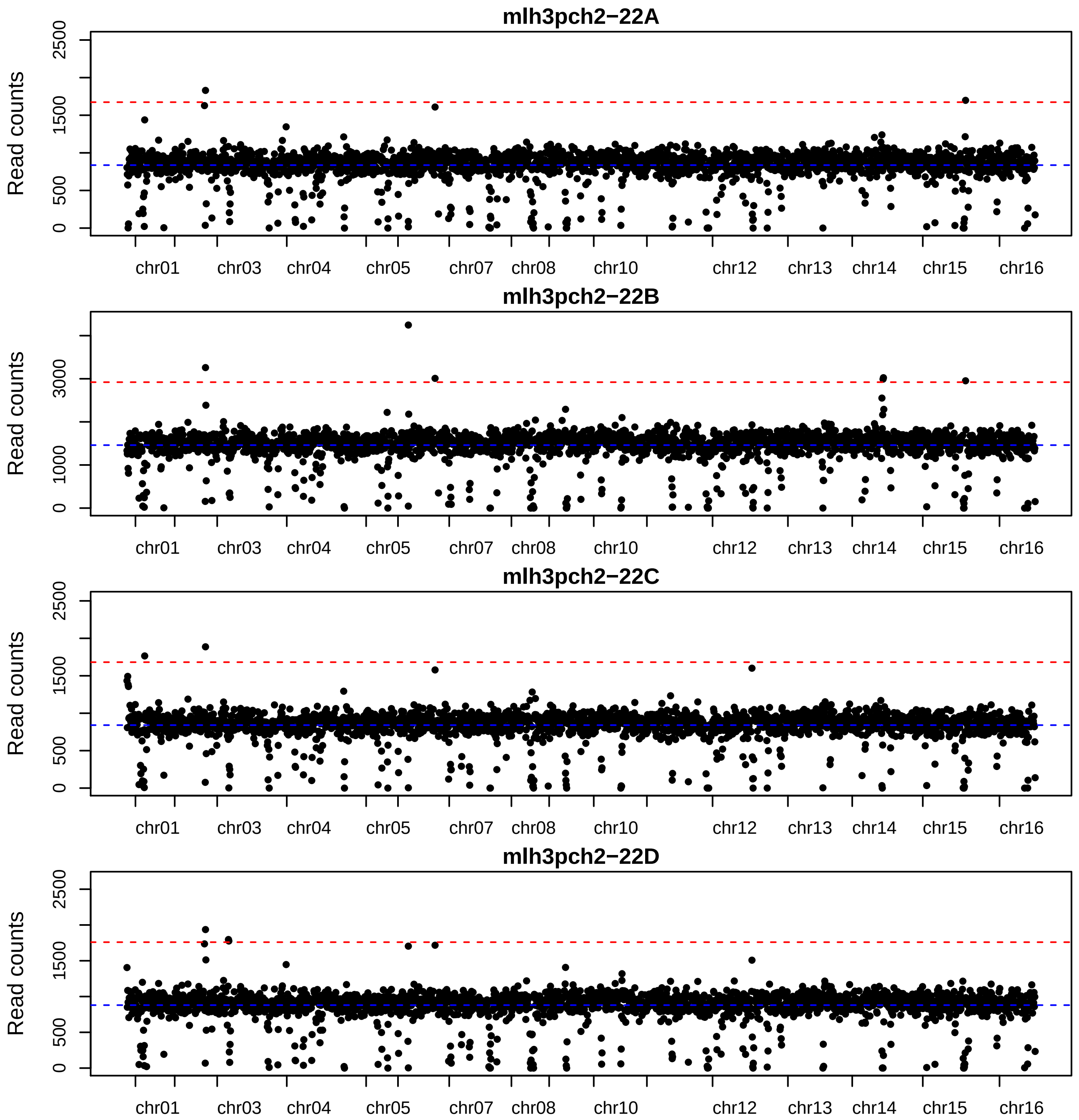
mlh3pch2−22A
mlh3pch2−22B
mlh3pch2−22C
mlh3pch2−22D
Read counts
0
500
1000
1500
2500
3000
chr01
chr03
chr04
chr05
chr07
chr08
chr10
chr12
chr13
chr14
chr15
chr16

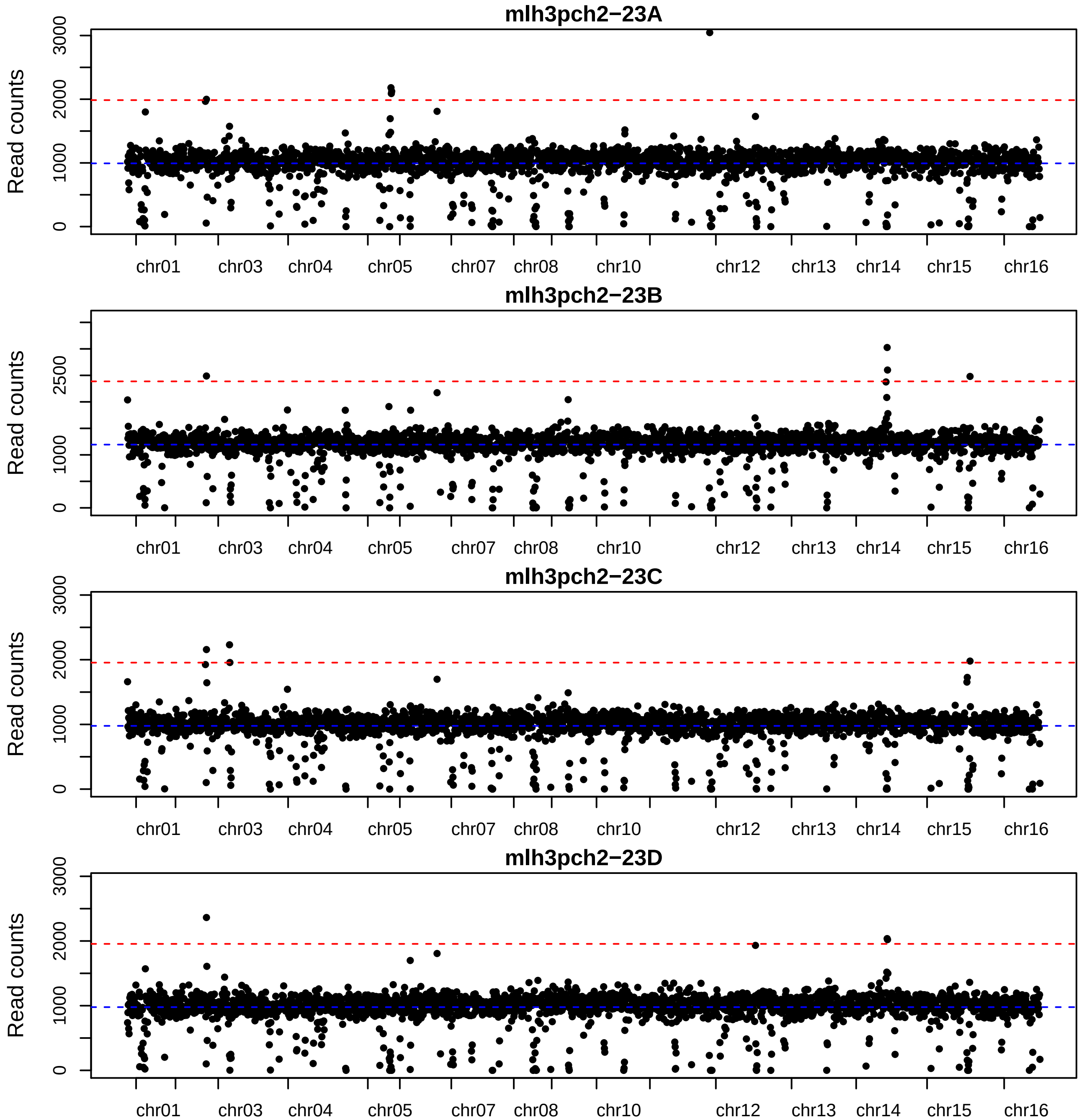
mlh3pch2−23A
mlh3pch2−23B
mlh3pch2−23C
mlh3pch2−23D
Read counts
0
1000
2000
2500
3000
chr01
chr03
chr04
chr05
chr07
chr08
chr10
chr12
chr13
chr14
chr15
chr16

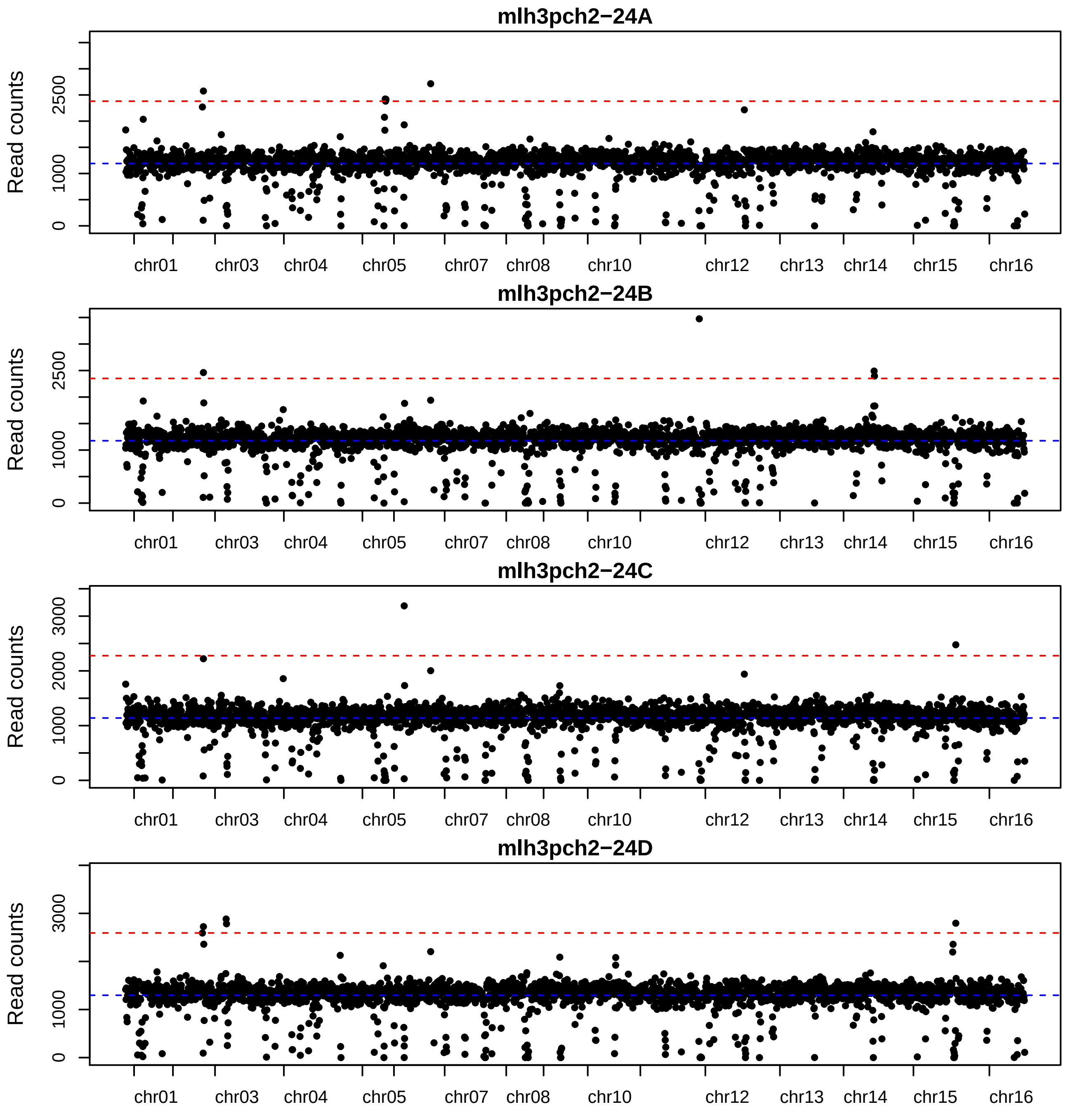
mlh3pch2−24A
mlh3pch2−24B
mlh3pch2−24C
mlh3pch2−24D
Read counts
0
1000
2000
2500
3000
chr01
chr03
chr04
chr05
chr07
chr08
chr10
chr12
chr13
chr14
chr15
chr16

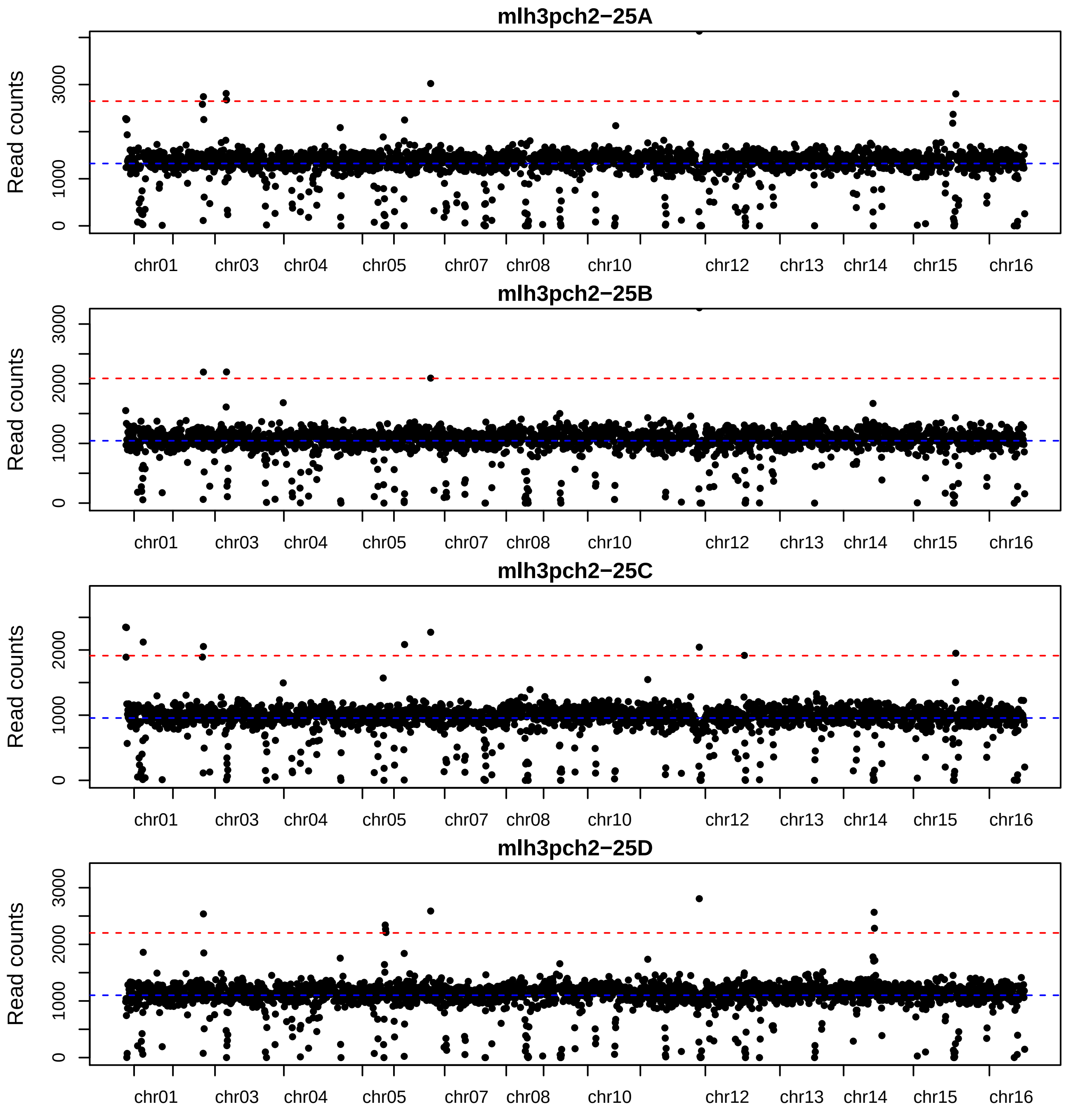
mlh3pch2−25A
mlh3pch2−25B
mlh3pch2−25C
mlh3pch2−25D
Read counts
0
1000
2000
3000
chr01
chr03
chr04
chr05
chr07
chr08
chr10
chr12
chr13
chr14
chr15
chr16

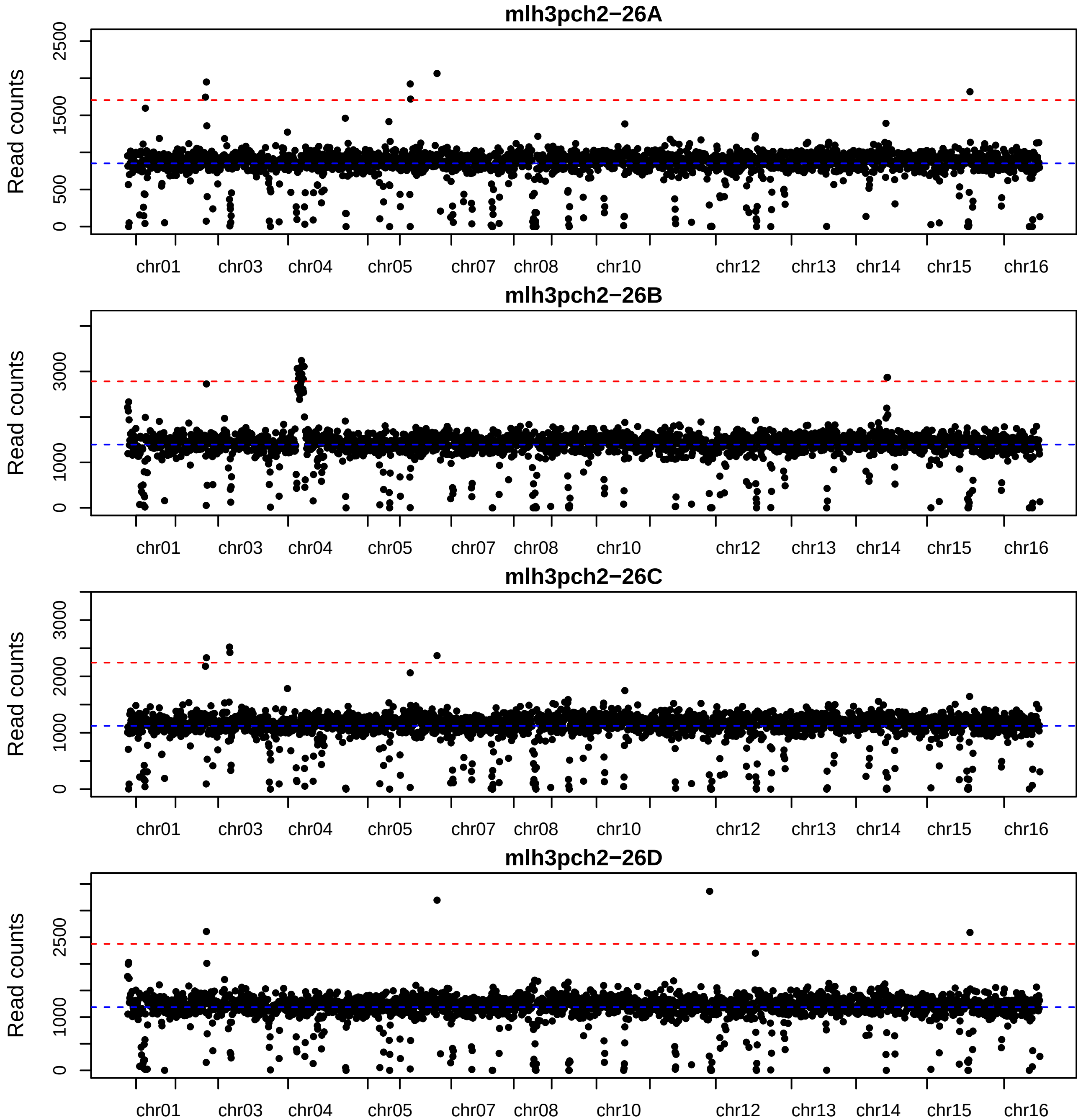
mlh3pch2−26A
Read counts
0
500
1500
2500
chr01
chr03
chr04
chr05
chr07
chr08
chr10
chr12
chr13
chr14
chr15
chr16
mlh3pch2−26B
Read counts
0
1000
3000
mlh3pch2−26C
Read counts
0
1000
2000
3000
mlh3pch2−26D
Read counts
0
1000
2500

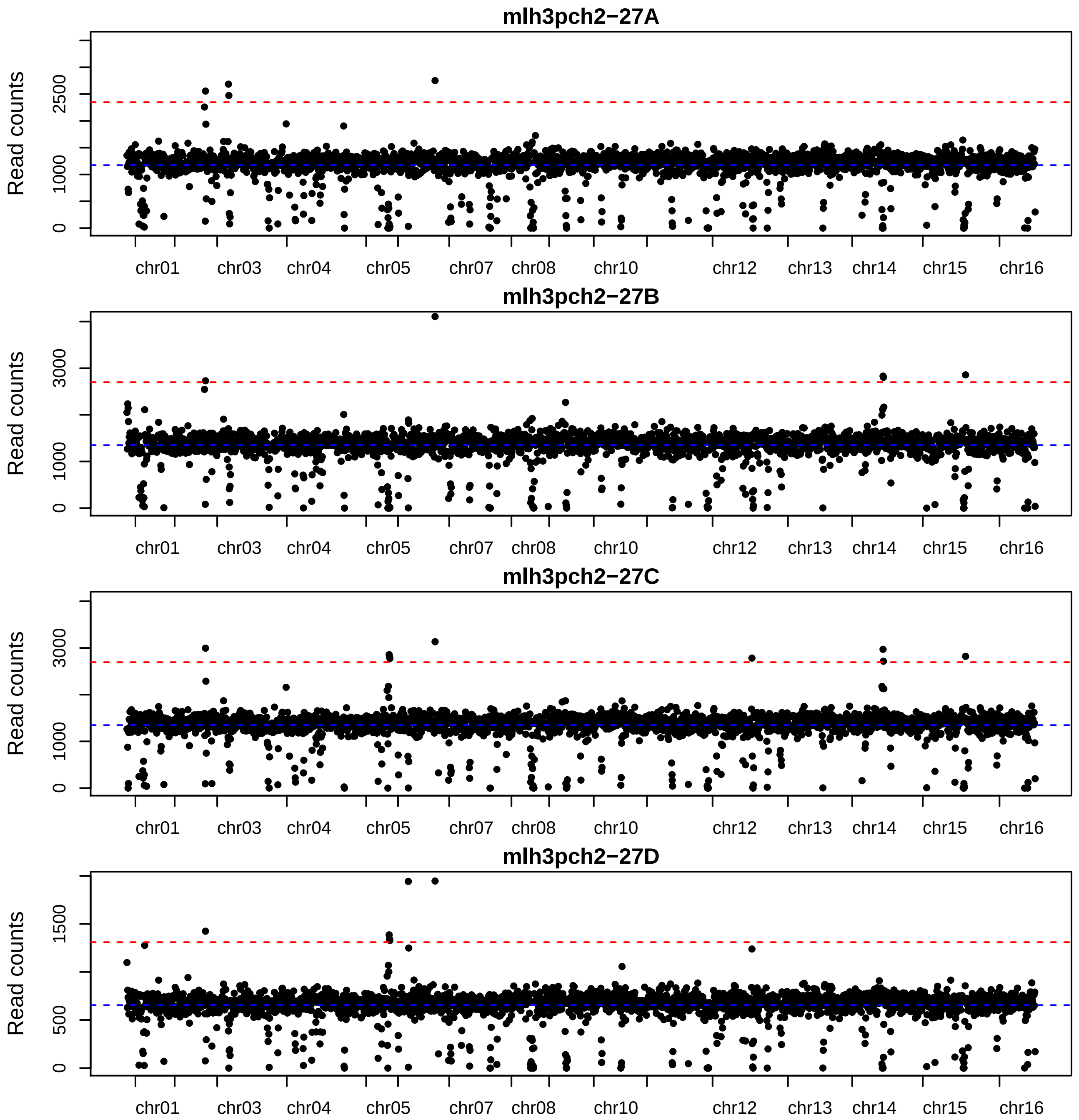
mlh3pch2−27A
Read counts
0
1000
2500
chr01
chr03
chr04
chr05
chr07
chr08
chr10
chr12
chr13
chr14
chr15
chr16
mlh3pch2−27B
Read counts
0
1000
3000
mlh3pch2−27C
Read counts
0
1000
3000
mlh3pch2−27D
Read counts
0
500
1500

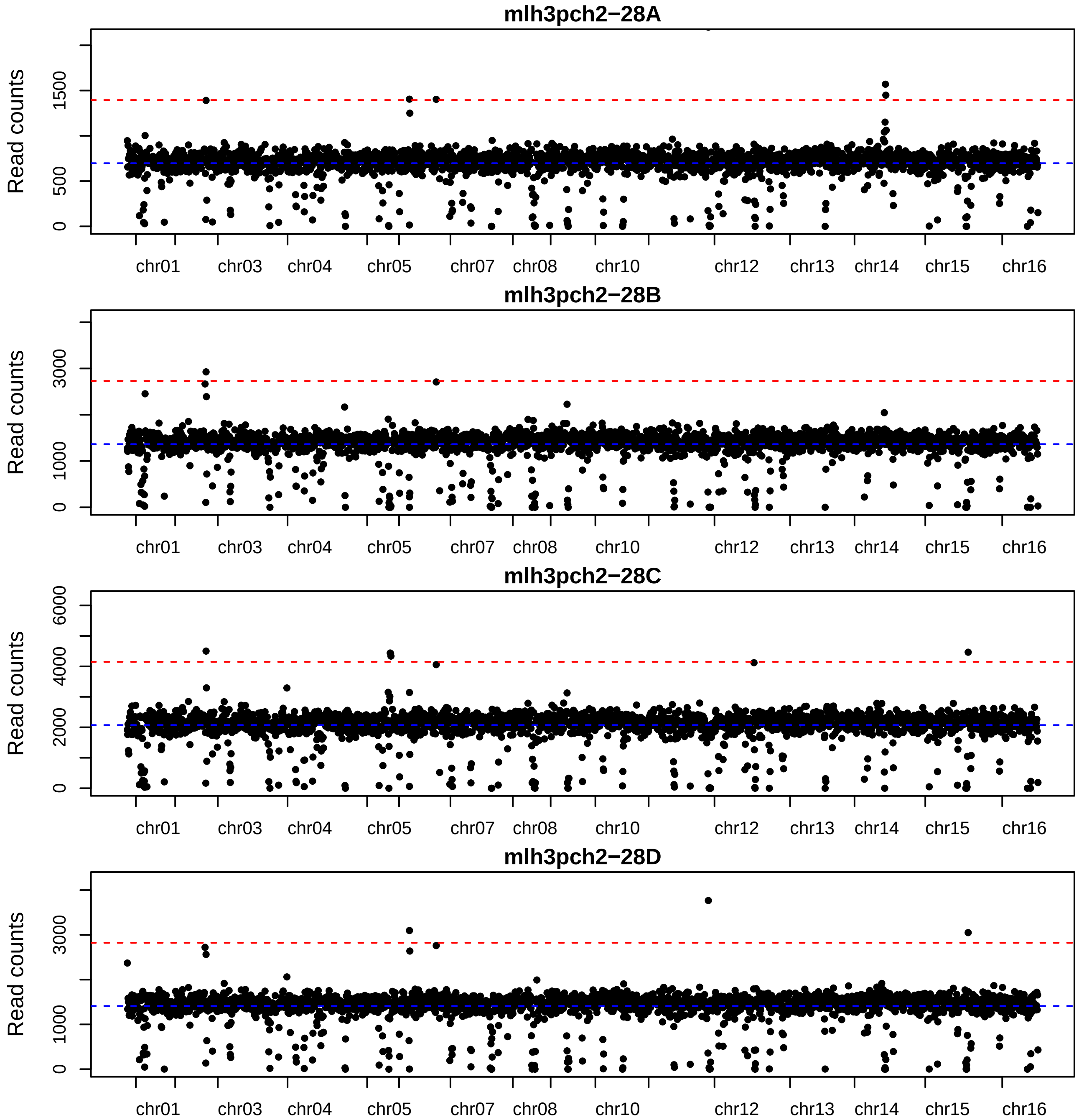
mlh3pch2−28A
mlh3pch2−28B
mlh3pch2−28C
mlh3pch2−28D
Read counts
chr01
chr03
chr04
chr05
chr07
chr08
chr10
chr12
chr13
chr14
chr15
chr16

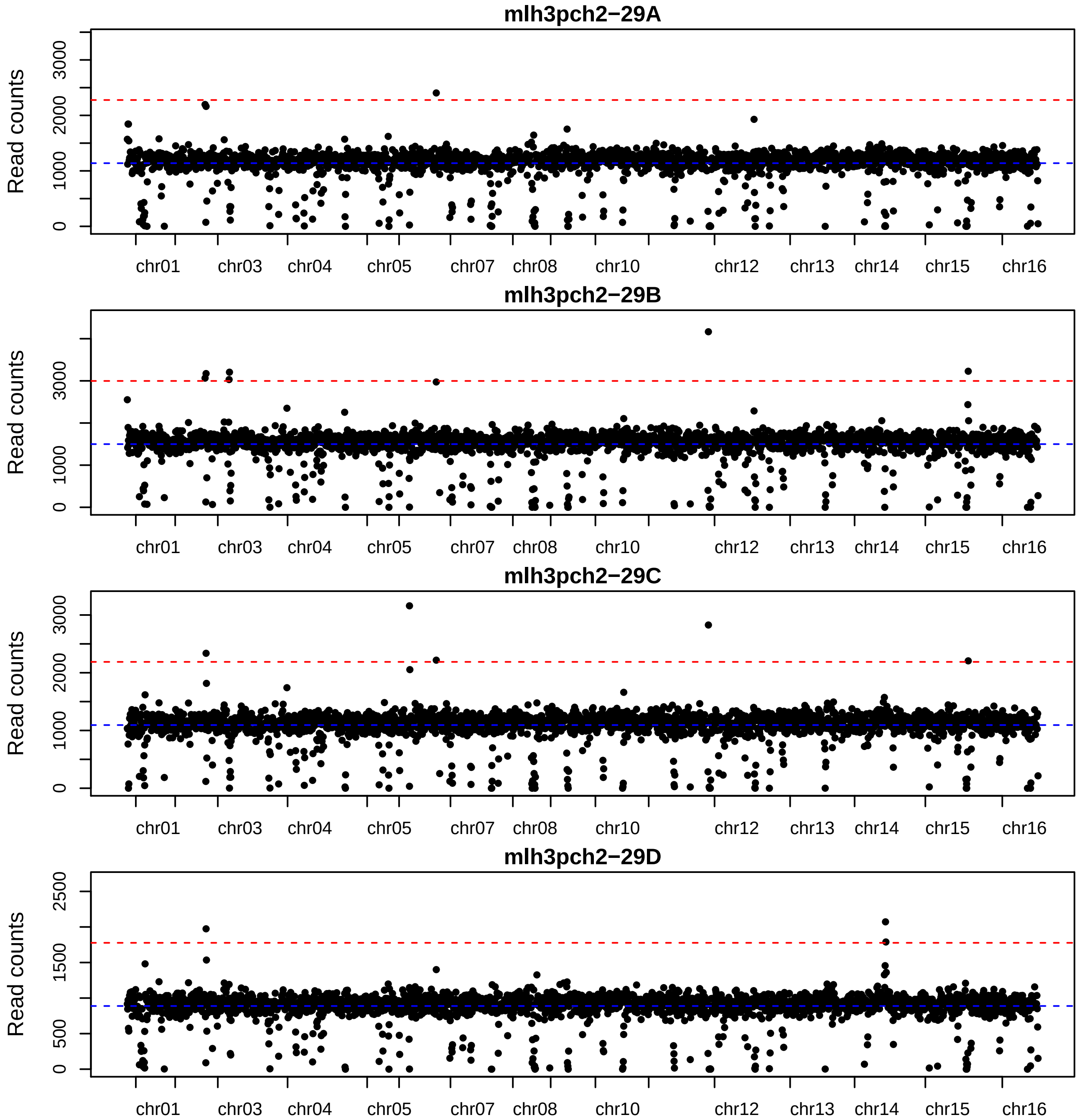
mlh3pch2−29A
mlh3pch2−29B
mlh3pch2−29C
mlh3pch2−29D
Read counts
0
500
1000
1500
2000
2500
3000
chr01
chr03
chr04
chr05
chr07
chr08
chr10
chr12
chr13
chr14
chr15
chr16

Figure S9 Genome wide coverage plot to detect aneuploidy in sequenced spores from *mlh3Δ*, *pch2Δ* and *mlh3Δ pch2Δ* tetrads. Each dot indicates the total read count in 5 kb bins. Average read count is shown as dotted blue line. Red line indicates 2X average read count.
