# Supplementary material for: Modulating Crossover Frequency and Interference for Obligate Crossovers in Saccharomyces cerevisiae Meiosis
Source: G3 (Bethesda). 2017 Mar 17;7(5):1511–24. doi: 10.1534/g3.117.040071 (PMC5427503; doi:10.1534/g3.117.040071)
Supplement: Supplementary file 10 [file 1511TableS1.docx]

| **Strain** | **Genotype** | **Source** |
| --- | --- | --- |
| S288c | *MATa ho lys5* | Mancera *et al.* 2008 |
| KTY448 | as S288c except *mlh3∆::kanMX4* | This study |
| KTY336 | as S288c except *pch2∆::kanMX4* | This study |
| KTY357 | as S288c except *mlh3Δ:: kanMX4, pch2Δ::natMX4* | This study |
| YJM789 | *MATα ho::hisG lys2 cyh* | Mancera *et al.* 2008 |
| KTY451 | as YJM789 except *mlh3Δ::kanMX4* | This Study |
| KTY335 | as YJM789 except *pch2Δ::natMX4* | This Study |
| KTY355 | as YJM789 except *mlh3Δ::kanMX4, pch2Δ::natMX4* | This Study |
| EAY1108 | *MATa, ho∷hisG, lys2, ura3, leu2∷hisG,trp1∷hisG, URA3-cenXVi, LEU2-chXVi, LYS2-chXVi* | Argueso *et al.* 2004 |
| KTY501 | as EAY1108 except *pch2Δ::natMX4, mlh3Δ::hphMX4* | This Study |
| KTY523 | as EAY1108 except *pch2Δ::natMX4, mlh3Δ::hphMX4, slx4:: kanMX4* | This Study |
| KTY606 | as EAY1108 except *pch2Δ::natMX4, mlh3Δ::hphMX4, kanMX-pCLB2-3HA-MMS4* | This Study |
| EAY1112 | *MATα, ho∷hisG,lys2, ura3, leu2∷hisG, trp1∷hisG, ade2::hisG, his3∷hisG, TRP1-cenXVi* | Argueso *et al.* 2004 |
| KTY503 | as EAY1112 except *pch2Δ::natMX4, mlh3Δ::hphMX4* | This Study |
| KTY525 | as EAY1112 except *pch2Δ::natMX4, mlh3Δ::hphMX4, slx4::kanMX4* | This Study |
| KTY608 | as EAY1112 except *pch2Δ::natMX4, mlh3Δ::hphMX4, kanMX-pCLB2-3HA-MMS4* | This Study |
| NHY1162 | *MAT*α*, ho::hisG, leu::hisG, ura3(∆Sma-Pst), his4X::LEU2-(NgoMIV)::URA3* | Martini *et al.* 2006 |
| NHY1168 | *MATa, ho::hisG, leu::hisG, ura3(∆Sma-Pst), HIS4::LEU2-(BamHI)* | Martini *et al.* 2006 |
| KTY444 | as NHY1162 except *pch2Δ::natMX4* | This Study |
| KTY445 | as NHY1168 except *pch2Δ::natMX4* | This Study |

**Table S1**  **List of strains used in this study.**

**Literature cited**

Argueso, J. L., J. Wanat, Z. Gemici and E. Alani, 2004 Competing crossover pathways act during meiosis in *Saccharomyces cerevisiae*. Genetics 168**:** 1805-1816.

Mancera, E., R. Bourgon, A. Brozzi, W. Huber and L. M. Steinmetz, 2008 High-resolution mapping of meiotic crossovers and non-crossovers in yeast. Nature 454**:** 479-485.

Martini, E., R. L. Diaz, N. Hunter and S. Keeney, 2006 Crossover homeostasis in yeast meiosis. Cell 126**:** 285-295.
