# Supplementary material for: Modulating Crossover Frequency and Interference for Obligate Crossovers in Saccharomyces cerevisiae Meiosis
Source: G3 (Bethesda). 2017 Mar 17;7(5):1511–24. doi: 10.1534/g3.117.040071 (PMC5427503; doi:10.1534/g3.117.040071)
Supplement: Supplementary file 13 [file 1511TableS4.docx]

**Table S4** **Average crossovers (CO) and non-crossovers (NCO) per chromosome for wild type, *mlh3Δ, pch2Δ* and *mlh3Δ pch2Δ* mutants.**

| **Chromosome** | **Wild type** | ***mlh3Δ*** | ***pch2Δ*** | ***mlh3Δ pch2Δ*** |
| --- | --- | --- | --- | --- |
| I | 2.35 | 2.00 | 2.53 | 1.55 |
| II | 6.41 | 4.32 | 10.27 | 6.80 |
| III | 3.36 | 2.63 | 4.93 | 3.20 |
| IV | 10.11 | 7.47 | 16.07 | 12.5 |
| V | 4.56 | 2.79 | 5.93 | 3.75 |
| VI | 2.65 | 2.11 | 3.40 | 2.55 |
| VII | 8.32 | 5.63 | 12.07 | 9.20 |
| VIII | 4.71 | 3.32 | 6.93 | 5.25 |
| IX | 3.53 | 2.58 | 4.20 | 3.30 |
| X | 5.98 | 4.16 | 9.00 | 6.70 |
| XI | 5.12 | 3.37 | 7.60 | 4.60 |
| XII | 8.58 | 5.63 | 13.00 | 9.95 |
| XIII | 6.73 | 4.21 | 9.40 | 6.90 |
| XIV | 5.94 | 4.26 | 7.67 | 6.15 |
| XV | 7.85 | 5.11 | 11.27 | 9.80 |
| XVI | 7.24 | 4.84 | 12.27 | 7.60 |
| **Total_CO** | **93.4** | **64.4** | **136.5** | **99.8** |
|  |  |  |  |  |
| **Chromosome** | **Wild type** | ***mlh3Δ*** | ***pch2Δ*** | ***mlh3Δ pch2Δ*** |
| I | 1.26 | 0.95 | 1.33 | 1.90 |
| II | 3.05 | 3.74 | 6.33 | 7.35 |
| III | 1.32 | 1.68 | 2.33 | 3.05 |
| IV | 5.23 | 5.05 | 11.27 | 12.15 |
| V | 2.56 | 2.68 | 4.13 | 3.50 |
| VI | 1.21 | 1.42 | 2.27 | 2.15 |
| VII | 4.02 | 3.84 | 7.87 | 10.85 |
| VIII | 1.97 | 2.16 | 3.20 | 3.85 |
| IX | 1.77 | 2.26 | 2.53 | 3.20 |
| X | 2.86 | 3.26 | 5.33 | 5.10 |
| XI | 2.64 | 2.84 | 5.40 | 3.95 |
| XII | 3.58 | 4.58 | 8.67 | 8.80 |
| XIII | 4.12 | 4.53 | 5.80 | 5.80 |
| XIV | 2.80 | 3.05 | 5.13 | 4.95 |
| XV | 3.80 | 4.11 | 8.53 | 9.25 |
| XVI | 3.82 | 3.32 | 5.80 | 7.80 |
| **Total_NCO** | **46** | **49.5** | **85.9** | **93.6** |
