# Supplementary material for: Modulating Crossover Frequency and Interference for Obligate Crossovers in Saccharomyces cerevisiae Meiosis
Source: G3 (Bethesda). 2017 Mar 17;7(5):1511–24. doi: 10.1534/g3.117.040071 (PMC5427503; doi:10.1534/g3.117.040071)
Supplement: Supplementary file 14 [file 1511TableS5.docx]

**Table S5** **Marker segregation frequency and gene conversion tracts in wild type, *mlh3Δ, pch2Δ* and *mlh3Δ pch2Δ* mutants.**

| **Strain** | **2:2** | **3:1** | **1:3** | **4:0** | **0:4** | **Median CO tract length (bp)** | **Median NCO tract length (bp)** | ***P* value** |
| --- | --- | --- | --- | --- | --- | --- | --- | --- |
|  |  |  |  |  |  |  |  |  |
| Wild type | 98.04 | 0.94 | 0.96 | 0.01 | 0.03 | 1958.5 | 1495.25 | 4.6 × 10^-42^ |
| *mlh3Δ* | 97.99 | 1.04 | 0.92 | 0.03 | 0.01 | 2420 | 1737 | 1.5 x 10^-20^ |
| *pch2Δ* | 94.71 | 2.58 | 2.58 | 0.06 | 0.03 | 2668.75 | 1838.5 | 2.2 x 10^-20^ |
| *mlh3Δ pch2Δ* | 94.02 | 2.96 | 2.91 | 0.06 | 0.02 | 3927.5 | 2272 | 6.6 x 10^-42^ |

The percentage of SNP markers with 2:2, 3:1, 1:3, 4:0 and 0:4 segregation (S288c:YJM789) is shown. Median gene conversion tract lengths in base pairs for crossover (CO) and non-crossover (NCO) are also shown. Statistical significance of difference between median CO and NCO gene conversion length is determined using the Wilcoxon rank sum test.
