# Supplementary material for: Modulating Crossover Frequency and Interference for Obligate Crossovers in Saccharomyces cerevisiae Meiosis
Source: G3 (Bethesda). 2017 Mar 17;7(5):1511–24. doi: 10.1534/g3.117.040071 (PMC5427503; doi:10.1534/g3.117.040071)
Supplement: Supplementary file 15 [file 1511TableS6.docx]

**Table S6** **Genetic map distances from tetrad data in wild type, *mlh3Δ pch2Δ* and *mlh3Δ pch2Δ slx4Δ*  mutants in the EAY1108/EAY1112 genetic background.**

|  | **Tetrads** | | | |  | |
| --- | --- | --- | --- | --- | --- | --- |
| **Genotype** | **N** | **PD** | **TT** | **NPD** | **cM** | **S.E** |
|  |  |  |  |  |  |  |
| *URA3-LEU2* |  |  |  |  |  |  |
| Wild type | 641 | 361 | 245 | 0 | 20.2 | 1.0 |
| *mlh3Δ pch2Δ* | 98 | 60 | 32 | 3 | 26.3 | 5.6 |
| *mlh3Δ pch2Δ slx4Δ* | 69 | 36 | 27 | 0 | 21.4 | 3.1 |
| *LEU2-LYS2* |  |  |  |  |  |  |
| Wild type | 641 | 271 | 329 | 6 | 30.1 | 1.5 |
| *mlh3Δ pch2Δ* | 98 | 66 | 27 | 2 | 20.5 | 4.8 |
| *mlh3Δ pch2Δ slx4Δ* | 69 | 48 | 14 | 1 | 15.9 | 5.2 |
| *LYS2-ADE2* |  |  |  |  |  |  |
| Wild type | 641 | 450 | 155 | 1 | 13.3 | 1.0 |
| *mlh3Δ pch2Δ* | 98 | 71 | 21 | 3 | 20.5 | 5.6 |
| *mlh3Δ pch2Δ slx4Δ* | 69 | 54 | 9 | 0 | 7.1 | 2.2 |
| *ADE2-HIS3* |  |  |  |  |  |  |
| Wild type | 641 | 234 | 345 | 27 | 41.8 | 2.5 |
| *mlh3Δ pch2Δ* | 98 | 42 | 46 | 7 | 46.3 | 7.7 |
| *mlh3Δ pch2Δ slx4Δ* | 69 | 29 | 29 | 5 | 46.8 | 9.8 |

Standard error (S.E) around the genetic distances (cM) was calculated using The Stahl Laboratory Online Tools website (http://groik.com/stahl/). N: four viable spore tetrads analysed.
