# Supplementary material for: Modulating Crossover Frequency and Interference for Obligate Crossovers in Saccharomyces cerevisiae Meiosis
Source: G3 (Bethesda). 2017 Mar 17;7(5):1511–24. doi: 10.1534/g3.117.040071 (PMC5427503; doi:10.1534/g3.117.040071)
Supplement: Supplementary file 16 [file 1511TableS7.docx]

**Table S7 Genetic map distances from spore data in wild type, *mlh3Δ pch2Δ*, *mlh3Δ pch2Δ mms4-md* and *mlh3Δ pch2Δ slx4Δ* mutants in the EAY1108/EAY1112 genetic background.**

|  | **Single spores** | | | |  | |
| --- | --- | --- | --- | --- | --- | --- |
| **Genotype** | **n** | **Par.** | **Rec.** | **cM** | | **95% C.I** |
|  |  |  |  |  | |  |
| *URA3-LEU2* |  |  |  |  | |  |
| Wild type | 2775 | 2207 | 568 | 20.5 | | 19-22 |
| *mlh3Δ pch2Δ* | 562 | 460 | 102 | 18.1 | | 15.2-21.5 |
| *mlh3Δ pch2Δ mms4-md* | 392 | 373 | 19 | 4.8 | | 3.1-7.4 |
| *mlh3Δ pch2Δ slx4Δ* | 571 | 432 | 139 | 24.3 | | 21-28 |
| *LEU2-LYS2* |  |  |  |  | |  |
| Wild type | 2775 | 1979 | 796 | 28.7 | | 27-30.4 |
| *mlh3Δ pch2Δ* | 562 | 470 | 92 | 16.4 | | 13.5-19.7 |
| *mlh3Δ pch2Δ mms4-md* | 392 | 365 | 27 | 6.9 | | 4.8-9.8 |
| *mlh3Δ pch2Δ slx4Δ* | 571 | 478 | 93 | 16.3 | | 13.5-19.5 |
| *LYS2-ADE2* |  |  |  |  | |  |
| Wild type | 2775 | 2401 | 374 | 13.5 | | 12.3-14.8 |
| *mlh3Δ pch2Δ* | 562 | 481 | 81 | 14.4 | | 11.7-17.6 |
| *mlh3Δ pch2Δ mms4-md* | 392 | 381 | 11 | 2.8 | | 1.6-5.0 |
| *mlh3Δ pch2Δ slx4Δ* | 571 | 521 | 50 | 8.8 | | 6.7-11.4 |
| *ADE2-HIS3* |  |  |  |  | |  |
| Wild type | 2775 | 1835 | 940 | 33.9 | | 32.1-35.6 |
| *mlh3Δ pch2Δ* | 562 | 388 | 174 | 31 | | 27.3-34.9 |
| *mlh3Δ pch2Δ mms4-md* | 392 | 356 | 36 | 9.2 | | 6.7-12.4 |
| *mlh3Δ pch2Δ slx4Δ* | 571 | 409 | 162 | 28.4 | | 24.8-32.2 |

Genetic map distances (cM) were calculated by multiplying recombination frequencies (recombinant spores / total spores) by100. 95% confidence intervals for genetic map distance were determined using VassarStats (http://faculty.vassar.edu/lowry/VassarStats.html).

n: number of single spores, Par. : parental single spores, Rec. : recombinant single spores
