# Supplementary material for: Modulating Crossover Frequency and Interference for Obligate Crossovers in Saccharomyces cerevisiae Meiosis
Source: G3 (Bethesda). 2017 Mar 17;7(5):1511–24. doi: 10.1534/g3.117.040071 (PMC5427503; doi:10.1534/g3.117.040071)
Supplement: Supplementary file 17 [file 1511TableS8.docx]

**Table S8** **Chromatid interference in wild type, *mlh3Δ*, *pch2Δ* and *mlh3Δ pch2Δ*** .

|  | **Wild type** | ***mlh3Δ*** | ***pch2Δ*** | ***mlh3Δ pch2Δ*** |
| --- | --- | --- | --- | --- |
| Observed ratios | 1340:2506:1267 | 242:461:227 | 481:862:466 | 472:803:405 |
| Expected ratios | 1278.25:2556.5:1278.25 | 232.5:465:232.5 | 452.25:904.5:452.25 | 420:840:420 |
| *P* value | 0.13 | 0.76 | 0.12 | 0.014 |

The Chi square test was performed to analyse significant difference between expected and observed ratios of 2, 3 or 4 strand crossovers in adjacent genetic intervals.
