# Supplementary material for: Modulating Crossover Frequency and Interference for Obligate Crossovers in Saccharomyces cerevisiae Meiosis
Source: G3 (Bethesda). 2017 Mar 17;7(5):1511–24. doi: 10.1534/g3.117.040071 (PMC5427503; doi:10.1534/g3.117.040071)
Supplement: Supplementary file 19 [file 1511TableS10.docx]

**Table S10 Proportion of Class I and Class II crossovers inferred from the two pathway analysis of interference.**

|  | Class I crossovers | Class II crossovers | Alpha |
| --- | --- | --- | --- |
|  |  |  |  |
| Wild type | 0.59 | 0.41 | 3.1 |
| *mlh3Δ* | 0.34 | 0.66 | 2.8 |
| *pch2Δ* | 0.22 | 0.78 | 6.3 |
| *mlh3Δ pch2Δ* | 0.5 | 0.5 | 1 |

Class I are interfering crossovers and Class II are non-interfering crossovers. Alpha is the

shape parameter for Class I crossovers.
