# Supplementary figures and images for: Modulating Crossover Frequency and Interference for Obligate Crossovers in Saccharomyces cerevisiae Meiosis
Source: G3 (Bethesda). 2017 Mar 17;7(5):1511–24. doi: 10.1534/g3.117.040071 (PMC5427503; doi:10.1534/g3.117.040071)

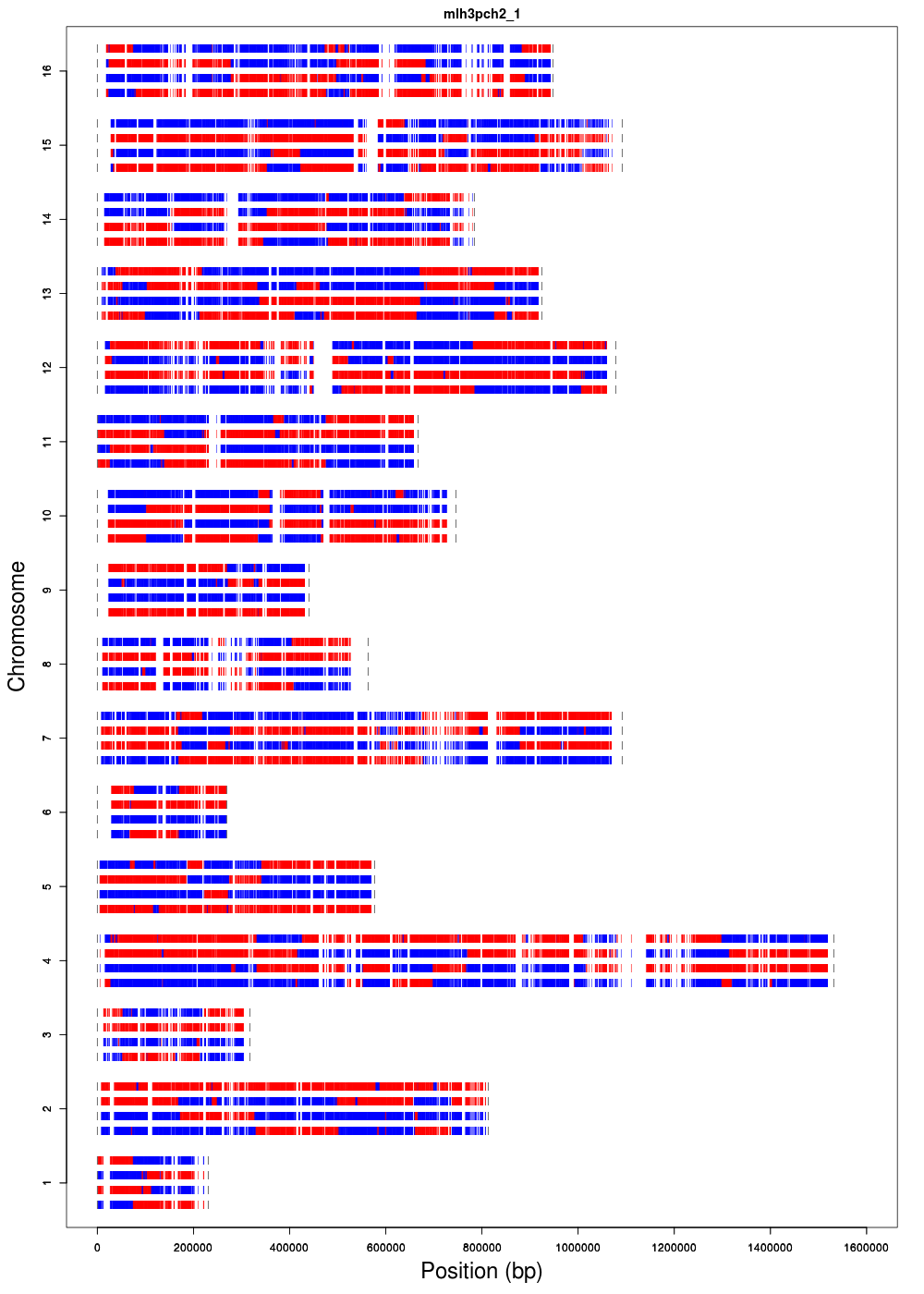

Supplement: Supplementary file 20 [file 1511FileS1.zip › S1 File/mlh3pch2_1.tiff]

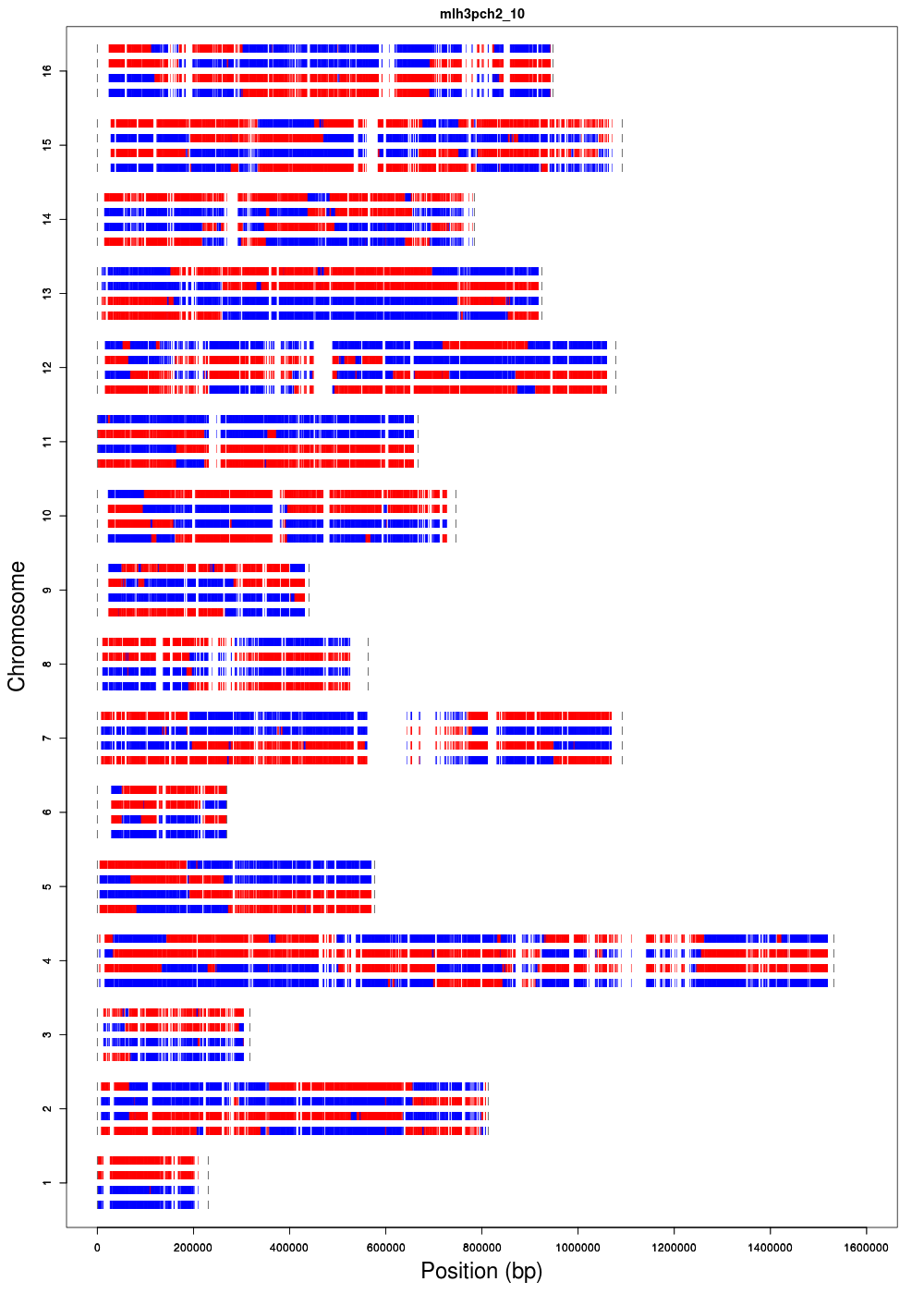

Supplement: Supplementary file 20 [file 1511FileS1.zip › S1 File/mlh3pch2_10.tiff]

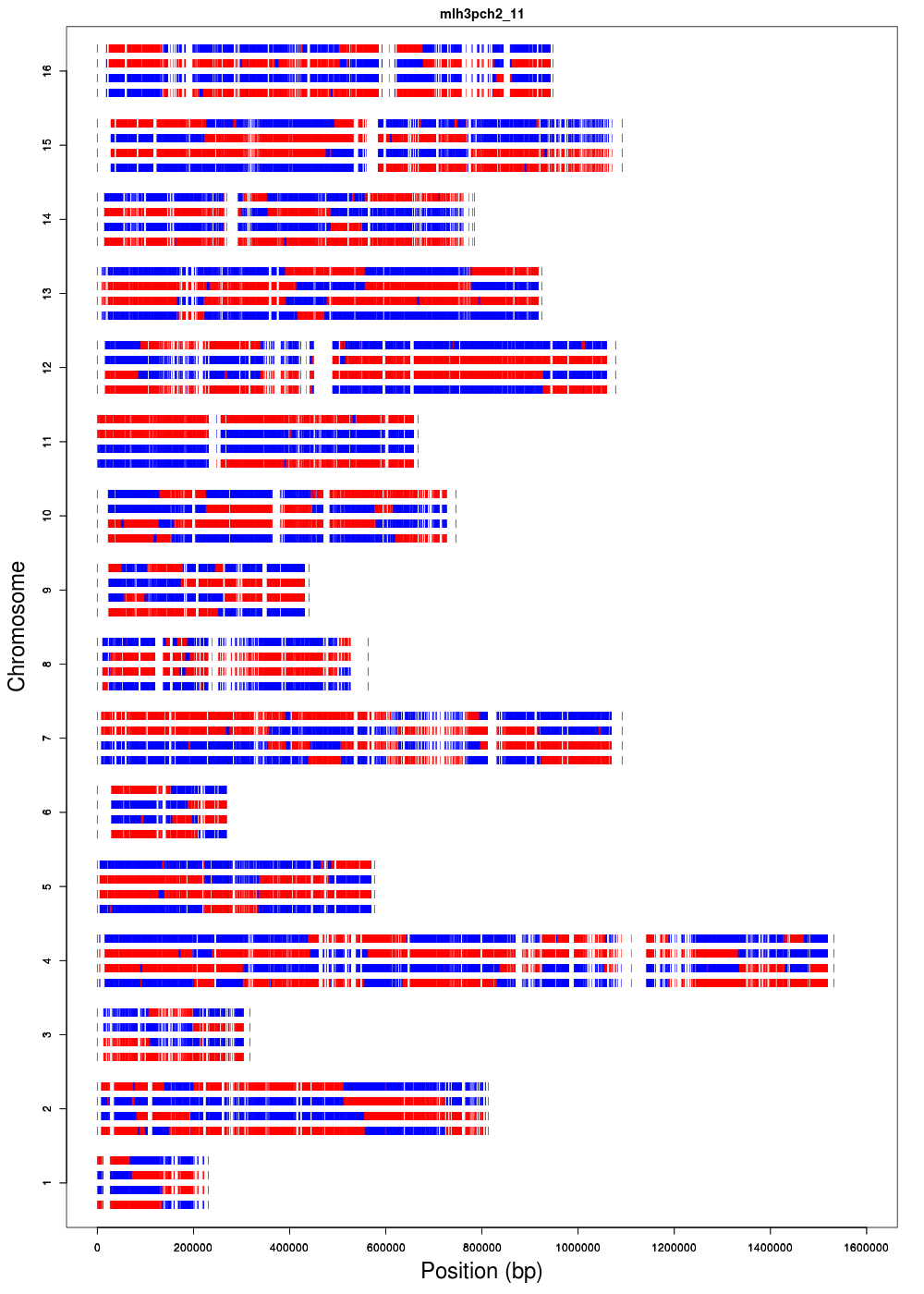

Supplement: Supplementary file 20 [file 1511FileS1.zip › S1 File/mlh3pch2_11.tiff]

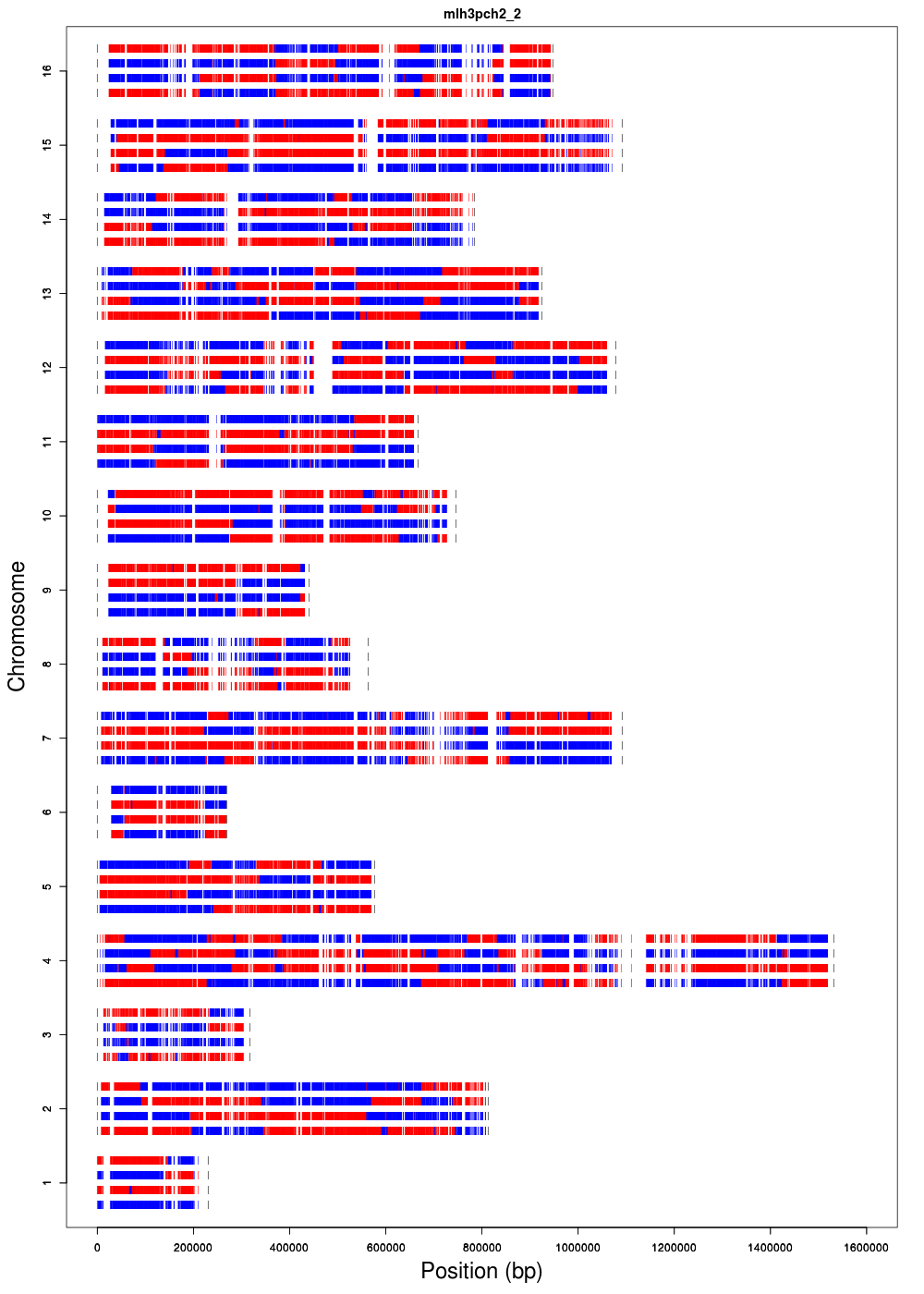

Supplement: Supplementary file 20 [file 1511FileS1.zip › S1 File/mlh3pch2_2.tiff]

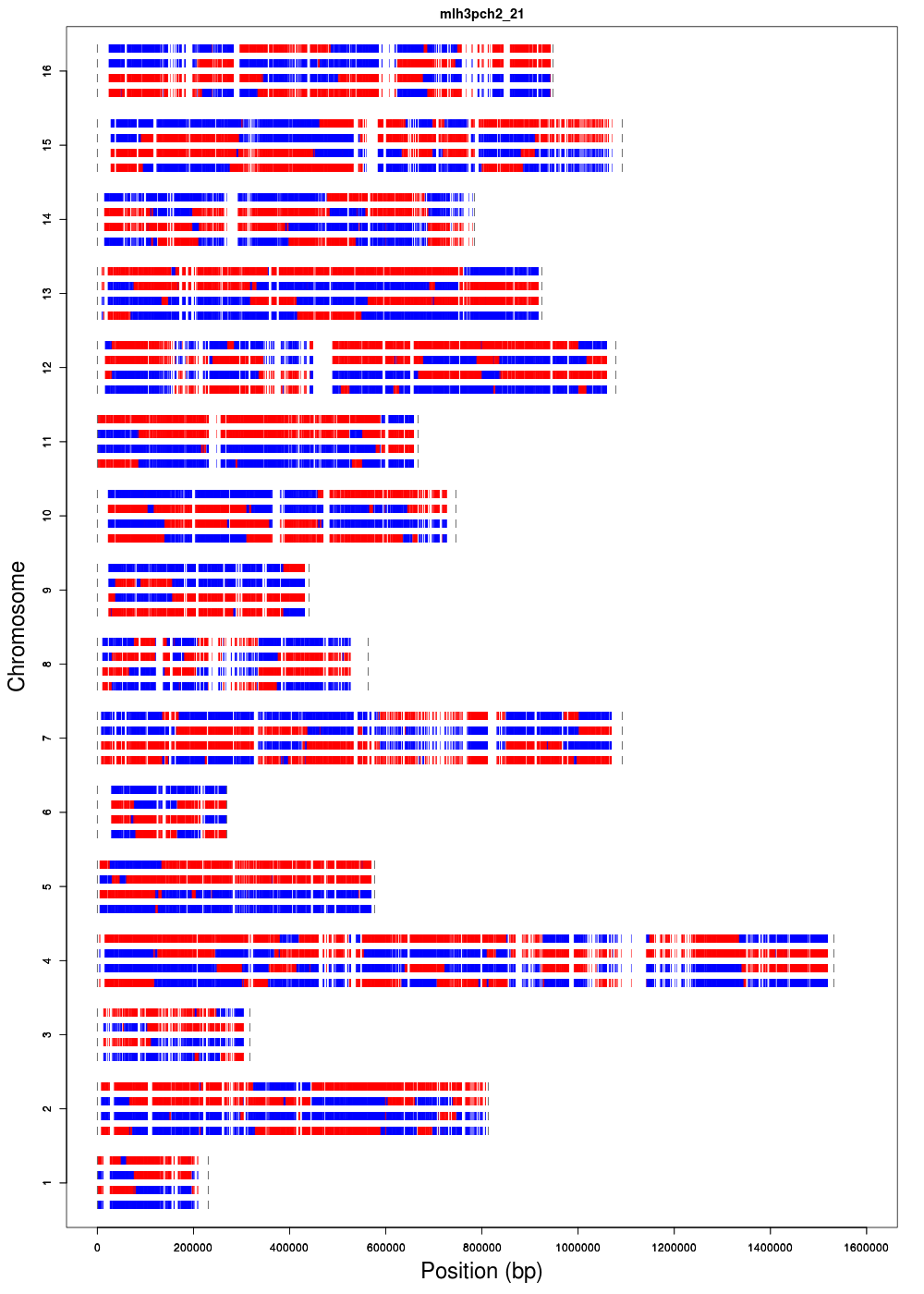

Supplement: Supplementary file 20 [file 1511FileS1.zip › S1 File/mlh3pch2_21.tiff]

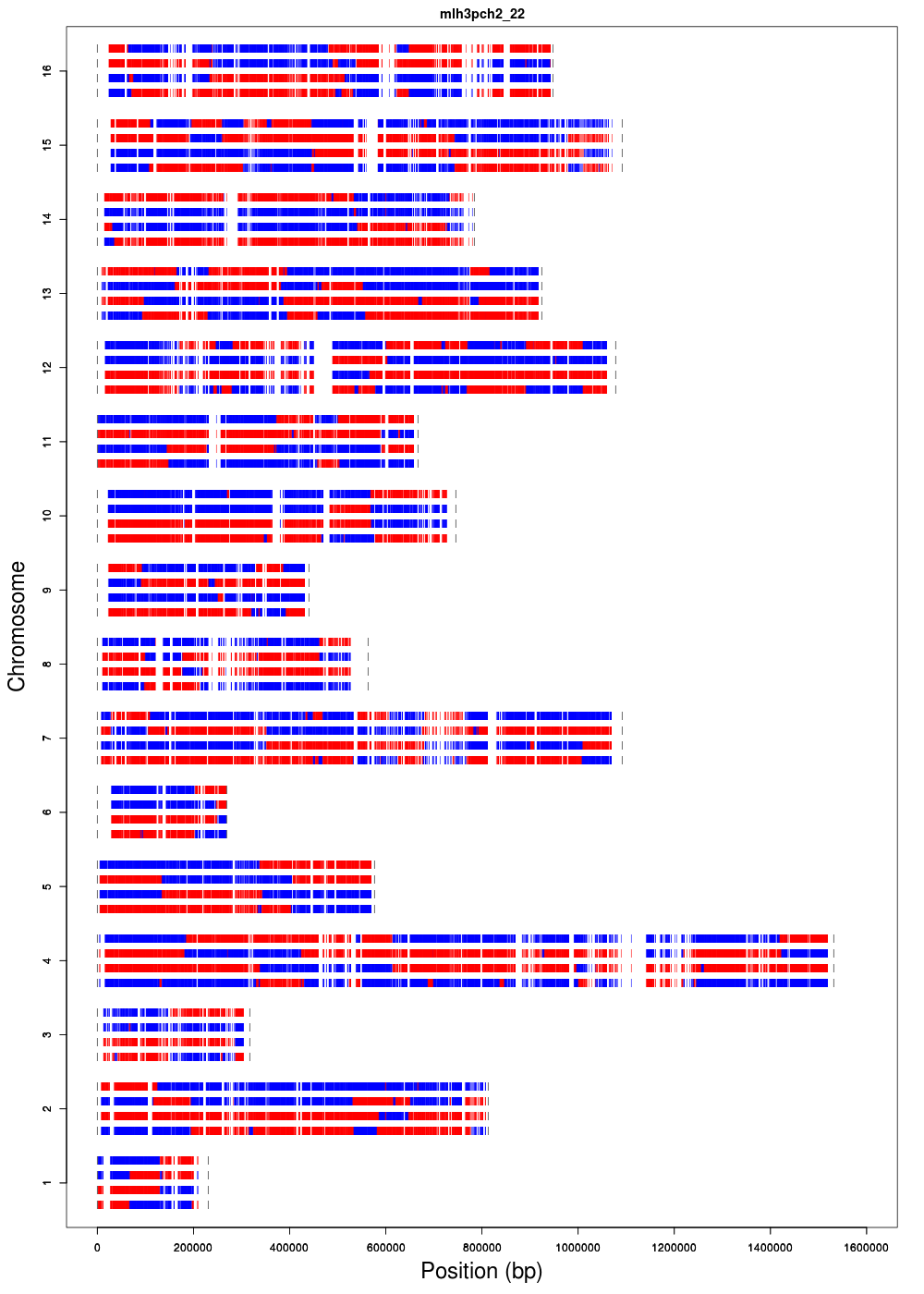

Supplement: Supplementary file 20 [file 1511FileS1.zip › S1 File/mlh3pch2_22.tiff]

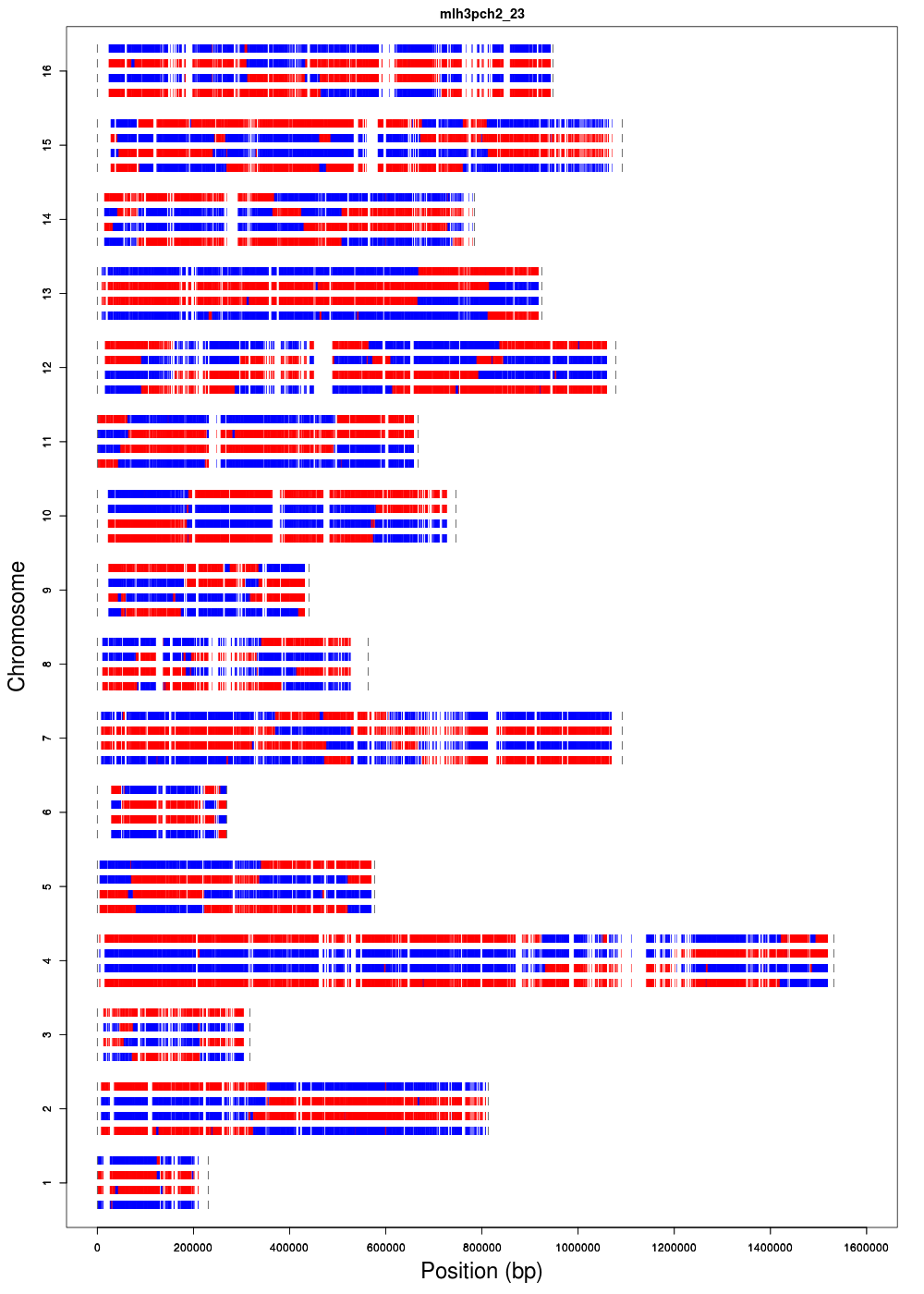

Supplement: Supplementary file 20 [file 1511FileS1.zip › S1 File/mlh3pch2_23.tiff]

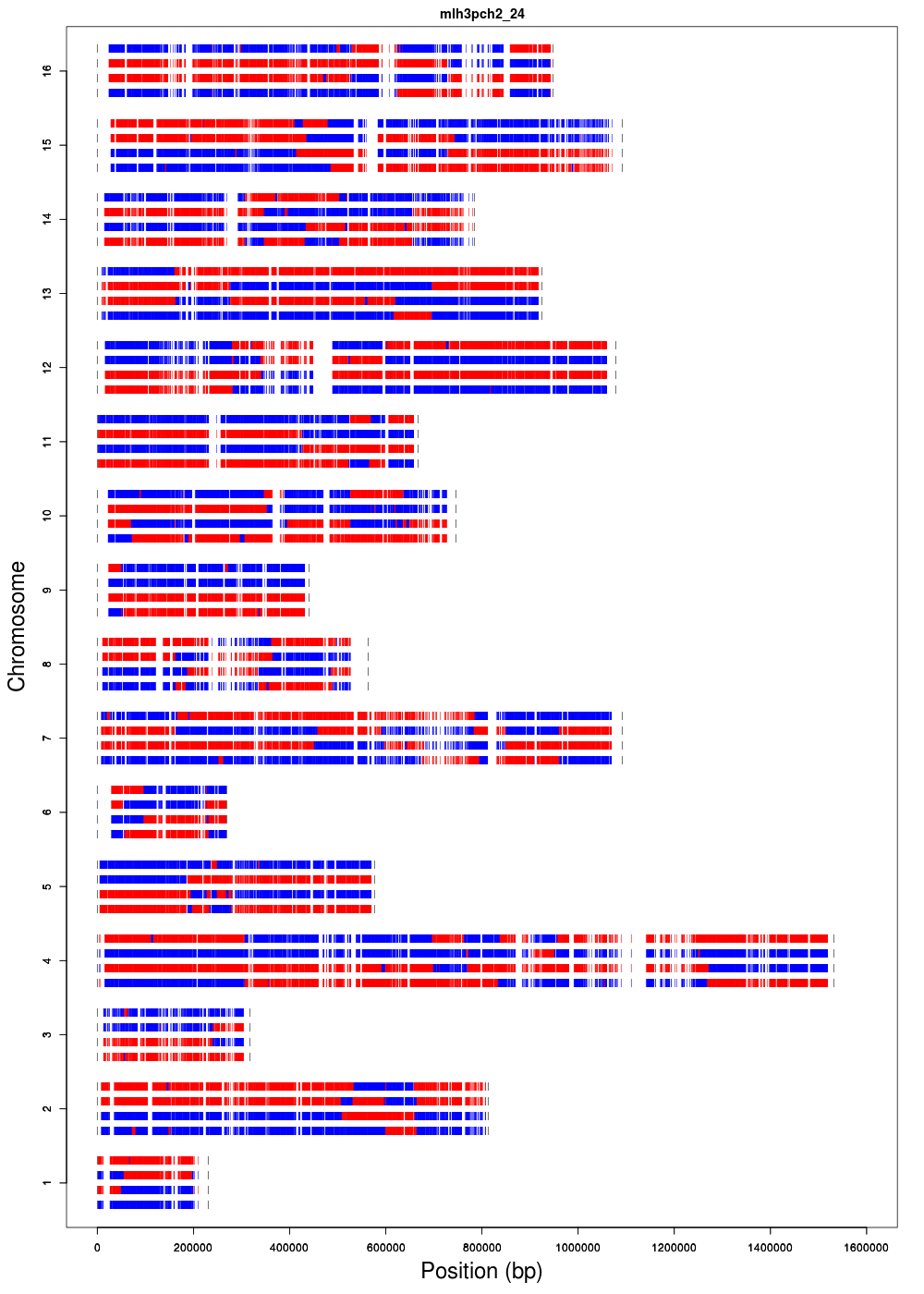

Supplement: Supplementary file 20 [file 1511FileS1.zip › S1 File/mlh3pch2_24.tiff]

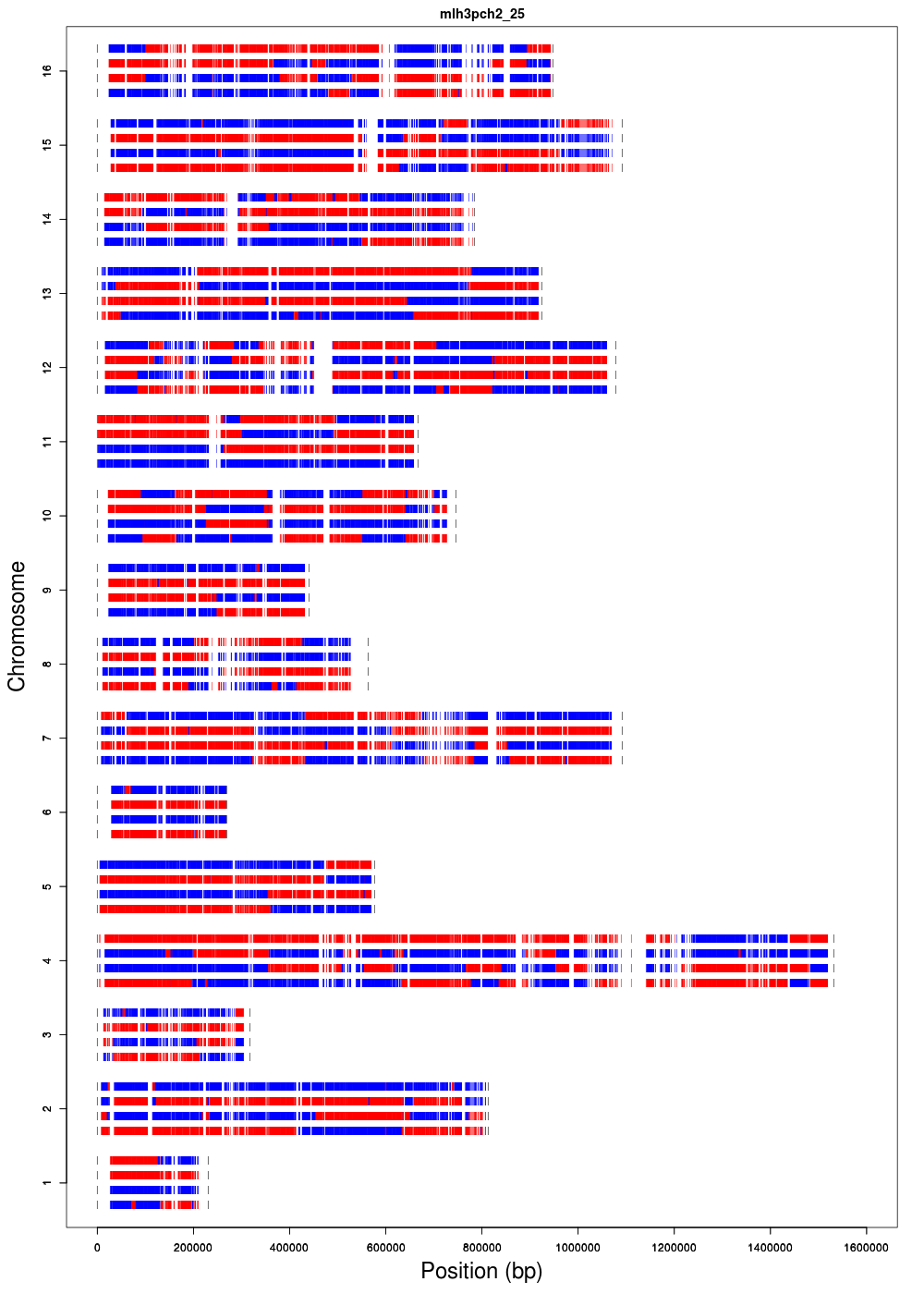

Supplement: Supplementary file 20 [file 1511FileS1.zip › S1 File/mlh3pch2_25.tiff]

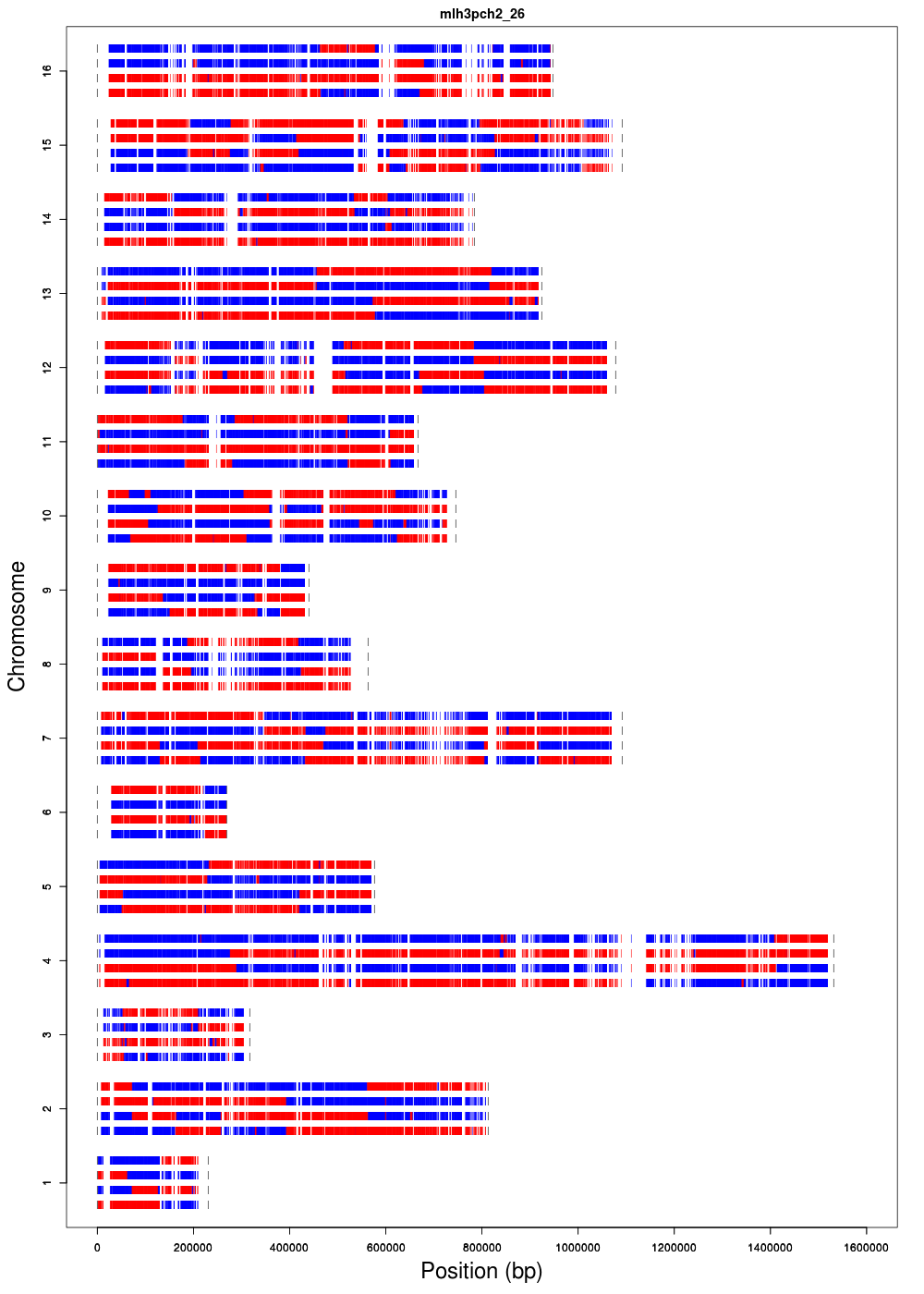

Supplement: Supplementary file 20 [file 1511FileS1.zip › S1 File/mlh3pch2_26.tiff]

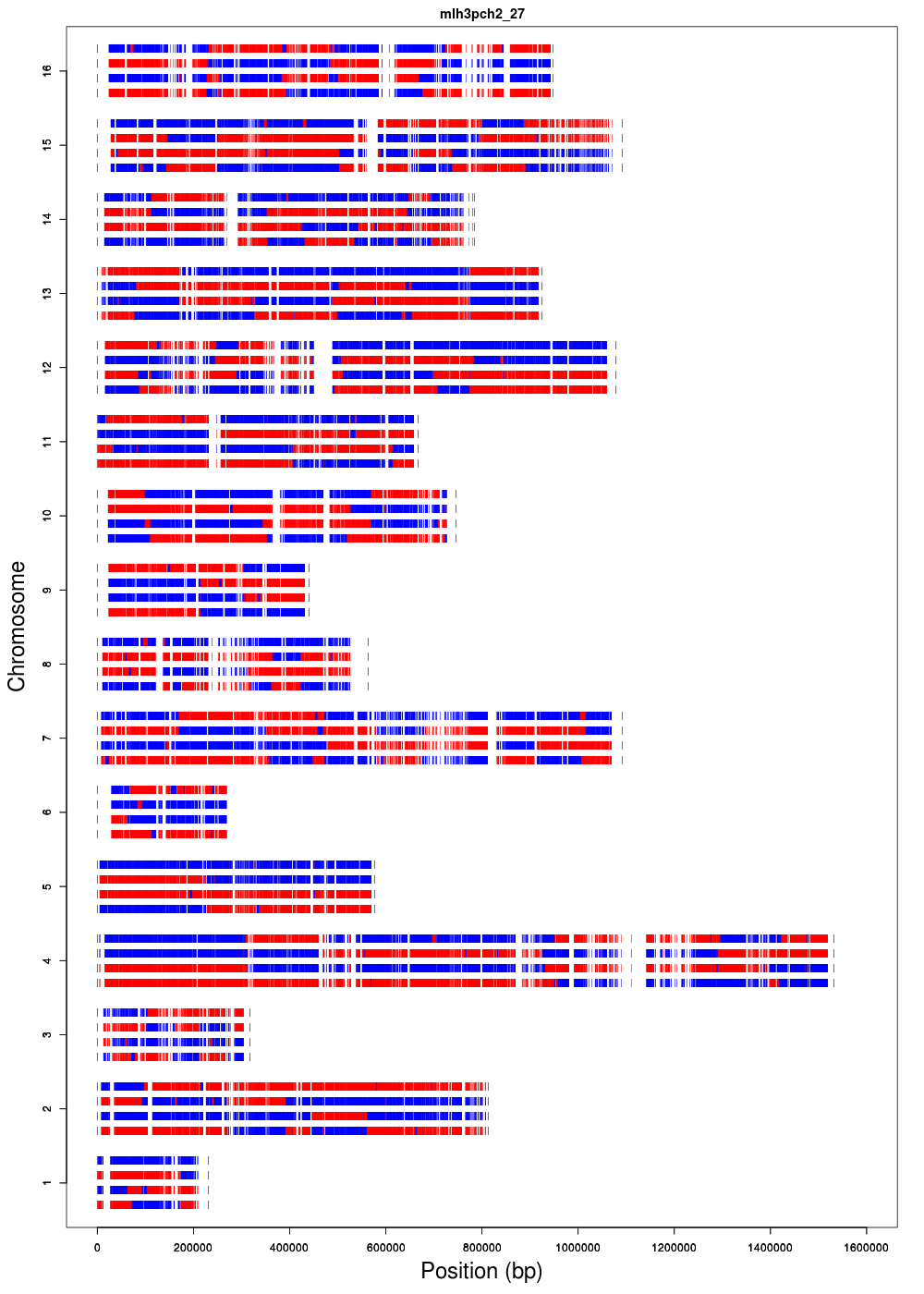

Supplement: Supplementary file 20 [file 1511FileS1.zip › S1 File/mlh3pch2_27.tiff]

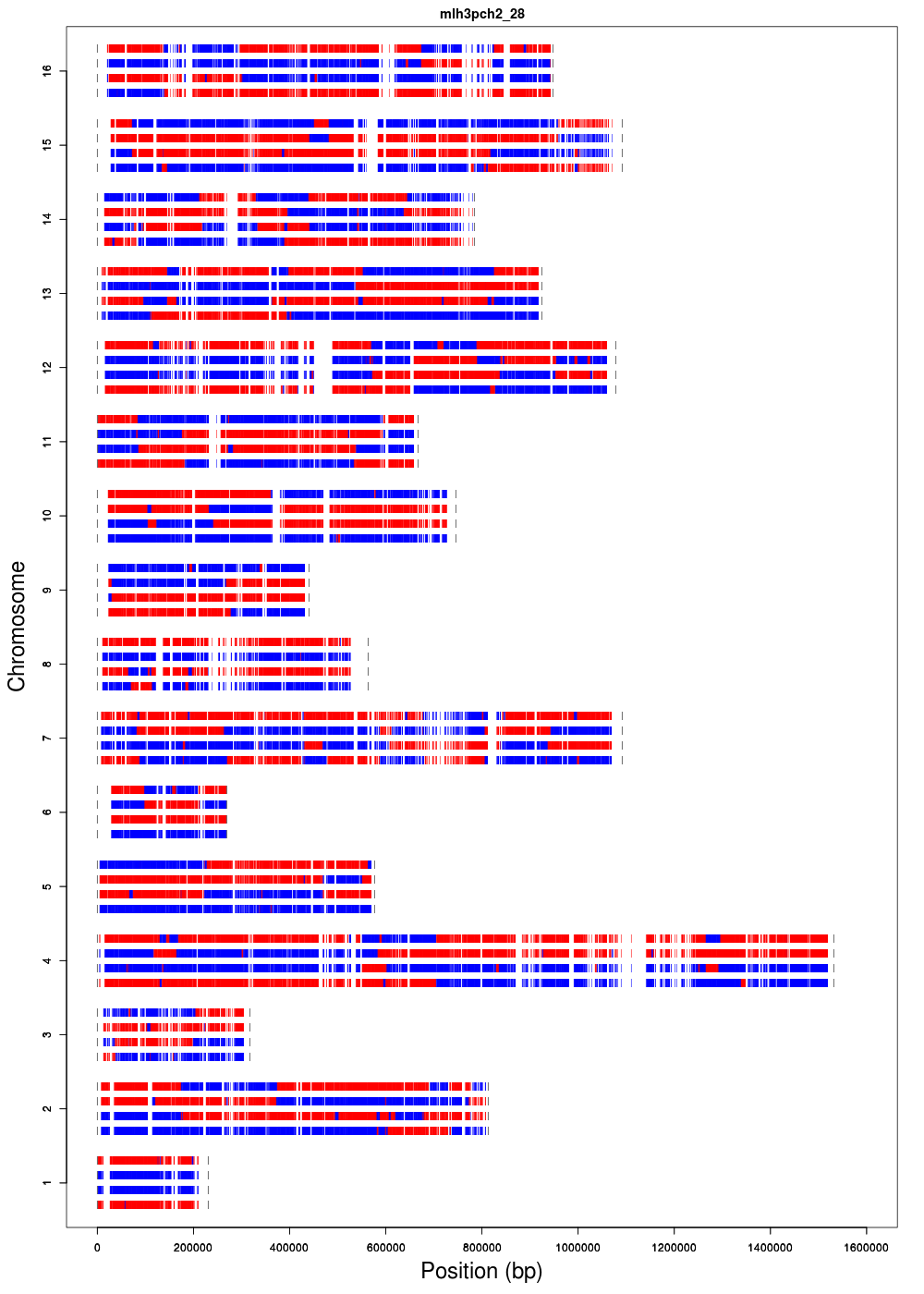

Supplement: Supplementary file 20 [file 1511FileS1.zip › S1 File/mlh3pch2_28.tiff]

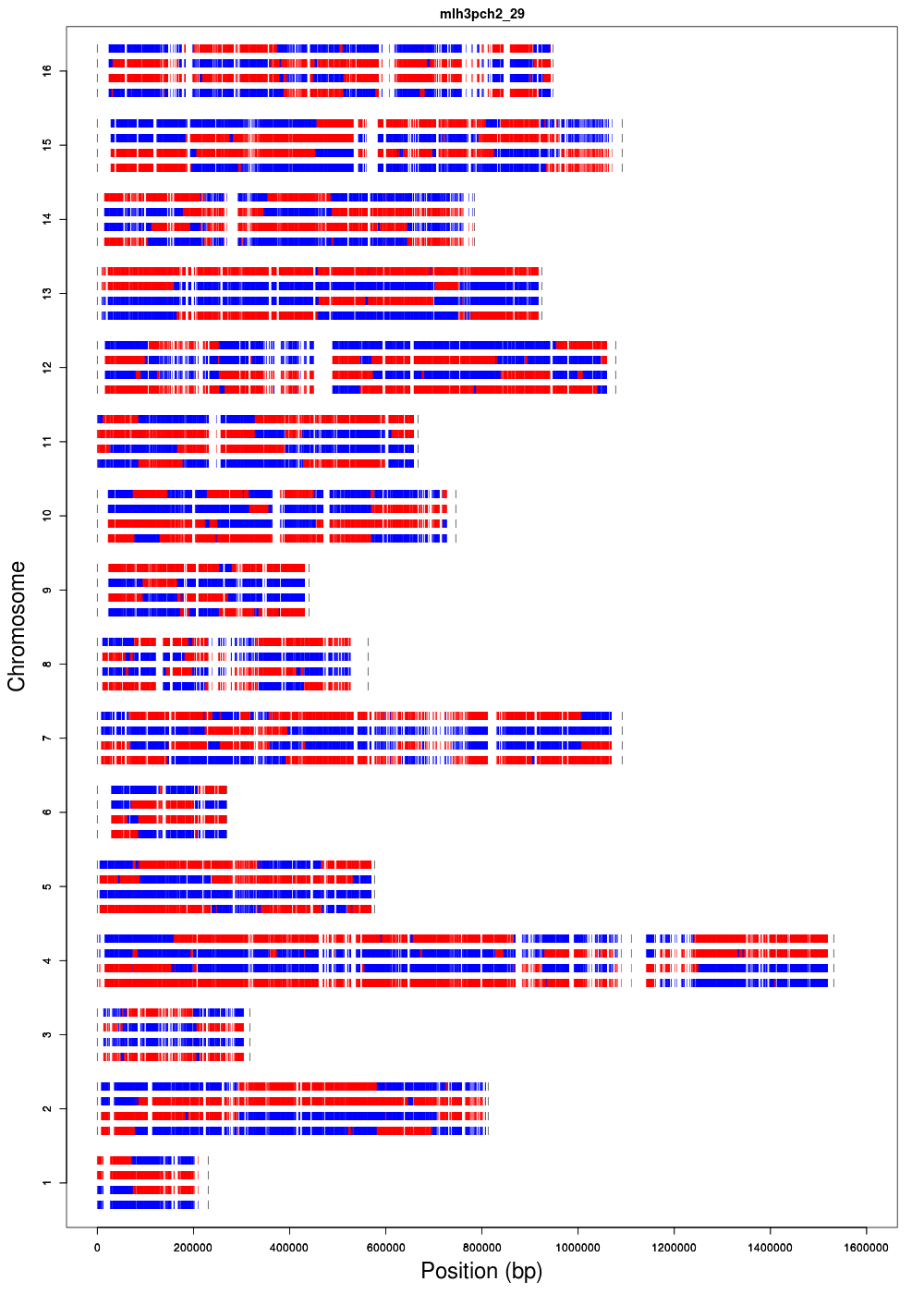

Supplement: Supplementary file 20 [file 1511FileS1.zip › S1 File/mlh3pch2_29.tiff]

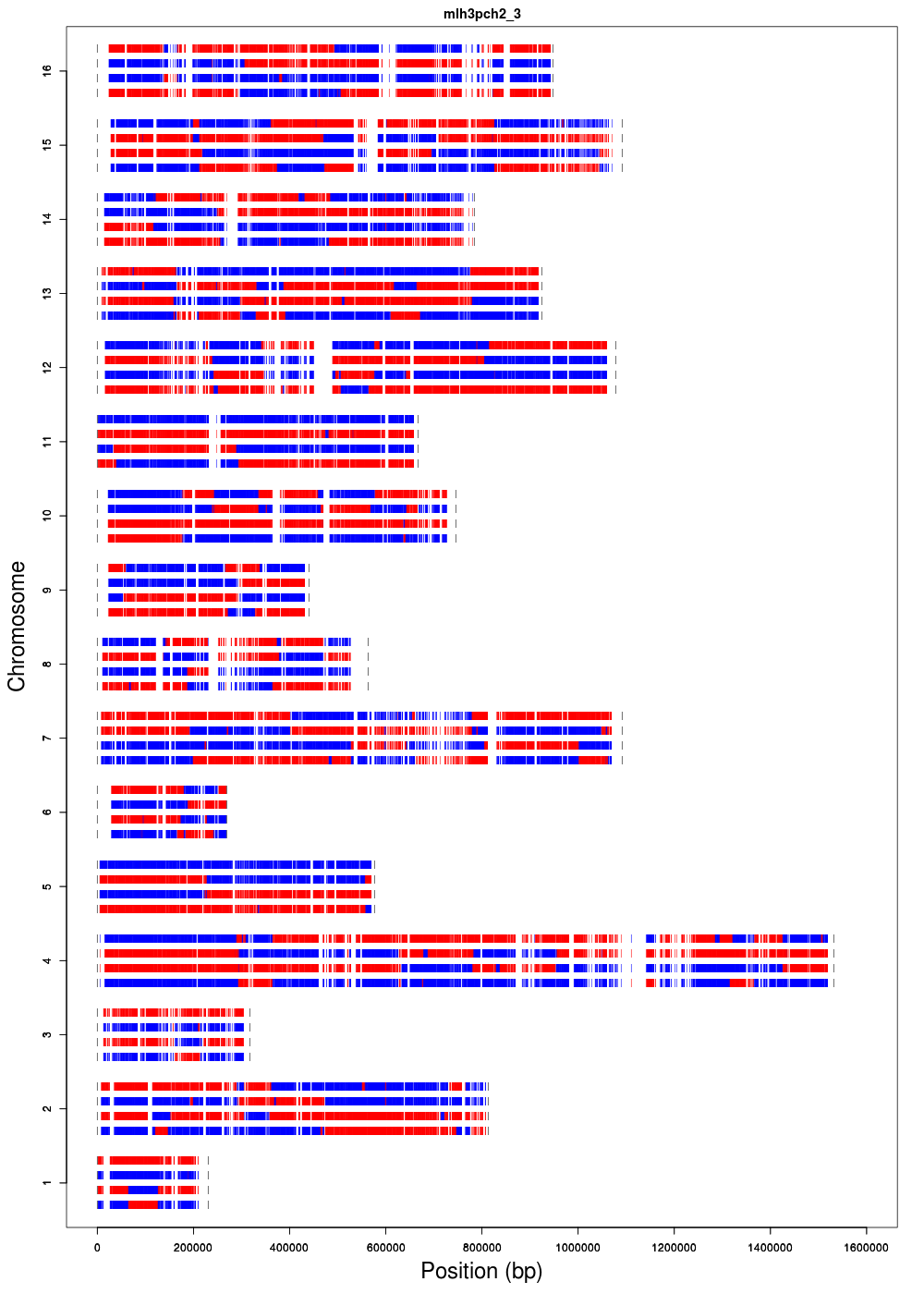

Supplement: Supplementary file 20 [file 1511FileS1.zip › S1 File/mlh3pch2_3.tiff]

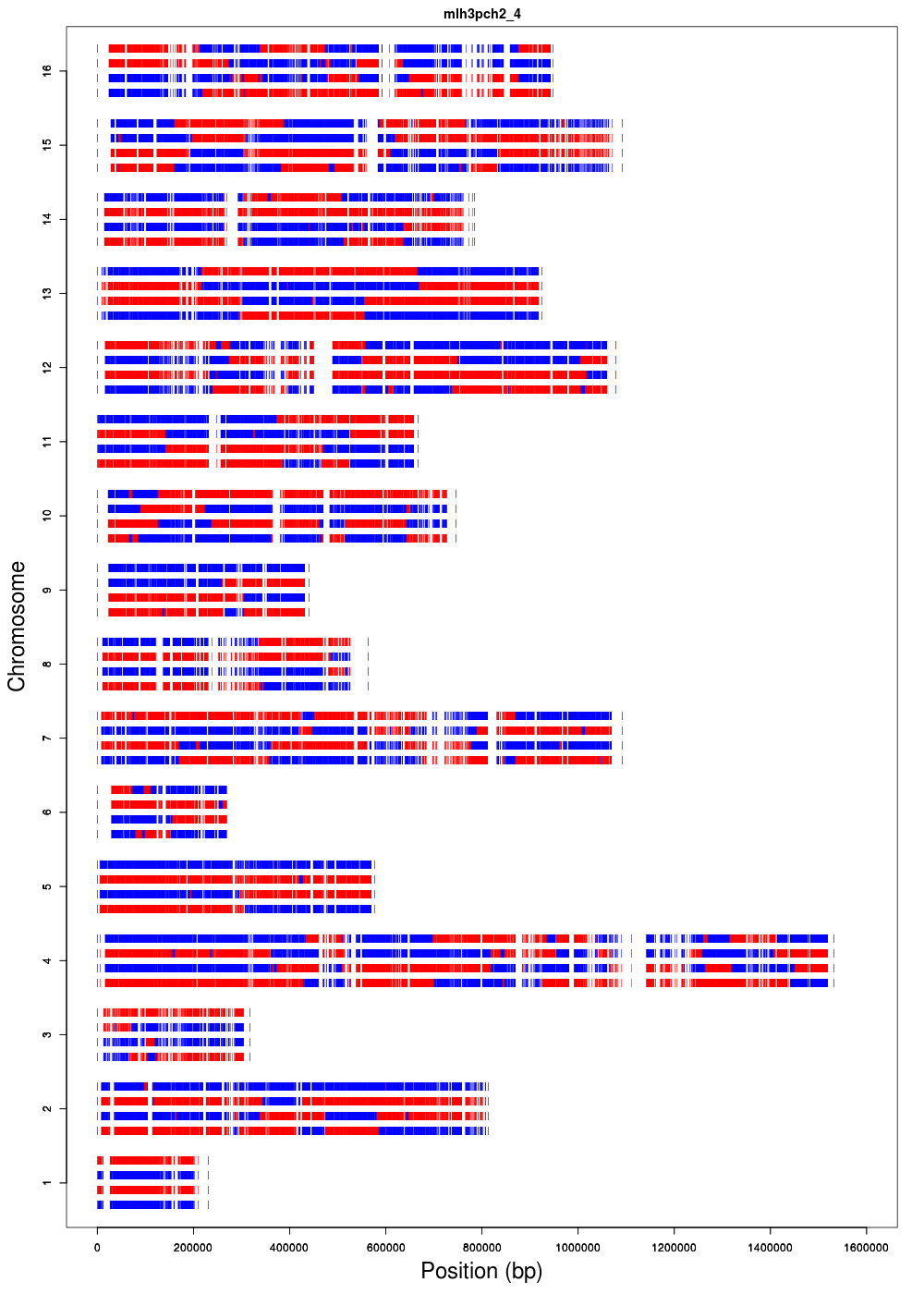

Supplement: Supplementary file 20 [file 1511FileS1.zip › S1 File/mlh3pch2_4.tiff]

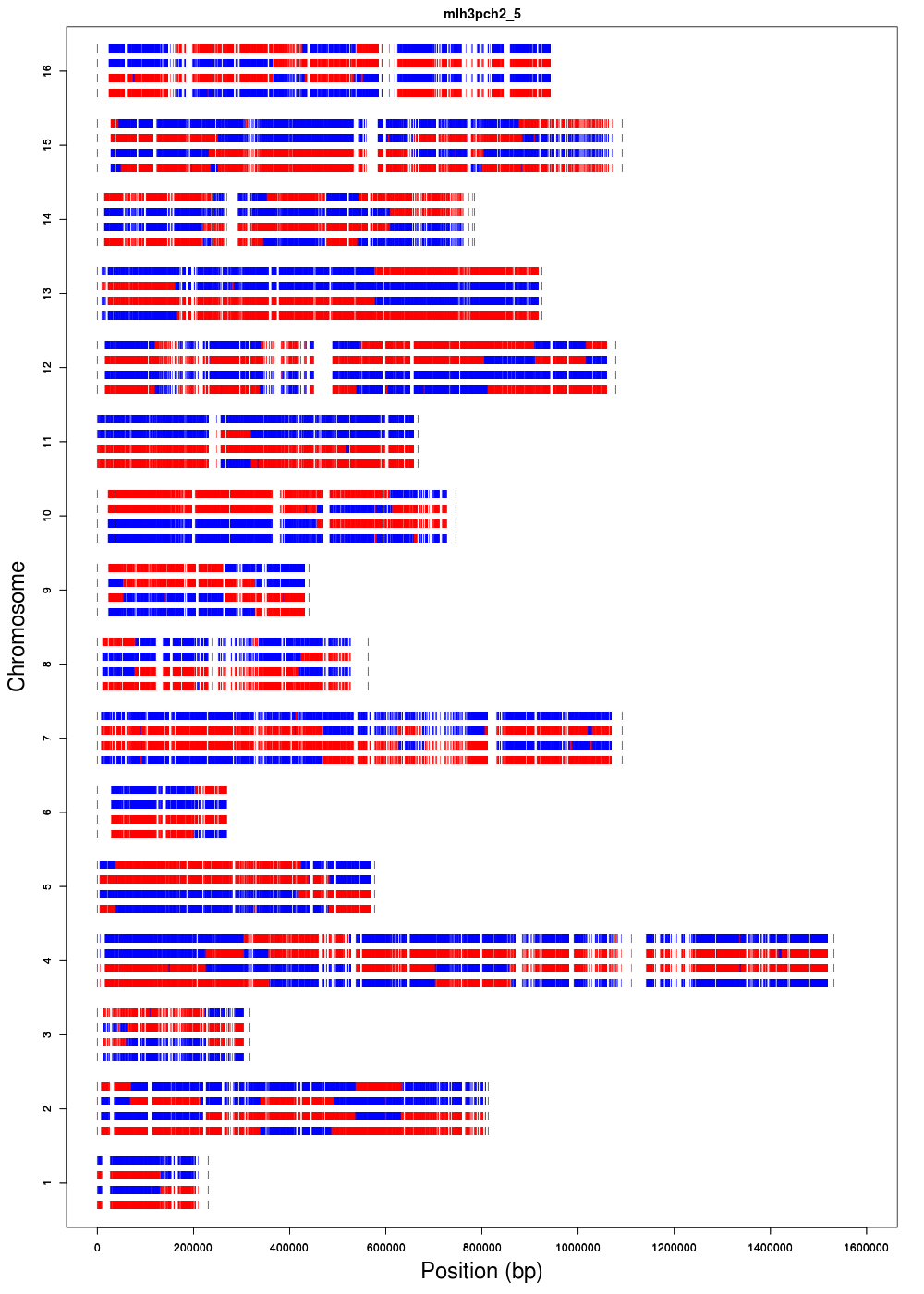

Supplement: Supplementary file 20 [file 1511FileS1.zip › S1 File/mlh3pch2_5.tiff]

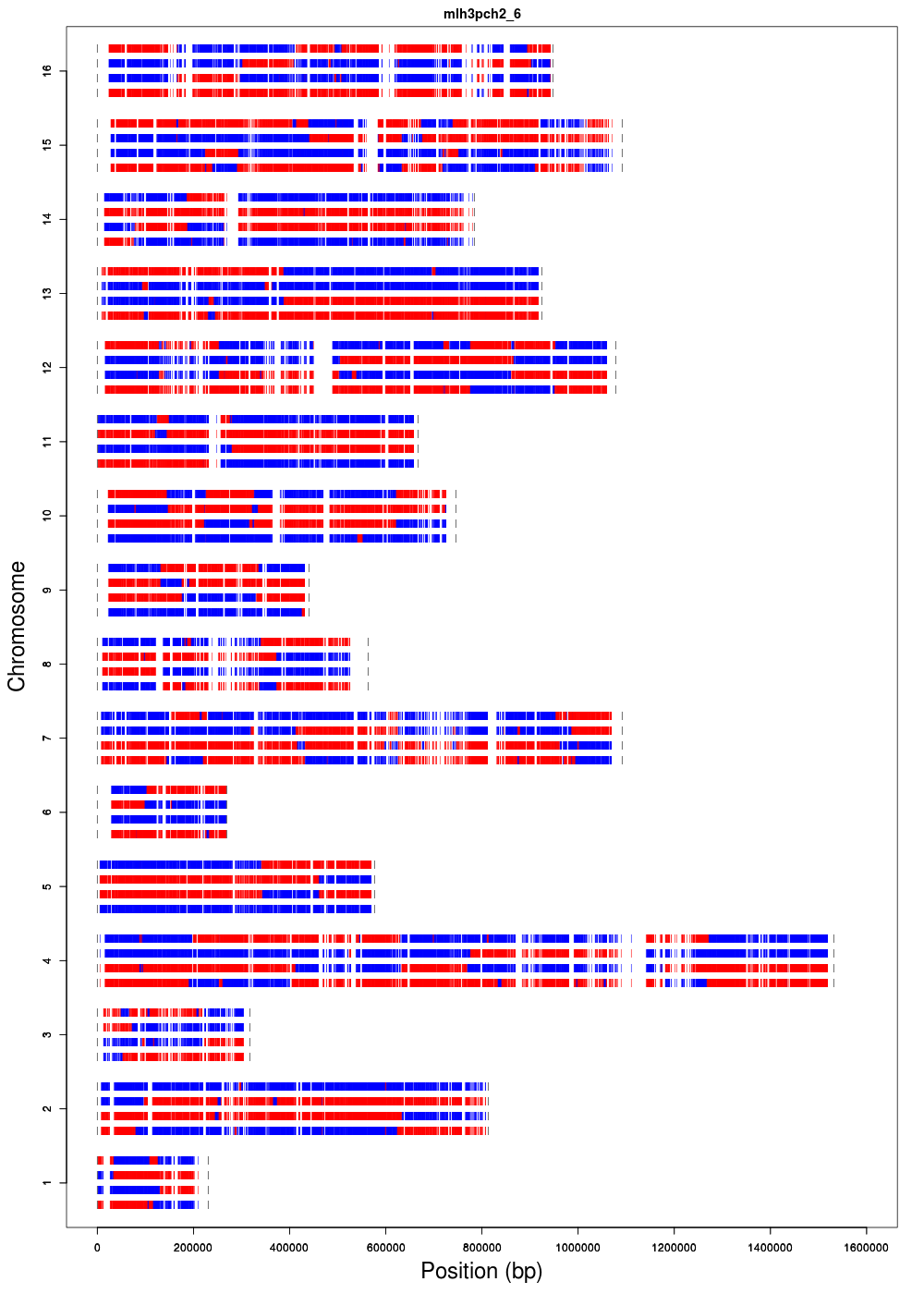

Supplement: Supplementary file 20 [file 1511FileS1.zip › S1 File/mlh3pch2_6.tiff]

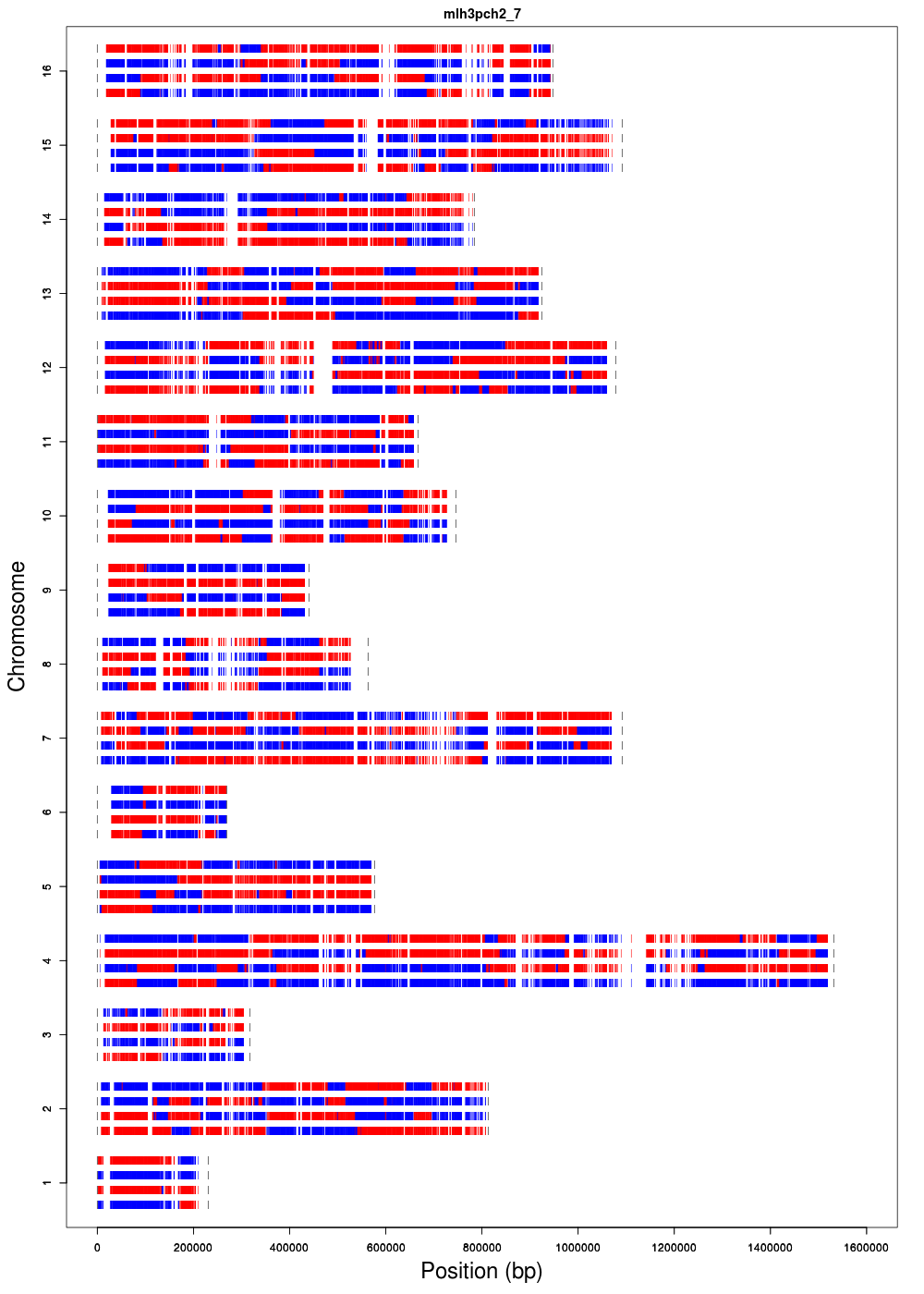

Supplement: Supplementary file 20 [file 1511FileS1.zip › S1 File/mlh3pch2_7.tiff]

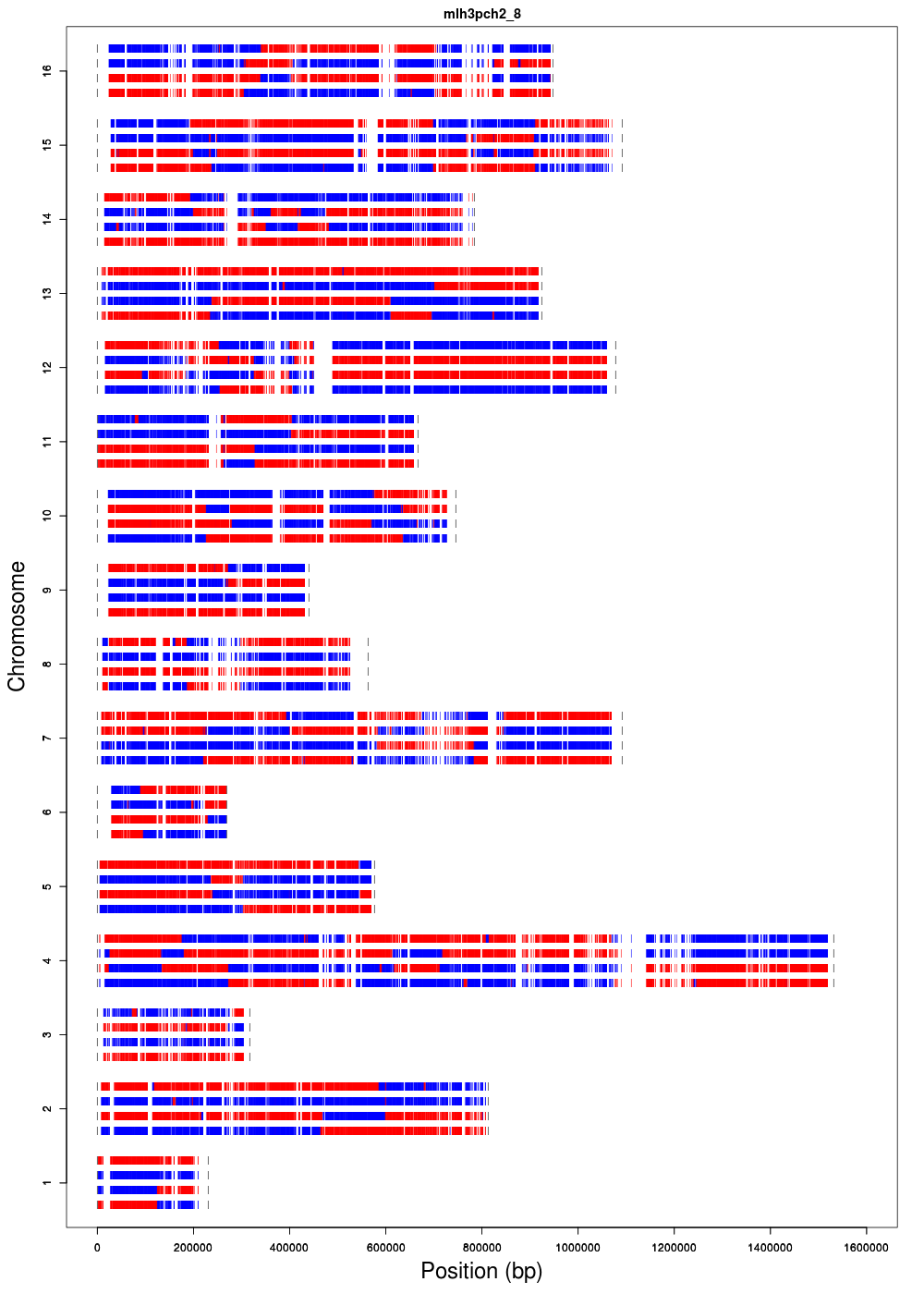

Supplement: Supplementary file 20 [file 1511FileS1.zip › S1 File/mlh3pch2_8.tiff]

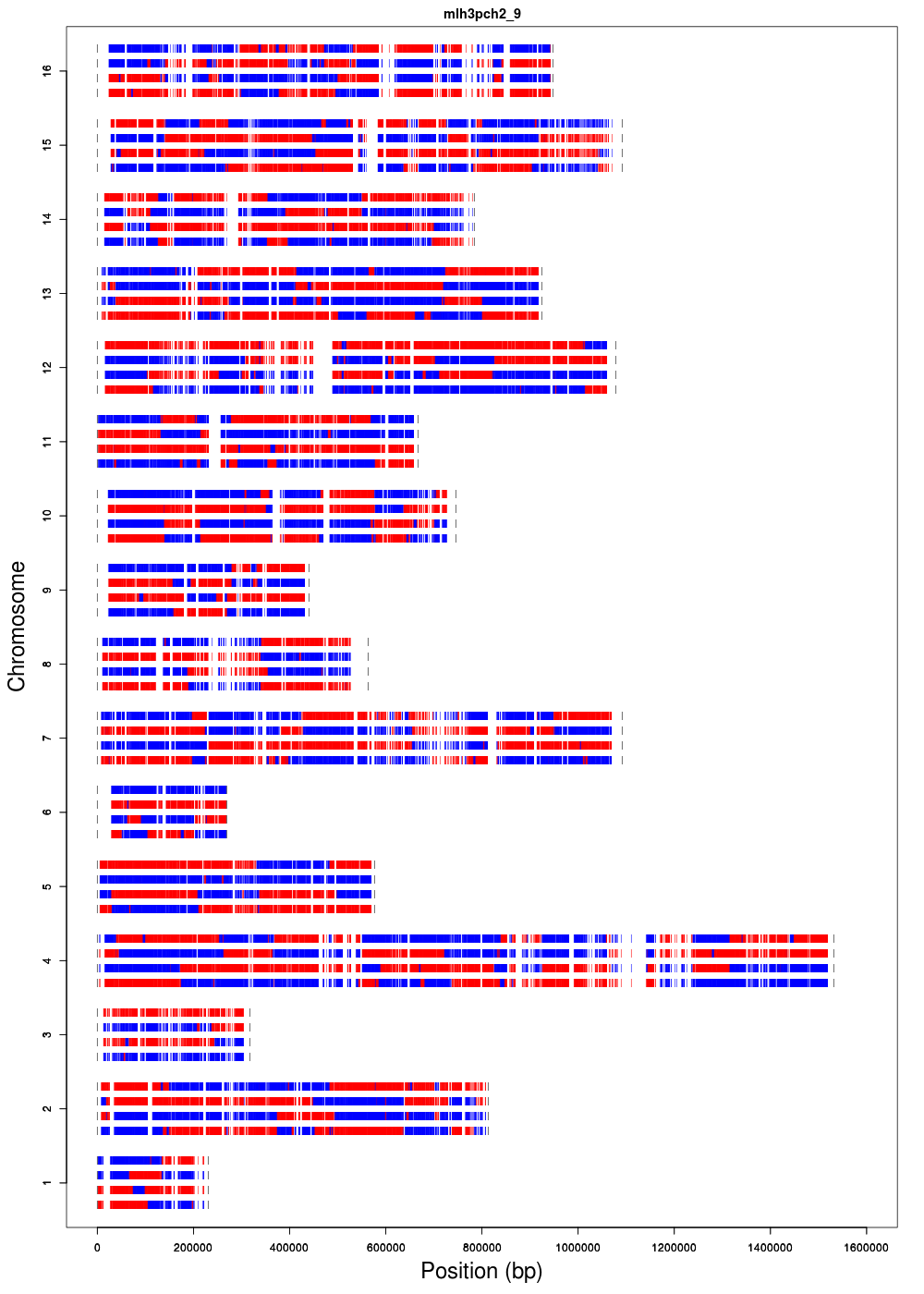

Supplement: Supplementary file 20 [file 1511FileS1.zip › S1 File/mlh3pch2_9.tiff]

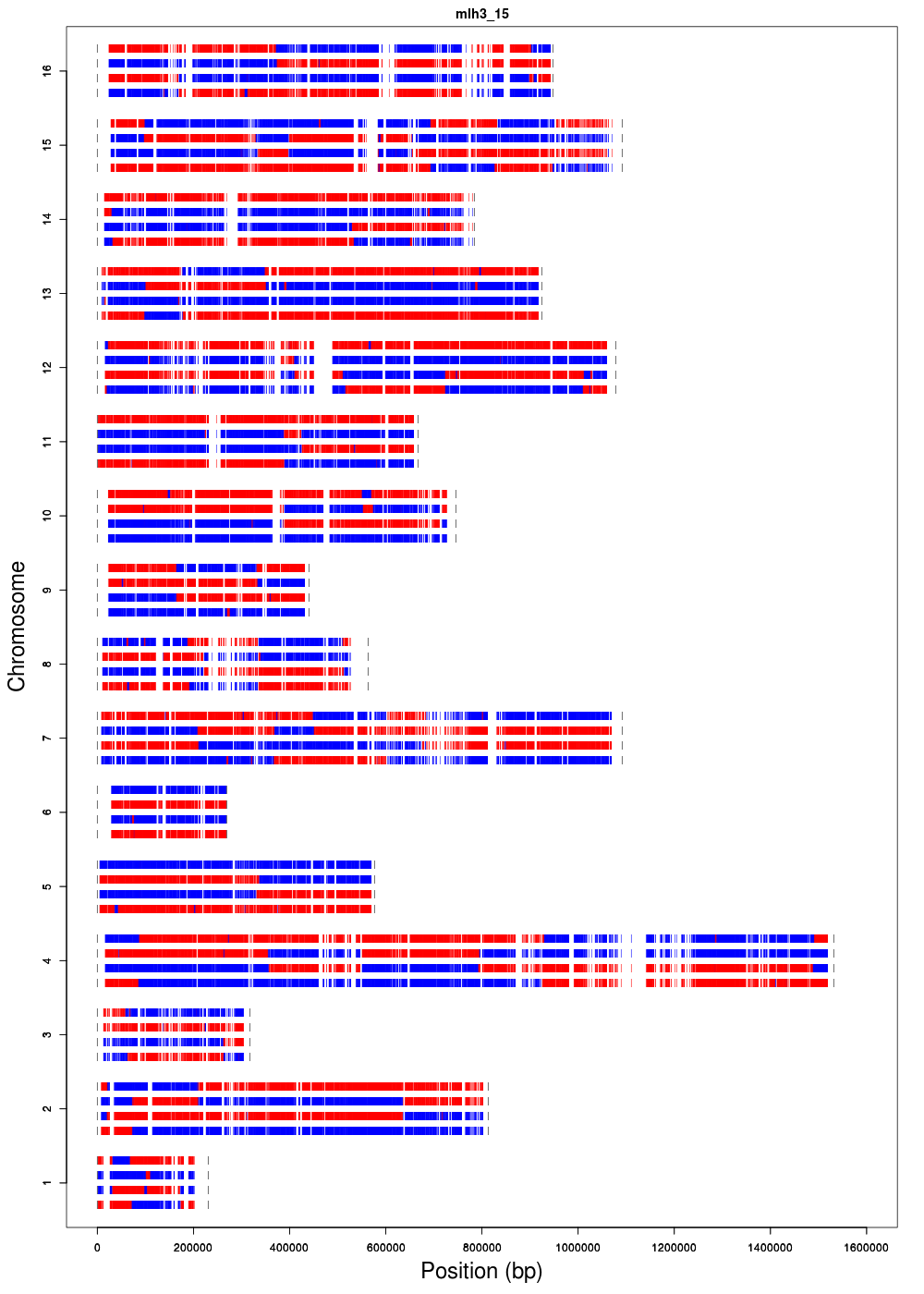

Supplement: Supplementary file 20 [file 1511FileS1.zip › S1 File/mlh3_15.tiff]

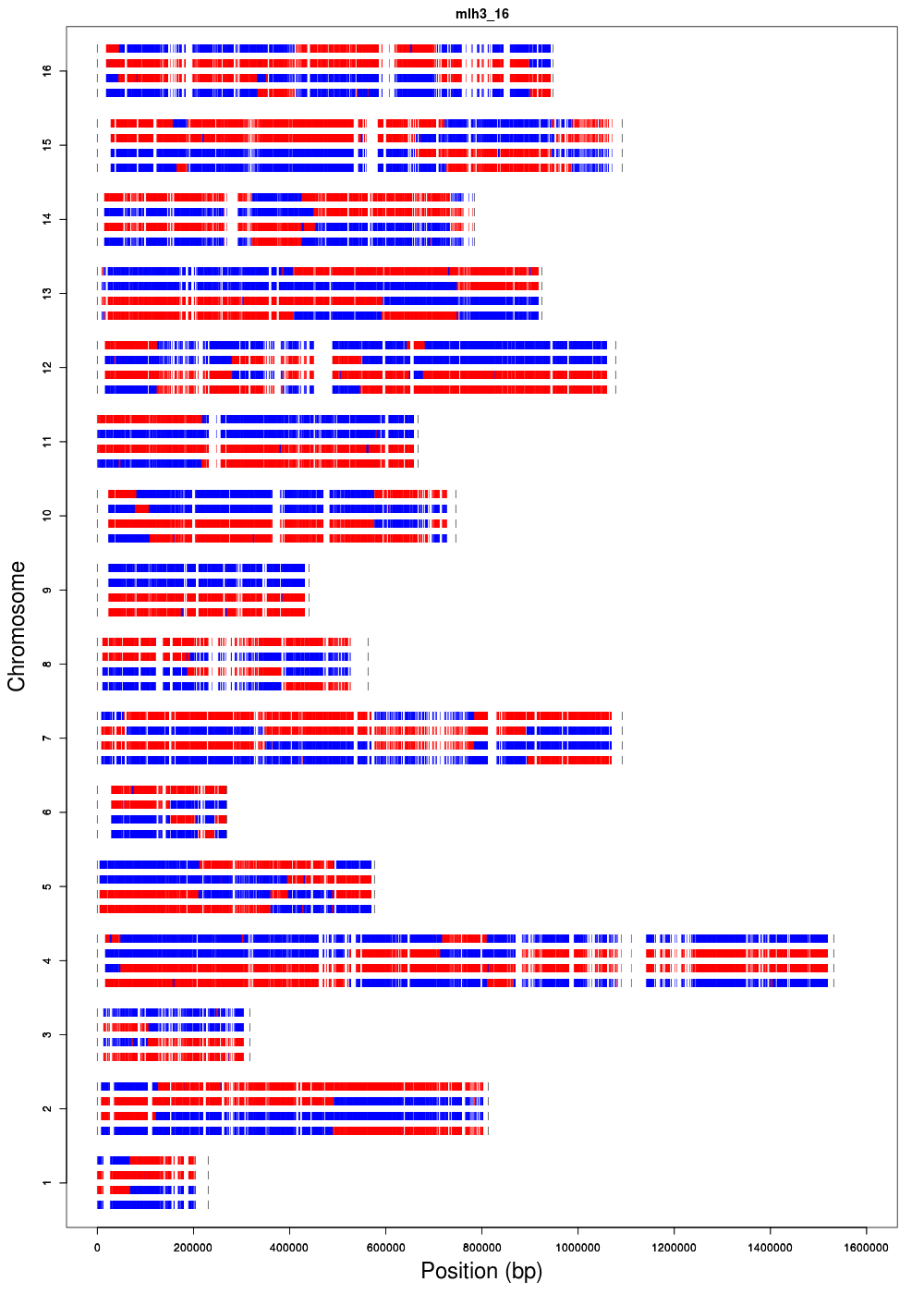

Supplement: Supplementary file 20 [file 1511FileS1.zip › S1 File/mlh3_16.tiff]

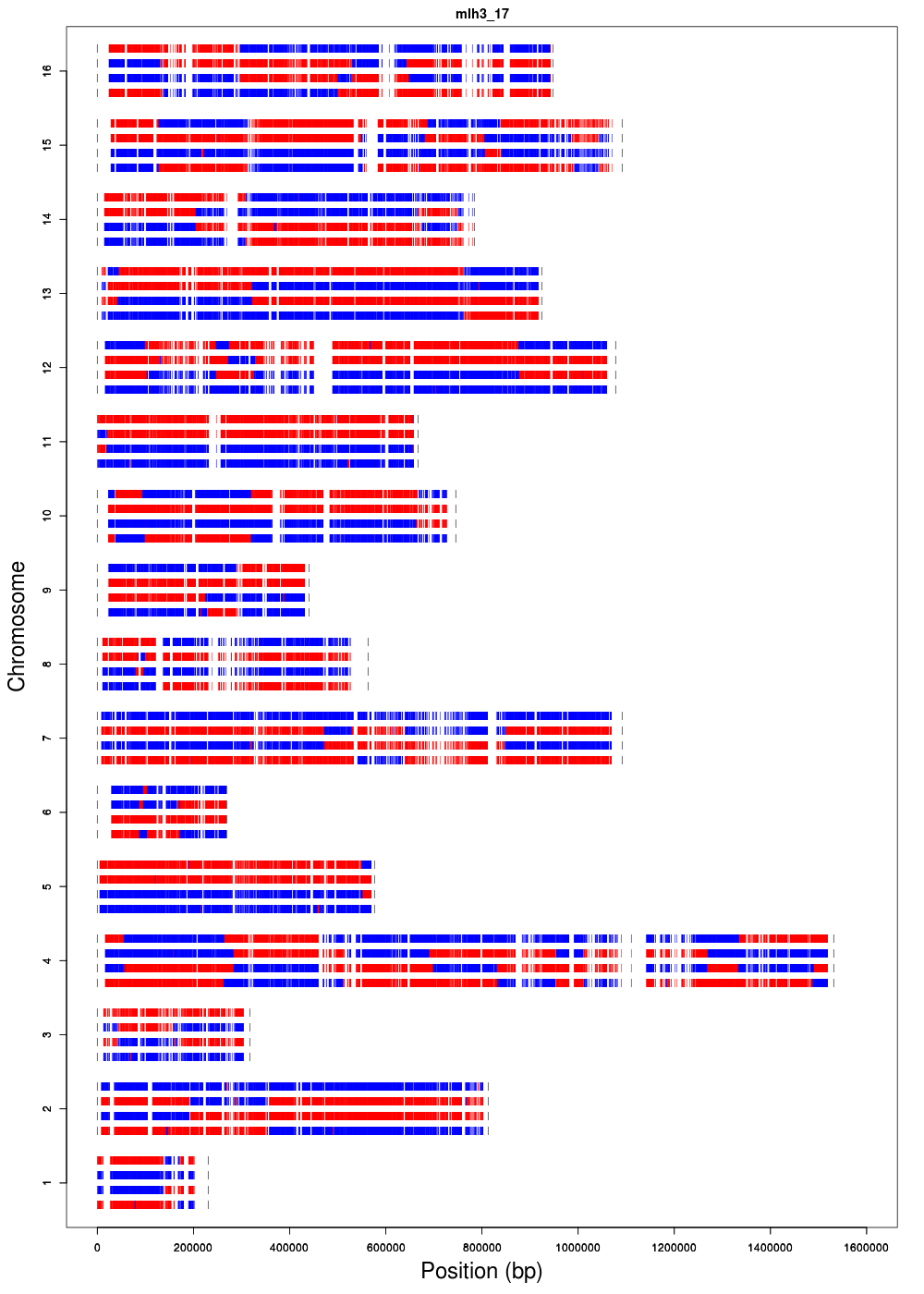

Supplement: Supplementary file 20 [file 1511FileS1.zip › S1 File/mlh3_17.tiff]

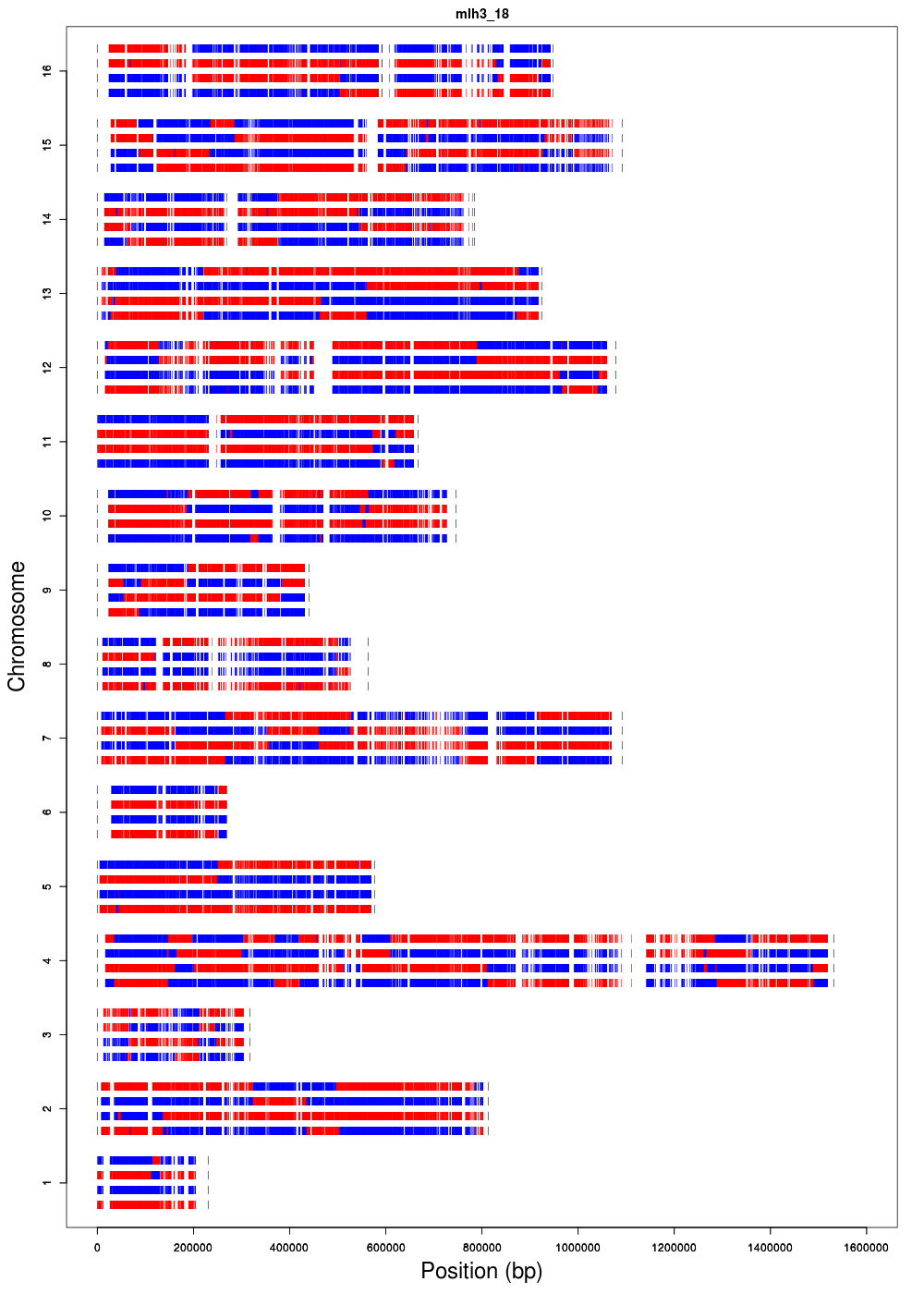

Supplement: Supplementary file 20 [file 1511FileS1.zip › S1 File/mlh3_18.tiff]

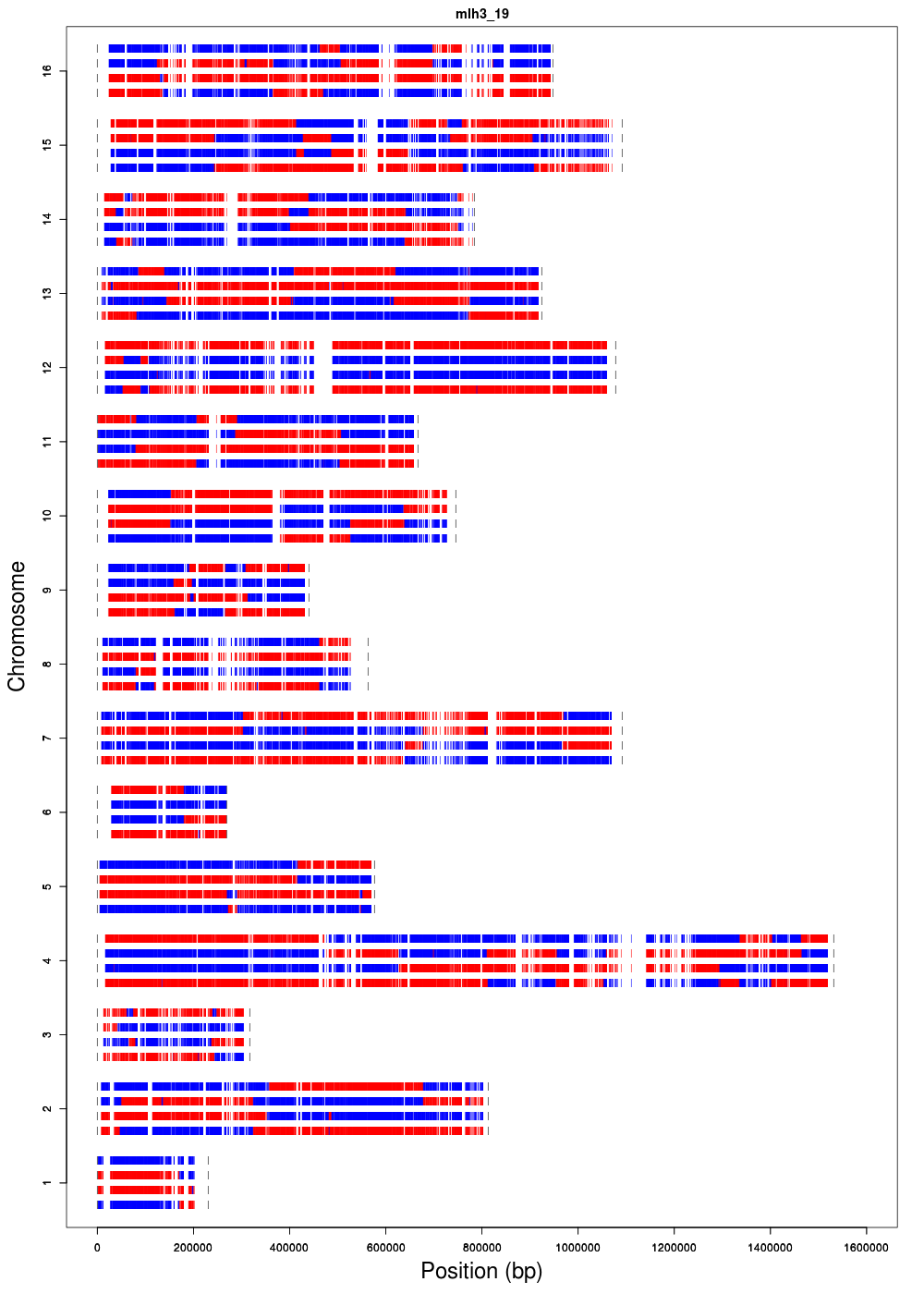

Supplement: Supplementary file 20 [file 1511FileS1.zip › S1 File/mlh3_19.tiff]

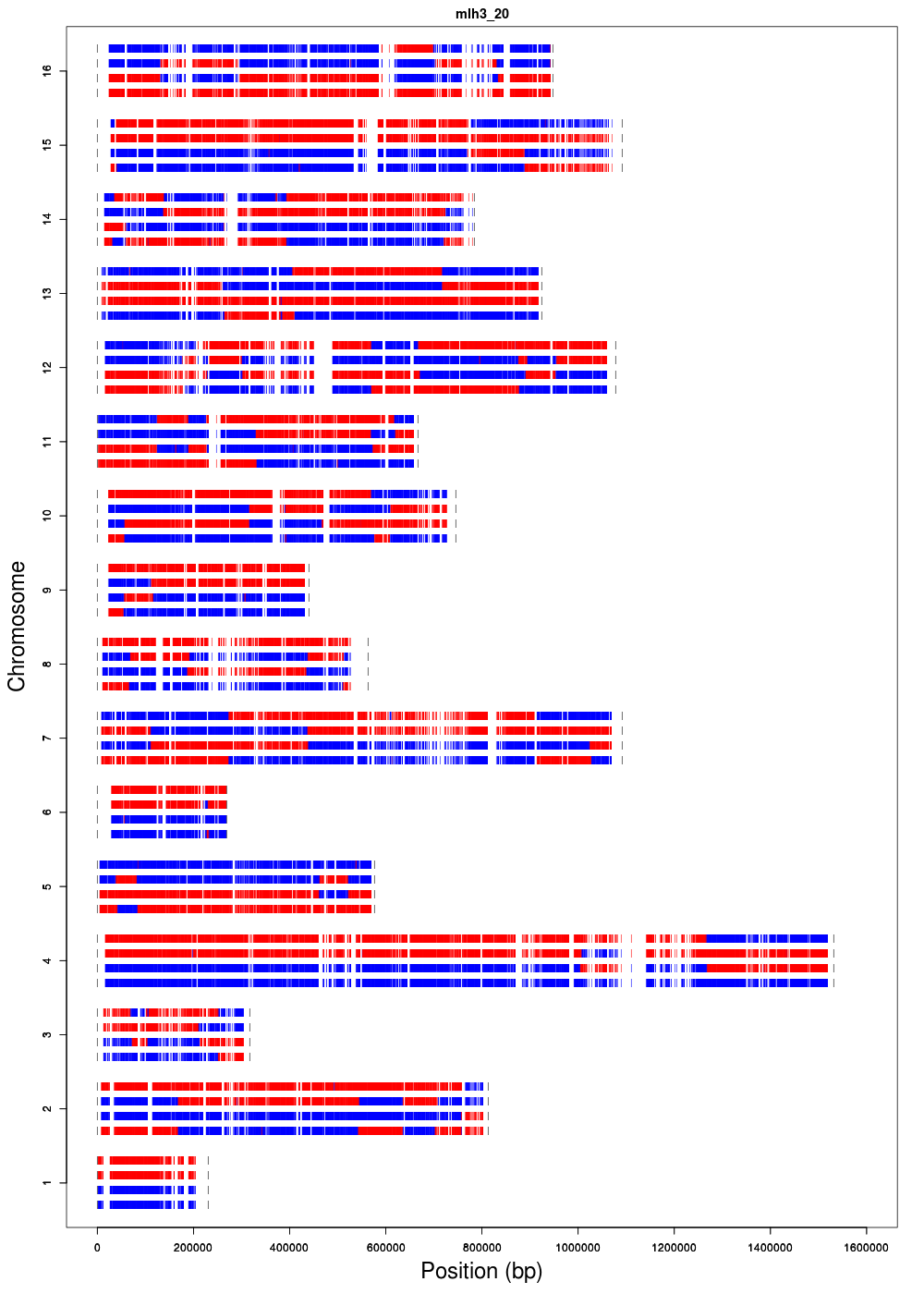

Supplement: Supplementary file 20 [file 1511FileS1.zip › S1 File/mlh3_20.tiff]

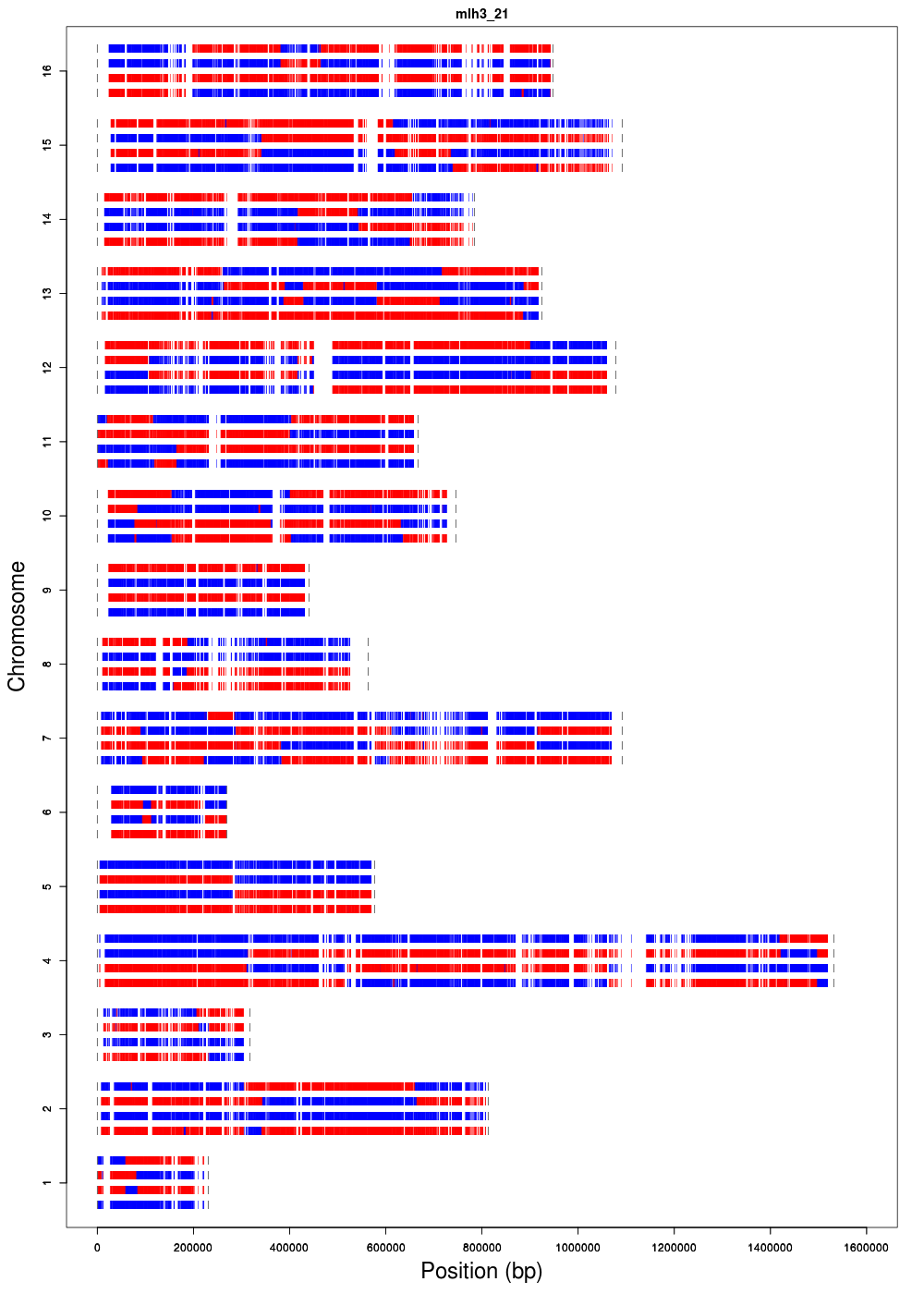

Supplement: Supplementary file 20 [file 1511FileS1.zip › S1 File/mlh3_21.tiff]

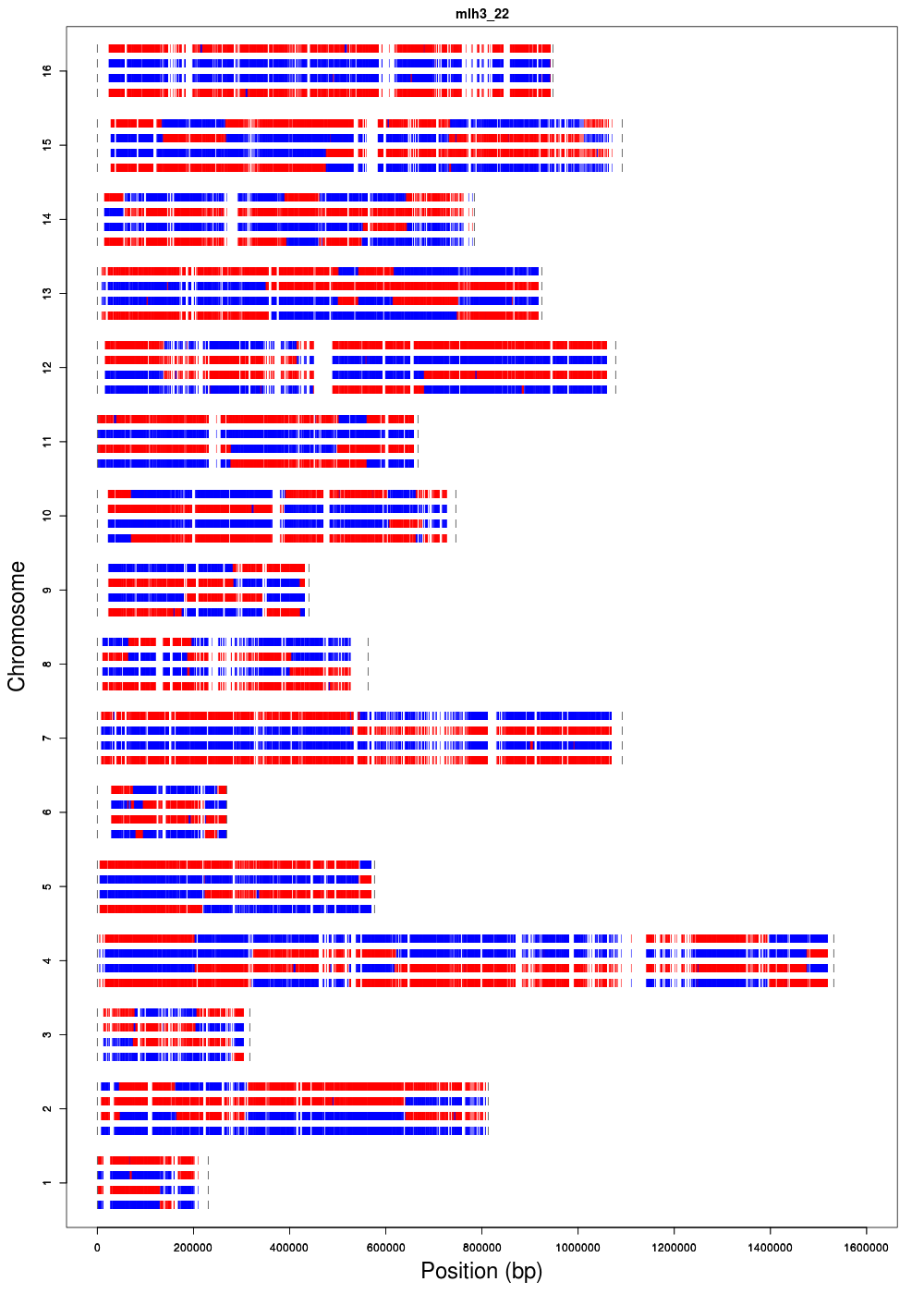

Supplement: Supplementary file 20 [file 1511FileS1.zip › S1 File/mlh3_22.tiff]

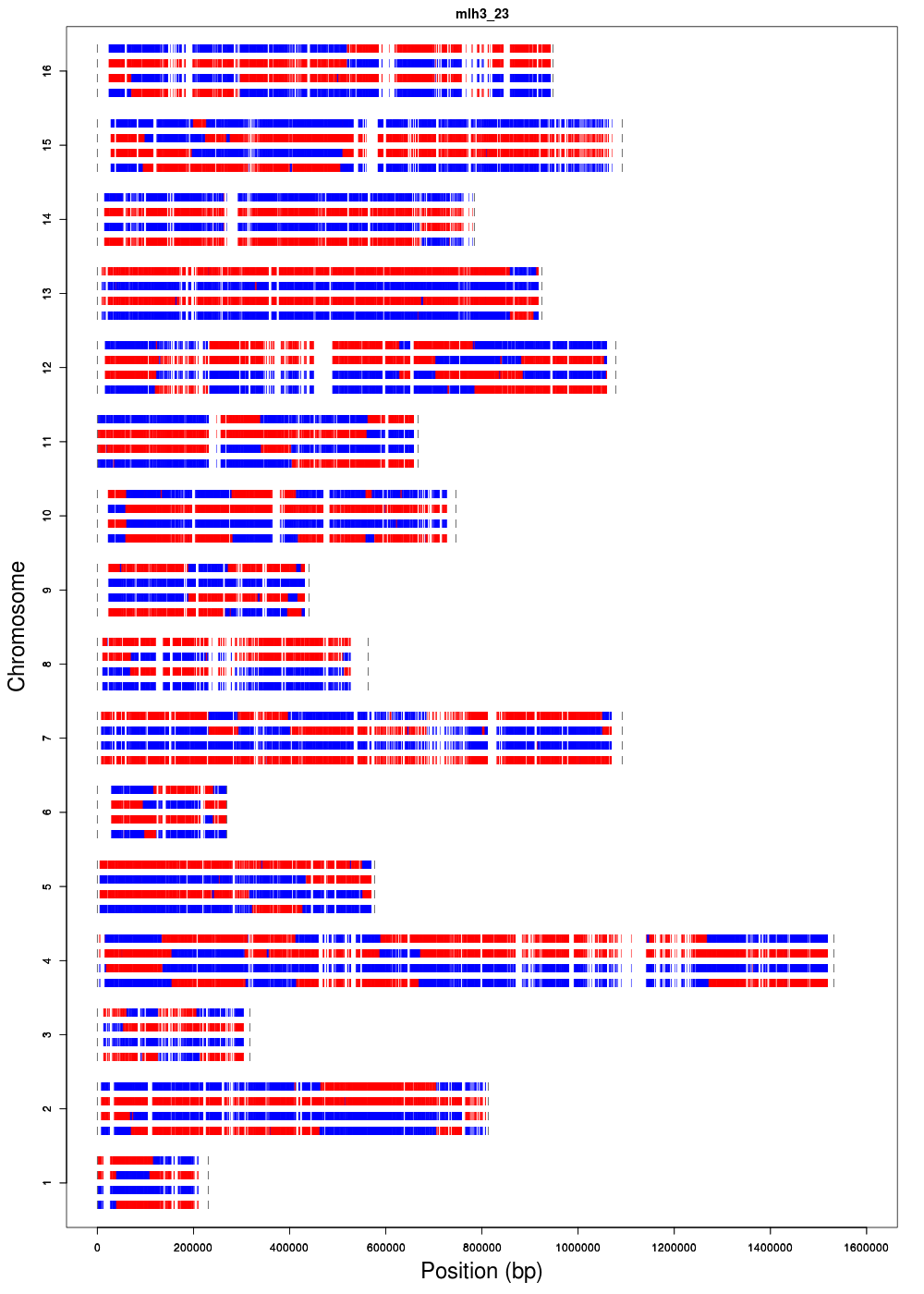

Supplement: Supplementary file 20 [file 1511FileS1.zip › S1 File/mlh3_23.tiff]

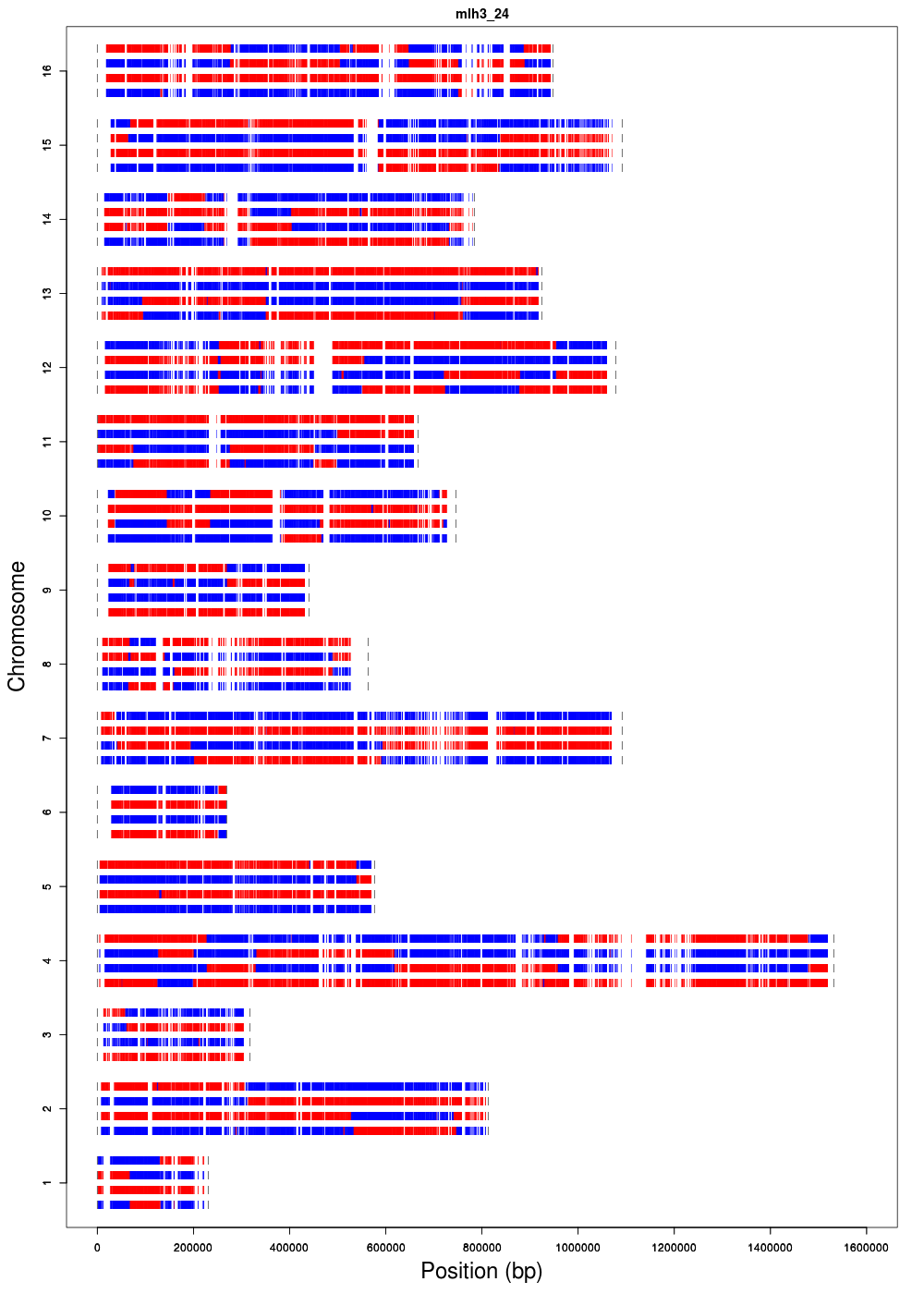

Supplement: Supplementary file 20 [file 1511FileS1.zip › S1 File/mlh3_24.tiff]

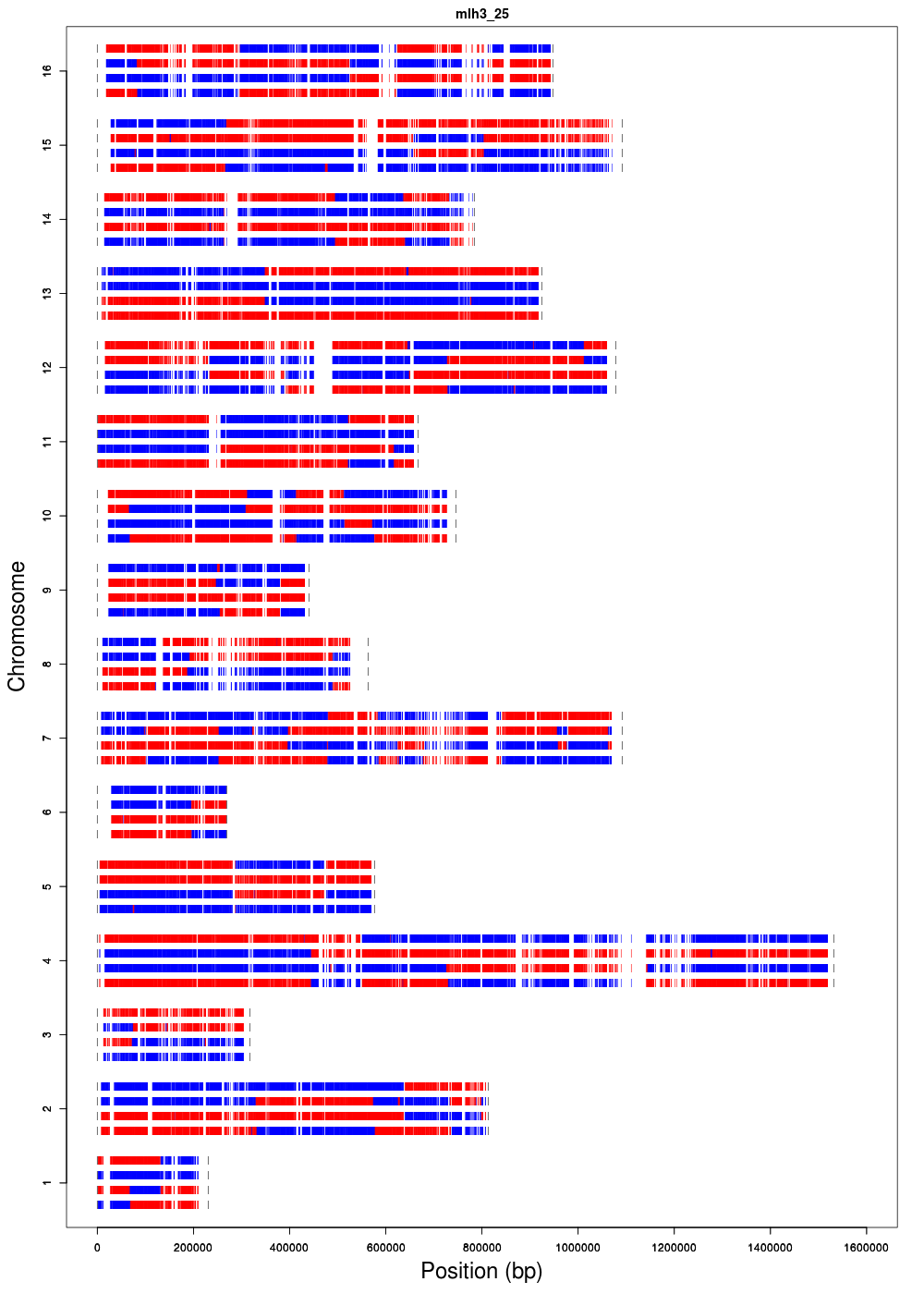

Supplement: Supplementary file 20 [file 1511FileS1.zip › S1 File/mlh3_25.tiff]

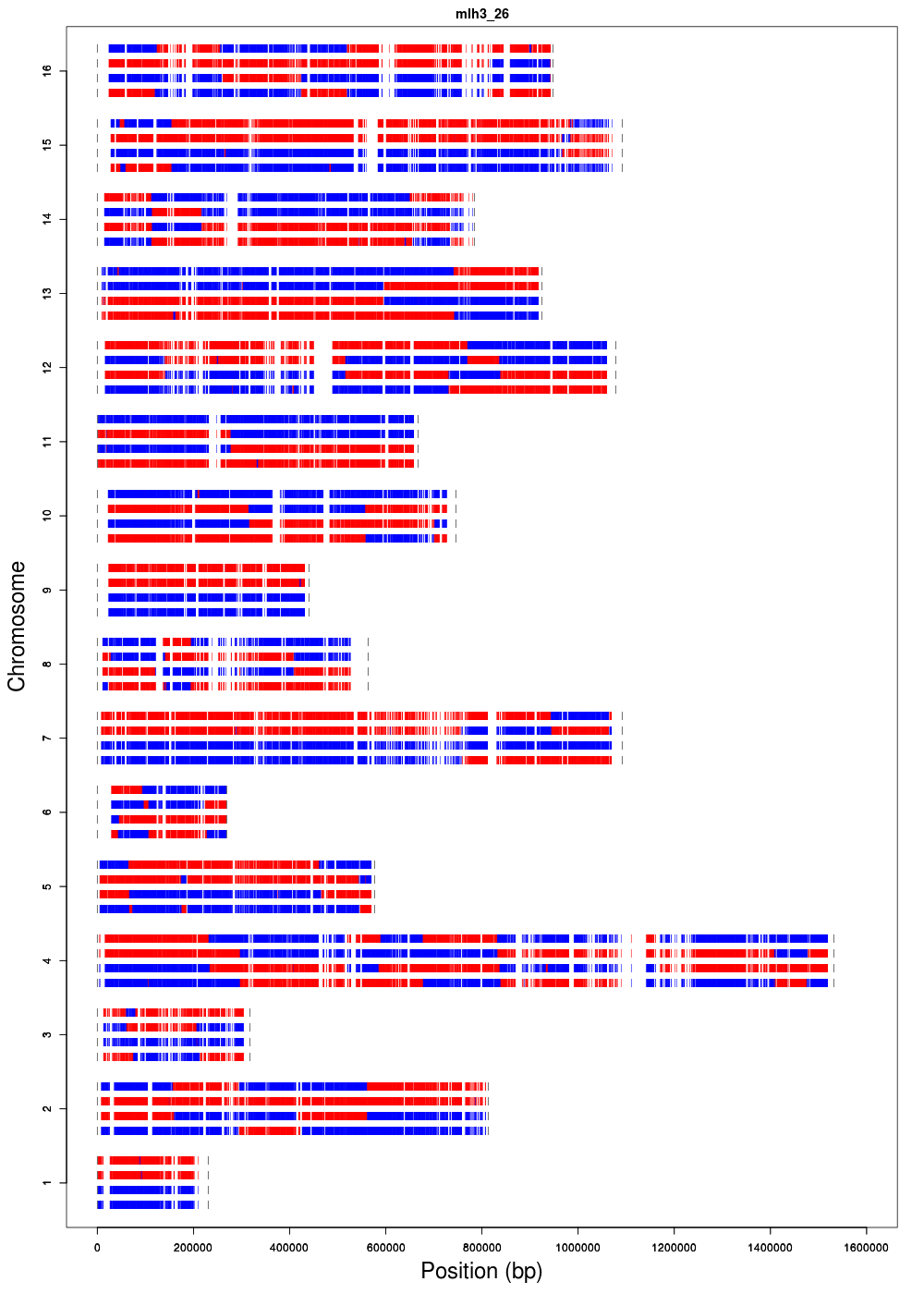

Supplement: Supplementary file 20 [file 1511FileS1.zip › S1 File/mlh3_26.tiff]

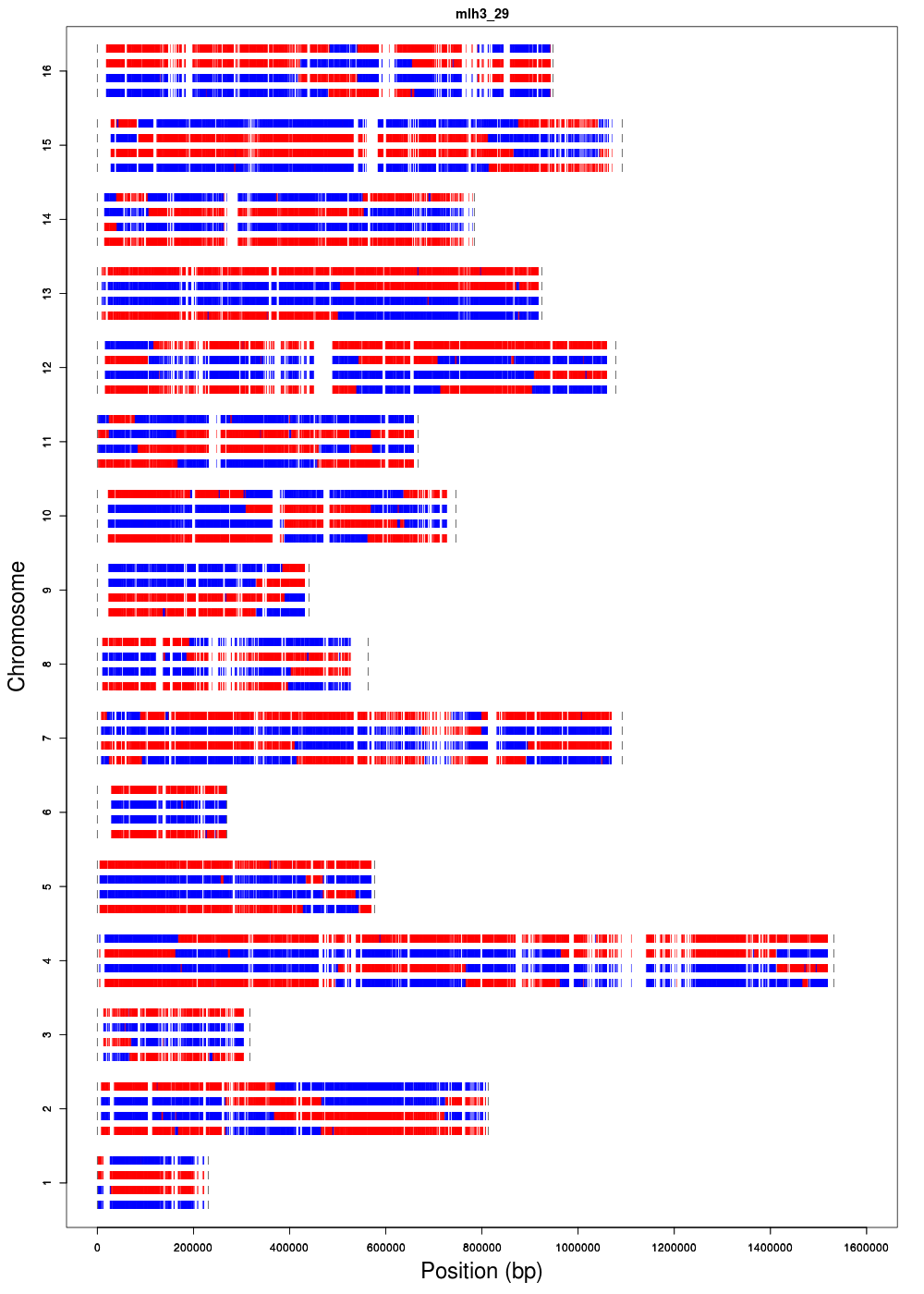

Supplement: Supplementary file 20 [file 1511FileS1.zip › S1 File/mlh3_29.tiff]

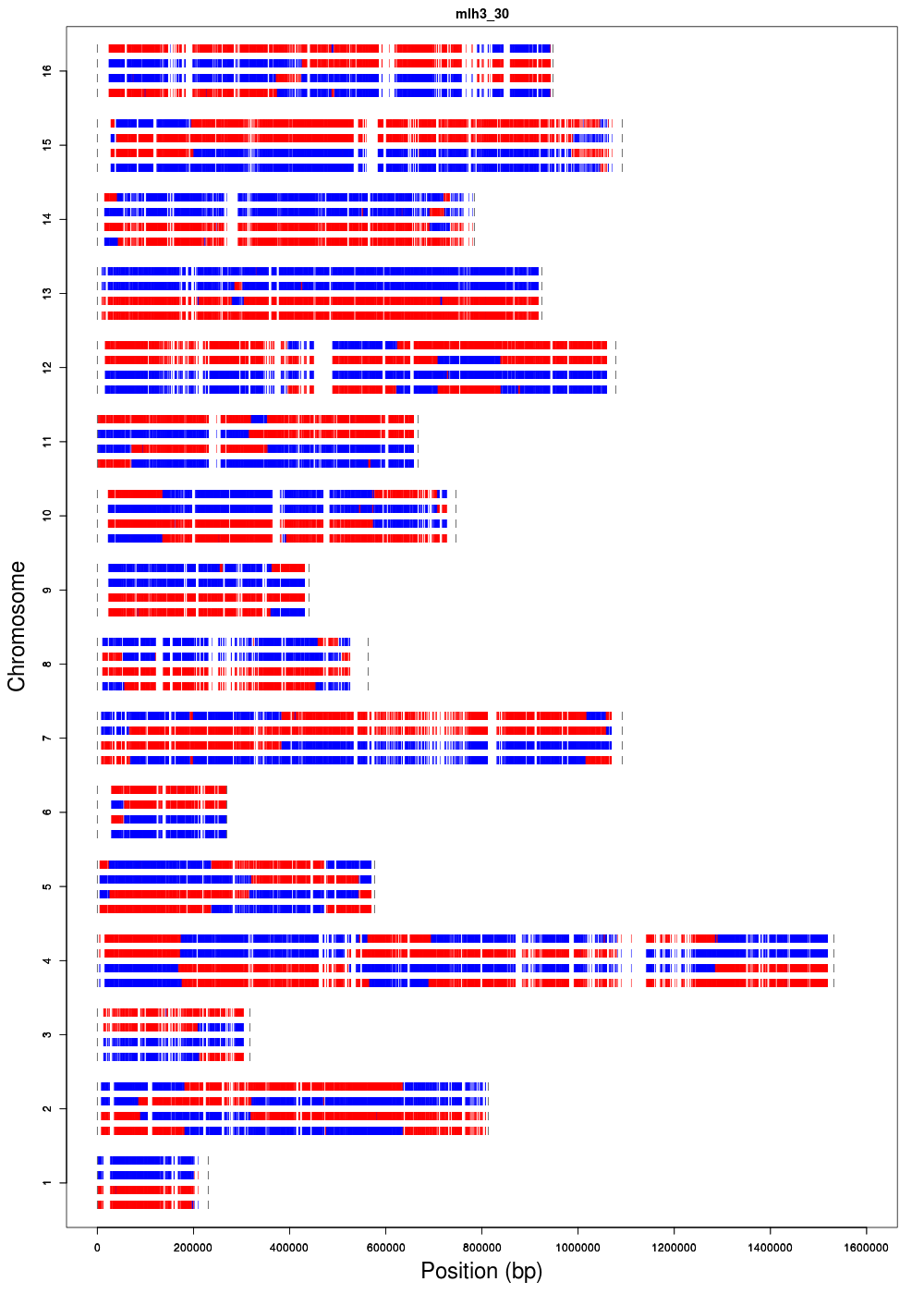

Supplement: Supplementary file 20 [file 1511FileS1.zip › S1 File/mlh3_30.tiff]

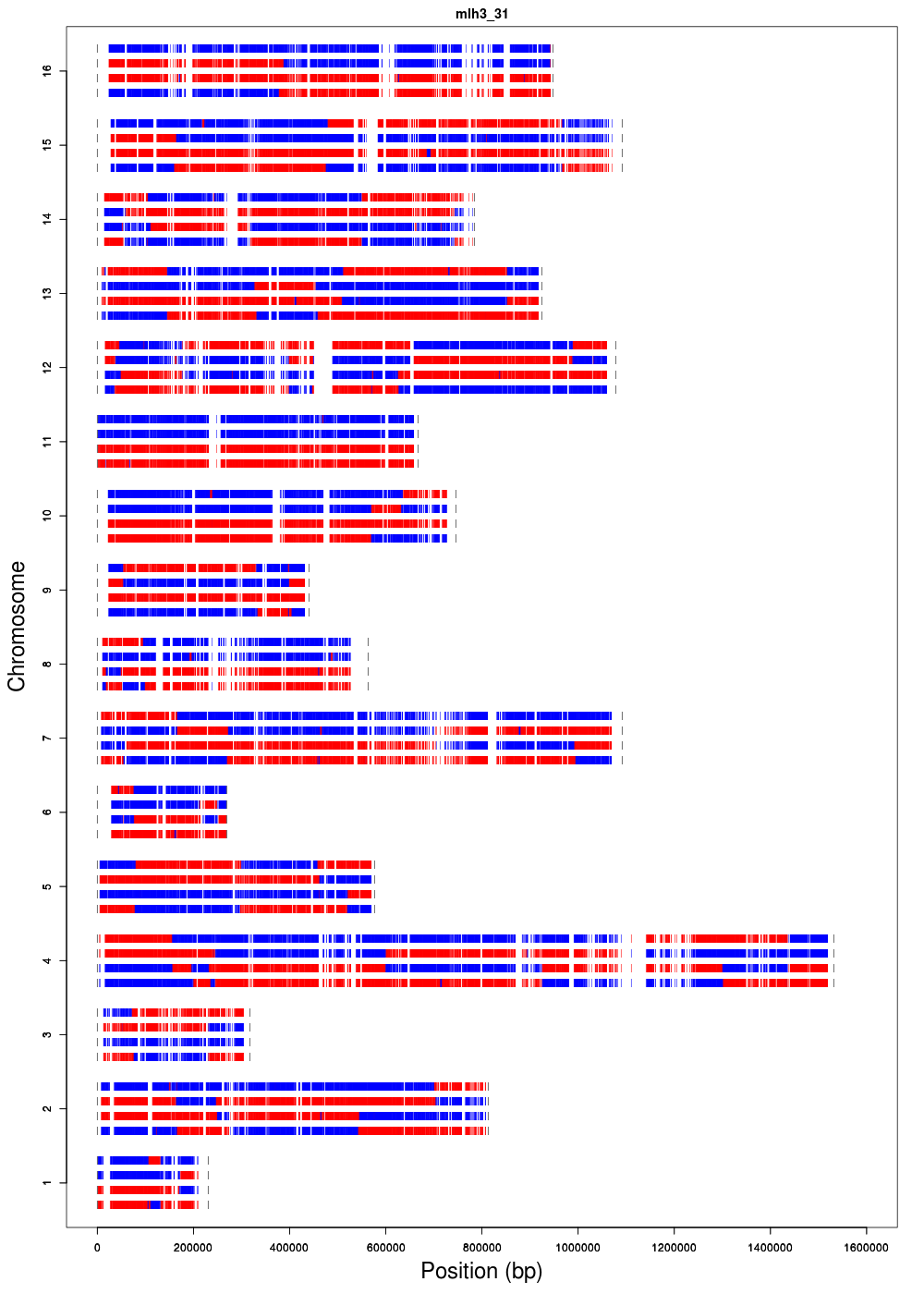

Supplement: Supplementary file 20 [file 1511FileS1.zip › S1 File/mlh3_31.tiff]

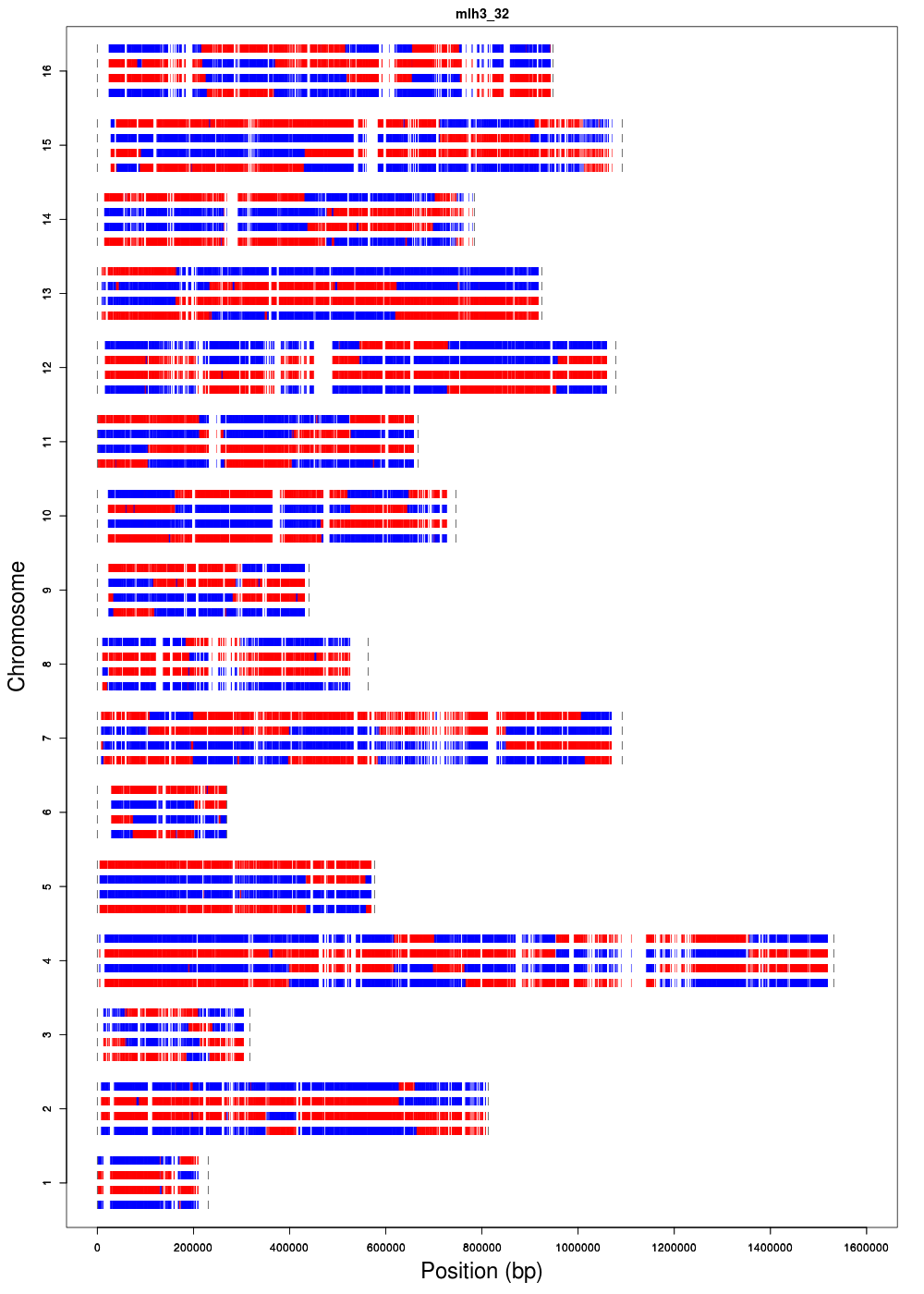

Supplement: Supplementary file 20 [file 1511FileS1.zip › S1 File/mlh3_32.tiff]

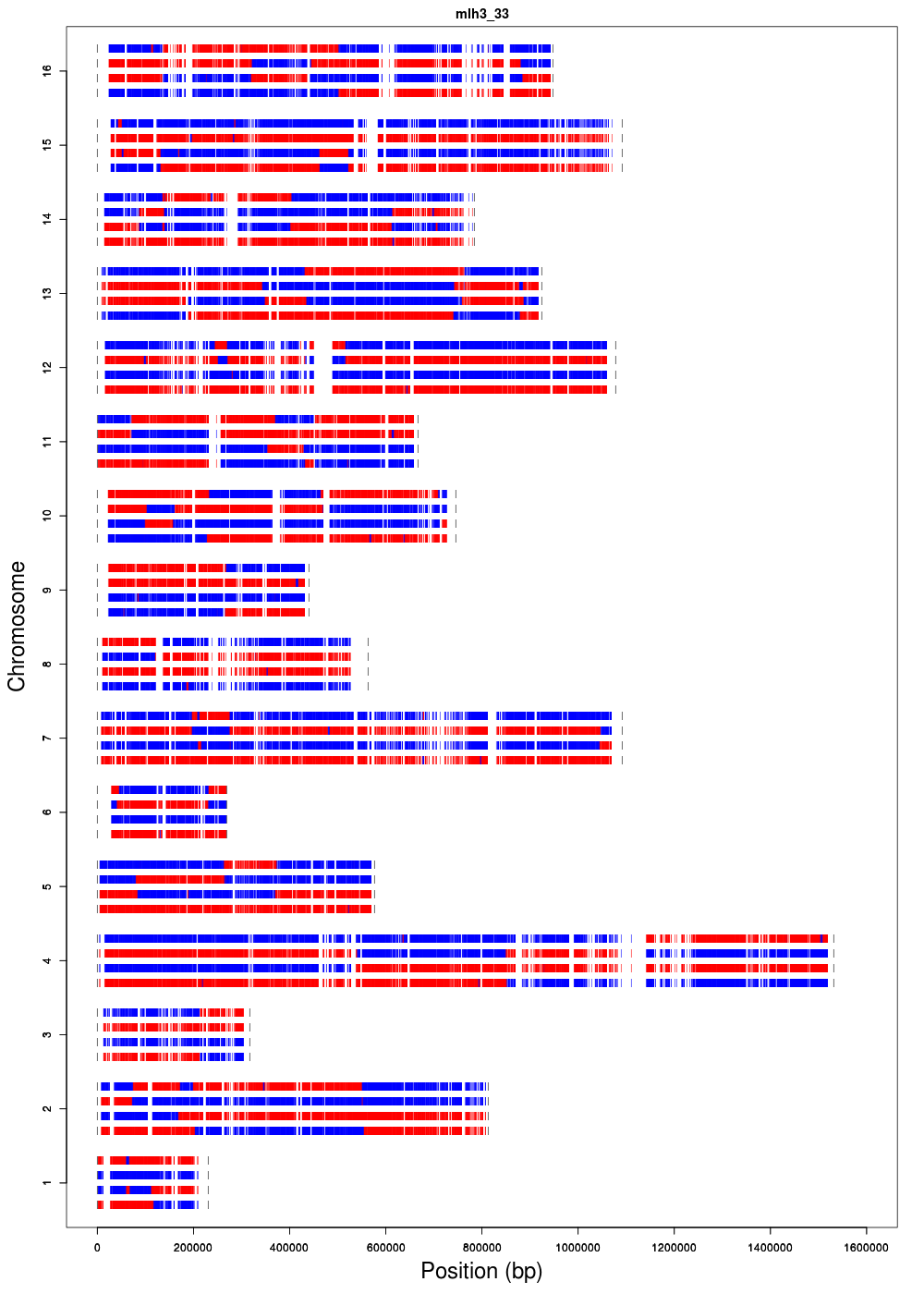

Supplement: Supplementary file 20 [file 1511FileS1.zip › S1 File/mlh3_33.tiff]

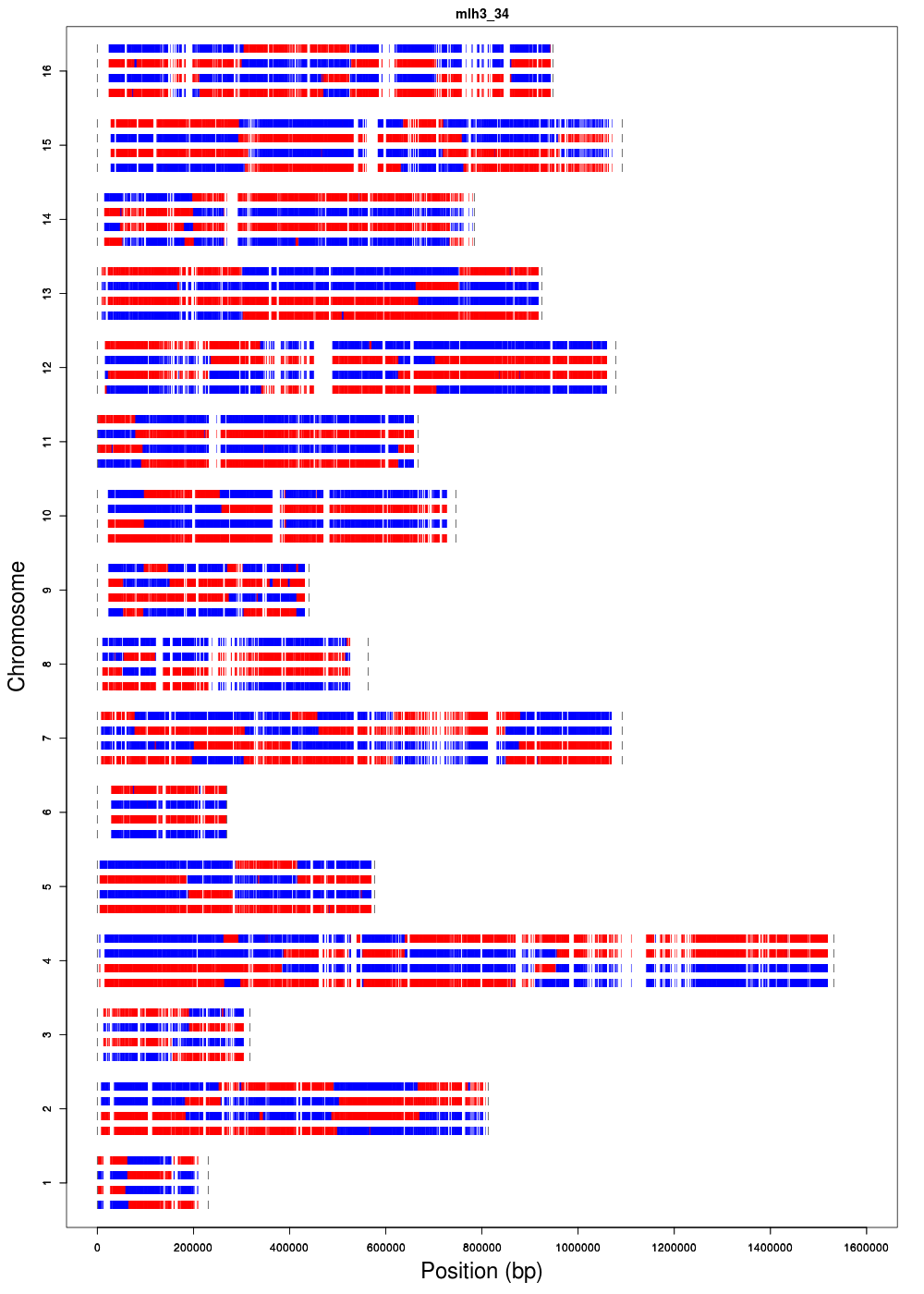

Supplement: Supplementary file 20 [file 1511FileS1.zip › S1 File/mlh3_34.tiff]

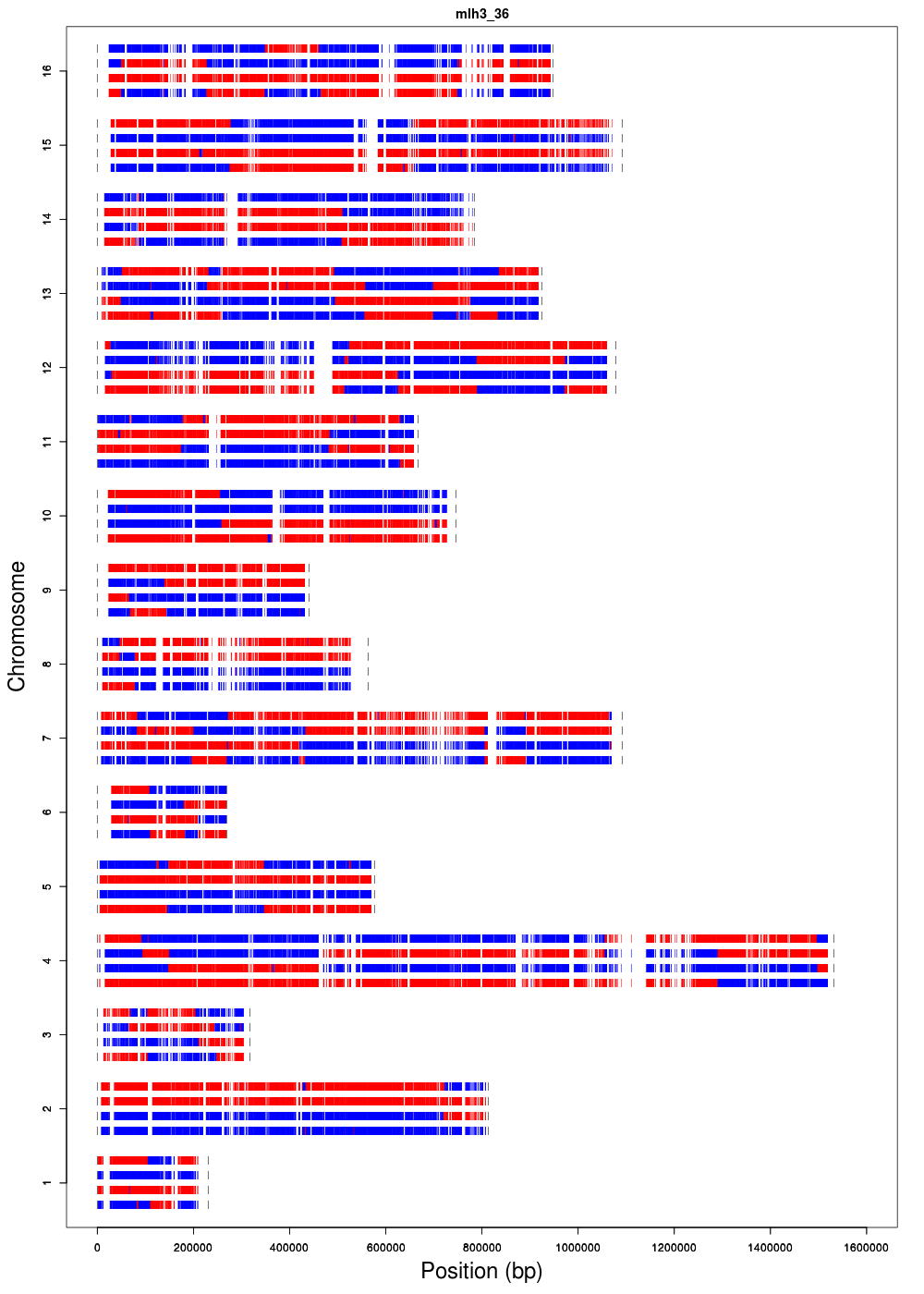

Supplement: Supplementary file 20 [file 1511FileS1.zip › S1 File/mlh3_36.tiff]

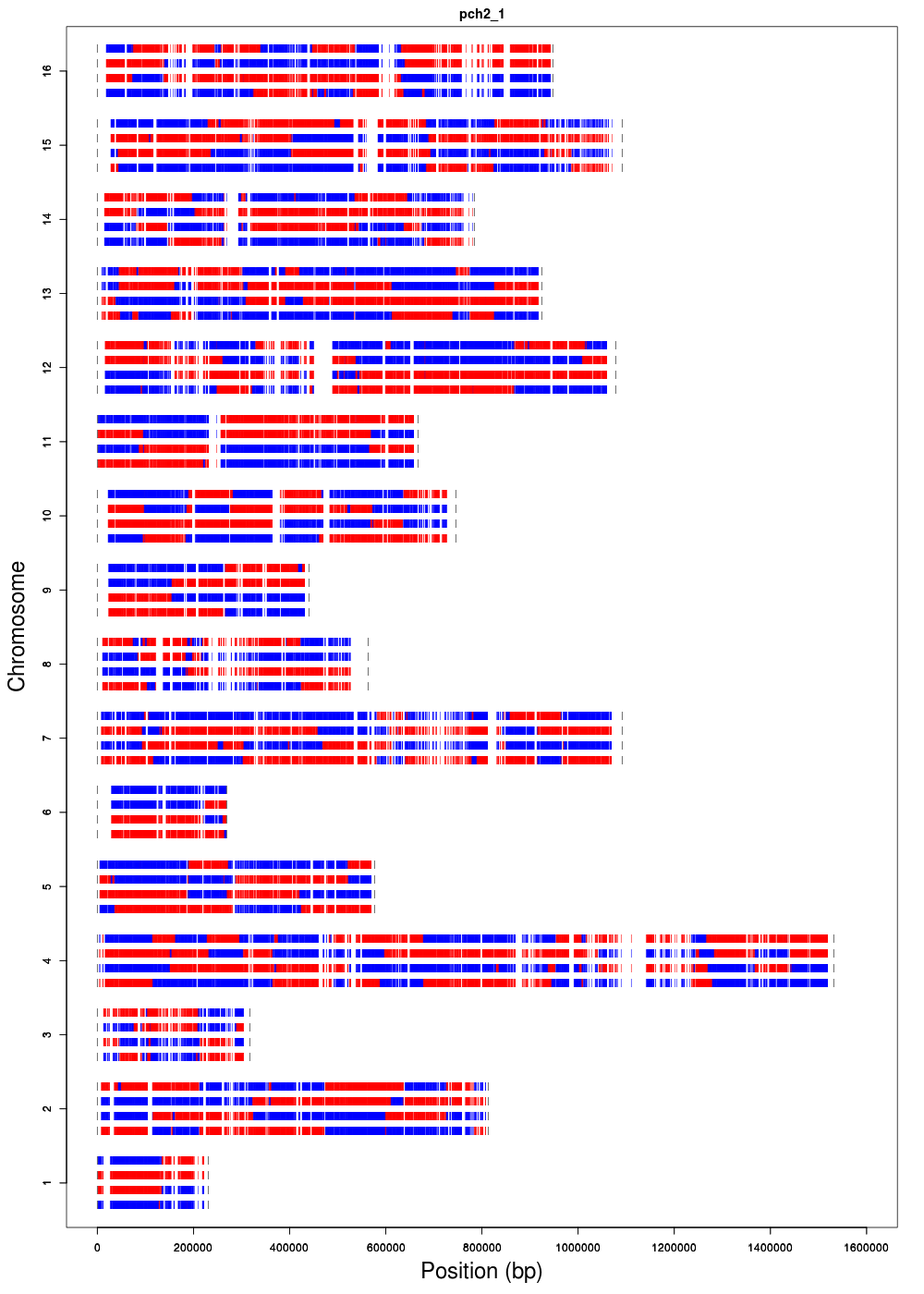

Supplement: Supplementary file 20 [file 1511FileS1.zip › S1 File/pch2_1.tiff]

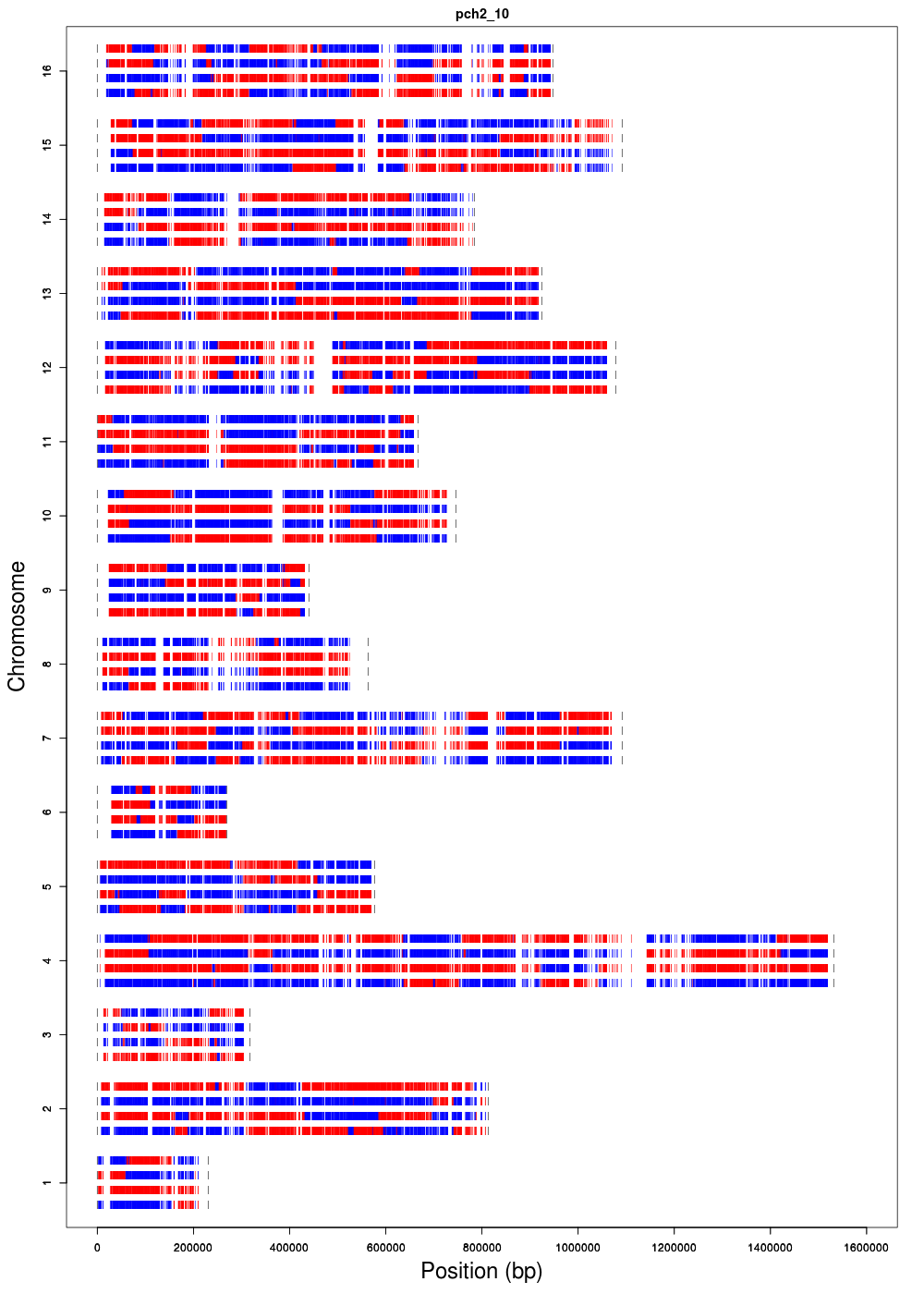

Supplement: Supplementary file 20 [file 1511FileS1.zip › S1 File/pch2_10.tiff]

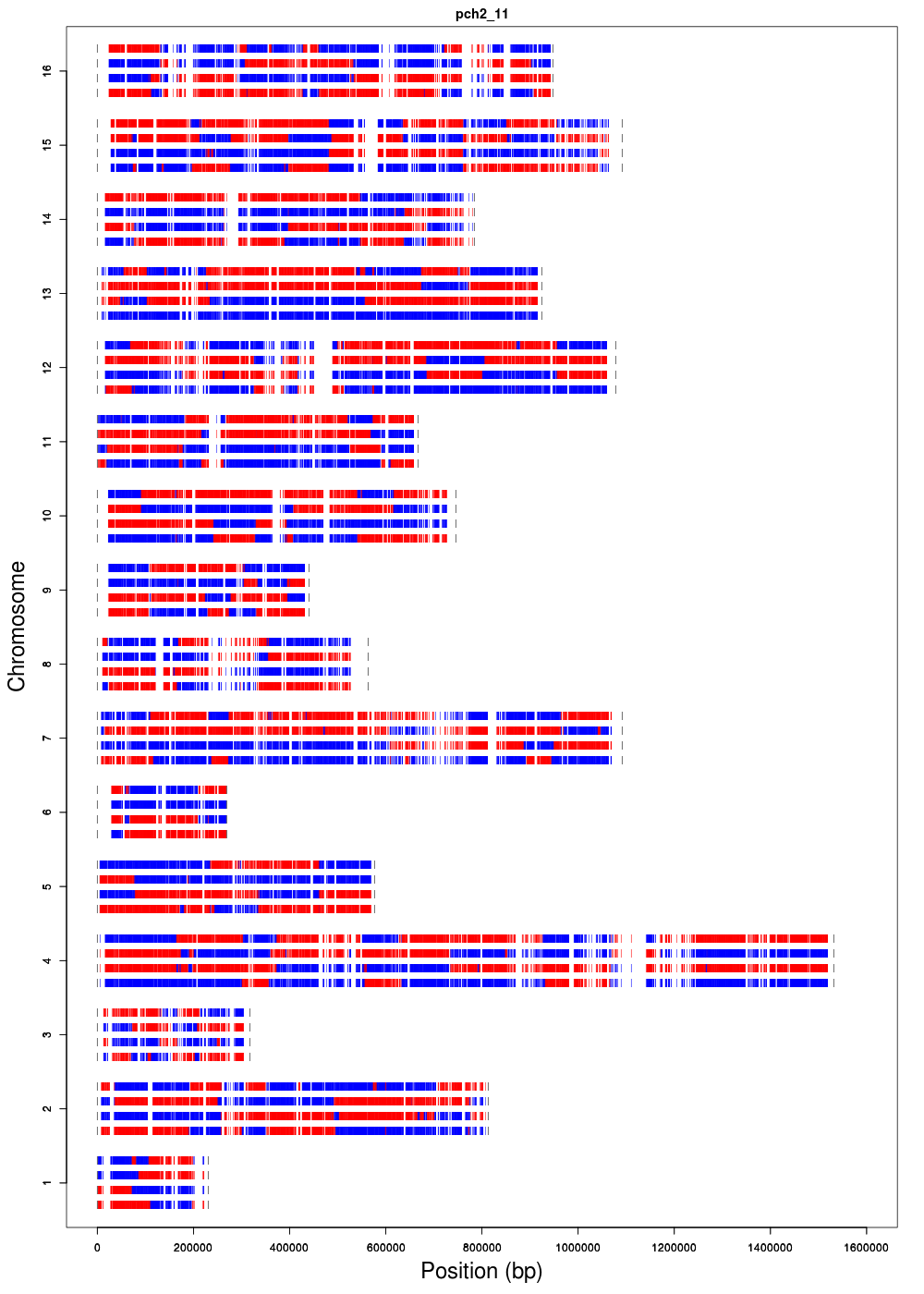

Supplement: Supplementary file 20 [file 1511FileS1.zip › S1 File/pch2_11.tiff]

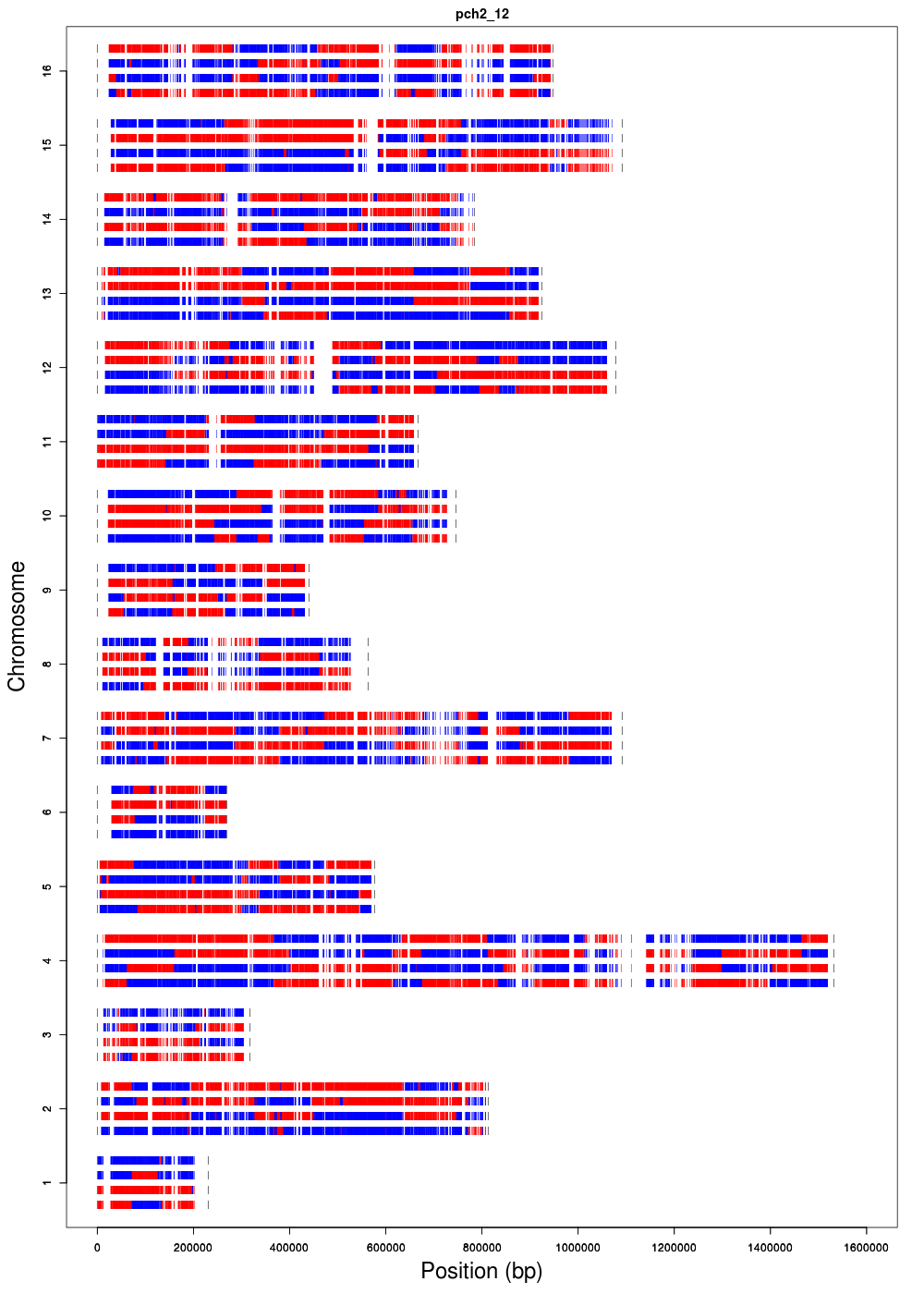

Supplement: Supplementary file 20 [file 1511FileS1.zip › S1 File/pch2_12.tiff]

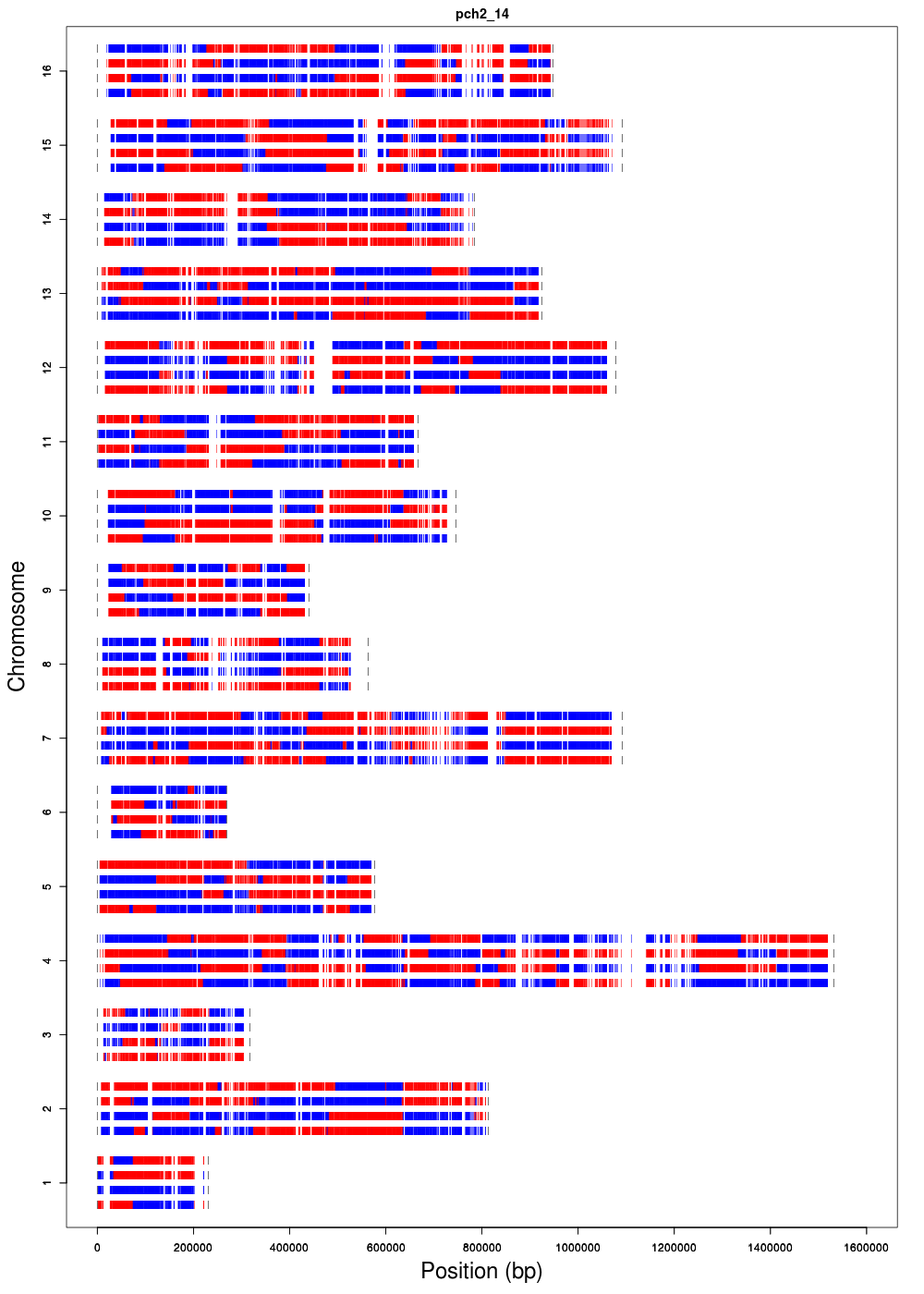

Supplement: Supplementary file 20 [file 1511FileS1.zip › S1 File/pch2_14.tiff]

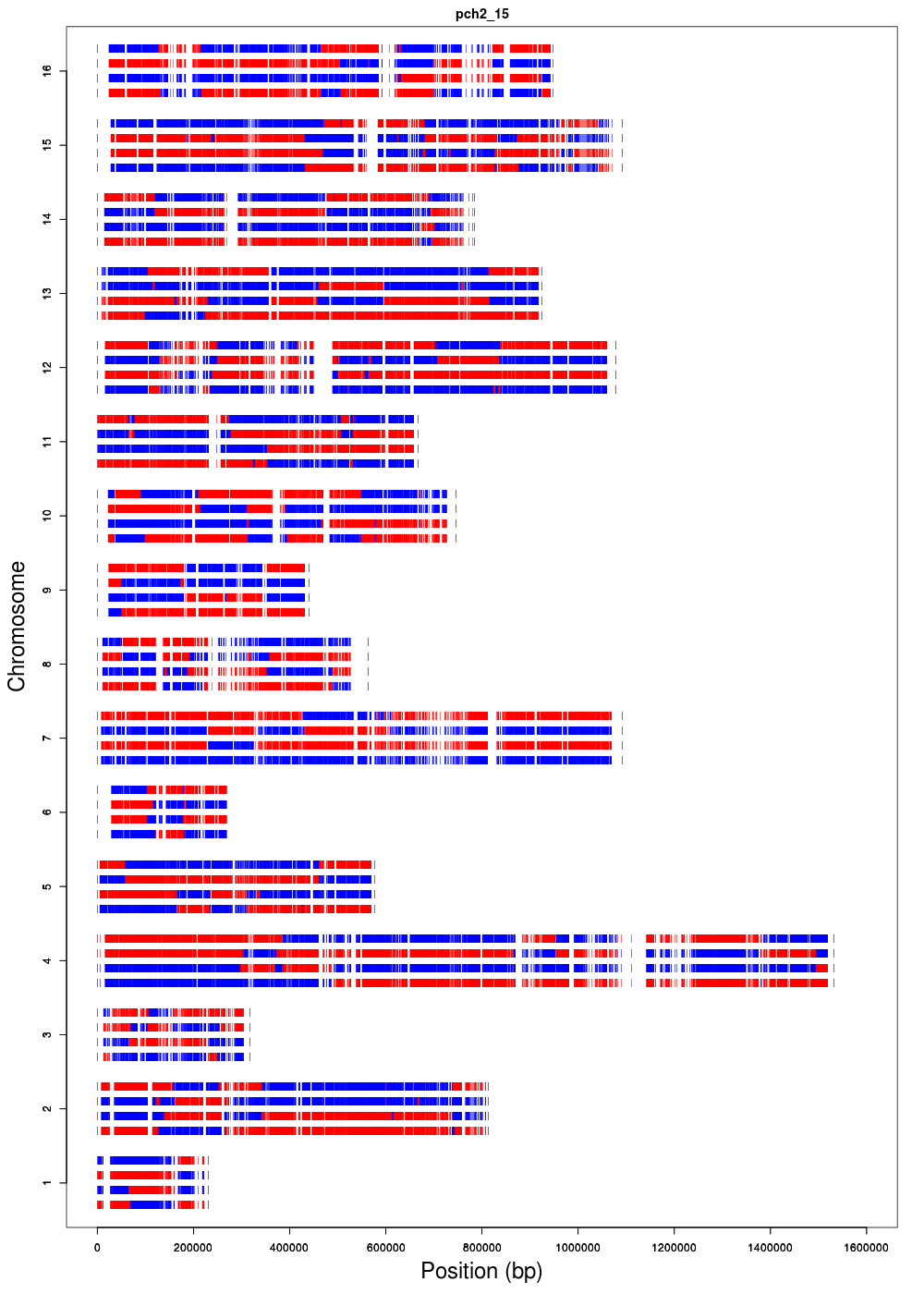

Supplement: Supplementary file 20 [file 1511FileS1.zip › S1 File/pch2_15.tiff]

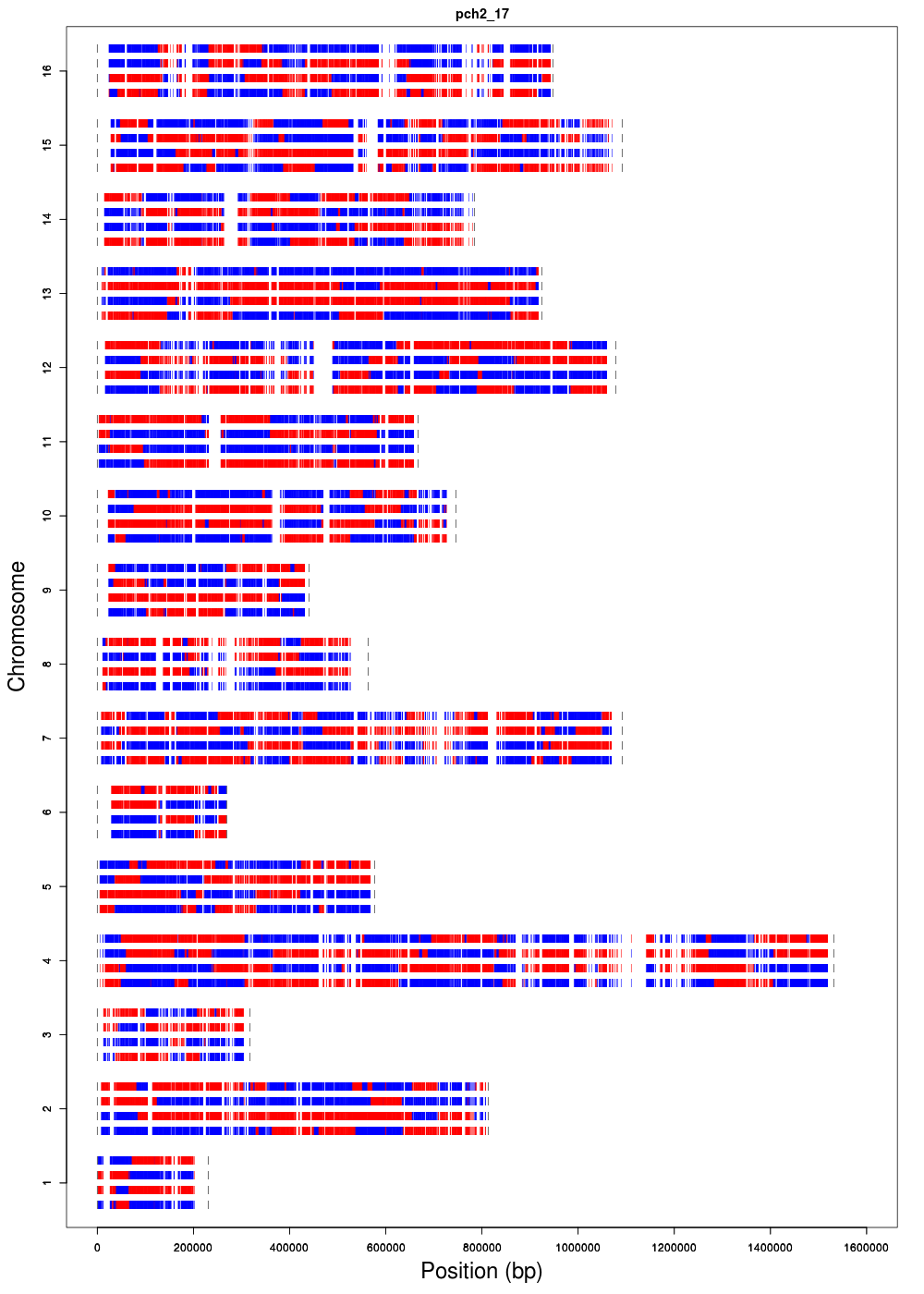

Supplement: Supplementary file 20 [file 1511FileS1.zip › S1 File/pch2_17.tiff]

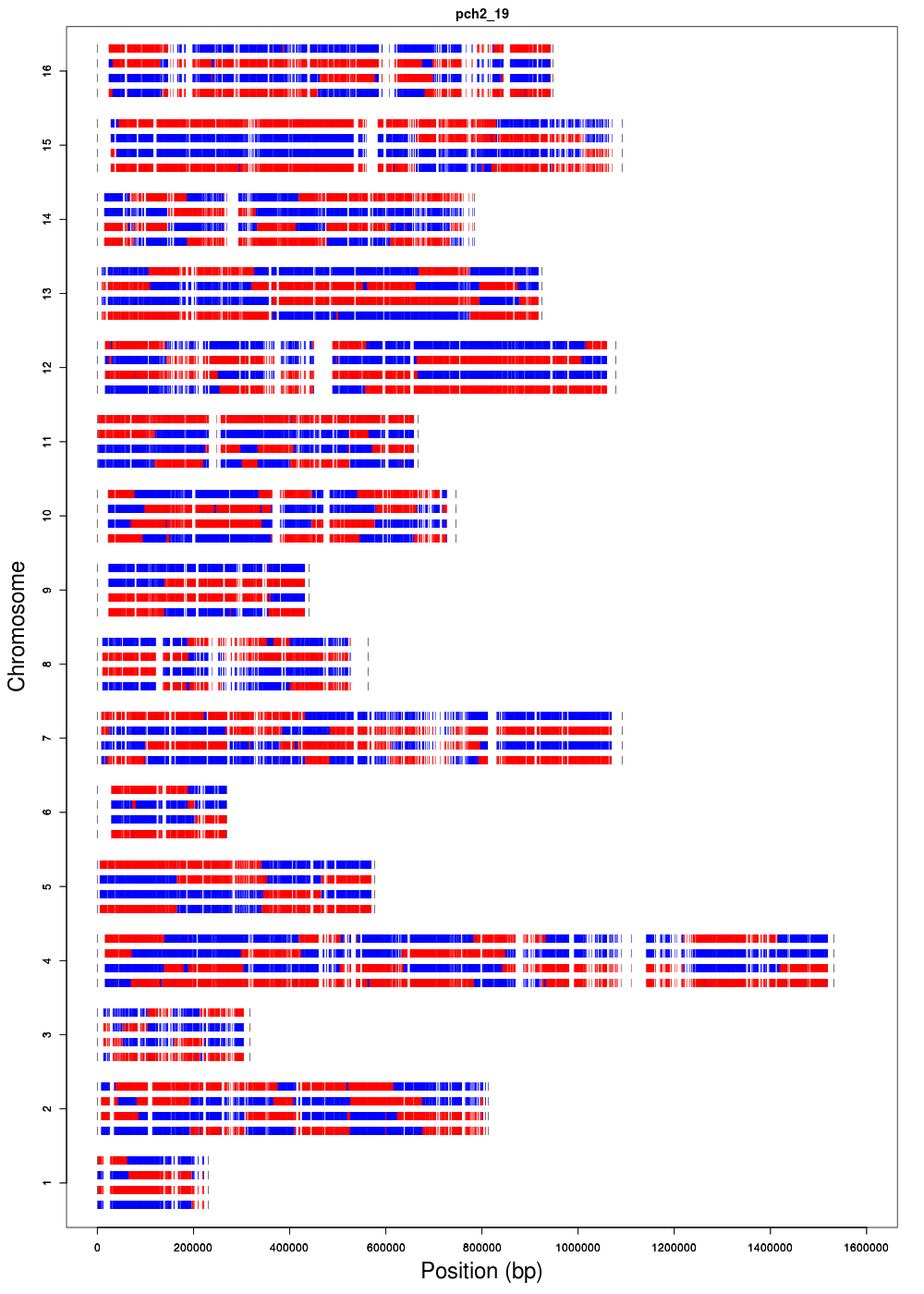

Supplement: Supplementary file 20 [file 1511FileS1.zip › S1 File/pch2_19.tiff]

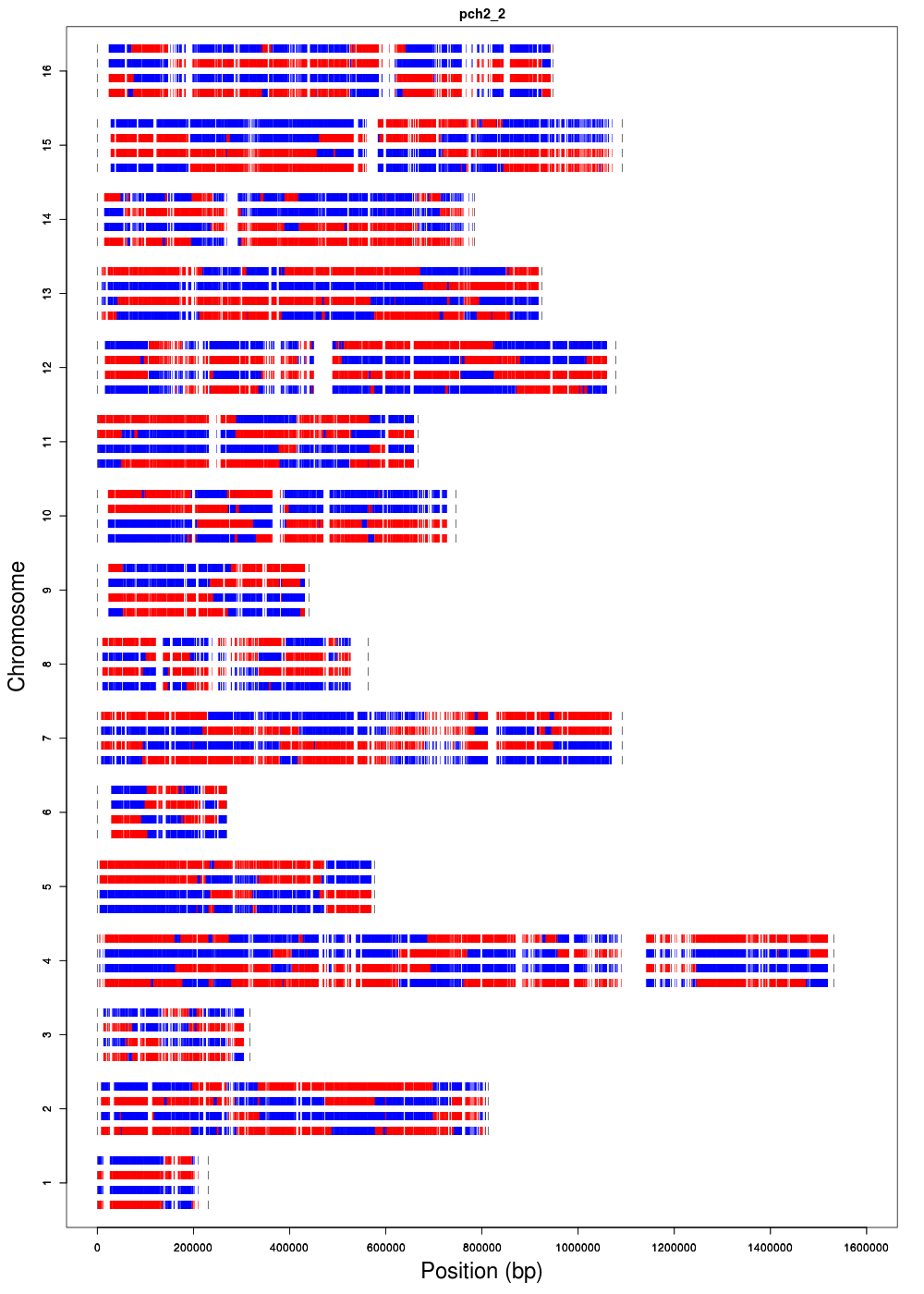

Supplement: Supplementary file 20 [file 1511FileS1.zip › S1 File/pch2_2.tiff]

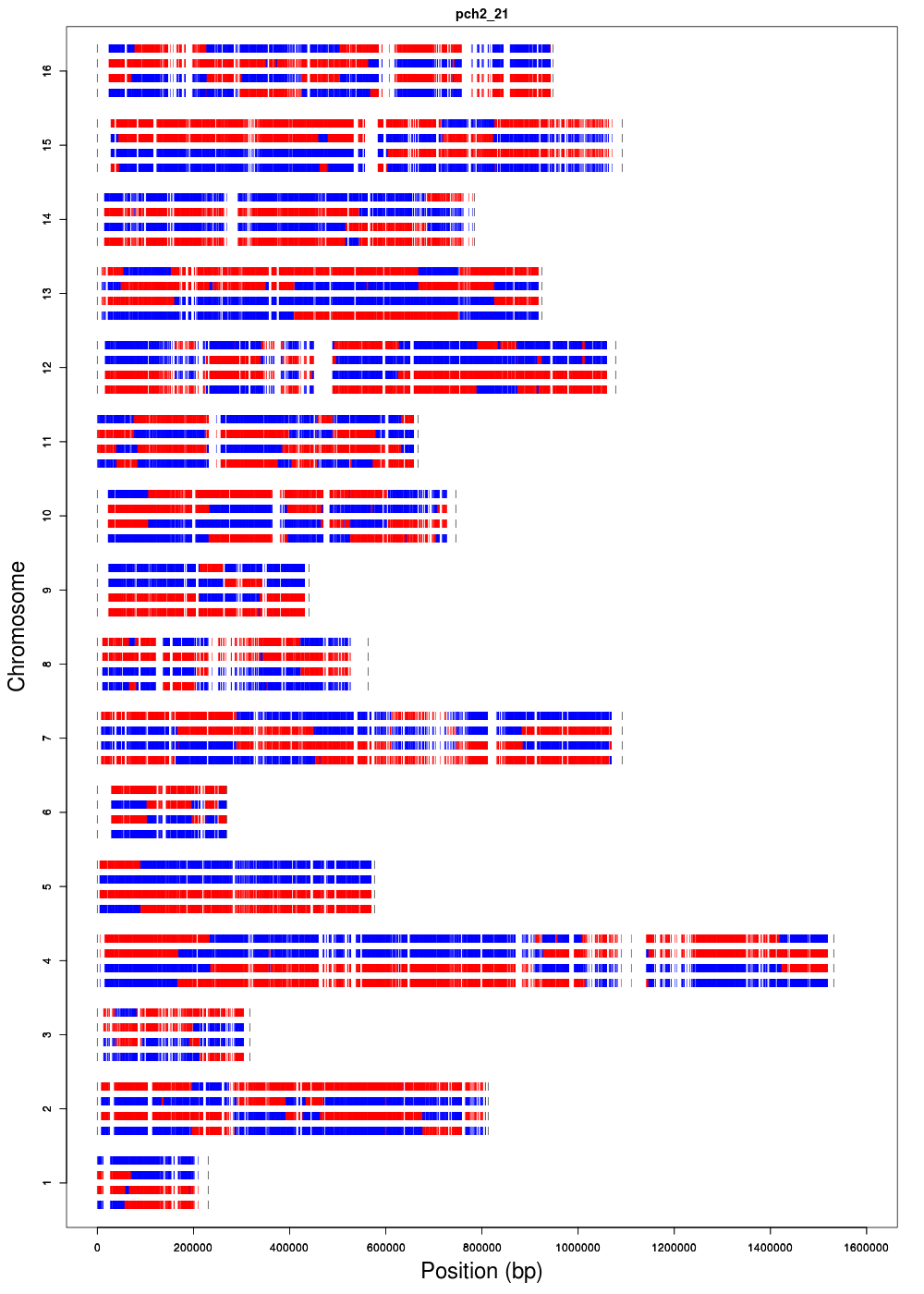

Supplement: Supplementary file 20 [file 1511FileS1.zip › S1 File/pch2_21.tiff]

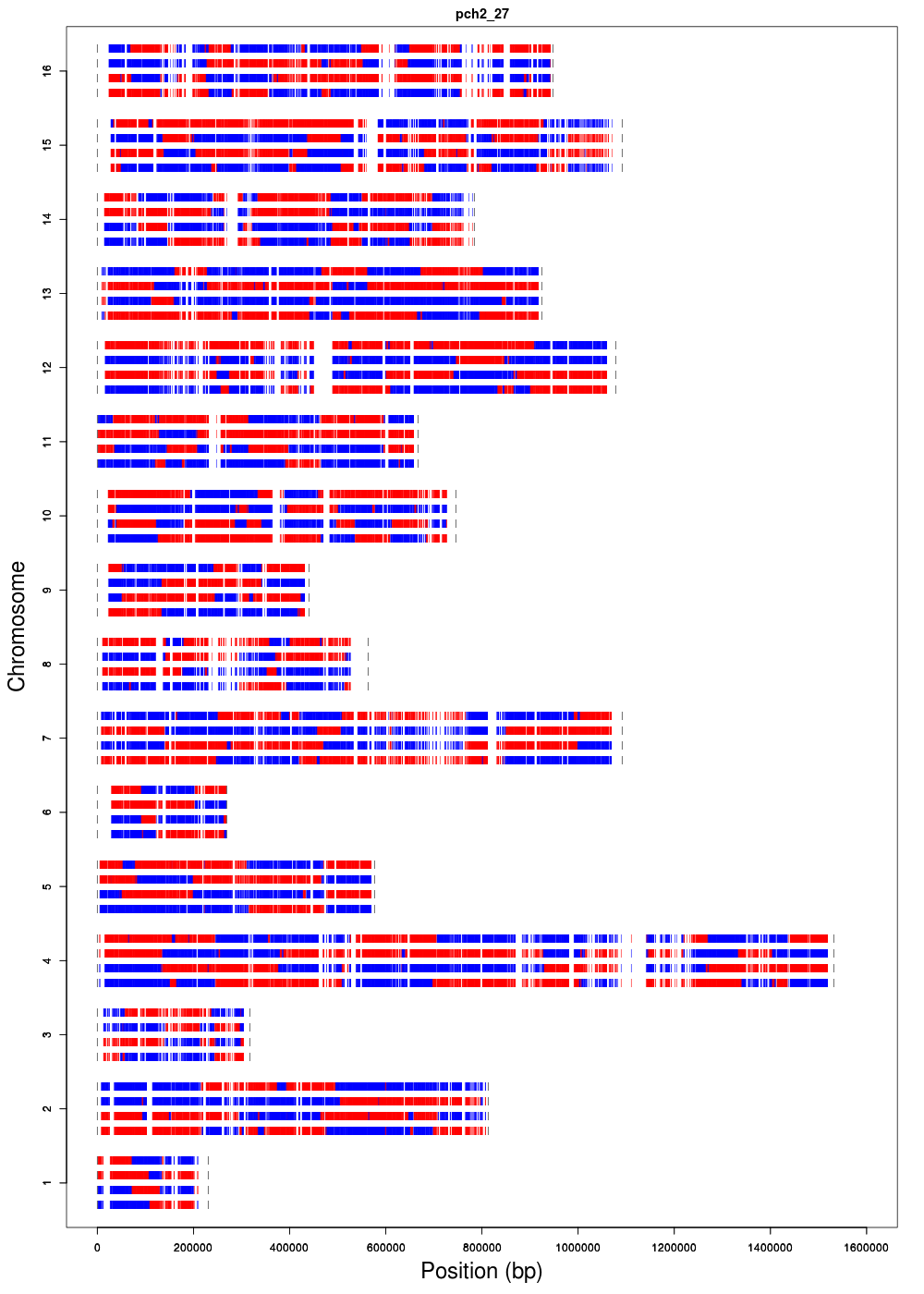

Supplement: Supplementary file 20 [file 1511FileS1.zip › S1 File/pch2_27.tiff]

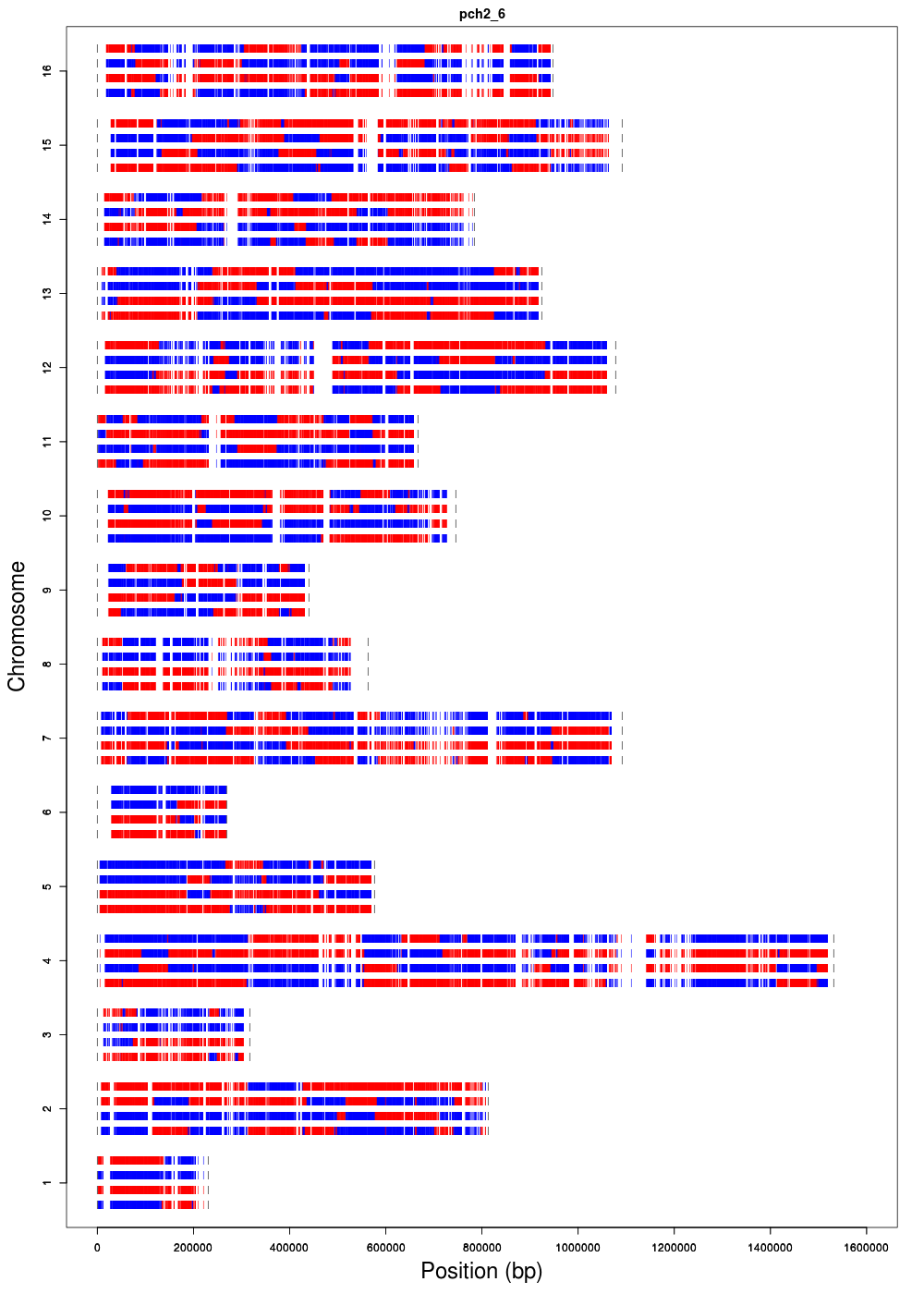

Supplement: Supplementary file 20 [file 1511FileS1.zip › S1 File/pch2_6.tiff]

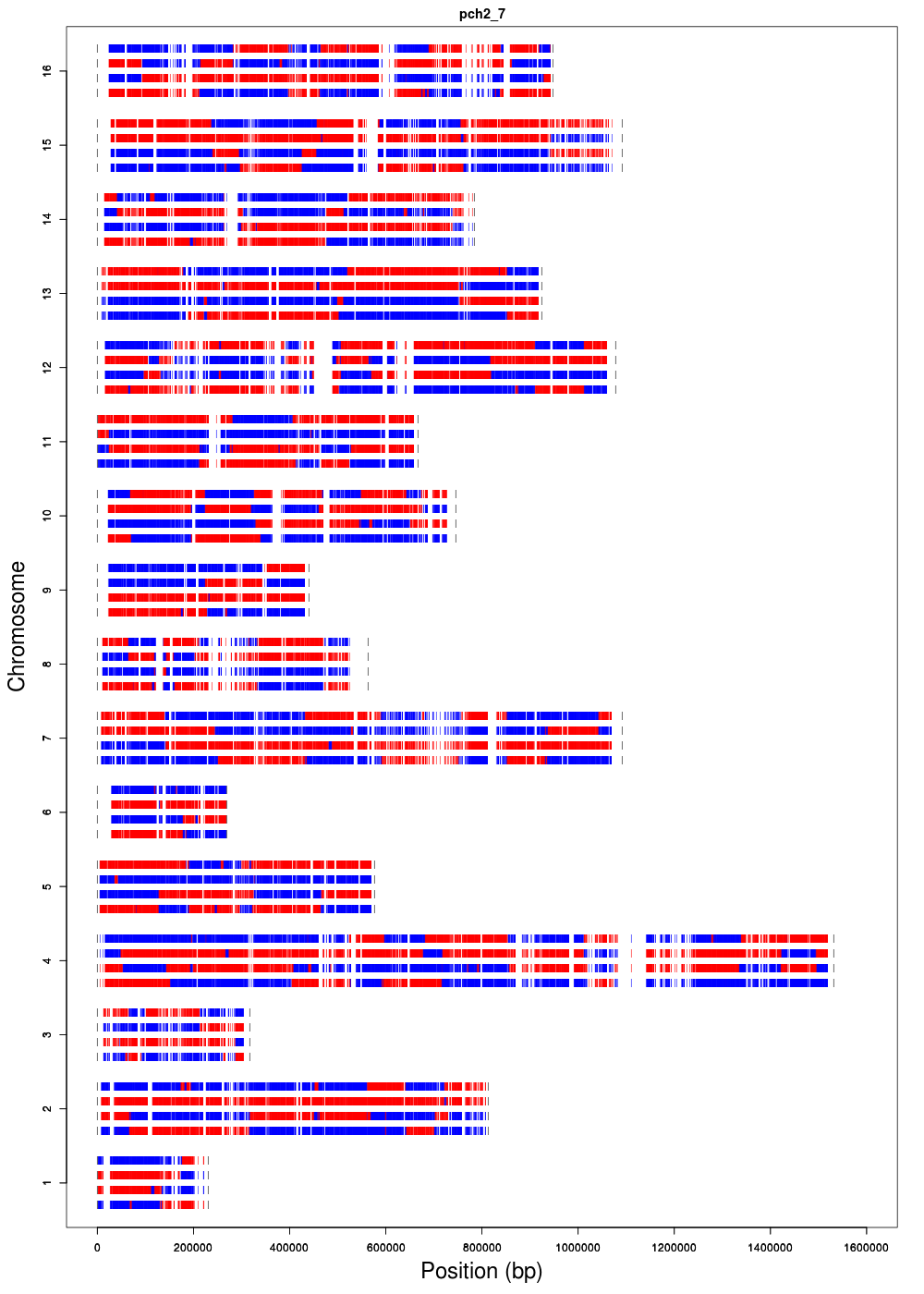

Supplement: Supplementary file 20 [file 1511FileS1.zip › S1 File/pch2_7.tiff]

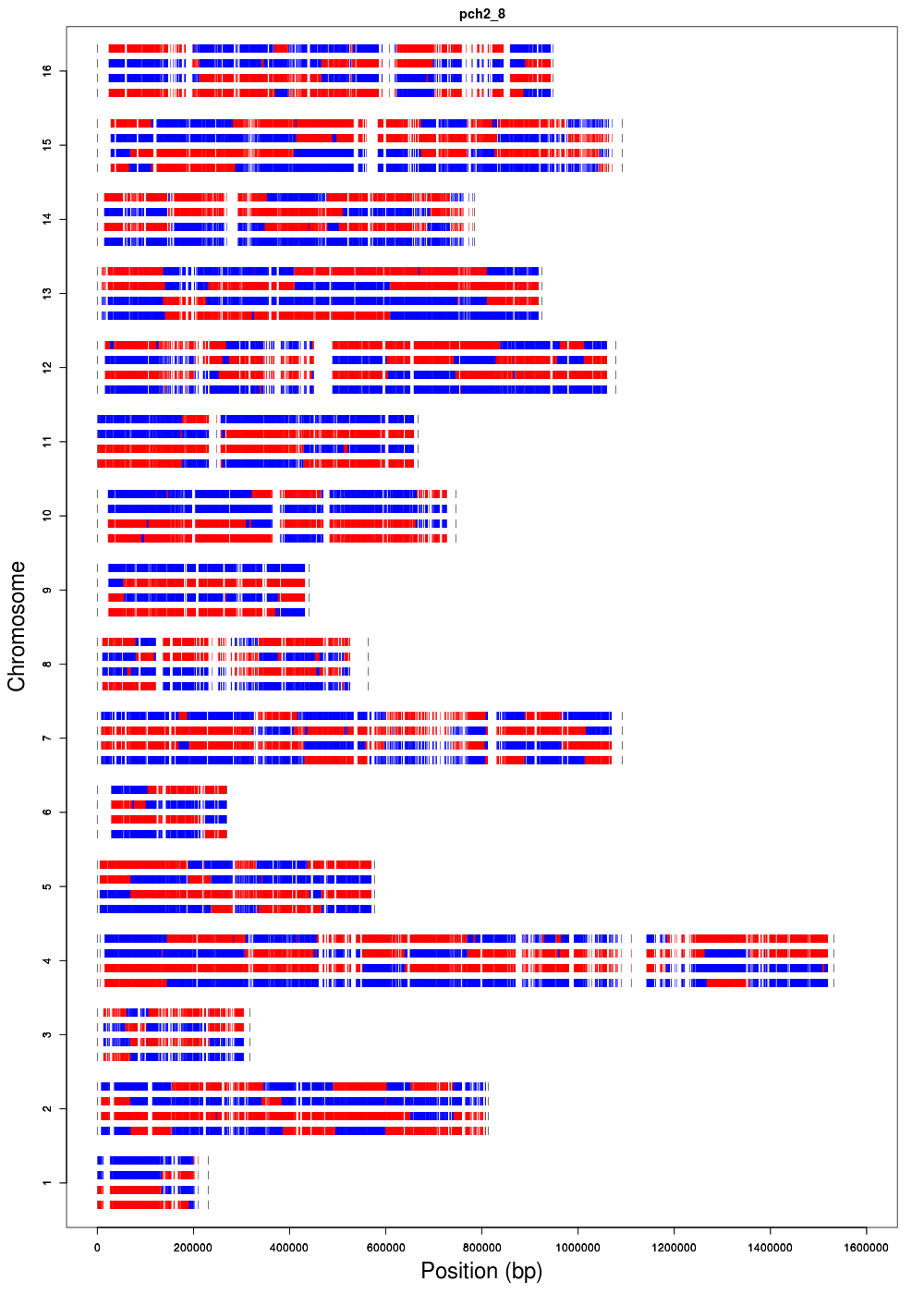

Supplement: Supplementary file 20 [file 1511FileS1.zip › S1 File/pch2_8.tiff]

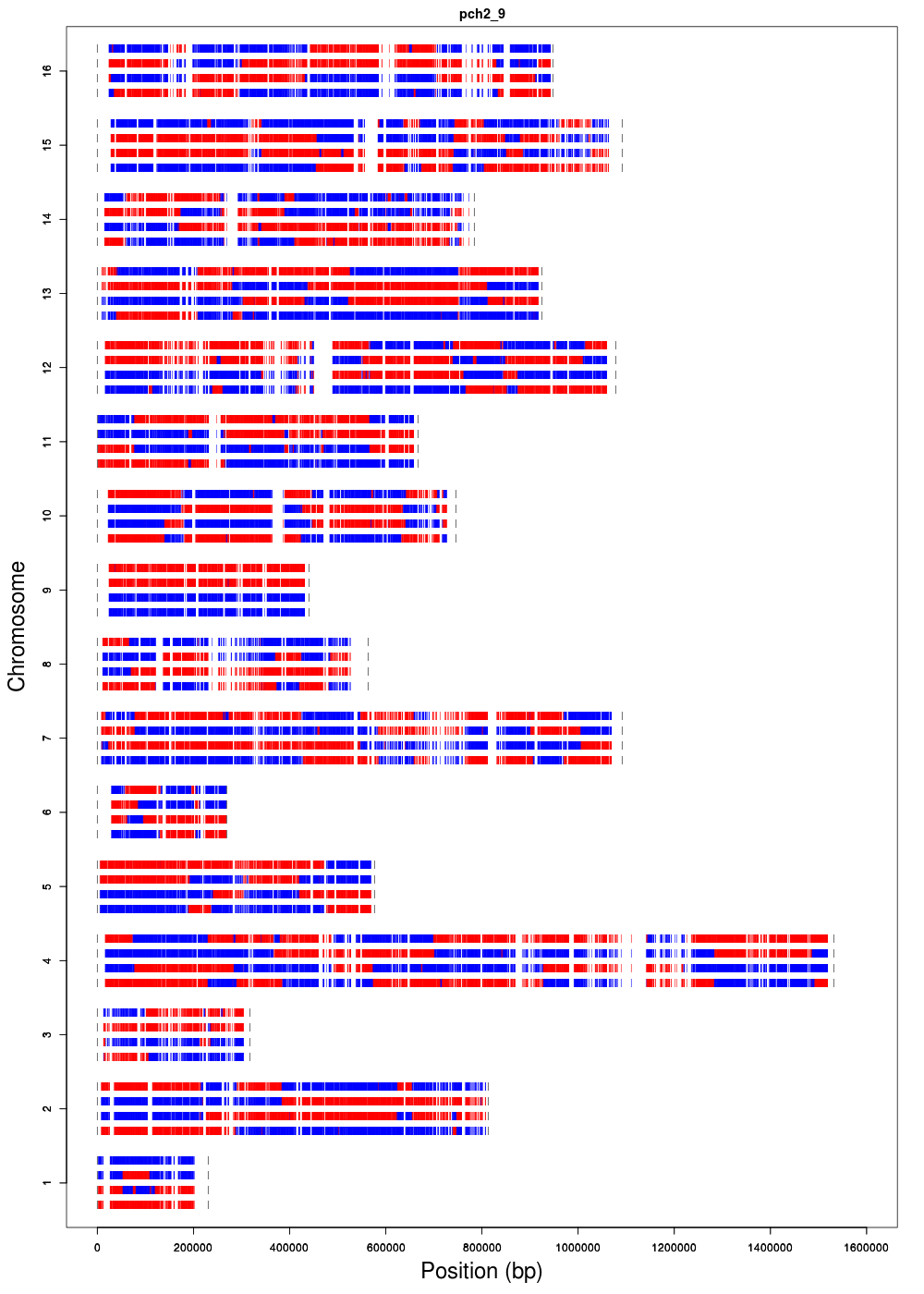

Supplement: Supplementary file 20 [file 1511FileS1.zip › S1 File/pch2_9.tiff]
