# Supplementary material for: Modulating Crossover Frequency and Interference for Obligate Crossovers in Saccharomyces cerevisiae Meiosis
Source: G3 (Bethesda). 2017 Mar 17;7(5):1511–24. doi: 10.1534/g3.117.040071 (PMC5427503; doi:10.1534/g3.117.040071)
Supplement: Supplementary file 21 [file 1511FileS2.docx]

**File S2 Crossover and non-crossover distributions along centromeres and telomeres in wild type, *mlh3Δ, pch2Δ and mlh3Δ pch2Δ.***

The distribution of crossovers near centromere and telomere regions was analyzed in wild type, *mlh3Δ, pch2Δ and mlh3Δ pch2Δ* mutants (Figure S10). Wild-type strain showed reduction in both crossovers (2.3 fold) and non-crossovers (2 fold) within 10 kb of centromeres as observed by Chen *et al.* 2008 and Krishnaprasad *et al.* 2015 (Figure S10A, C). Similar reductions in crossovers were observed for *mlh3Δ* (2 fold), *pch2Δ* (2.4 fold) and *mlh3Δ pch2Δ* (1.8 fold) mutants also. Non-crossovers also showed suppression comparable to wild type within 10 kb of centromeres in *mlh3Δ* (1.5 fold), *pch2Δ* (3 fold) and *mlh3Δ pch2Δ* (1.5 fold). Crossovers in wild type are suppressed by 3.7 fold within 20 kb of telomeres as observed previously by Chen *et al.* 2008 and Krishnaprasad *et al.* 2015 (Figure S10B). The *mlh3Δ* (3.4 fold), *pch2Δ* (3.8 fold) and *mlh3Δ pch2Δ* (3.8 fold) mutants show comparable suppression of crossovers near telomeres. Non-crossovers also showed strong reduction within 20 kb of telomeres in wild type (4.2 fold), *mlh3Δ* (3.2 fold), *pch2Δ* (3.6 fold) and *mlh3Δ pch2Δ* (4.5 fold) (Figure S10D).


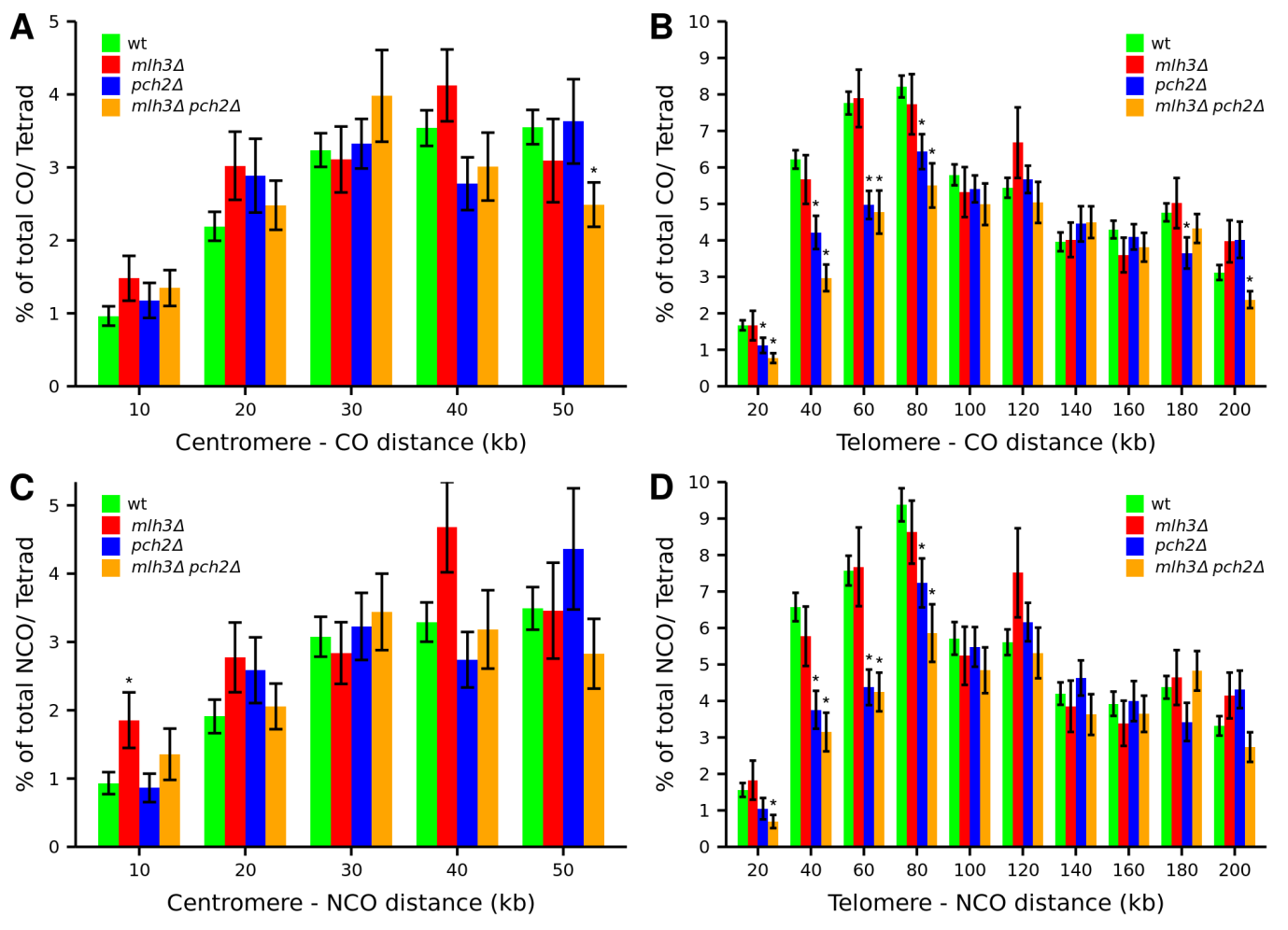


**Figure S10** Crossover and non-crossover distributions near centromeres (Panels A and C) and telomeres (Panels B and D) in wild type, *mlh3Δ, pch2Δ* and *mlh3Δ pch2Δ*. The crossover and non-crossover events / tetrad are shown as a percentage of the total number of events. Statistically significant (*P* <0.05, t-test) differences between the wild type and mutants are shown by asterisk (*).

**Literature cited**

Chen, S. Y., T. Tsubouchi, B. Rockmill, J. S. Sandler, D. R. Richards *et al.*, 2008 Global analysis of the meiotic crossover landscape. Dev. Cell 15**:** 401-415.

Krishnaprasad, G. N., M. T. Anand, G. Lin, M. M. Tekkedil, L. M. Steinmetz *et al.*, 2015 Variation in crossover frequencies perturb crossover assurance without affecting meiotic chromosome segregation in *Saccharomyces cerevisiae*. Genetics 199**:** 399-412.
