# Supplementary material for: Modulating Crossover Frequency and Interference for Obligate Crossovers in Saccharomyces cerevisiae Meiosis
Source: G3 (Bethesda). 2017 Mar 17;7(5):1511–24. doi: 10.1534/g3.117.040071 (PMC5427503; doi:10.1534/g3.117.040071)
Supplement: Supplementary file 22 [file 1511FileS3.docx]

**File S3 Two pathway analysis of interference in wild type, *mlh3Δ, pch2Δ* and *mlh3Δ pch2Δ***

The likelihood ratio test was performed to test if the two pathway model provides a better fit to the crossover data than the one pathway interference alone model. The log_2_ likelihood ratios showed a better fit to wild type (230.7, p = 8.05 x 10^-51^) and *pch2Δ* (65.7, p = 5.5 x 10^-15^) compared to *mlh3Δ* (14.9, p = 5.7 x 10^-4^) and *mlh3Δ pch2Δ* (12.5, p = 1.9 x 10^-3^). This is consistent with the observation that most crossovers in *mlh3Δ* and *mlh3Δ pch2Δ* are made through a single (Mus81-Mms4 dependent) pathway. The two pathway analysis of interference in wild type, *mlh3Δ, pch2Δ* and *mlh3Δ pch2Δ* showed that the proportion of Class I crossovers (interferring) was greatest in wild type (Table S10, Figure S11). *mlh3Δ* and *pch2Δ* showed reduced proportion of interferring crossovers consistent with the results from the one pathway gamma model (Figure 6A,B in manuscript text).

When inter-crossover distances are fit to a gamma distribution, the variability of the distances is inversely proportional to the shape parameter alpha. So a larger alpha indicates less variability in crossover distances, meaning most crossovers are separated by an optimal distance, instead of being clustered (when the shape parameter is 1). *pch2Δ* has a higher alpha value for Class I crossovers compared to that of wild type (6.2 vs 3.1). In *pch2Δ*, there is an increased density of double strand breaks, and we hypothesize that this makes it more likely for the crossovers to occur at an optimal distance. Therefore the variability of inter-crossover distances is reduced, resulting in a higher alpha value for *pch2Δ*. In *mlh3Δ pch2Δ* the alpha for Class I crossovers is equal to 1 (Table S10, Figure S11). In the absence of the major resolvase (Mlh3) in *mlh3Δ pch2Δ*, the crossovers are more clustered (more variability in inter crossover distances as they are randomly produced) and therefore alpha is reduced compared to wild type. An alpha of 1 suggests that crossovers from both pathways in *mlh3Δ pch2Δ* are non-interferring consistent with the results from the one pathway gamma model and the experimental observations (Figure 5B and Figure 6 in the manuscript text).

**Table S10 Proportion of Class I and Class II crossovers inferred from the two pathway analysis of interference.**

|  | Class I crossovers | Class II crossovers | Alpha |
| --- | --- | --- | --- |
|  |  |  |  |
| Wild type | 0.59 | 0.41 | 3.1 |
| *mlh3Δ* | 0.34 | 0.66 | 2.8 |
| *pch2Δ* | 0.22 | 0.78 | 6.3 |
| *mlh3Δ pch2Δ* | 0.5 | 0.5 | 1 |

Class I are interfering crossovers and Class II are non-interfering crossovers. Alpha is the

shape parameter for Class I crossovers.

**
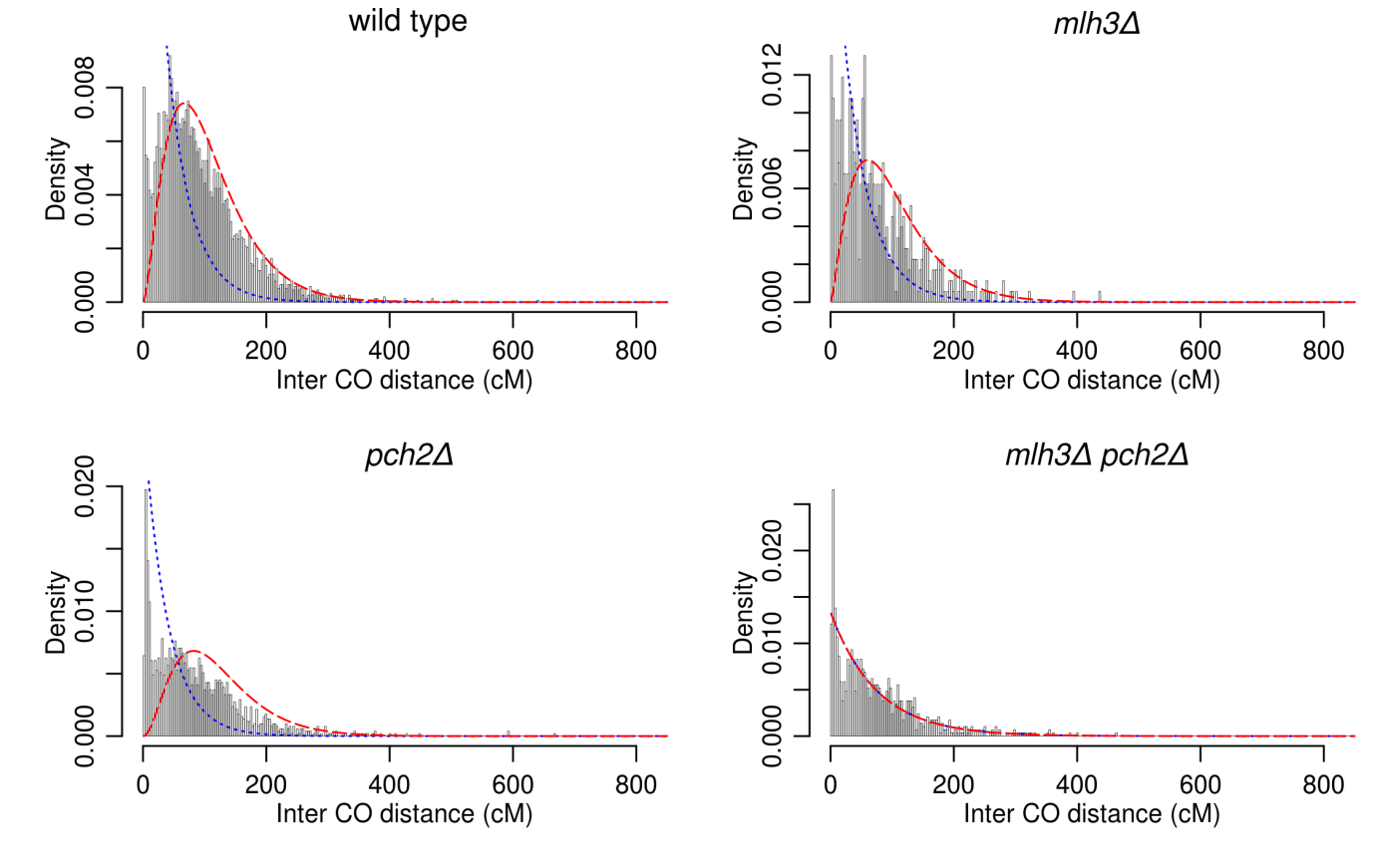
**

**Figure S11** Density plot for inter-crossover distances based on the two pathway model. The blue curve is the probability distribution of non-interfering crossovers. The red curve is the probability distribution of the interfering crossovers. The Y axis is density, which reflects the probability distribution of the inter-crossover distances.
